# Transient-axial-chirality controlled asymmetric rhodium-carbene C(sp^2^)-H functionalization for the synthesis of chiral fluorenes

**DOI:** 10.1038/s41467-020-16098-8

**Published:** 2020-05-12

**Authors:** Kuiyong Dong, Xing Fan, Chao Pei, Yang Zheng, Sailan Chang, Ju Cai, Lihua Qiu, Zhi-Xiang Yu, Xinfang Xu

**Affiliations:** 1 Guangdong Provincial Key Laboratory of Chiral Molecule and Drug Discovery, School of Pharmaceutical Sciences, Sun Yat-sen University, Guangzhou, 510006 China; 2 College of Chemistry, Chemical Engineering and Materials Science, Soochow University, Suzhou, 215123 China; 3 Beijing National Laboratory for Molecular Sciences (BNLMS), Key Laboratory of Bioorganic Chemistry and Molecular Engineering of Ministry of Education, College of Chemistry, Peking University, Beijing, 100871 China

**Keywords:** Asymmetric catalysis, Homogeneous catalysis, Synthetic chemistry methodology, Reaction mechanisms

## Abstract

In catalytic asymmetric reactions, the formation of chiral molecules generally relies on a direct chirality transfer (point or axial chirality) from a chiral catalyst to products in the stereo-determining step. Herein, we disclose a transient-axial-chirality transfer strategy to achieve asymmetric reaction. This method relies on transferring point chirality from the catalyst to a dirhodium carbene intermediate with axial chirality, namely a transient-axial-chirality since this species is an intermediate of the reaction. The transient chirality is then transferred to the final product by C(sp^2^)-H functionalization reaction with exceptionally high enantioselectivity. We also generalize this strategy for the asymmetric cascade reaction involving dual carbene/alkyne metathesis (CAM), a transition-metal-catalyzed method to access chiral 9-aryl fluorene frameworks in high yields with up to 99% *ee*. Detailed DFT calculations shed light on the mode of the transient-axial-chirality transfer and the detailed mechanism of the CAM reaction.

## Abstract (web summary)

The formation of chiral molecules generally relies on direct chirality transfer from catalyst to products. Here, the authors report a strategy based on point chirality transfer from the catalyst to a dirhodium carbene intermediate with axial chirality, which is then transferred to products via C(sp^2^)-H functionalization.

## Introduction

**Fig. 1:**
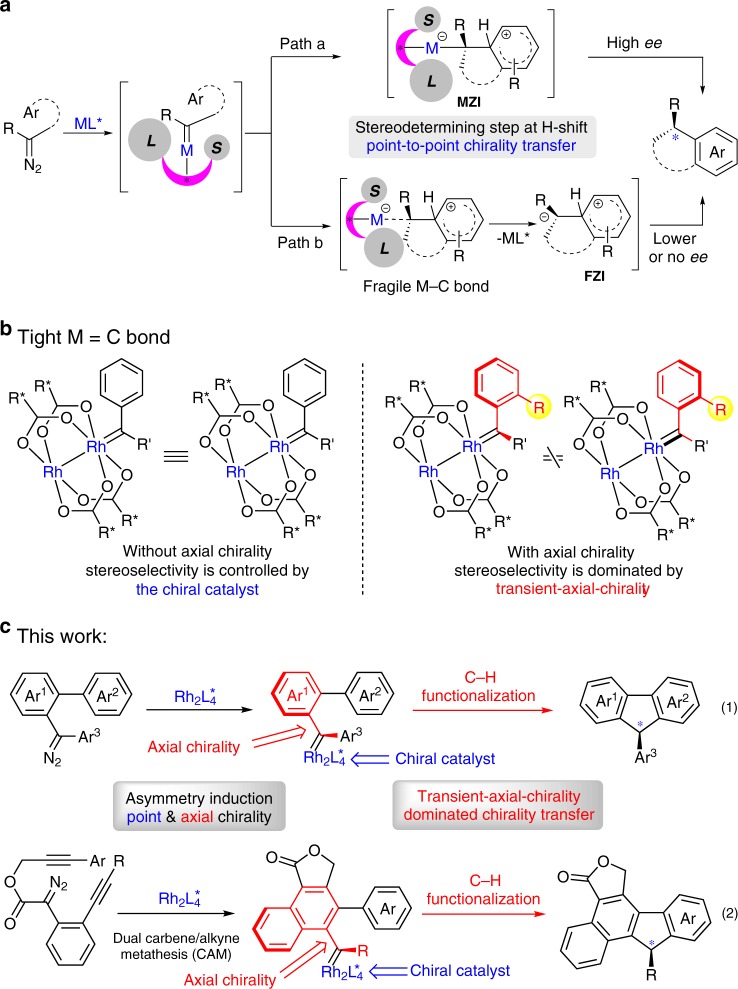
Asymmetry induction in metal-carbene reactions. **a** Asymmetry induction in catalytic metal-carbene C(sp^2^)-H functionalization. **b** Chirality in transient carbene intermediate. **c** This work describes the transient axial-chirality-controlled asymmetric C(sp^2^)–H functionalization. The pink crescent = chiral ligand (L = large group, S = small group). M = metal catalyst. Rh_2_L_4_* = chiral dirhodium complex.

Metal carbene reaction is one of the most versatile methods for the assembly of valuable molecules with structural complexity and diversity^1–8^. In this regard, the pursuit of practical and efficient catalytic approach has been of long-standing appealing, especially the catalytic asymmetric carbene transformations, such as cyclopropanation^9,10^, X–H insertion^11,12^, C–H insertion^13–16^, hydride migration^17^, cycloaddition^18,19^, ylide formation followed by rearrangement^20^ or interception^21^, and others^22–28^. Generally, the asymmetry induction in these metal-carbene reactions heavily relied on the chiral catalyst-associated species, and the asymmetric transfer strategy is a point-to-point chirality transfer manner. For example, the enantioselectivity control in catalytic asymmetric electrophilic aromatic substitution reaction, which happens at the *H*-shift step, is enabled by the point chirality of the catalyst via a metal-associated zwitterionic intermediate^29^ or Wheland-type intermediate^30^ (Fig. 1a, path a, **MZI**, through a M–C single bond). However, in most cases, partially leaving or even dissociation of the metal catalyst could occur to form the free zwitterionic intermediate (Fig. 1a, path b, **FZI**), especially in the case with the neutral dirhodium(II) complex^31^, so the subsequent transformation will not secure the high stereoselectivity. Therefore, it is highly challenging and desirable for the development of stereoselective carbene transformations with efficient and practical strategies.

On the other hand, the axial chirality has been found in a variety of rotation-hindered molecules^32–39^, such as BINAP and BINOL derivatives, which have been widely used as privileged ligands or catalysts in asymmetric catalysis^40–45^. Inspired by the unique structures of these chiral ligands with axial chirality, we hypothesized that, when the dirhodium complexes catalyze the generation of metal-carbene species with steric bulky carbene precursors, such as *ortho*-substituted aryl carbene, a transient axial-chirality will be formed in the corresponding carbene intermediate (Fig. 1b). The axial chirality in the intermediate is called transient axial chirality, considering this chirality will then be transfer to the final product by the followed reaction. In other word, instead of heavily relying on the point chirality of the metal catalyst in the later stereo-determining step (e.g., Fig. 1a), the final chirality transfer from catalyst to product in this mode would be determined by the initially formed axial chirality between the catalyst and the substrate, due to the restricted rotation of these carbene intermediates in the followed transformations. Thus, high enantioselectivity could be envisioned in metal-carbene reactions based on this transient axial-chirality transfer strategy. Herein, we report our recent results by applying this asymmetric transfer strategy, the asymmetric formal C(sp^2^)-H bond insertion reaction of donor/donor carbene through a transient axial-chirality-induced point chirality strategy, which provides a straightforward access to chiral 9-aryl fluorene frameworks with exceptionally high enantioselectivity (Fig. 1c, reaction 1). Moreover, we generalize this strategy for asymmetric cascade reaction, in which the donor/donor carbene is generated in situ via a dual carbene/alkyne metathesis (CAM) process^46–54^, and directly construction of polycyclic 9-aryl fluorenes with high enantioselectivity (Fig. 1c, reaction 2)^55,56^. Considering the chiral fluorenes have found broad applications in various fields, including in pharmaceuticals^57^, photoelectrical materials^58^, and theoretical studies^59^; the present asymmetric reaction could add complementary values in this respect.

## Results

### Reaction optimization

We began our investigation of the asymmetric C–H functionalization reaction with diaryl diazo compound **1a**, which is a typical donor/donor-type carbene precursor, as model substrate (Table 1). To optimize reaction conditions, Rh_2_(*S*-TCPTTL)_4_ was used as the catalyst, and solvents were initially evaluated (entries 1–5), from which we found that reaction in *tert*-butyl methyl ether (TBME) afforded **2a** with the highest selectivity (entry 5, 82% *ee* and 92% yield). Lowering the reaction temperature did not improve the selectivity (entry 6). Further investigation of a variety of dirhodium complexes turned out that the optimum enhancement was achieved by Rh_2_(*S*-TFPTTL)_4_ with four electron-withdrawing fluoro substituents on the phthalimide ring (entry 10, 90% yield, 99% *ee*). It should be mentioned that slowly addition of the rhodium catalyst to the diazo compound is essential in all these reactions to ensure the high enantioselectivity. Notably, in comparison with the analogous thermal induced version, no Buchner reaction product was observed under current conditions^60^.

**Table 1:** Condition optimization.


Entry^a^	Rh(II)	Solvent	Yield (%)^b^	Ee (%)^c^
1	Rh_2_(*S*-TCPTTL)_4_	DCM	91	60
2	Rh_2_(*S*-TCPTTL)_4_	DCE	90	63
3	Rh_2_(*S*-TCPTTL)_4_	Toluene	85	75
4	Rh_2_(*S*-TCPTTL)_4_	Hexane	90	62
5	Rh_2_(*S*-TCPTTL)_4_	TBME	92	82
6^d^	Rh_2_(*S*-TCPTTL)_4_	TBME	80	81
7^d^	Rh_2_(*S*-PTTL)_4_	TBME	82	15
8	Rh_2_(*S*-NTTL)_4_	TBME	90	65
9	Rh_2_(*S*-TBPTTL)_4_	TBME	92	70
10	Rh_2_(*S*-TFPTTL)_4_	TBME	90	99
11	Rh_2_(*S*-PTPA)_4_	TBME	75	5
12	Rh_2_(*S*-PTA)_4_	TBME	72	2
13	Rh_2_(*S*-DOSP)_4_	TBME	90	13
14^d^	Rh_2_(*S*-PTAD)_4_	TBME	92	25


*DCM* dichloromethane, *DCE* 1,2-dichloroethane, *TBME* tert-butyl methyl ether.

^a^The reaction was carried out on a 0.2 mmol scale: **1a** (0.2 mmol), and 4 Å MS (100 mg) in 1.0 mL solvent, was added a solution the catalyst in 1.0 mL of the same solvent *via* syringe pump in 40 min under inert atmosphere.

^b^Isolated yields.

^c^Determined by chiral HPLC analysis, see SI for details.

^d^The reaction was conducted at 0 °C for 24 h.

### Substrate scope

With the optimized reaction conditions in hand for reaction 1, the catalytic asymmetric C–H insertion reaction with a variety of substituted diaryl diazo compounds **1** has been tested, and the results are summarized in Table 2. Unexceptionally, a series of substrates **1a**–**1f** bearing electron-neutral, -deficient, or -rich substitutions on the aromatic ring react smoothly to give the corresponding products in 90–96% yields with 90–99% *ee* (**2a**–**2f**). Substrates with 1-naphthyl, 2-fluorophenyl, and 3-fluorophenyl groups are all tolerated under current conditions, delivering the corresponding products in excellent yields and selectivity (**2g**–**2i**). For the detail of the regioselectivity of **2i**, see Supplementary Fig. 147. Substrates with substitutions on the other aryl group do not affect the high reactivity, the corresponding products **2j** and **2k** are isolated in high yields with 95% and 92% *ee*, respectively.

**Table. 2:**
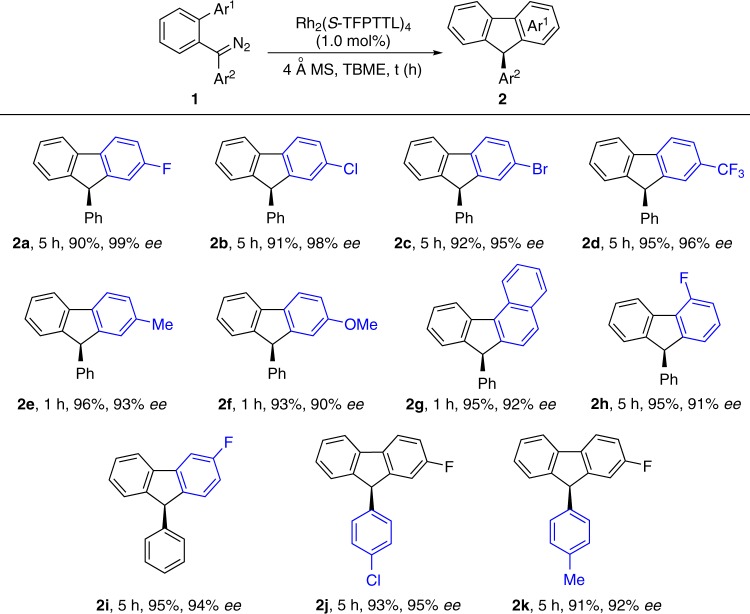
Substrates scope of direct asymmetric C–H functionalization^a^.

^a^The reaction was carried out on a 0.2 mmol scale: Rh_2_(*S*-TFPTTL)_4_ (1.0 mol%) was added as a solution in TBME (1.0 mL) via syringe pump in 40 min under inert atmosphere at room temperature.

Considering the limited accessibility and inherent instability of the precursors of the donor/donor-type carbene species, we intend to utilize the carbene/alkyne metathesis reaction for the generation of the analogous carbene intermediate in situ (Fig. 1c, reaction 2). After a brief optimization, polycyclic fluorene product **4a** was obtained in 90% yield with 92% *ee* from the alkyne-tethered propargyl diazoacetate **3a** in the presence of 1.0 mol% Rh_2_(*S*-TFPTTL)_4_ in TBME at 40 °C, and a detrimental effect on the selectivity by increasing the amounts of the chiral rhodium complex has been observed in this case (see Supplementary Table 1 for details). It is worth mentioning that the only catalyst involved in this four-step cascade transformation is a chiral dirhodium catalyst; and this catalyst is responsible for the observed asymmetry induction with high enantiocontrol in this carbene/alkyne metathesis-aromatic substitution cascade reaction^46–53^. The (*R*)-configuration of the generated chiral center in the 9-aryl fluorene is confirmed by single-crystal X-ray diffraction analysis of its chloro-derivative **4b**, and the configurations of other compounds are assigned by analogy.

Then, the generality of this cascade reaction with a variety of alkyne-tethered diazo compounds **3a–3u** was tested with the optimized reaction conditions (Table 3). A series of substrates **3a**–**3f** bearing electron-neutral, -deficient, or -rich substitutions on the terminating aromatic ring reacted smoothly to give the corresponding products in 63–90% yields with high to excellent enantioselectivity (**4a**–**4f**), and 99% *ee* was observed with CF_3_-substituted product **4d**. The donor/donor carbene intermediate that generated via two carbene/alkyne metathesis process provides a valuable tool for the complement of the selective carbene transformations^61^, which indicates that a different asymmetry induction/transfer model could be involved in this catalytic reaction. Reaction with *meta*-substituted material **3** **g** led to the preferential formation of sterically less encumbered **4** **g** in 53% yield with 97% *ee*, concomitant with a small amount of **4** **g’** in 60% *ee* (see Supplementary Fig. 147 for detail). On the other hand, the *ortho*-substituted diazo compound **3** **h** was found to have very low reactivity, which was caused by the slower C–H insertion step due the steric repulsion between the methyl group and lactone ring (see Supplementary Fig. 148 and Supplementary Table 7 for DFT explanation), and its reaction at 60 °C overnight gave **4** **h** in only 38% yield with <5% *ee* (the low *ee* was possibly due to the racemization of the final product at high temperature, which could be initially generated in higher *ee*). In addition, the naphthyl group was also tolerated for the terminating step, providing the hexacyclic fluorene **4i** in 89% yield with 92% *ee*. In line with our previous work, the alkyl propargyl alcohol derived diazo compound **3j** only led to the β-elimination product **4j** after the first CAM process. The substitution pattern on the second alkyne unit (R, **4k**–**4u**) was then examined, its scope was general regardless of the electronic-influence (**4k**–**4o** and **4r**) or steric-effect (**4o**–**4q**) of the substituted groups on the aromatic ring, and high yields with >90% *ee* were obtained in these reactions. Notably, the alkyl alkyne-tethered substrate **3** **s** was equally reactive and offered the corresponding product in 83% yield with 68% *ee*. In addition, we found that the desired product **4t** could be obtained in 78 yield with 84% *ee* in the case of the TIPS protected alkyne under the conditions with minor optimization. The terminal alkyne was also accommodated to give achiral product (**4** **u**, 44%). To show the synthetic potential of this strategy, a gram-scale reaction of **3c** was performed at 0.5 mol% catalyst loading (Table 3, note b), affording **4c** with comparative results (1.52 g, 89%, 94% *ee*). Derivatizations of these products were also carried out to give corresponding fluorene derivatives with structural complexity, including bromination at the benzyl position, Suzuki coupling with the bromo-derivative, and saponification of the lactone (see Supplementary Methods for the synthesis of **6c**, **7c**, and **8c** for details).

**Table 3:**
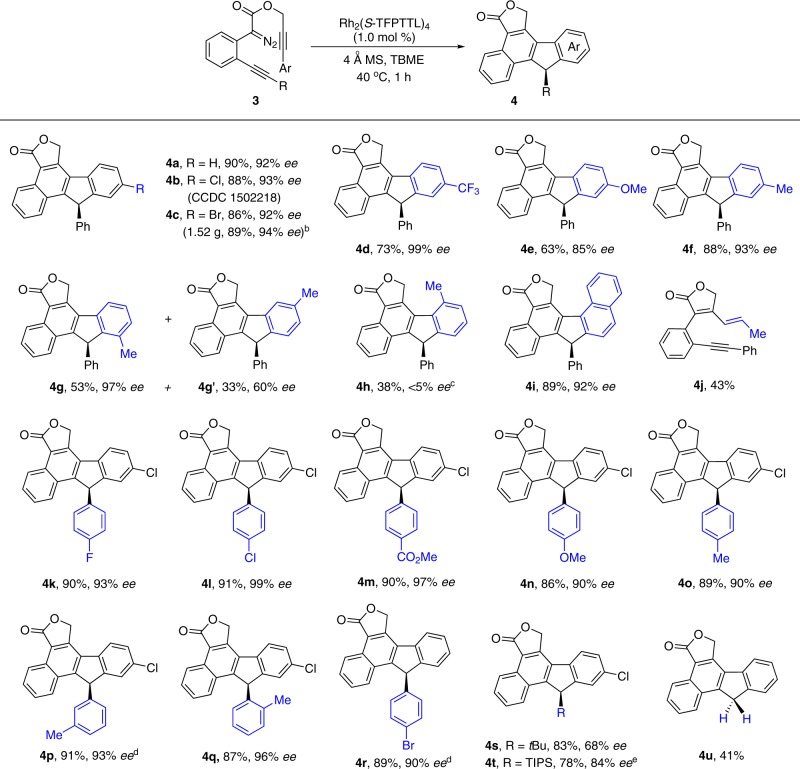
Substrates scope of carbene/alkyne metathesis terminated with asymmetric C–H functionalization^a^.

^a^The reaction was carried out on a 0.2 mmol scale, and Rh_2_(*S*-TFPTTL)_4_ (1.0 mol%) was added as a solution in TBME (1.0 mL) via syringe pump in 40 min under inert atmosphere at 40 °C. The yields are given in isolated yields.

^b^The reaction was carried out on a 4.0 mmol scale with 0.5 mol% catalyst loading.

^c^At 60 °C for 12 h.

^d^Rh_2_(*S*-PTAD)_4_ (1.0 mol%) was used as the catalyst.

^e^The reaction was carried out in cyclohexane:TBME = 1:10 at 30 °C.

### DFT calculations on reaction 1

**Fig. 2:**
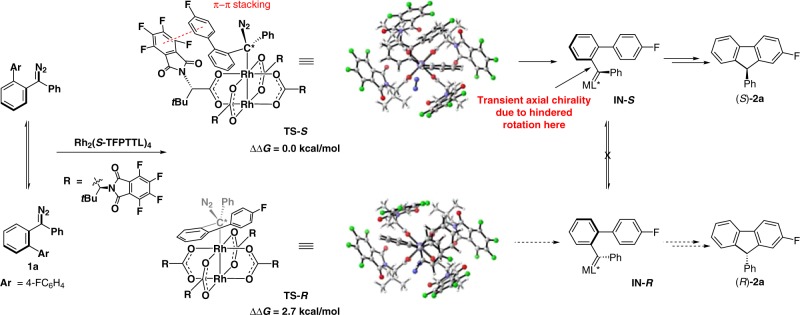
Enantioselectivity transition states of reaction 1 with **1a** to **2a** as example. The forming of the *S*-configuration product **2a** is the favored one (**TS-*S*** is favored over **TS-*R*** by 2.7 kcal/mol. Calculations at SMD(toluene)-M06L-D3/Def2TZVP//PBE-D3/Def2SVP/W06 level).

To support our hypothesis on enantiocontrol by the transient axial chirality, DFT calculations were carried out for reaction 1 (Tables 1 and 2). Substrate **1a** was chosen as the model substrate. Calculation results show that the Rh(II)-catalyst provides a helical chirality environment (Fig. 2). Analogous acceptor-type carbene structures have been investigated by Hashimoto^62^, Charette^63^, Müller^64^, Fox^65^, Davies, and Sigman^66^, independently in their corresponding metal-carbene reactions. Initially, the dirhodium catalyst and the diazo substrate forms a complex reversibly. In the diazo-decomposition transition state, the formed chiral carbon (C*) induces the formation of the axial chirality after the leaving of N_2_ gas. Based on the conformation analysis of diaryl-substituted dirhodium(II) carbene, two chiral conformers were located. DFT calculation results show that forming of the *S*-configuration is the favored one (**TS-*S*** is favored over **TS-*R*** by 2.7 kcal/mol). In the structure of **TS-*S***, remarkable π–π stacking interaction between substrate and chiral ligand can be observed. The diazo decomposition is irreversible and an activation-controlled process (not a diffusion-controlled process, see discussion in the DFT study of reaction 2 below), suggesting that the reaction will favor the *S*-pathway via **TS-*S*** to generate chiral rhodium(II) carbene intermediate **IN-*S***, which has transient axial chirality because the rotation of the C–C bond is hindered by the bulky dirhodium catalysts (we did not calculate this step, but this can be well understood by the DFT study of reaction 2 below, where rotation of the axial chiral intermediate is difficult). This transitent-axial chirality is then transferred to the final product via formal C–H insertion reaction (only one transition state is available).

### DFT calculations on reaction 2

**Fig. 3:**
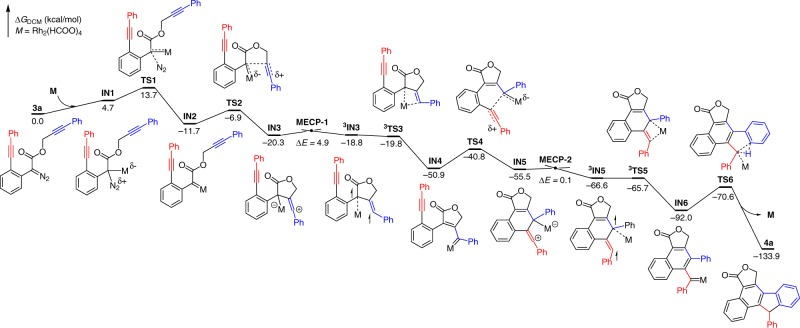
Gibbs energy profile for catalytic cascade reaction 2 with **3a** to **4a** as example. The discovery of carbene–Rh-dimer complex formation via ISC process in CAM process. Calculations at SMD(DCM)-M06L/6-311G(d,p)&SDD//PBE/6-31G(d)&SDD level). MECP minimum energy-crossing point.

Detailed DFT calculations have also been carried out for understanding the mechanism of the key carbene/alkyne metathesis step and the transient axial chirality induced chirality transfer in this asymmetric cascade transformation (Fig. 3).

First, the nonchiral rhodium-catalyzed reaction was calculated for understanding the mechanism, especially the key carbene/alkyne metathesis step. The diazo compound **3a** and Rh_2_(HCOO)_4_ were chosen as a substrate and catalyst of this reaction, respectively. The potential energy surface of racemic model reaction is illustrated in Fig. 3. The reaction starts from the complexation of Rh(II) catalyst with diazo substrate **3a**, leading to the formation of **IN1** (endergonic, by 4.7 kcal/mol). The complex **IN1** undergoes decomposition step via **TS1**, which requires an activation-free energy of 9.0 kcal/mol and is exergonic by 16.4 kcal/mol, giving rise to the corresponding Rh–carbene **IN2** and liberating N_2_ gas. The diazo decomposition has a computed activation-free energy of 13.7 kcal/mol and can be regarded as an activation controlled process, not diffusion-controlled process^67^. Then 5-*exo*-*dig* cyclization, via **TS2** with a computed activation-free energy of 4.8 kcal/mol, converts **IN2** to the zwitterionic intermediate **IN3**, which is a very reactive vinyl cationic species^68^. We could not locate the corresponding intramolecular cyclopropenation transition state from **IN2**, and this can be understood by considering that such reaction would generate a fused cyclopropene with high ring strain. Intermediate **IN3** is subsequently converted to a more stable Rh–carbene complex **IN4** via Rh-1,3-shift^69^. Our calculations indicated that this step involves an intersystem crossing (ISC) process via **MECP-1** to give the triplet species, ^**3**^**IN3**, which then undergoes 1,3-Rh migration via a triplet-transition state ^**3**^**TS3**, finally giving the closed-shell singlet Rh–carbene complex **IN4**. Rhodium–carbene **IN4** then attacks the second tethered alkyne moiety to give the other vinyl cation intermediate **IN5**. This step requires an activation-free energy of 10.1 kcal/mol. Again, **IN4** converted via ISC process to a triplet species ^**3**^**IN5** with a coordinated Rh-dimer. Further coordination of Rh dimer to the carbene site via ^**3**^**TS5** (triplet diradical) to form **IN6** (singlet carbene). Formal carbene C–H insertion reaction needs an activation-free energy of 21.4 kcal/mol. The final step of the catalytic cycle is releasing the product and regenerating the catalyst to continue the next catalytic cycle. The whole catalytic cycle is a downhill process and every step is easy with low activation-free energy. The final C–H insertion could be regarded as the rate-determining step of the whole catalytic process.

The above computed potential energy surface provides us with a complete picture of the present cascade reaction. This mechanism has also been used to explain why the substrate **3** **h** has lower reactivity, mainly due to the slower C–H insertion step (see Supplementary Fig. 148 and Supplementary Table 7 for details). Of the same importance, the discovery of carbene–Rh-dimer complex formation via ISC process is significant for the future understanding of similar processes.

### Stereochemistry discussion for the reaction 2

**Fig. 4:**
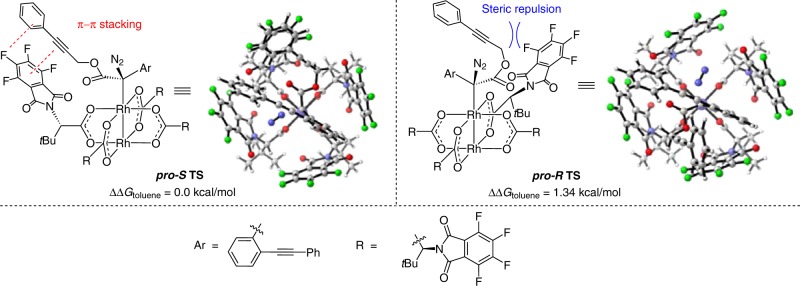
Enantioselectivity transition states of reaction 2 with substrate **3a**. The ***pro*-*R*** **TS** is 1.34 kcal/mol higher than the corresponding ***pro*-*S*** **TS**, suggesting that the experimenal *ee*% value should be up to 80%. Calculations at SMD(toluene)-M06L-D3/Def2TZVP//PBE-D3/Def2SVP/W06 level.

**Fig. 5:**
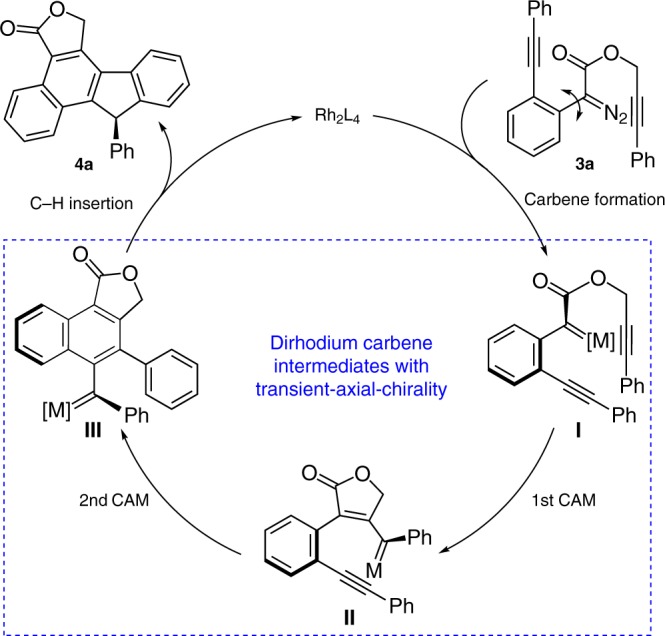
Overview of the catalytic cycle. The dual-carbene/alkyne metathesis process through the formation of the carbene intermediates with axial chirality.

The enantioselectivity of the real system was then investigated based on the above mechanistic insights. After formation of the complex with the diazo substrate, two possible transition states with different orientations were obtained. The ***pro*-*R*** **TS** is 1.34 kcal/mol higher than corresponding ***pro*-*S*** **TS**, suggesting that the experimental *ee*% value should be up to 80% (Fig. 4). We found that in the favored one, the phenylpropargyl group has less steric repulsion with the ligand. This transition state also benefits from weak π–π stacking interaction between phenylpropargyl group and aromatic ring of the ligand. Low-barrier intramolecular CAM process inhibits the dissociation of the catalyst and other side reactions, thus this initially formed transient axial chirality in the metal-carbene intermediate that controlled the following asymmetric transfer processes, and enabling the stereospecificity chirality transfer in the final formal C–H insertion step via an axial-to-point chirality transfer model^70^. According to the above results, the mechanistic proposal of this fluorene ring formation using chiral dirhodium as a catalyst is depicted in Fig. 5. In the initial step, Rh(II)-mediated decomposition of diazo compound **3a** generated the first chiral carbene intermediate **I** with axial chirality due to the hindered rotation of the congested geometry. Followed by a dual-carbene/alkyne metathesis process to form the third axial chirality–carbene-intermediate **III** via the second one **II**. Finally, the catalytic cycle is finished by a selective C(sp^2^)-H insertion with the axial chirality transfer to the carbon chirality center via an axial-induced-point chirality transfer model.

**Fig. 6:**
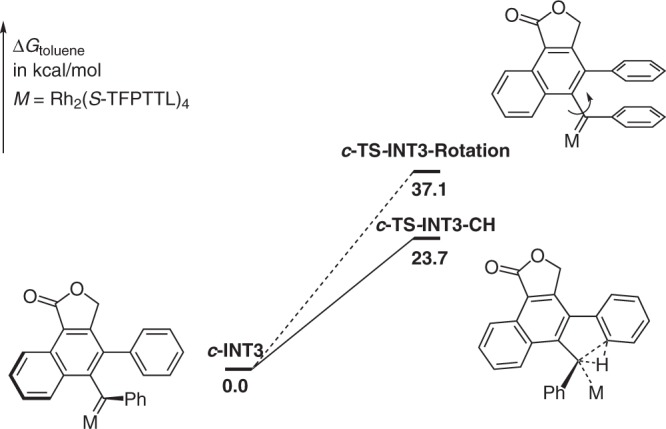
Rotation vs Csp^2^–H insertion. The calculated rotation barrier of ***c*-INT3** is 13.4 kcal/mol higher than C–H insertion reaction.

Considering the C–H insertion is the rate-determine step in this tandem reaction, we calculated the barrier of C–H insertion and rotation of ***c*-INT3** in real system (Fig. 6), the barrier of final C–H insertion reaction is 23.7 kcal/mol, which is consistent with the experimental result. The rotation barrier here is 13.4 kcal/mol higher than C–H insertion reaction, so the chiral transfer is complete in this step and the enantioselective determining step is not C–H insertion step here. For other two optimized key structures of **I** and **II** in real system, see Supplementary Fig. 152 for details.

## Discussion

In summary, we have reported a transient axial-chirality transfer strategy for asymmetric reaction, which takes advantage of the point chirality of the dirhodium catalyst that can be transferred to a Rh–carbene intermediate with transient axial chirality due to the hindered rotation introduced by the bulky catalyst. This transient axial-chirality can ensure the high stereoselectivity in the followed formal Csp^2^–H insertion reaction. We also generalized this strategy for asymmetric cascade reaction via dual-carbene/alkyne metathesis process. DFT calculations reveal the π–π stacking predominant chiral recognition pattern and the unique axial-to-point chirality transfer. Also detailed mechanistic study of the reaction mechanism of two reactions have been investigated, finding that intersystem crossing (ISC) process have been encountered in generating Rh–carbene species. Further applications of this transient axial-chirality transfer concept could be envisioned for other asymmetric reactions.

## Methods

### General methods

See Supplementary Methods for further details.

### Typical procedure for the direct asymmetric C–H functionalization reaction

To a 10-mL oven-dried vial containing a magnetic stirring bar, diazo compound **1** (0.2 mmol), and 4 Å MS (100 mg) in TBME (1.0 mL) and Rh_2_(S-TFPTTL)_4_ (3.0 mg, 1.0 mol%) was added as a solution in TBME (1.0 mL) via a syringe pump over 40 min under argon atmosphere at room temperature. After addition, the reaction mixture was stirred for additional 1–5 h, as indicated, and then purified by column chromatography on silica gel without any additional treatment (hexanes: DCM = 20:1 to 10:1) to give the desired 9-aryl fluorene products **2**.

### Typical procedure for the asymmetric cascade reaction

To a 10-mL oven-dried vial containing a magnetic stirring bar, diazo compound **3** (0.2 mmol), and 4 Å MS (100 mg) in TBME (1.0 mL) and Rh_2_(S-TFPTTL)_4_ (3.0 mg, 1.0 mol%) was added as a solution in TBME (1.0 mL) via a syringe pump over 40 min under argon atmosphere at 40 °C. After addition, the reaction mixture was stirred for additional 20 min, and then purified by column chromatography on silica gel without any additional treatment (hexanes: DCM = 2:1 to 1:1) to give the desired polycyclic products **4**.

## Supplementary information

Supplementary Information

Peer Review File

Description of Additional Supplementary Files

Supplementary Data 1

## Data Availability

Additional data and computational study details supporting the findings described in this manuscript are available in the Supplementary Information. For full characterization data of new compounds and experimental details, see Supplementary Methods and Figures in Supplementary Information file. The X-ray crystallographic coordinates for structure **4b** reported in this study have been deposited at the Cambridge Crystallographic Data Centre (CCDC), under deposition number 1502218. These data can be obtained free of charge from The Cambridge Crystallographic Data Centre via http://www.ccdc.cam.ac.uk/data_request/cif. All other data are available from the authors upon reasonable request.

## References

[1] Ford A (2015). Modern organic synthesis with α−diazocarbonyl compounds. Chem. Rev..

[2] Doyle MP, Ratnikov M, Liu Y (2011). Intramolecular catalytic asymmetric carbon–hydrogen insertion reactions. Synthetic advantages in total synthesis in comparison with alternative approaches. Org. Biomol. Chem..

[3] Lombard FJ, Coster MJ (2015). Rhodium(**ii**)−catalysed intramolecular C–H insertion α− to oxygen: reactivity, selectivity and applications to natural product synthesis. Org. Biomol. Chem..

[4] Fuwa H, Sasaki M (2016). Exploiting ruthenium carbene-catalyzed reactions in total synthesis of marine oxacyclic natural products. Bull. Chem. Soc. Jpn..

[5] Davies HML, Denton JR (2009). Application of donor/acceptor-carbenoids to the synthesis of natural products. Chem. Soc. Rev..

[6] Shim SY, Ryu DH (2019). Enantioselective carbonyl 1,2- or 1,4-addition reactions of nucleophilic silyl and diazo compounds catalyzed by the chiral oxazaborolidinium ion. Acc. Chem. Res..

[7] Herndon JW (2016). The chemistry of the carbon-transition metal double and triple bond: annual survey covering the year 2015. Coord. Chem. Rev..

[8] Candeias NR, Paterna R, Gois PMP (2016). Homologation reaction of ketones with diazo compounds. Chem. Rev..

[9] Lebel H, Marcoux JF, Molinaro C, Charette AB (2003). Stereoselective cyclopropanation reactions. Chem. Rev..

[10] Ebner C, Carreira EM (2017). Cyclopropanation strategies in recent total syntheses. Chem. Rev..

[11] Zhu S, Zhou Q (2012). Transition−metal−catalyzed enantioselective heteroatom–hydrogen bond insertion reactions. Acc. Chem. Res..

[12] Keipour H, Carreras V, Ollevier T (2017). Recent progress in the catalytic carbene insertion reactions into the silicon–hydrogen bond. Org. Biomol. Chem..

[13] Davies HML, Manning JR (2008). Catalytic C–H functionalization by metal carbenoid and nitrenoid insertion. Nature.

[14] Peña−López M, Beller M (2017). Functionalization of unactivated C(sp^3^)−H bonds using metal‐carbene insertion reactions. Angew. Chem. Int. Ed..

[15] Che C, Lo VK, Zhou C, Huang J (2011). Selective functionalisation of saturated C–H bonds with metalloporphyrincatalysts. Chem. Soc. Rev..

[16] Gaillard S, Cazin CSJ, Nolan SP (2012). *N*−heterocyclic carbene gold (I) and copper (I) complexes in C–H bond activation. Acc. Chem. Res..

[17] DeAngelis A, Panish R, Fox JM (2016). Rh-catalyzed intermolecular reactions of α-Alkyl−α-diazo carbonyl compounds with selectivity over β-hydride migration. Acc. Chem. Res..

[18] Davies HML, Lian Y (2012). The combined C–H functionalization/cope rearrangement: discovery and applications in organic synthesis. Acc. Chem. Res..

[19] Xu X, Doyle MP (2014). The [3 + 3]−cycloaddition alternative for heterocycle syntheses: catalytically generated metalloenolcarbenes as dipolar adducts. Acc. Chem. Res..

[20] Zhang ZK (2017). Catalytic asymmetric trifluoromethylthiolation via enantioselective [2,3]−sigmatropic rearrangement of sulfonium ylides. Nat. Chem..

[21] Guo X, Hu W (2013). Novel multicomponent reactions via trapping of protic onium ylides with electrophiles. Acc. Chem. Rev..

[22] Barluenga J, Valdés C (2011). Tosylhydrazones: new uses for classic reagents in palladium‐catalyzed cross‐coupling and metal‐free reactions. Angew. Chem. Int. Ed..

[23] Xia Y, Qiu D, Wang J (2017). Transition−metal−catalyzed cross-couplings through carbene migratory insertion. Chem. Rev..

[24] Hashmi ASK (2008). “High noon” in gold catalysis: carbene versus carbocation intermediates. Angew. Chem. Int. Ed..

[25] Zhang L (2014). A non-diazo approach to α-oxo gold carbenes via gold-catalyzed alkyne oxidation. Acc. Chem. Res..

[26] Fürstner A, Morency L (2008). On the nature of the reactive intermediates in gold‐catalyzed cycloisomerization reactions. Angew. Chem. Int. Ed..

[27] Echavarren AM, Hashmi ASK, Toste FD (2016). Gold catalysis–steadily increasing in importance. Adv. Synth. Catal..

[28] Fructos MR, Diaz−Requejo MM, Perez PJ (2016). Gold and diazo reagents: a fruitful tool for developing molecular complexity. Chem. Commun..

[29] Li YP, Li ZQ, Zhu SF (2018). Recent advances in transition-metal-catalyzed asymmetric reactions of diazo compounds with electron-rich (hetero-) arenes. Tetrahedron Lett..

[30] Chowdhury AD (2018). Electrophilic aromatic substitution over zeolites generates Wheland-type reaction intermediates. Nat. Catal..

[31] Liang Y, Zhou H, Yu ZX (2009). Why is copper(I) complex more competent than dirhodium(II) complex in catalytic asymmetric O−H insertion reactions? A computational study of the metal carbenoid O−H insertion into water. J. Am. Chem. Soc..

[32] Wang Y, Tan B (2018). Construction of axially chiral compounds via asymmetric organocatalysis. Acc. Chem. Res..

[33] Wencel−Delord J, Panossian A, Leroux FR, Colobert F (2015). Recent advances and new concepts for the synthesis of axially stereoenriched biaryls. Chem. Soc. Rev..

[34] Deng R, Xi J, Li Q, Gu ZH (2019). Enantioselective carbon−carbon bond cleavage for biaryl atropisomers synthesis. Chem.

[35] Shen D, Xu Y, Shi SL (2019). A bulky chiral *N*−heterocyclic carbene palladium catalyst enables highly enantioselective suzuki−miyaura cross-coupling reactions for the synthesis of biaryl atropisomers. J. Am. Chem. Soc..

[36] Tan Y (2018). Organocatalytic enantioselective construction of axially chiral sulfone-containing styrenes. J. Am. Chem. Soc..

[37] Luo J (2019). Synthesis of chiral aldehyde catalysts by Pd‐catalyzed atroposelective C−H naphthylation. Angew. Chem. Int. Ed..

[38] Ma C (2019). Design and catalytic asymmetric construction of axially chiral 3,3’−bisindole skeletons. Angew. Chem. Int. Ed..

[39] Qi LW, Mao JH, Zhang J, Tan B (2018). Organocatalytic asymmetric arylation of indoles enabled by azo groups. Nat. Chem..

[40] Giacalone F, Gruttadauria M, Agrigento P, Noto R (2012). Low-loading asymmetric organocatalysis. Chem. Soc. Rev..

[41] Akiyama T, Mori K (2015). Stronger brønsted acids: recent progress. Chem. Rev..

[42] Carroll MP, Guiry PJ (2014). P, N ligands in asymmetric catalysis. Chem. Soc. Rev..

[43] Fu W, Tang W (2016). Chiral monophosphorus ligands for asymmetric catalytic reactions. ACS Catal..

[44] Xie JH, Zhou QL (2008). Chiral diphosphine and monodentate phosphorus ligands on a spiro scaffold for transition-metal-catalyzed asymmetric reactions. Acc. Chem. Res..

[45] Gao D, Gu Q, Zheng C, You S (2017). Synthesis of planar chiral ferrocenes via transition-metal-catalyzed direct C–H bond functionalization. Acc. Chem. Res..

[46] Pei C, Zhang C, Qian Y, Xu X (2018). Catalytic carbene/alkyne metathesis (CAM): a versatile strategy for alkyne bifunctionalization. Org. Biomol. Chem..

[47] Archambeau A, Miege F, Meyer C, Cossy J (2015). Intramolecular cyclopropanation and C–H insertion reactions with metal carbenoids generated from cyclopropenes. Acc. Chem. Res..

[48] Torres Ò, Pla−Quintana A (2016). The rich reactivity of transition metal carbenes with alkynes. Tetrahedron Lett..

[49] González−Rodríguez C, Suárez JM, Varela JA, Saá CC (2015). Nucleophilic addition of amines to ruthenium carbenes: *ortho*‐(alkynyloxy)benzylamine cyclizations towards 1,3‐benzoxazines. Angew. Chem. Int. Ed..

[50] Ni Y, Montgomery J (2016). Synthetic studies and mechanistic insight in nickel-catalyzed [4+ 2+ 1] cycloadditions. J. Am. Chem. Soc..

[51] Le PQ, May JA (2015). Hydrazone-initiated carbene/alkyne cascades to form polycyclic products: ring-fused cyclopropenes as mechanistic intermediates. J. Am. Chem. Soc..

[52] Yao R, Rong G, Yan B, Qiu L, Xu X (2016). Dual-functionalization of alkynes via copper−catalyzed carbene/alkyne metathesis: a direct access to the 4-carboxyl quinolines. ACS Catal..

[53] Dong K (2018). Selective C(sp^3^)−H bond insertion in carbene/alkyne metathesis reactions. Enantioselective construction of dihydroindoles. ACS Catal..

[54] Torres Ò, Roglans A, Pla-Quintana A (2016). An enantioselective cascade cyclopropanation reaction catalyzed by rhodium(I): asymmetric synthesis of vinylcyclopropanes. Adv. Synth. Catal..

[55] Sun F, Zeng M, Gu Q, You S (2009). Enantioselective synthesis of fluorene derivatives by chiral phosphoric acid catalyzed tandem double Friedel–Crafts reaction. Chem. Eur. J..

[56] Kim J, Ohk Y, Park SH, Jung Y, Chang S (2011). Intramolecular aromatic carbenoid insertion of biaryldiazoacetates for the regioselective synthesis of fluorenes. Chem. Asian J..

[57] Veiga MI (2016). Globally prevalent PfMDR1 mutations modulate *Plasmodium falciparum* susceptibility to artemisinin-based combination therapies. Nat. Commun..

[58] Beaupré S, Boudreault PL, Leclerc M (2010). Solar-energy production and energy-efficient lighting: photovoltaic devices and white-light-emitting diodes using poly(2,7−fluorene), poly(2,7−carbazole), and poly(2,7−dibenzosilole) derivatives. Adv. Mater..

[59] Tian Y (2014). Design and synthesis of new stable fluorenyl-based radicals. J. Am. Chem. Soc..

[60] Liu Z (2015). Transition‐metal‐free intramolecular carbene aromatic substitution/büchner reaction: synthesis of fluorenes and [6, 5, 7] benzo‐fused rings. Angew. Chem. Int. Ed..

[61] Soldi C (2014). Enantioselective intramolecular C–H insertion reactions of donor–donor metal carbenoids. J. Am. Chem. Soc..

[62] Goto T (2011). Highly enantioselective cyclopropenation reaction of 1‐alkynes with α‐alkyl‐α‐diazoesters catalyzed by dirhodium (II) carboxylates. Angew. Chem. Int. Ed..

[63] Lindsay VNG, Lin W, Charette AB (2009). Experimental evidence for the all-up reactive conformation of chiral rhodium(II) carboxylate catalysts: enantioselective synthesis of *cis*-cyclopropane α-amino acids. J. Am. Chem. Soc..

[64] Ghanem, A., Gardiner, M. G., Williamson, R. M. & Müller, P. First X‐ray structure of a *N*‐naphthaloyl‐tethered chiral dirhodium(II) complex: structural basis for tether substitution improving asymmetric control in olefin cyclopropanation. *Chem. Eur. J.* **16**, 3291–3295 (2010).. 10.1002/chem.200903231 20175164

[65] DeAngelis A, Dmitrenko O, Yap GPA, Fox JM (2009). Chiral crown conformation of Rh_2_(S−PTTL)_4_: enantioselective cyclopropanation with α-alkyl−α-diazoesters. J. Am. Chem. Soc..

[66] Liao K (2018). Site-selective carbene-induced C–H functionalization catalyzed by dirhodium tetrakis (triarylcyclopropanecarboxylate) complexes. ACS Catal..

[67] Limanto J (2002). Intramolecular cycloadditions of cyclobutadiene with olefins. J. Am. Chem. Soc..

[68] Wang Y, Cai P-J, Yu Z-X (2020). Mechanistic study on gold-catalyzed cycloisomerization of dienediynes involving aliphatic C-H functionalization and inspiration for developing a new strategy to access polycarbocycles. J. Am. Chem. Soc..

[69] Kang C (2018). Living metathesis and metallotropy polymerization gives conjugated polyenynes from multialkynes: how to design sequence-specific cascades for polymers. J. Am. Chem. Soc..

[70] Gheewala CD, Collins BE, Lambert TH (2016). An aromatic ion platform for enantioselective Brønsted acid catalysis. Science.

