# Supplementary material for: Transient-axial-chirality controlled asymmetric rhodium-carbene C(sp2)-H functionalization for the synthesis of chiral fluorenes
Source: Nat Commun. 2020 May 12;11:2363. doi: 10.1038/s41467-020-16098-8 (PMC7217916; doi:10.1038/s41467-020-16098-8)
Supplement: Supplementary file 1 — Supplementary Information [file 41467_2020_16098_MOESM1_ESM.pdf]

Supplementary Information

Transient-axial-chirality controlled asymmetric rhodium-carbene C(sp^2)-H functionalization for the synthesis of chiral fluorenes

Kuiyong Dong *et al*

Supplementary methods

General Information

All reactions were performed in 10 ml oven-dried glassware under atmosphere of argon. Analytical thin-layer chromatography was performed using glass plates pre-coated with 200-300 mesh silica gel impregnated with a fluorescent indicator (254 nm). Flash column chromatography was performed using silica gel (300-400 mesh). 1 H NMR and 13C NMR spectra were recorded in CDCl3 or DMSO-6d on a 400 MHz spectrometer; chemical shifts are reported in ppm with the solvent signals as reference, and coupling constants (J) are given in Hertz. The peak information is described as: br = broad, s = singlet, d = doublet, t = triplet, q = quartet, m = multiplet, comp = composite. Enantioselectivity was determined on HPLC using Chiralpak IA-3 and IB-3 column. High-resolution mass spectra (HRMS) were recorded on a commercial apparatus (ESI Source) and (CI Source).

Supplementary Table 1. Condition optimization studies

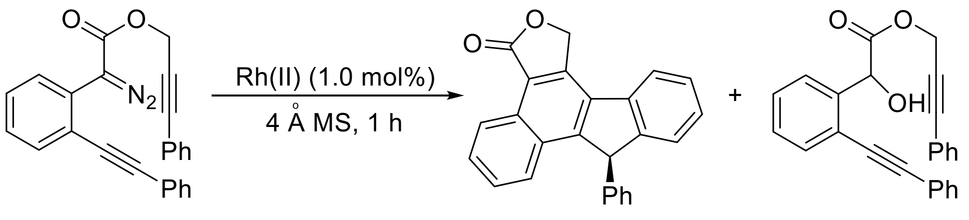

Entry[a]	Cat.	Solvent	T (°C)	Yield (%)[b]	*ee* (%)[c]
1	Cu(hfacac)$_2$	DCM	rt	78	-
2	$Rh_2(OAc)_4$	DCM	rt	35(42)[d]	-
3	$Rh_2(OAc)_4$	DCM	40	82	-
4	Rh_2(S-PTTL)$_4$	DCM	rt	78	30
5	Rh_2(S-PTTL)$_4$	DCM	-20	66	39
6	Rh_2(S-PTTL)$_4$	DCM	40	81	59
7	Rh_2(S-PTTL)$_4$	DCE	40	83	55
8	Rh_2(S-PTTL)$_4$	toluene	40	70	75
9	Rh_2(S-PTTL)$_4$	$CF_3C_6H_5$	40	78	64
10	Rh_2(S-PTTL)$_4$	TBME	40	77	80
11	Rh_2(S-PTTL)$_4$	DMB	40	81	75
12	Rh_2(S-PTPA)$_4$	TBME	40	76	36
13	Rh_2(S-PTA)$_4$	TBME	40	82	8
14	Rh_2(S-NTTL)$_4$	TBME	40	83	73
15	Rh_2(S-TCPTTL)$_4$	TBME	40	81	58
16	Rh_2(S-TFPTTL)$_4$	TBME	40	90	92
17[e]	Rh_2(S-TFPTTL)$_4$	TBME	40	85	91
18[f]	Rh_2(S-TFPTTL)$_4$	TBME	40	78	70
19	Rh_2(S-TBPTL)$_4$	TBME	40	85	40
20	Rh_2(S-DOSP)$_4$	TBME	40	87	52
21	Rh_2(S-PTAD)$_4$	TBME	40	91	85

[a]The reaction was carried out on 0.2 mmol scale. The dirhodium catalyst was added as a solution in 1.0 mL of the same solvent *via* syringe pump in 40 min under inert atmosphere, and the reaction mixture was stirred for additional 20 min before purification *via* column chromatography.

[b]Isolated yields of **4a**.

[c]Determined by chiral HPLC analysis.

[d]In the absence of 4Å MS and data in parentheses is yield of **5a**.

[e]The **3a** in TBME (1.0 mL) was added to a solution of Rh_2(S-TFPTTL)$_4$ (1.0 mol%) in 40 min.

[f]The **3a** in TBME (1.0 mL) was added to a solution of Rh_2(S-TFPTTL)$_4$ (5.0 mol%) in 40 min.

DCM = dichloromethane. TBME = *tert*-butyl methyl ether. DMB = 2,2-dimethyl butane.

General Procedure for the Preparation of Diazo Compounds 1

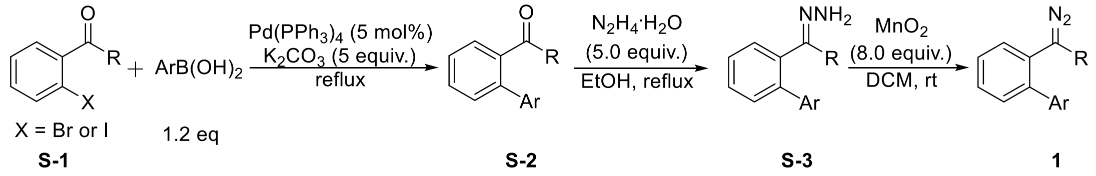

*Synthesis of **S-2**[1]:* To a 50-mL oven-dried flask containing a magnetic stirring bar **S-1**[2] (5.0 mmol), aryl boronic acid (6.0 mmol), K_2CO_3 (3.4 g, 25 mmol), and $Pd(PPh_3)_4$ (238 mg, 5.0 mol%), in toluene/EtOH/H_2O (16 mL/2 mL/2 mL) was stirred under N_2 protected refluxing for 5 hours. Then the reaction mixture was quenched by adding brine (15 mL) and extracted with ethyl acetate (15 mL X 3). The combined organic layers were dried over Na_2SO_4, and solvent was evaporated in *vacuo* after filtration, and the residue was purified by column chromatography on silica gel (Hexanes:EtOAc = 50:1) to provide the corresponding coupling products **S-2** (> 90% yield).

*Synthesis of **S-3**[3]:* To a 50-mL oven-dried flask containing a magnetic stirring bar and compound **S-2** (4.0 mmol) in ethanol (10 ml), was added $N_2H_4{\cdot}H_2O$ (1.0 g, 20 mmol). The solution was stirred under refluxing. After consumption of the material (monitored by TLC, EtOAc:PE = 30:1), the solvent was evaporated under reduced pressure and the resulting crude product extracted with ethyl acetate (3×15 mL). The combined organic layers were dried over Na_2SO_4, and solvent was evaporated in *vacuo* after filtration to give the crude products **S-3**, which were directly used for the next step without purification.

*Synthesis of **1**[2]:* To a 50-mL oven-dried flask containing a magnetic stirring bar and the above obtained **S-3** in dry DCM (20.0 ml), was added MnO_2 (2.7 g, 8.0 eq) slowly, After consumption of the material (monitored by TLC, EtOAc:PE = 5:1), the reaction mixture was filtered through Celtie and rinsed with DCM (20 mL). The combined organic layers were dried over Na_2SO_4, and solvent was evaporated in *vacuo* after filtration. The residue was purified by pretreated column chromatography on silica gel (Hexanes with 1.0 % Et_3N) to provide the corresponding pure diazo compounds **1** as red solid (> 80% yield).

Characterization of Diazo Compounds 1.

2-[Diazo(phenyl)methyl]-4'-fluoro-1,1'-biphenyl (1a)

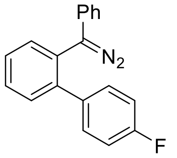

Red solid, ^{1}H NMR (400 MHz, $CDCl_3$) (δ, ppm) δ 7.61 – 7.54 (m, 1H), 7.45 (comp, 3H), 7.39 – 7.28 (comp, 4H), 7.16 – 6.99 (comp, 5H); ^{13}C NMR (100 MHz, $CDCl_3$) (δ, ppm) δ 162.2 (d, *J* = 246.5 Hz), 141.0, 136.5, 131.6, 131.2

(d, J = 6.4 Hz), 130.0 (d, J = 8.1 Hz), 129.1, 128.6, 128.2, 126.6, 124.4, 122.8), 115.9, 115.6. HRMS (TOF MS CI$^+$) calculated for $C_{19}H_{14}FN_2$ $[M + H]^+$, 289.1141; found, 289.1149.

4'-Chloro-2-[diazo(phenyl)methyl]-1,1'-biphenyl (1b)

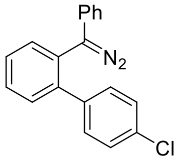

Red solid, ^{1}H NMR (400 MHz, $CDCl_3$) (δ, ppm) δ 7.59 (m, 1H), 7.50 – 7.44 (comp, 3H), 7.33 (comp, 6H), 7.12 (t, J = 7.4 Hz, 1H), 7.09 – 7.04 (m, 2H); ^{13}C NMR (100 MHz, $CDCl_3$) δ 140.7, 138.9, 133.4, 131.5, 131.2, 131.1, 129.6, 129.1, 129.0, 128.6, 128.4, 126.5, 124.4, 122.8. HRMS (TOF MS CI$^+$) calculated for $C_{19}H_{14}ClN_2$ $[M + H]^+$, 305.0846; found, 305.0837.

4'-Bromo-2-[diazo(phenyl)methyl]-1,1'-biphenyl (1c)

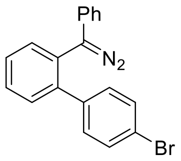

Red solid, ^{1}H NMR (400 MHz, $CDCl_3$) (δ, ppm) δ 7.58 (m, 1H), 7.47 (comp 5H), 7.34 (t, J = 7.8 Hz, 2H), 7.24 (d, J = 8.4 Hz, 2H), 7.11 (t, J = 7.4 Hz, 1H), 7.04 (d, J = 7.6 Hz, 2H); ^{13}C NMR (100 MHz, $CDCl_3$) (δ, ppm) δ 140.8, 139.4, 132.0, 131.5, 131.3, 131.1, 130.0, 129.2, 128.6, 128.4, 126.5, 124.5, 122.8, 121.6. HRMS (TOF MS CI$^+$) calculated for $C_{19}H_{14}BrN_2$ $[M + H]^+$, 349.0340; found, 349.0346.

2-[Diazo(phenyl)methyl]-4'-(trifluoromethyl)-1,1'-biphenyl (1d)

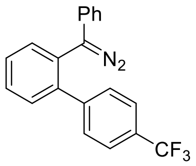

Red solid, ^{1}H NMR (400 MHz, $CDCl_3$) (δ, ppm) δ 7.66 – 7.58 (comp, 3H), 7.55 – 7.44 (comp, 5H), 7.33 (m, 2H), 7.11 (t, J = 7.4 Hz, 1H), 7.08 – 7.02 (m, 2H); ^{13}C NMR (100 MHz, $CDCl_3$) (δ, ppm) δ 144.2, 140.5, 131.3, 131.3, 131.2, 129.2, 129.1, 129.0(q, J = 200.4 Hz), 128.8, 128.7, 128.4, 126.7, 125.8(q, J = 14.8 Hz), 124.6, 122.9. HRMS (TOF MS CI$^+$) calculated for $C_{20}H_{14}F_3N_2$ $[M + H]^+$, 339.1109; found, 339.1117.

2-[Diazo(phenyl)methyl]-4'-methyl-1,1'-biphenyl (1e)

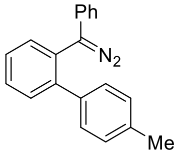

Red solid, ^{1}H NMR (400 MHz, $CDCl_3$) (δ, ppm) δ 7.68 – 7.60 (m, 1H), 7.59 – 7.46 (comp, 3H), 7.45 – 7.32 (comp, 4H), 7.30 – 7.23 (m, 2H), 7.21 – 7.10 (comp, 3H), 2.46 (s, 3H); ^{13}C NMR (100 MHz, $CDCl_3$) (δ, ppm) δ 142.0, 137.6, 136.9, 131.9, 131.3, 131.1, 129.5, 129.0, 128.4, 128.1, 127.8, 126.4, 124.2, 122.8, 21.3. HRMS (TOF MS CI$^+$) calculated for $C_{20}H_{17}N_2$ $[M + H]^+$, 258.1392; found, 258.1385.

2-(Diazo(phenyl)methyl)-4'-methoxy-1,1'-biphenyl (1f)

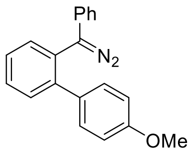

Red solid, ^{1}H NMR (400 MHz, $CDCl_3$) (δ, ppm) δ 7.60 (m, 1H), 7.45 (comp, 3H), 7.34 (comp, 4H), 7.10 (comp, 3H), 6.99 – 6.87 (m, 2H), 3.83 (s, 3H); ^{13}C NMR (100 MHz, $CDCl_3$) δ 158.9, 141.7, 132.7, 131.9, 131.2, 131.1, 129.4, 129.0, 128.4, 127.6, 126.3, 124.2, 122.8, 114.2, 55.3. HRMS (TOF MS CI^+) calculated for $C_{20}H_{17}ON_2$ $[M + H]^+$, 301.1341; found, 301.1341.

1-{2-[Diazo(phenyl)methyl]phenyl}naphthalene (1g)

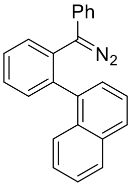

Pink solid, ^{1}H NMR (400 MHz, $CDCl_3$) (δ, ppm) δ 7.90 (m, 2H), 7.69 (t, *J* = 9.4 Hz, 2H), 7.61 – 7.45 (comp, 5H), 7.43 – 7.35 (m, 2H), 7.28 (m, 2H), 7.11 – 7.01 (m, 3H); ^{13}C NMR (100 MHz, $CDCl_3$) (δ, ppm) δ 140.1, 138.0, 133.8, 132.5, 131.4, 131.3, 130.1, 128.8, 128.5, 128.4, 128.2, 128.0, 127.8, 126.9, 126.1, 125.9, 125.7, 125.6, 124.3, 123.2. HRMS (TOF MS CI^+) calculated for $C_{23}H_{17}N_2$ $[M + H]^+$, 321.1392; found, 321.1386.

2-[Diazo(phenyl)methyl]-2'-fluoro-1,1'-biphenyl (1h)

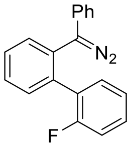

Red solid, ^{1}H NMR (400 MHz, $CDCl_3$) (δ, ppm) δ 7.61 – 7.55 (m, 1H), 7.51 – 7.44 (comp, 3H), 7.28 (comp, 4H), 7.18 – 7.13 (m, 1H), 7.11 – 7.02 (comp, 4H); ^{13}C NMR (101 MHz, $CDCl_3$) (δ, ppm) δ 159.5 (d, *J* = 247.1 Hz), 131.9(d, *J* = 1.1 Hz), 131.3, 131.0 (d, *J* = 3.2 Hz), 130.5, 129.5, 129.4, 128.9, 128.7, 128.2, 128.0, 124.5 (d, *J* = 3.6 Hz), 124.4, 123.2, 116.1,115,9. HRMS (TOF MS CI^+) calculated for $C_{19}H_{14}FN_2$ $[M + H]^+$, 289.1141; found, 289.1143.

2-[Diazo(phenyl)methyl]-3'-fluoro-1,1'-biphenyl (1i)

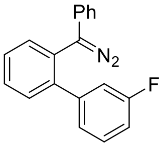

Red solid, ^{1}H NMR (400 MHz, $CDCl_3$) (δ, ppm) δ 7.59 (m, 1H), 7.51 – 7.44 (comp, 3H), 7.39 – 7.30 (comp, 3H), 7.17 (d, *J* = 7.7 Hz, 1H), 7.10 (comp, 4H), 7.02 (m, 1H); ^{13}C NMR (100 MHz, $CDCl_3$) (δ, ppm) δ 163.1 (d, *J* = 246.0 Hz), 142.7 (d, *J* = 7.7 Hz), 140.5, 131.4, 131.2, 131.0, 130.3, 130.2, 129.1, 128.5 (d, *J* = 2.3 Hz), 126.6, 124.5, 124.1 (d, *J* = 2.8 Hz), 123.0, 115.3 (d, *J* = 21.9 Hz), 114.2 (d, *J* = 21.1 Hz). HRMS (TOF MS CI^+) calculated for $C_{19}H_{14}FN_2$ $[M + H]^+$, 289.1141; found, 289.1137.

2-[(4-Chlorophenyl)(diazo)methyl]-4'-fluoro-1,1'-biphenyl (1j)

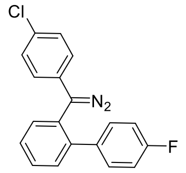

Red solid, ^{1}H NMR (400 MHz, $CDCl_3$) (δ, ppm) δ 7.51 (m, 1H), 7.47 – 7.39 (comp, 3H), 7.33 – 7.20 (comp, 4H), 7.03 (comp, 2H), 6.91 (d, J = 8.6 Hz, 2H); ^{13}C NMR (100 MHz, $CDCl_3$) (δ, ppm) δ 162.3 (d, J = 247.2 Hz), 131.3, 131.1, 130.3, 129.9 (d, J = 8.1 Hz), 129.8, 129.3, 128.9, 128.4, 126.1, 124.4, 123.8, 115.8 (d, J = 21.5 Hz). HRMS (TOF MS CI^+) calculated for $C_{19}H_{13}ClFN_2$ $[M + H]^+$, 323.0751; found, 323.0743.

2-[Diazo(*p*-tolyl)methyl]-4'-fluoro-1,1'-biphenyl (1k)

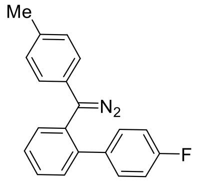

Red solid, ^{1}H NMR (400 MHz, $CDCl_3$) (δ, ppm) δ 7.57 – 7.51 (m, 1H), 7.42 (comp, 3H), 7.35 – 7.29 (m, 2H), 7.15 (d, J = 8.3 Hz, 2H), 7.05 (m, 2H), 6.95 (d, J = 8.2 Hz, 2H), 2.35 (s, 3H); ^{13}C NMR (100 MHz, $CDCl_3$) (δ, ppm) δ 162.2 (d, J = 246.3 Hz), 140.8, 136.5 (d, J = 3.3 Hz), 134.2, 131.2, 131.0, 130.0, 129.93, 129.92, 128.3 (d, J = 23.4 Hz), 128.2, 126.89, 115.8 (d, J = 21.5 Hz), 21.0 (s). HRMS (TOF MS CI^+) calculated for $C_{20}H_{16}FN_2$ $[M + H]^+$, 303.1298; found, 303.1289.

General Procedure for the Preparation of Diazoacetates 3.

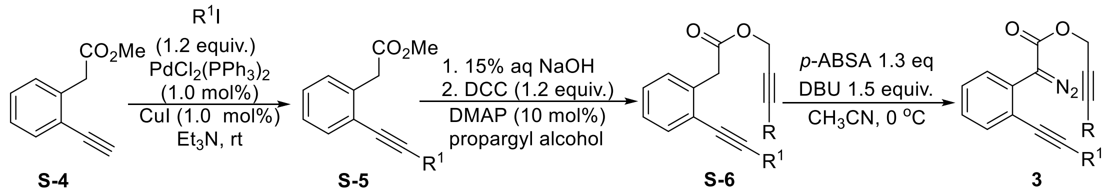

<u>*Synthesis of **S-5**[5]:*</u> To a 50-mL oven-dried flask containing a magnetic stirring bar, **S-4**[4] (3.0 mmol, 523 mg),[2] $PdCl_2(PPh_3)_2$ (1.0 mol%, 21 mg), CuI (1.0 mol%, 6 mg), Et_3N (10 mL) and aryl iodide (3.6 mmol) were added in sequence. The mixture was stirred at room temperature overnight. Then ether (10 mL) and H_2O (10 mL) were added to quench the reaction, and the aqueous layer was extracted with ether (10 mL). The combined organic layers were dried over Na_2SO_4. The solvent was evaporated in *vacuo* after filtration. The residue was purified by column chromatography on silica gel (Hexanes:EtOAc = 20:1) to give the corresponding coupling products **S-5** as pale yellow oil (> 80% yield).

<u>*Synthesis of **S-6**[6]:*</u> To a 50-mL oven-dried flask containing a magnetic stirring bar and compound **S-5** (2.0 mmol) in THF (5.0 ml), was added 15% NaOH (10 ml). The solution was stirred at room temperature for 5 h. After consumption of the material (monitored by TLC), the mixture was

acidified with 1N HCl solution (to PH~3.0), The mixture was extracted with DCM (10 mL X 2) and the combined organic extracts was dried over Na_2SO_4, and solvent was evaporated in *vacuo* after filtration to give a pale yellow solid, this solid was directly used for the next step without purification.

To a 50-mL oven-dried flask containing a magnetic stirring bar, the above obtained acid, propargyl alcohol (2.4 mmol), and DMAP (4-dimethylaminopyridine, 24.4 mg, 0.2 mmol) in DCM (10 mL), was added DCC (dicyclohexylcarbodiimide, 0.63 g, 2.4 mmol) in batches at 0 ^{o} C, and the reaction mixture was stirred at room temperature overnight. After that, the reaction mixture was filtered through Celtie and rinsed with EtOAc (10 mL), and the filtrates were combined. After evaporating the solvents, the residue was purified by column chromatography on silica gel (Hexanes:EtOAc = 20:1) to provide the corresponding esters **S-6** as white solid (> 90% yield).

*Synthesis of **3:*** To a 50-mL oven-dried flask containing a magnetic stirring bar, esters **S-6** (1.5 mmol) and *p*-ABSA (4-Acetamidobenzenesulfonyl azide, 468 mg, 1.95 mmol, 1.3 eq) in CH_3CN (10 mL) was added DBU (1,8-diazabicyclo[5.4.0]undec-7-ene, 342 mg, 2.25 mmol, 1.5 eq) in CH_3CN (2.0 mL) slowly at 0 ^oC, and the resulting reaction mixture was stirred at room temperature for 5 h. The reaction mixture was diluted with ether (10 mL), and washed with saturated aqueous NH_4Cl (20 mL), $NaHCO_3$ (20 mL) and NaCl (20 mL) in sequence, and the separated organic phase was dried with anhydrous Na_2SO_4. The solvent was evaporated in *vacuo* after filtration, and the residue was purified by column chromatography on silica gel (Hexanes:EtOAc:Et_3N= 20:1:0.2) to provide diazo compounds **3** as orange oil or solid depend on the substrate (> 90% yield).

Characterization of Diazoacetates 3.

3-Phenylprop-2-yn-1-yl 2-diazo-2-[2-(phenylethynyl)phenyl]acetate (3a)

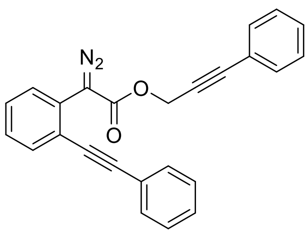

Orange oil, ^1H NMR (400 MHz, CDCl3) (δ, ppm) δ 7.70 (d, *J* = 7.5 Hz, 1H), 7.64 – 7.58 (comp, 3H), 7.52 – 7.47 (m, 2H), 7.45 – 7.40 (m, 1H), 7.39 – 7.29 (comp, 7H), 5.13 (s, 2H); ^{13}C NMR (100 MHz, $CDCl_3$) (δ, ppm) δ 165.1, 133.1, 132.0, 131.7, 130.0, 128.89, 128.85, 128.8, 128.5, 128.4, 127.7, 126.4, 122.83, 122.2, 121.3, 96.8, 86.8, 86.6, 83.1, 53.4. HRMS (TOF MS CI^+) calculated for $C_{25}H_{17}N_2O_2$ $[M + H]^+$, 377.1290; found, 377.1298.

3-(4-Chlorophenyl)prop-2-ynyl 2-diazo-2-[2-(phenylethynyl)phenyl]acetate (3b)

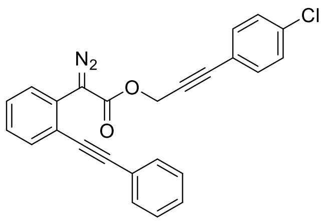

Orange oil, ^{1}H NMR (400 MHz, $CDCl_3$) (δ, ppm) δ 7.69 – 7.63 (m, 1H), 7.62 – 7.53 (comp, 3H), 7.49 – 7.31 (comp, 7H), 7.31 – 7.23 (comp, 3H), 5.08 (s, 2H); ^{13}C NMR (100 MHz, $CDCl_3$) (δ, ppm) δ 164.1, 134.1, 134.0, 133.1, 131.7, 130.0, 128.8, 128.5, 127.7, 126.4, 122.8, 121.3, 118.3, 115.8, 115.6, 96.8, 86.6, 85.7, 82.9, 53.3. HRMS (TOF MS CI^+) calculated for $C_{25}H_{16}ClN_2O_2$ $[M + H]^+$, 411.0900; found, 411.0906.

3-(4-Bromophenyl)prop-2-ynyl 2-diazo-2-[2-(phenylethynyl)phenyl]acetate (3c)

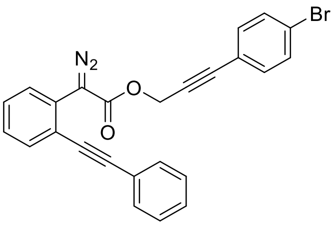

Orange oil, ^{1}H NMR (400 MHz, $CDCl_3$) (δ, ppm) δ 7.64 (d, *J* = 7.8 Hz, 1H), 7.59 – 7.52 (comp, 3H), 7.42 – 7.34 (comp, 3H), 7.34 – 7.23 (comp, 6H), 5.05 (s, 2H); ^{13}C NMR (100 MHz, $CDCl_3$) (δ, ppm) δ 165.0, 133.4, 133.0, 131.7, 131.6, 129.9, 128.8, 128.5, 127.7, 126.3, 123.2, 122.7, 121.3, 121.1, 96.8, 86.6, 85.6, 84.3, 53.2. HRMS (TOF MS CI^+) calculated for $C_{25}H_{16}BrN_2O_2$ $[M + H]^+$, 455.0395; found, 455.0391.

3-[4-(Trifluoromethyl)phenyl]prop-2-ynyl2-diazo-2-[2-(phenylethynyl)phenyl]acetate (3d)

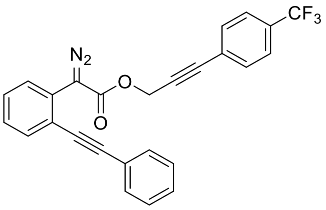

Orange solid, ^{1}H NMR (400 MHz, $CDCl_3$) (δ, ppm) δ 7.65 (d, *J* = 8.0 Hz, 1H), 7.60 – 7.50 (comp, 7H), 7.43 – 7.22 (comp, 5H), 5.09 (s, 2H); ^{13}C NMR (100 MHz, $CDCl_3$) δ 165.1, 133.1, 132.3, 131.7, 130.6 (m), 130.0, 128.9, 128.5,128.0(m) 127.8, 126.3, 126.1 (m), 125.3 (m), 122.8,122.6, 121.4, 96.8, 86.6, 85.6, 85.3, 53.1. HRMS (TOF MS CI^+) calculated for $C_{26}H_{16}F_3N_2O_2$ $[M + H]^+$, 455.1164; found, 455.1159.

3-(4-Methoxyphenyl)prop-2-ynyl 2-diazo-2-[2-(phenylethynyl)phenyl]acetate (3e)

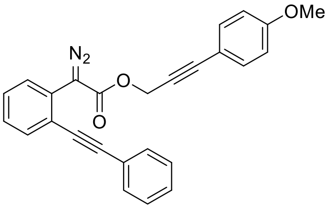

Orange oil, ^{1}H NMR (400 MHz, $CDCl_3$) (δ, ppm) δ 7.66 (d, *J* = 8.0 Hz, 1H), 7.63 – 7.53 (comp, 3H), 7.43 – 7.32 (comp, 6H), 7.31 – 7.23 (m, 1H), 6.89 – 6.76 (m, 2H), 5.08 (s, 2H), 3.78 (s, 3H); ^{13}C NMR (100 MHz, $CDCl_3$) (δ, ppm) δ 160.1, 133.6, 133.1, 131.8, 130.0, 128.8, 128.8, 128.5, 127.7, 126.5, 122.9, 121.3, 114.33, 114.0, 96.8, 86.8, 86.6, 81.8, 55.4, 53.6. HRMS (TOF MS CI^+) calculated for $C_{26}H_{19}N_2O_3$ $[M + H]^+$, 407.1396; found, 407.1390.

3-*p*-Tolylprop-2-ynyl 2-diazo-2-[2-(phenylethynyl)phenyl]acetate (3f)

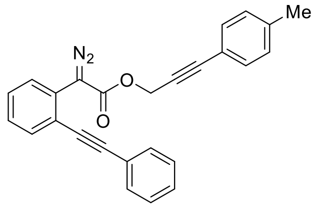

Orange oil, ^{1}H NMR (400 MHz, $CDCl_3$) (δ, ppm) δ 7.73 – 7.67 (m, 1H) δ 7.64 – 7.57 (comp, 3H), 7.45 – 7.35 (comp, 7H), 7.34 – 7.28 (m, 1H), 7.13 (d, *J* = 7.9 Hz, 2H), 5.12 (s, 2H), 2.36 (s, 3H); ^{13}C NMR (100 MHz, $CDCl_3$) (δ, ppm) δ 165.2, 139.1, 133.1, 132.0, 131.7, 130.0, 129.2, 128.8, 128.8, 128.5, 127.7, 126.5, 122.9, 121.3, 119.1, 96.8, 87.0, 86.6, 82.4, 53.5, 21.6. HRMS (TOF MS CI^+) calculated for $C_{26}H_{19}N_2O_2$ $[M + H]^+$, 391.1447; found, 391.1441.

3-*m*-Tolylprop-2-ynyl 2-diazo-2-[2-(phenylethynyl)phenyl]acetate (3g)

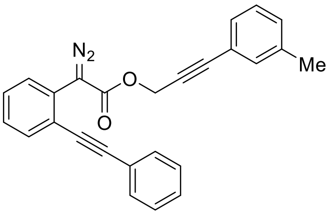

Orange solid, ^{1}H NMR (400 MHz, $CDCl_3$) (δ, ppm) δ 7.66 (d, *J* = 7.9 Hz, 1H), 7.61 – 7.52 (comp, 3H), 7.40 – 7.34 (m, 1H), 7.34 – 7.29 (comp, 3H), 7.29 – 7.22 (comp, 3H), 7.16 (t, *J* = 7.7 Hz, 1H), 7.10 (d, *J* = 7.6 Hz, 1H), 5.08 (s, 2H), 2.27 (s, 3H); ^{13}C NMR (100 MHz, $CDCl_3$) (δ, ppm) δ 165.0, 138.0, 133.0, 132.5, 131.6, 129.9, 129.7, 129.0, 128.8, 128.7, 128.4, 128.2, 127.6, 126.3, 122.7, 121.9, 121.2, 96.8, 86.9, 86.6, 82.7, 53.3, 21.1. HRMS (TOF MS CI^+) calculated for $C_{26}H_{19}N_2O_2$ $[M + H]^+$, 391.1447; found, 391.1443.

3-*o*-Tolylprop-2-ynyl 2-diazo-2-[2-(phenylethynyl)phenyl]acetate (3h)

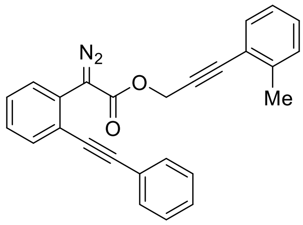

Orange oil, ^{1}H NMR (400 MHz, $CDCl_3$) (δ, ppm) δ 7.70 (d, *J* = 7.9 Hz, 1H), 7.65 – 7.54 (comp, 3H), 7.49 – 7.44 (m, 1H), 7.44 – 7.39 (m, 1H), 7.39 – 7.34 (m, 3H), 7.34 – 7.27 (m, 2H), 6.95 – 6.83 (m, 2H), 5.18 (s, 2H), 3.86 (s, 3H); ^{13}C NMR (100 MHz, $CDCl_3$) (δ, ppm) δ 165.02, 140.65, 132.98, 132.23, 131.65, 129.96, 129.48, 128.81, 128.77, 128.76, 128.44, 127.65, 126.38, 125.55, 122.76, 121.93, 121.26, 96.75, 86.89, 86.57, 85.73, 53.49, 20.63. HRMS (TOF MS CI^+) calculated for $C_{26}H_{19}N_2O_2$ $[M + H]^+$, 391.1447; found, 391.1451.

3-(Naphthalen-1-yl)prop-2-ynyl 2-diazo-2-[2-(phenylethynyl)phenyl]acetate (3i)

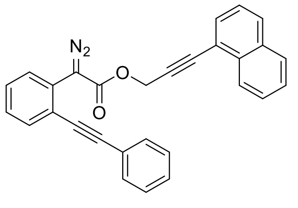

Orange oil, ^{1}H NMR (400 MHz, $CDCl_3$) (δ, ppm) δ 8.33 (d, *J* = 8.4 Hz, 1H), 7.86 – 7.77 (m, 2H), 7.69 (d, *J* = 7.3 Hz, 2H), 7.63 – 7.53 (comp, 4H), 7.52 – 7.46 (m, 1H), 7.43 – 7.36 (m, 2H), 7.34 – 7.26 (comp, 3H), 5.24 (s, 2H); ^{13}C NMR (100 MHz, $CDCl_3$) δ (ppm) δ 165.2, 133.5,

133.2, 133.1, 131.7, 131.1, 130.1, 129.4, 128.9, 128.8, 128.5, 128.4, 127.8, 127.1, 126.6, 126.4, 126.1, 125.2, 122.8, 121.4, 119.8, 96.8, 88.0, 86.6, 85.0, 53.6. HRMS (TOF MS CI$^+$) calculated for $C_{29}H_{19}N_2O_2$ [M + H]$^+$, 427.1447; found, 427.1451.

Pent-2-yn-1-yl 2-diazo-2-[2-(phenylethynyl)phenyl]acetate (3j)

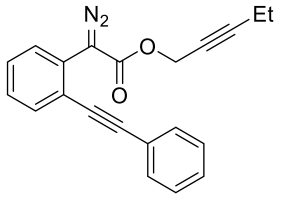

Orange oil, ^{1}H NMR (400 MHz, $CDCl_3$) (δ, ppm) δ 7.69 – 7.64 (m, 1H), 7.62 – 7.55 (comp, 3H), 7.40 – 7.34 (comp, 4H), 7.32 – 7.26 (m, 1H), 4.87 (t, *J* = 2.2 Hz, 2H), 2.32 – 2.19 (m, 2H), 1.16 (t, *J* = 4.8 Hz, 3H); ^{13}C NMR (100 MHz, $CDCl_3$) (δ, ppm) δ 165.1, 133.4, 133.0, 131.7, 129.9, 128.7, 128.4, 127.5, 126.4, 122.8, 121.2, 96.7, 89.2, 89.2, 86.6, 73.4, 53.4, 12.5. HRMS (TOF MS CI$^+$) calculated for $C_{21}H_{17}N_2O_2$ [M + H]$^+$, 329.1290; found, 329.1296.

3-(4-Chlorophenyl)prop-2-yn-1-yl 2-diazo-2-[2-((4-fluorophenyl)ethynyl)phenyl)acetate (3k)

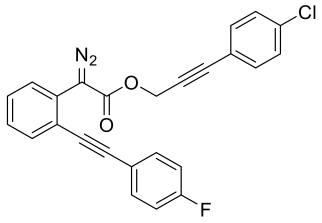

Orange oil, ^{1}H NMR (400 MHz, $CDCl_3$) (δ, ppm) δ 7.72 – 7.65 (m, 1H), 7.62 – 7.56 (comp, 3H), 7.49 – 7.42 (m, 2H), 7.40 – 7.35 (comp, 3H), 7.33 – 7.28 (m, 1H), 7.05 – 6.97 (m, 2H), 5.10 (s, 2H); ^{13}C NMR (100 MHz, $CDCl_3$) (δ, ppm) δ 164.1, 161.1, 134.1, 134.0, 133.1, 131.7, 130.0, 128.8 (d, *J* = 1.2 Hz), 128.5, 127.7, 126.4, 122.8, 121.3, 118.3 (d, *J* = 3.5 Hz), 115.7 (d, *J* = 22.1 Hz), 96.8, 86.6, 85.7, 82.9 , 53.3. HRMS (TOF MS CI$^+$) calculated for $C_{25}H_{15}ClFN_2O_2$ [M + H]$^+$, 429.0806; found, 428.0801.

3-(4-Chlorophenyl)prop-2-yn-1-yl 2-{2-[(4-chlorophenyl)ethynyl]phenyl}-2-diazoacetate (3l)

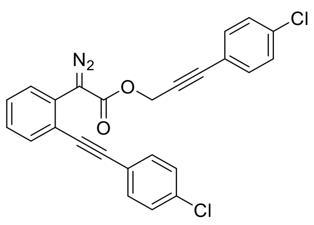

Yellow solid, ^{1}H NMR (400 MHz, $CDCl_3$) (δ, ppm) δ 7.67 (d, *J* = 7.9 Hz, 1H), 7.60 – 7.55 (m, 1H), 7.53 – 7.47 (m, 2H), 7.46 – 7.36 (comp, 3H), 7.35 – 7.27 (comp, 5H), 5.09 (s, 2H); ^{13}C NMR (100 MHz, $CDCl_3$) (δ, ppm) δ 165.0, 135.0, 134.9, 133.3, 133.1, 132.9, 130.0, 129.1, 128.9, 128.8, 127.8, 126.4, 121.3, 121.1, 120.7, 95.6, 87.5, 85.7, 84.1, 77.5, 77.2, 76.8, 53.3. HRMS (TOF MS CI$^+$) calculated for $C_{25}H_{15}Cl_2N_2O_2$ [M + H]$^+$, 445.0511; found, 444.0508.

Methyl -4-{[2-(2-((3-(4-chlorophenyl)prop-2-yn-1-yl)oxy)-1-diazo-2-oxoethyl)phenyl]ethynyl} benzoate (3m)

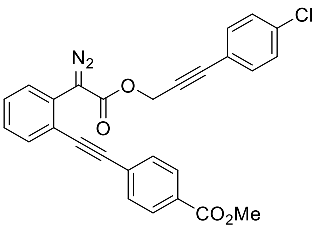

Yellow solid, ^{1}H NMR (400 MHz, $CDCl_3$) (δ, ppm) δ 8.04 – 7.97 (m, 2H), 7.70 – 7.56 (comp, 4H), 7.47 – 7.40 (m, 1H), 7.40 – 7.35 (m, 2H), 7.35 – 7.26 (comp, 3H), 5.09 (s, 2H), 3.93 (s, 3H); ^{13}C NMR (100 MHz, $CDCl_3$) (δ, ppm) δ 171.7, 166.7, 135.1, 133.3, 131.6, 130.10, 130.04, 129.7, 129.6, 129.4, 128.8, 127.9, 127.5, 126.7, 120.9, 120.7, 95.8, 89.4, 85.7, 84.1, 77.5, 77.2, 76.8, 53.4, 52.4. HRMS (TOF MS CI$^+$) calculated for $C_{27}H_{17}ClN_2O_4$ [M + H]$^+$, 469.0955; found, 469.0964.

3-(4-Chlorophenyl)prop-2-yn-1-yl 2-diazo-2-{2-[(4-methoxyphenyl)ethynyl]phenyl}acetate (3n)

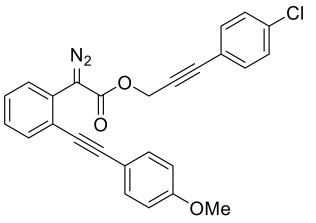

Orange oil, ^{1}H NMR (400 MHz, $CDCl_3$) (δ, ppm) δ 7.65 (d, *J* = 7.9 Hz, 1H), 7.60 – 7.55 (m, 1H), 7.54 – 7.48 (m, 2H), 7.42 – 7.36 (comp, 3H), 7.33 – 7.26 (comp, 3H), 6.93 – 6.84 (m, 2H), 5.09 (s, 2H), 3.82 (s, 3H); ^{13}C NMR (100 MHz, $CDCl_3$) (δ, ppm) δ 165.2, 160.1, 135.0, 133.3, 133.2, 132.9, 123.0, 128.8, 128.5, 127.8, 126.1, 121.8, 120.7, 114.9, 114.2, 97.0, 85.6, 85.5, 84.2, 55.4, 53.2. HRMS (TOF MS CI$^+$) calculated for $C_{26}H_{18}ClN_2O_3$ [M + H]$^+$, 411.1006; found, 411.1610.

3-(4-chlorophenyl)prop-2-yn-1-yl 2-diazo-2-[2-(*p*-tolylethynyl)phenyl]acetate (3o)

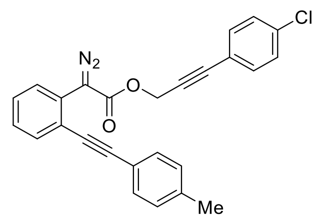

Orange oil, ^{1}H NMR (400 MHz, $CDCl_3$) (δ, ppm) δ 7.66 (d, *J* = 8.0 Hz, 1H), 7.58 (d, *J* = 7.7 Hz, 1H), 7.48 (d, *J* = 7.9 Hz, 2H), 7.44 – 7.36 (comp, 3H), 7.34 – 7.27 (comp, 3H), 7.17 (d, *J* = 8.0 Hz, 2H), 5.09 (s, 2H), 2.38 (s, 3H); ^{13}C NMR (100 MHz, $CDCl_3$) (δ, ppm) δ 165.2, 139.1, 1345.0, 133.3, 133.02, 131.7, 130.0, 129.3, 128.8, 128.7, 127.8, 126.3, 121.6, 120.8, 119.8, 97.1, 86.0, 85.6, 84.2, 53.2, 21.7. HRMS (TOF MS CI$^+$) calculated for $C_{26}H_{18}ClN_2O_2$ [M + H]$^+$, 425.1057; found, 425.1063.

3-(4-Chlorophenyl)prop-2-yn-1-yl 2-diazo-2-[2-(*m*-tolylethynyl)phenyl]acetate (3p)

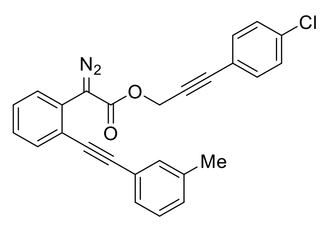

Orange oil, ^{1}H NMR (400 MHz, $CDCl_3$) (δ, ppm) δ 7.69 (d, *J* = 8.0 Hz, 1H), 7.64 – 7.59 (m, 1H), 7.47 – 7.39 (comp, 5H), 7.36 – 7.27 (comp, 4H), 7.20 (d, *J* = 7.4 Hz, 1H), 5.12 (s, 2H), 2.39 (s, 3H); ^{13}C NMR (100 MHz, $CDCl_3$) (δ, ppm) δ 165.2, 138.3, 135.0, 133.3,

133.1, 132.3, 130.0, 129.8, 128.9, 128.81, 128.78, 128.5, 127.8, 126.3, 122.7, 121.5, 120.6, 97.1, 86.3, 85.6, 84.2, 53.3, 21.4. HRMS (TOF MS CI$^+$) calculated for $C_{26}H_{18}ClN_2O_2$ [M + H]$^+$, 425.1057; found, 425.1059.

3-(4-Chlorophenyl)prop-2-yn-1-yl 2-diazo-2-[2-(*o*-tolylethynyl)phenyl]acetate (3q)

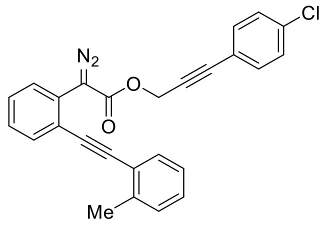

Orange oil, ^{1}H NMR (400 MHz, CDCl$_3$) (δ, ppm) δ 7.68 – 7.60 (m, 2H), 7.49 – 7.42 (comp, 3H), 7.37 – 7.28 (comp, 6H), 7.27 – 7.22 (m, 1H), 5.15 (s, 2H), 2.57 (s, 3H); ^{13}C NMR (100 MHz, CDCl$_3$) (δ, ppm) δ 165.1, 140.2, 135.0, 133.3, 133.2, 133.1, 132.2, 130.2, 129.7, 128.9, 128.80, 128.76, 127.9, 126.2, 125.8, 122.6, 120.7, 95.57, 9.40, 85.6, 84.2, 53.2, 20.8. HRMS (TOF MS CI$^+$) calculated for $C_{26}H_{18}ClN_2O_2$ [M + H]$^+$, 425.1057; found, 425.1053.

3-Phenylprop-2-yn-1-yl 2-{2-[(4-bromophenyl)ethynyl]phenyl}-2-diazoacetate (3r)

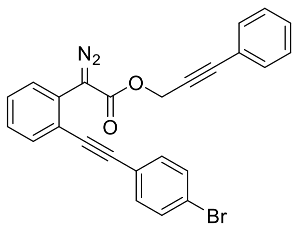

Orange oil, ^{1}H NMR (400 MHz, CDCl$_3$) (δ, ppm) δ 7.66 – 7.62 (m, 1H), 7.59 – 7.55 (m, 1H), 7.53 – 7.46 (comp, 4H), 7.43 – 7.38 (m, 2H), 7.37 – 7.28 (comp, 5H), 7.25 – 7.19 (m, 1H), 5.10 (s, 2H); ^{13}C NMR (100 MHz, CDCl$_3$) (δ, ppm) δ 164.4, 133.0, 132.6, 131.5, 131.3, 129.8, 129.5, 128.6, 128.4, 128.4, 127.9, 127.4, 127.3, 125.0, 121.7, 95.1, 87.2, 86.3, 82.5, 53.0. HRMS (TOF MS CI$^+$) calculated for $C_{25}H_{16}BrN_2O_2$ [M + H]$^+$, 455.0395; found, 455.0389.

3-(4-Chlorophenyl)prop-2-yn-1-yl 2-diazo-2-[2-(3,3-dimethylbut-1-yn-1-yl)phenyl]acetate(3s)

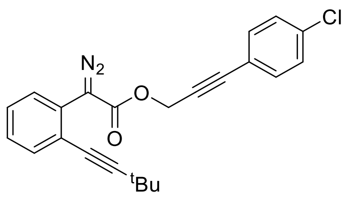

Orange oil, ^{1}H NMR (400 MHz, CDCl$_3$) (δ, ppm) δ 7.57 (d, *J* = 7.9 Hz, 1H), 7.47 – 7.37 (comp, 3H), 7.36 – 7.27 (comp, 3H), 7.26 – 7.19 (m, 1H), 5.07 (s, 2H), 1.34 (s, 9H); ^{13}C NMR (100 MHz, CDCl$_3$) (δ, ppm) δ 164.7, 134.4, 132.7, 132.7, 132.5, 129.4, 128.3, 128.2, 127.5, 127.1, 125.4, 121.7, 120.2, 105.7, 84.9, 83.7, 76.1, 52.6, 30.5. HRMS (TOF MS CI$^+$) calculated for $C_{23}H_{20}ClN_2O_2$ [M + H]$^+$, 391.1213; found, 391.1218.

3-(4-Chlorophenyl)prop-2-yn-1-yl 2-diazo-2-{2-[(triisopropylsilyl)ethynyl]phenyl}acetate (3t)

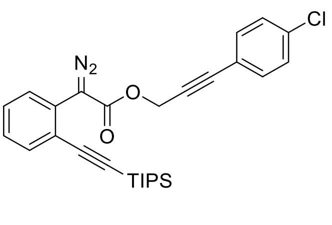

Yellow oil. ^{1}H NMR (400 MHz, CDCl$_3$) (δ, ppm) δ 7.60 (t, *J* = 7.6 Hz, 1H), 7.55 (d, *J* = 7.7 Hz, 1H), 7.37 (comp, 3H), 7.32 – 7.24 (comp, 3H), 5.08 (s, 2H), 1.99 – 1.68 (m, 3H), 1.14 (d, *J* = 4.2 Hz,

18H); ^{13}C NMR (101 MHz, $CDCl_3$) (δ, ppm) δ 165.0, 135.0, 133.7, 133.2, 130.4, 128.9, 128.8, 127.8, 126.8, 122.2, 120.8, 103.8, 98.7, 85.5, 84.2, 53.1, 18.7, 11.4. HRMS (TOF MS CI^+) calculated for $C_{28}H_{32}ClN_2O_2Si$ $[M + H]^+$, 491.1922; found, 491.1924.

3-Phenylprop-2-yn-1-yl 2-diazo-2-(2-ethynylphenyl)acetate (3u)

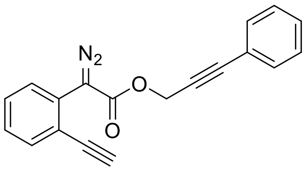

Orange oil, ^{1}H NMR (400 MHz, $CDCl_3$) (δ, ppm) δ 7.72 (d, J = 8.1 Hz, 1H), 7.61 (d, J = 7.0 Hz, 1H), 7.58 – 7.52 (comp, 3H), 7.42 – 7.36 (comp, 4H), 5.17 (s, 2H), 3.58 (s, 1H); ^{13}C NMR (100 MHz, $CDCl_3$) (δ, ppm) δ 164.4, 133.1, 131.5, 129.7, 128.8, 128.4, 127.9, 127.2, 126.78, 121.7, 86.3, 84.2, 82.6, 80.3, 52.6, 29.3. HRMS (TOF MS CI^+) calculated for $C_{19}H_{13}N_2O_2$ $[M + H]^+$, 301.0997; found, 301.0998.

General Procedure for the Asymmetric C-H Functionalization

Scheme 2: To a 10-mL oven-dried vial containing a magnetic stirring bar, diazo compound **1** (0.2 mmol), and 4Å MS (100 mg) in TBME (1.0 mL), Rh_2(S-TFPTTL)$_4$ (3.0 mg, 1.0 mol%) was slowly added as a solution in TBME (1.0 mL) *via* a syringe pump over 40 min under argon atmosphere at room temperature. After addition, the reaction mixture was stirred for additional 1-5 hours as indicated, and then purified by column chromatography on silica gel without any additional treatment (Hexanes: DCM = 20:1 to 10:1) to give the desired fluorene products **2**.

Scheme 3: To a 10-mL oven-dried vial containing a magnetic stirring bar, diazo compound **3** (0.2 mmol), and 4Å MS (100 mg) in TBME (1.0 mL), Rh_2(S-TFPTTL)$_4$ (3.0 mg, 1.0 mol%) was added as a solution in TBME (1.0 mL) *via* a syringe pump over 40 min under argon atmosphere at 40 ^{o}C. After addition, the reaction mixture was stirred for additional 20 min, and then purified by column chromatography on silica gel without any additional treatment (Hexanes: DCM = 2:1 to 1:1) to give the desired polycyclic products **4**.

Characterization of Asymmetric C-H Functionalization Products 2 and 4.

(*S*)-2-Fluoro-9-phenyl-9*H*-fluorene (2a)

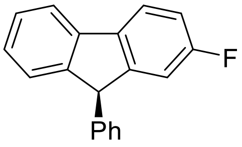

White solid**,** 46.8 mg, 90% yield, 99% *ee*, mp: 130.9-132.1 ^{o}C. ^{1}H NMR (400 MHz, $CDCl_3$) (δ, ppm) δ 7.86 – 7.73 (m, 2H), 7.50 – 7.39 (m, 1H), 7.38 – 7.27 (comp, 5H), 7.19 – 7.08 (comp, 3H), 7.07 – 7.01 (m, 1H), 5.06 (s, 1H); ^{13}C NMR (100 MHz, $CDCl_3$) (δ, ppm) δ 162.8 (d, J = 245.5 Hz), 150.2 (d, J = 8.2 Hz), 147.9, 141.05 (s), 140.3, 137.1, 129.0, 128.4, 127.6, 127.4 (d, J = 39.2 Hz),

127.1, 125.5, 121.0 (d, J = 8.8 Hz), 119.7, 114.7 (d, J = 23.1 Hz), 112.9 (d, J = 23.0 Hz), 54.6 (d, J = 2.3 Hz). HRMS (TOF MS CI$^+$) calculated for $C_{19}H_{14}F$ [M + H]$^+$, 261.1080; found, 261.1076. HPLC conditions for determination of enantiomeric excess: Chiral IB-3, λ = 272 nm, Condition: hexane/2-propanol/ethanol = 99.2:0.4:0.4, flow rate= 1.0 mL/min, t_{major} = 4.3 min, t_{minor} = 4.9 min.

(*S*)-2-Chloro-9-phenyl-9*H*-fluorene (2b)

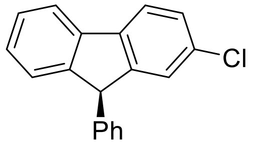

White solid, 50.2 mg, 91% yield, 98% *ee*, mp: 142.9-143.5 ^oC. ^1H NMR (400 MHz, $CDCl_3$) (δ, ppm) δ 7.79 (d, J = 7.6 Hz, 1H), 7.73 (d, J = 8.1 Hz, 1H), 7.40 (comp, 2H), 7.35 – 7.27 (comp, 6H), 7.13 – 7.07 (comp, 2H), 5.04 (s, 1H); ^{13}C NMR (100 MHz, $CDCl_3$) (δ, ppm) δ 149.7, 147.8, 140.8, 140.1, 139.7, 133.1, 129.0, 128.4, 127.8, 127.7, 127.6, 127.2, 125.8, 125.5, 120.9, 120.0, 54.5. HRMS (TOF MS CI$^+$) calculated for $C_{19}H_{14}Cl$ [M + H]$^+$, 277.0784; found, 277.0783. HPLC conditions for determination of enantiomeric excess: Chiral IB-3, λ = 272 nm, Condition: hexane/2-propanol/ethanol = 99.2:0.4:0.4, flow rate= 1.0 mL/min, t_{major} = 4.6 min, t_{minor} = 4.9 min.

(*S*)-2-Bromo-9-phenyl-9*H*-fluorene (2c)

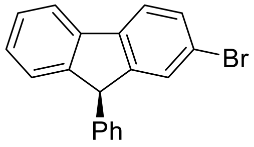

White solid, 58.9 mg, 92% yield, 95% *ee*, mp: 167.5-168.4 ^oC. ^1H NMR (400 MHz, $CDCl_3$) (δ, ppm) δ 7.78 (d, J = 7.6 Hz, 1H), 7.66 (d, J = 8.1 Hz, 1H), 7.51 (comp, 1H), 7.44 (s, 1H), 7.42 – 7.37 (m, 1H), 7.29 (comp, 5H), 7.12 – 7.05 (m, 2H), 5.03 (s, 1H); ^{13}C NMR (100 MHz, $CDCl_3$) (δ, ppm) δ 150.0, 147.7, 140.8, 140.1, 132.1, 130.6, 129.0, 128.7, 128.4, 127.9, 127.7, 127.3, 125.5, 121.3, 121.2, 120.1, 54.48. HRMS (TOF MS CI$^+$) calculated for $C_{19}H_{14}Br$ [M + H]$^+$, 321.0279; found, 321.0286. HPLC conditions for determination of enantiomeric excess: Chiral IB-3, λ = 272 nm, Condition: hexane/2-propanol/ethanol = 99.2:0.4:0.4, flow rate= 1.0 mL/min, t_{major} = 6.0 min, t_{minor} = 7.0 min.

(*S*)-9-Phenyl-2-(trifluoromethyl)-9*H*-fluorene (2d)

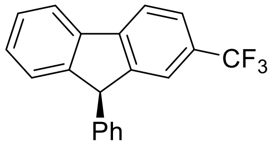

White solid, 58.9 mg, 95% yield, 96% *ee*, mp: 111.2-112.8 ^oC. ^1H NMR (400 MHz, $CDCl_3$) (δ, ppm) δ 7.88 (t, J = 8.5 Hz, 2H), 7.68 (d, J = 8.0 Hz, 1H), 7.59 (s, 1H), 7.45 (m, 1H), 7.40 – 7.28 (comp, 5H), 7.11 (d, J = 7.1 Hz, 2H), 5.10 (s, 1H); ^{13}C NMR (100 MHz, $CDCl_3$) (δ, ppm) δ 148.7, 148.3, 144.6, 140.5, 139.7,129.3 (q, J = 128.0 Hz) 129.1, 128.7, 128.4, 127.8, 127.4, 125.7, 124.86 (q, J = 3.7 Hz), 124.6 (q, J = 248.4 Hz) 122.40 (q, J = 3.8 Hz), 120.7, 120.1, 54.6. HRMS (TOF MS CI$^+$) calculated for $C_{20}H_{14}F_3$ [M + H]$^+$, 311.1048; found, 311.1041. HPLC conditions for determination of enantiomeric excess: Chiral IB-3, λ = 272 nm, Condition:

hexane/2-propanol/ethanol = 99.2:0.4:0.4, flow rate= 1.0 mL/min, t_{major} = 4.5 min, t_{minor} = 5.0 min.

(*S*)-2-Methyl-9-phenyl-9*H*-fluorene (2e)

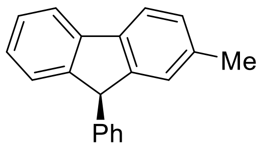

White solid, 49.2 mg, 96% yield, 93% *ee*, mp: 103.2-104.9 °C. ^{1}H NMR (400 MHz, $CDCl_3$) (δ, ppm) δ 7.84 (d, *J* = 7.6 Hz, 1H), 7.77 (d, *J* = 7.7 Hz, 1H), 7.44 (t, *J* = 7.4 Hz, 1H), 7.40 – 7.24 (comp, 6H), 7.19 (comp, 3H), 5.08 (s, 1H), 2.43 (s, 3H); ^{13}C NMR (100 MHz, $CDCl_3$) (δ, ppm) δ 148.3, 147.9, 141.9, 141.2, 138.5, 137.3, 128.8, 128.5, 128.3, 127.4, 126.9, 126.9, 126.1, 125.4, 119.7, 119.7, 54.4, 21.7. HRMS (TOF MS CI$^+$) calculated for $C_{20}H_{17}$ [M + H]$^+$, 257.1330; found, 257.1324. HPLC conditions for determination of enantiomeric excess: Chiral IB-3, λ = 272 nm, Condition: hexane/2-propanol/ethanol = 99.2:0.4:0.4, flow rate= 1.0 mL/min, t_{major} = 4.4 min, t_{minor} = 4.1 min.

(*S*)-2-Methoxy-9-phenyl-9*H*-fluorene (2f)

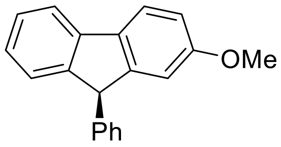

White solid, 50.6 mg, 93% yield, 90% *ee*, mp: 169.2-170.6 °C. ^{1}H NMR (400 MHz, $CDCl_3$) (δ, ppm) δ 8.00 (d, *J* = 7.8 Hz, 1H), 7.44 (t, *J* = 7.5 Hz, 1H), 7.38 (d, *J* = 7.5 Hz, 1H), 7.34 – 7.25 (comp, 4H), 7.19 – 7.23 (comp, 3H), 7.13 (comp, 2H), 5.06 (s, 1H), 2.81 (s, 3H);^{13}C NMR (100 MHz, $CDCl_3$) (δ, ppm) δ 148.5, 148.5, 142.1, 142.1, 139.1, 133.1, 129.7, 128.8, 128.5, 127.3, 127.1, 126.9, 126.7, 125.4, 123.2, 123.0, 54.5, 21.1. HRMS (TOF MS CI$^+$) calculated for $C_{20}H_{17}O$ [M + H]$^+$, 273.1279; found, 273.1277. HPLC conditions for determination of enantiomeric excess: Chiral IB-3, λ = 272 nm, Condition: hexane/2-propanol/ethanol = 99.6:0.2:0.2, flow rate= 1.0 mL/min, t_{major} = 6.0 min, t_{minor} = 6.6 min.

(*S*)-7-Phenyl-7*H*-benzo[*c*]fluorine (2g)

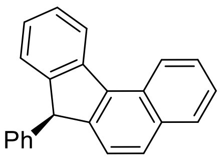

White solid, 55.5 mg, 95% yield, 92% *ee*, mp: 133.2-134.6 °C. ^{1}H NMR (400 MHz, $CDCl_3$) (δ, ppm) δ 8.88 (d, *J* = 8.5 Hz, 1H), 8.47 (d, *J* = 7.8 Hz, 1H), 8.00 (d, *J* = 8.1 Hz, 1H), 7.82 (d, *J* = 8.3 Hz, 1H), 7.78 – 7.66 (m, 1H), 7.56 (comp, 3H), 7.46 (d, *J* = 7.4 Hz, 1H), 7.40 – 7.28 (comp, 4H), 7.16 (comp, 2H), 5.15 (s, 1H); ^{13}C NMR (100 MHz, $CDCl_3$) (δ, ppm) δ 149.3, 146.9, 142.2, 141.2, 135.6, 133.8, 129.6, 129.4, 128.9, 128.6, 128.5, 127.6, 127.1, 126.8, 126.5, 125.42, 125.34, 124.1, 123.4, 123.0, 55.0. HRMS (TOF MS CI$^+$) calculated for $C_{23}H_{17}$ [M + H]$^+$, 293.1330; found, 293.1328. HPLC conditions for determination of enantiomeric excess: Chiral IB-3, λ = 272 nm, Condition: hexane/2-propanol/ethanol = 99.1:0.45:0.45, flow rate= 1.0 mL/min, t_{major} = 5.8 min, t_{minor} = 6.3

min.

(*S*)-4-Fluoro-9-phenyl-9*H*-fluorene (2h)

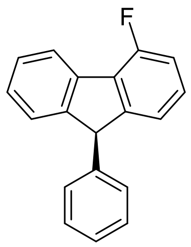

White solid, 49.4 mg, 95% yield, 91% *ee*, mp: 112.3-113.5 °C. ^{1}H NMR (400 MHz, CDCl$_3$) (δ, ppm) δ 8.05 (d, *J* = 7.4 Hz, 1H), 7.45 (t, *J* = 7.2 Hz, 1H), 7.40 – 7.20 (comp, 6H), 7.11 (comp, 4H), 5.12 (s, 1H); ^{13}C NMR (100 MHz, CDCl$_3$) (δ, ppm) δ 158.4 (d, *J* = 250.1 Hz), 150.8 (d, *J* = 5.1 Hz), 147.4, 141.2, 138.2, 128.9, 128.56 (d, *J* = 7.0 Hz), 128.4, 128.3 127.8, 127.6, 127.2, 125.2, 123.6 (d, *J* = 5.6 Hz), 121.1 (d, *J* = 3.3 Hz), 114.4 (d, *J* = 19.7 Hz), 55.1. HRMS (TOF MS CI$^+$) calculated for $C_{19}H_{14}F$ [M + H]$^+$, 261.1080; found, 261.1082. HPLC conditions for determination of enantiomeric excess: Chiral IB-3, λ = 272 nm, Condition: hexane/2-propanol/ethanol = 99.6:0.2:0.2, flow rate= 1.0 mL/min, t_{major} = 6.9 min, t_{minor} = 8.0 min.

(*S*)-3-Fluoro-9-phenyl-*9H*-fluorene (2i)

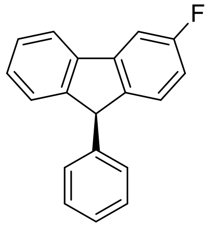

White solid, 49.4 mg, 95% yield, 94% *ee*, mp: 123.4-124.7 °C. ^{1}H NMR (400 MHz, CDCl$_3$) (δ, ppm) δ 7.69 (d, *J* = 7.6 Hz, 1H), 7.40 (m, 1H), 7.33 (t, *J* = 7.1 Hz, 1H), 7.26 – 7.12 (comp, 6H), 7.02 (d, *J* = 6.8 Hz, 2H), 6.88 (m, 1H), 4.95 (s, 1H); ^{13}C NMR (100 MHz, CDCl$_3$) (δ, ppm) δ 163.0 (d, *J* = 243.9 Hz), 148.9, 143.4 (d, *J* = 2.5 Hz), 143.1 (d, *J* = 8.9 Hz), 141.4, 140.3 (d, *J* = 3.2 Hz), 128.9, 128.4, 128.0, 127.6, 127.1, 126.5 (d, *J* = 9.1 Hz), 125.6, 120.3, 114.3 (d, *J* = 23.0 Hz), 107.0 (d, *J* = 23.0 Hz), 54.0. HRMS (TOF MS CI$^+$) calculated for $C_{19}H_{14}F$ [M + H]$^+$, 261.1080; found, 261.1081. HPLC conditions for determination of enantiomeric excess: Chiral IB-3, λ = 272 nm, Condition: hexane/2-propanol = 100:0, flow rate= 1.0 mL/min, t_{major} = 5.9 min, t_{minor} = 7.1 min.

(*S*)-9-(4-Chlorophenyl)-2-fluoro-9*H*-fluorene (2j)

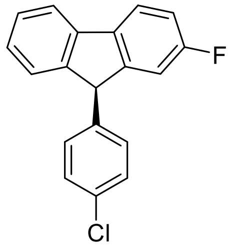

White solid, 54.7 mg, 93% yield, 95% *ee*, mp: 147.8-149.3 °C. ^{1}H NMR (400 MHz, CDCl$_3$) (δ, ppm) δ 7.82 – 7.71 (m, 2H), 7.47 – 7.39 (m, 1H), 7.28 (m, 4H), 7.12 (m, 1H), 7.07 – 6.98 (m, 3H), 5.02 (s, 1H); ^{13}C NMR (100 MHz, CDCl$_3$) (δ, ppm) δ 162.8 (d, *J* = 245.8 Hz), 149.7 (d, *J* = 8.1 Hz), 147.4, 140.3, 139.6, 137.1, 133.0, 129.7, 129.1, 127.8, 127.2, 125.4, 121.0 (d, *J* = 8.8 Hz), 119.8, 114.9 (d, *J* = 23.1 Hz), 112.7 (d, *J* = 23.1 Hz), 53.9 (d, *J* = 2.3 Hz). HRMS (TOF MS CI$^+$) calculated for $C_{19}H_{13}ClF$ [M + H]$^+$, 295.0690; found, 295.0696. HPLC conditions for determination of enantiomeric excess: Chiral IB-3, λ = 272 nm, Condition:

hexane/2-propanol/ethanol = 99:0.5:0.5, flow rate= 1.0 mL/min, t_{major} = 5.8 min, t_{minor} = 6.7 min.

(*S*)-2-Fluoro-9-(*p*-tolyl)-9*H*-fluorene (2k)

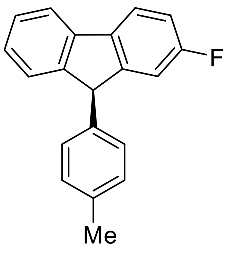

White solid, 49.9 mg, 91% yield, 92% *ee*, mp: 137.6-138.9 °C. ^{1}H NMR (400 MHz, $CDCl_3$) (δ, ppm) δ 7.82 – 7.71 (m, 2H), 7.41 (t, *J* = 7.3 Hz, 1H), 7.34 (d, *J* = 7.4 Hz, 1H), 7.31 – 7.27 (m, 1H), 7.12 (comp, 3H), 7.07 – 6.96 (comp, 3H), 5.03 (s, 1H), 2.37 (s, 3H); ^{13}C NMR (100 MHz, $CDCl_3$) (δ, ppm) δ 162.8 (d, *J* = 245.2 Hz), 150.4 (d, *J* = 8.2 Hz), 148.0, 140.3, 138.0, 137.1, 136.8, 129.7, 128.3, 127.5, 127.1, 125.4, 120.9 (d, *J* = 8.8 Hz), 119.7, 114.6 (d, *J* = 23.1 Hz), 112.7 (d, *J* = 23.0 Hz), 54.2 (d, *J* = 2.2 Hz), 21.2. HRMS (TOF MS CI^{+}) calculated for $C_{20}H_{16}F$ [M + H]$^{+}$, 275.1236; found, 275.1230; HPLC conditions for determination of enantiomeric excess: Chiral IB-3, λ = 272 nm, Condition: hexane/2-propanol/ethanol = 99.2:0.4:0.4, flow rate= 1.0 mL/min, t_{major} = 6.0 min, t_{minor} = 7.4 min.

(*R*)-8-Phenyl-1*H*-benzo[1,2]fluoreno[3,4-*c*]furan-3(8*H*)-one (4a)

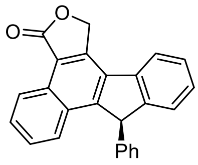

White solid, 62.7 mg, 90% yield, 92% *ee*, $[\alpha]_D^{20}$ = 78.2° (c = 0.5, MeOH), mp: 267.2-268.5 °C. ^{1}H NMR (400 MHz, $CDCl_3$) (δ, ppm) δ 9.14 (d, *J* = 8.4 Hz, 1H), 7.76 (d, *J* = 8.4 Hz, 1H), 7.71 – 7.64 (m, 1H), 7.56 (d, *J* = 7.5 Hz, 1H), 7.53 – 7.46 (m, 2H), 7.44 – 7.36 (m, 2H), 7.34 – 7.25 (comp, 3H), 7.11 – 6.94 (m, 2H), 5.69 (dd, *J* = 65.4, 15.9 Hz, 2H), 5.24 (s, 1H); ^{13}C NMR (100 MHz, $CDCl_3$) (δ, ppm) δ171.4, 150.2, 149.2, 142.7, 140.5, 138.6, 133.3, 130.3, 129.3, 129.2, 128.3, 128.05,128.03, 127.9, 127.7, 127.4, 125.5, 125.3, 124.4, 121.2, 120.4, 68.6, 55.1. HRMS (TOF MS CI^{+}) calculated for $C_{25}H_{17}O_2$ [M + H]$^{+}$, 349.1223; found, 349.1233. HPLC conditions for determination of enantiomeric excess: Chiral IB-3, λ = 272 nm, hexane/2-propanol /ethanol= 97.5:1.25:1.25, flow rate= 1.0 mL/min, t_{major} = 35.3 min, t_{minor} = 38.2 min.

(*R*)-10-Chloro-8-phenyl-1*H*-benzo[1,2]fluoreno[3,4-*c*]furan-3(8*H*)-one (4b)

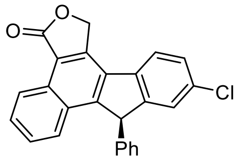

White solid, 67.2 mg, 88% yield, 93% *ee*, $[\alpha]_D^{20}$ = 136.4° (c = 0.5, MeOH), mp: > 280 °C. ^{1}H NMR (400 MHz, $CDCl_3$) (δ, ppm) δ 9.10 (d, *J* = 8.0 Hz, 1H), 7.70-7.62 (m, 2H), 7.54-7.51 (m, 2H), 7.49-7.44 (m, 2H), 7.35-7.34 (m, 2H), 7.24-7.22 (m, 2H), 6.99-6.96 (m, 2H), 5.77-5.71 (m, 1H), 5.64-5.57 (m, 1H), 5.25-5.23 (m, 1H); ^{13}C NMR (100 MHz, $CDCl_3$) δ 171.2, 149.6, 148.7, 142.6, 139.0, 138.6, 133.33, 133.25, 130.1, 129.6, 129.4, 129.2, 128.4, 128.2, 127.8, 125.4, 125.10, 125.08, 124.5, 121.3, 120.6, 68.6, 54.3. HRMS (TOF MS CI^{+}) calculated for $C_{25}H_{16}ClO_2$ [M +

H]$^+$, 383.0839; found, 383.0828. HPLC conditions for determination of enantiomeric excess: Chiral IB-3, λ = 272 nm, hexane/2-propanol /ethanol= 97.5:1.25:1.25, flow rate= 1.0 mL/min, t_{major} = 41.5 min, t_{minor} = 44.0 min.

(*R*)-10-Bromo-8-phenyl-1*H*-benzo[1,2]fluoreno[3,4-c]furan-3(8*H*)-one (4c)

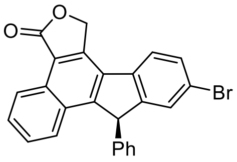

Yellow solid, 73.5 mg, 86% yield, 92% *ee*, $[\alpha]_D^{20}$ = 86.8° (c = 0.5, MeOH), mp: > 280 °C. ^{1}H NMR (400 MHz, $CDCl_3$) (δ, ppm) δ 9.11 (d, *J* = 8.3 Hz, 1H), 7.77 (d, *J* = 8.4 Hz, 1H), 7.64 (t, *J* = 7.7 Hz, 1H), 7.52 – 7.40 (comp, 3H), 7.37 (s, 1H), 7.34 – 7.26 (comp, 3H), 7.13 – 7.03 (m, 2H), 5.76 (dd, *J* =26.8 Hz, 15.9 Hz, 2H), 5.46 (s, 1H); ^{13}C NMR (100 MHz, $CDCl_3$) (δ, ppm) δ 171.2, 150.8, 150.2, 142.4, 139.7, 137.2, 134.1, 130.3, 129.6, 129.4, 128.5, 128.4, 128.1, 127.9, 127.8, 126.1, 125.3, 124.5, 122.05, 120.8, 68.5, 55.2. HRMS (TOF MS CI$^+$) calculated for $C_{25}H_{16}BrO_2$ [M + H]$^+$, 427.0334; found, 427.0338. HPLC conditions for determination of enantiomeric excess: Chiral IA-3, λ = 272 nm, hexane/2-propanol = 92:8, flow rate= 1.0 mL/min, t_{major} = 18.4 min, t_{minor} = 20.1 min.

(*R*)-8-Phenyl-10-(trifluoromethyl)-1*H*-benzo[1,2]fluoreno[3,4-*c*]furan-3(8*H*)-one (4d)

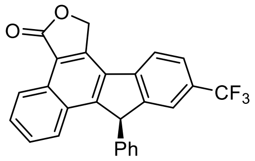

Yellow solid, 64.1mg, 77% yield, 99% *ee*, $[\alpha]_D^{20}$ = 122.6° (c = 0.5, MeOH), mp: 226.1-227.8 °C. ^{1}H NMR (400 MHz, $CDCl_3$) (δ, ppm) δ 9.11 (d, 1H, *J* = 8.3 Hz), 7.80-7.78 (m, 1H), 7.73-7.71 (m, 1H), 7.68-7.61 (comp, 3H), 7.50-7.46 (m, 1H), 7.31-7.28 (comp, 3H), 7.08-7.07 (m, 2H), 5.80 (d, 1H, *J* = 15.9 Hz), 5.71 (d, 1H, *J* = 15.9 Hz), 5.45 (s, 1H); ^{13}C NMR (100 MHz, $CDCl_3$) (δ, ppm) δ171.0, 151.4, 149.5, 142.7, 142.1, 139.3, 131.9, 130.2, 130.1, 129.8, 129.7, 129.6, 129.0, 128.0, 127.9, 125.6, 125.49, 125.45, 125.41, 125.37, 124.5, 122.9, 122.4 (q, *J* = 15.0 Hz), 121.3, 120.9, 68.4, 55.2, 29.8. HRMS (TOF MS CI$^+$) calculated for $C_{26}H_{16}F_3O_2$ [M + H]$^+$, 417.1102; found, 417.1109. HPLC conditions for determination of enantiomeric excess: Chiral IA-3, λ = 272 nm, hexane/2-propanol = 95:5, flow rate= 1.0 mL/min, t_{major} =22.9 min.

(*R*)-10-Methoxy-8-phenyl-1*H*-benzo[1,2]fluoreno[3,4-*c*]furan-3(8*H*)-one (4e)

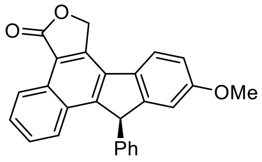

Yellow solid, 47.6 mg, 63% yield, 82% *ee*. mp: 248.2-249.4 °C. ^{1}H NMR (400 MHz, $CDCl_3$) (δ, ppm) δ 9.04 (d, *J* = 8.8 Hz, 1H), 7.59-7.56 (comp, 2H), 7.42-7.38 (m, 1H), 7.33-7.31 (m, 1H), 7.25-7.18 (comp, 3H), 6.96-6.92 (comp, 3H), 6.84-6.83 (m, 1H), 5.60-5.56 (m, 1H), 5.37-5.32 (m, 1H), 4.87 (s, 1H), 3.82 (s, 3H); ^{13}C NMR (100 MHz, $CDCl_3$) (δ, ppm) δ 171.3,

160.2, 151.2, 149.0, 142.1, 140.5, 133.1, 131.3, 130.1, 129.3, 128.5, 127.9, 127.7, 127.5, 127.4, 124.9, 124.3, 121.8, 120.1, 113.59, 113.57, 111.37, 111.34, 68.4, 55.7, 54.9. HRMS (TOF MS CI$^+$) calculated for $C_{26}H_{19}O_3$ [M + H]$^+$, 379.1334; found, 379.1335. HPLC conditions for determination of enantiomeric excess: Chiral IA-3, λ = 272 nm, hexane/2-propanol = 96:04, flow rate= 1.0 mL/min, t_{major} =22.0 min, t_{minor} = 26.6 min.

(*R*)-10-methyl-8-phenyl-1*H*-benzo[1,2]fluoreno[3,4-*c*]furan-3(8*H*)-one (**4f**)

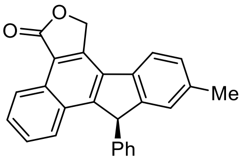

White solid, 63.7 mg, 88% yield, 96% ee, $[\alpha]_D^{20}$ = 110.0^o (c = 0.5, MeOH), mp: >280 ^{o}C. ^{1}H NMR (400 MHz, $CDCl_3$) (δ, ppm) δ 9.19 (d, J = 8.4 Hz, 1H), 7.80 (d, *J* = 8.4 Hz, 1H), 7.72 (t, *J* = 8.0 Hz, 1H), 7.56-7.48 (m, 2H), 7.37-7.34 (comp, 4H), 7.13-7.11 (m, 2H), 5.81 (d, *J* = 15.6 Hz, 1H), 5.66 (d, *J* = 15.6 Hz, 1H), 5.26 (s, 1H), 2.50 (s, 3H); ^{13}C NMR (100 MHz, $CDCl_3$) (δ, ppm) δ 171.4, 149.8, 149.4, 142.5, 140.7, 138.3, 133.4, 131.8, 130.3, 129.3, 129.0, 128.8, 128.1, 128.0, 127.6, 127.4, 126.2, 125.2, 124.4, 120.9, 120.3, 68.6, 54.9, 21.8. HRMS (TOF MS CI$^+$) calculated for $C_{26}H_{19}O_2$ [M + H]$^+$, 363.1385; found, 363.1380. HPLC conditions for determination of enantiomeric excess: Chiral IA-3, λ = 272 nm, hexane/2-propanol = 93:7, flow rate= 1.0 mL/min, t_{major} =29.7 min, t_{minor} = 32.7 min.

(*R*)-9-Methyl-8-phenyl-1*H*-benzo[1,2]fluoreno[3,4-*c*]furan-3(8*H*)-one (**4g**)

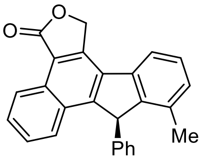

White solid, 38.4 mg, 53% yield, 97% *ee*, $[\alpha]_D^{20}$ = 148.8^o (c = 0.5, MeOH), mp: 248.8-250.1 ^{o}C. ^{1}H NMR (400 MHz, $CDCl_3$) (δ, ppm) δ 9.07 (d, *J* = 8.4 Hz, 1H), 7.69 (d, *J* = 8.4 Hz, 1H), 7.63-7.59 (m, 1H), 7.45-7.41 (m, 1H), 7.28-7.20 (comp, 5H), 7.16-7.14 (m, 1H), 6.99-6.97 (m, 2H), 5.70 (d, *J* = 15.6 Hz, 1H), 5.56-5.50 (m, 1H), 5.12-5.10 (m, 1H), 2.49 (s, 3H); ^{13}C NMR (100 MHz, $CDCl_3$) (δ, ppm) δ 171.2, 150.4, 146.3, 142.5, 140.6, 138.8, 137.7, 133.1, 130.1, 129.1, 128.9, 128.8, 128.0, 127.8, 127.4, 127.2, 125.1, 125.0, 124.2, 121.6, 120.1, 68.5, 54.5, 21.6. HRMS (TOF MS CI$^+$) calculated for $C_{26}H_{19}O_2$ [M + H]$^+$, 363.1385; found, 363.1392. HPLC conditions for determination of enantiomeric excess: Chiral IB-3, λ = 272 nm, hexane/2-propanol /ethanol= 98.5:0.75:0.75**,** flow rate= 1.0 mL/min, t_{major} =34.7 min, t_{minor} = 37.9 min.

(*R*)-12-Methyl-8-phenyl-1*H*-benzo[1,2]fluoreno[3,4-*c*]furan-3(8*H*)-one (**4g’**)

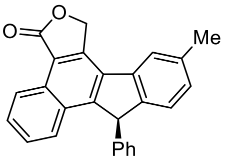

White solid, 23.9 mg, 33% yield, 60% *ee*, mp: 276.6-277.8 ^oC. ^1H NMR (400 MHz, $CDCl_3$) (δ, ppm) δ 9.19 (d, *J* = 8.4 Hz, 1H), 7.79 (d, *J* = 8.4 Hz, 1H), 7.64-7.59 (m, 1H), 7.45-7.41 (m, 1H), 7.24-7.16 (comp, 6H), 7.03-7.01 (m, 2H), 5.91 (d, *J* = 15.6 Hz, 1H), 5.81 (d, *J* = 15.6 Hz, 1H), 5.29 (s, 1H), 2.84 (s, 3H); ^{13}C NMR (100 MHz, $CDCl_3$) (δ, ppm) δ 171.6, 151.6, 150.2, 142.2, 141.6, 138.0, 134.3, 131.0, 130.9, 130.3, 129.3, 129.1, 128.5, 128.2, 127.9, 127.8, 127.3, 125.5, 124.2, 123.5, 121.3, 71.6, 54.6, 23.1. HRMS (TOF MS CI^+) calculated for $C_{26}H_{19}O_2$ $[M + H]^+$, 363.1385; found, 363.1391. HPLC conditions for determination of enantiomeric excess: Chiral IB-3, λ = 272 nm, hexane/2-propanol /ethanol= 97.5:1.25:1.25**,** flow rate= 1.0 mL/min, t_{major} =41.5 min, t_{minor} = 43.8 min.

(*R*)-11-Methyl-8-phenyl-1*H*-benzo[1,2]fluoreno[3,4-*c*]furan-3(8*H*)-one (**4h**)

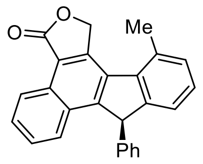

White solid, 27.5 mg, 38% yield, < 5% *ee*, mp: 274.3-275.6 ^oC. ^1H NMR (400 MHz, $CDCl_3$) (δ, ppm) δ 9.05 (d, *J* = 8.4 Hz, 1H), 7.84 (d, *J* = 8.4 Hz, 1H), 7.62-7.58 (m, 1H), 7.48-7.44 (m, 1H), 7.41-7.38 (m, 1H), 7.34-7.32 (m, 1H), 7.20-7.21 (comp, 4H), 6.99-6.97 (m, 2H), 5.67 (d, *J* = 16.0 Hz, 1H), 5.47-5.42 (m, 1H), 5.07-5.04 (m, 1H), 2.11 (s, 3H); ^{13}C NMR (100 MHz, $CDCl_3$) (δ, ppm) δ 171.3, 151.0, 147.4, 142.7, 139.1, 139.0, 135.6, 132.7, 130.0, 129.8, 129.1, 128.95, 128.93, 128.4, 128.1, 127.6, 127.1, 124.8, 124.4, 120.2, 118.8, 68.6, 54.8, 19.2. HRMS (TOF MS CI^+) calculated for $C_{26}H_{19}O_2$ $[M + H]^+$, 363.1385; found, 363.1391. HPLC conditions for determination of enantiomeric excess: Chiral IB-3, λ = 272 nm, hexane/2-propanol /ethanol = 97.5:1.25:1.25, flow rate = 1.0 mL/min, t_{major} = 41.8 min, t_{minor} = 45.1 min.

(*R*)-8-Phenyl-1*H*-dibenzo[1,2:5,6]fluoreno[3,4-*c*]furan-3(8*H*)-one (**4i**)

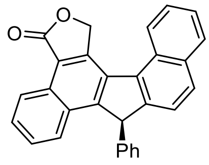

Yellow solid, 70.9 mg, 89% yield, 92% *ee*, mp: > 280 ^oC. ^1H NMR (400 MHz, $CDCl_3$) (δ, ppm) δ 9.23 (d, *J* = 8.4 Hz, 1H), 8.42-8.28 (m, 1H), 7.96 (d, *J* = 7.8 Hz, 1H), 7.90-7.76 (m, 2H), 7.73-7.53 (comp, 3H), 7.46 (t, *J* = 8.8 Hz, 2H), 7.23 (comp, 3H), 7.04 (d, *J* = 7.0 Hz, 2H), 6.12 (d, *J* = 15.0 Hz, 1H), 5.92 (d, *J* = 14.8 Hz, 1H), 5.30 (s, 1H); ^{13}C NMR (100 MHz, $CDCl_3$) (δ, ppm) δ 171.6, 152.5, 149.0, 142.2, 140.5, 135.1, 134.7, 134.0, 130.3, 130.0, 129.7, 129.4, 128.9, 128.5, 128.3, 128.1, 127.9, 127.5, 126.9, 125.7, 125.3, 124.2, 123.86, 123.2, 121.4, 71.8, 55.4. HRMS (TOF MS CI^+) calculated for $C_{26}H_{19}O_2$ $[M + H]^+$, 399.1385; found, 399.1378. HPLC conditions for determination of enantiomeric excess: Chiral IB-3, λ = 272 nm, hexane/2-propanol = 95:5, flow rate= 1.0

mL/min, t_{major} =28.7 min, t_{minor} = 37.4 min.

(*E*)-3-(2-(Phenylethynyl)phenyl)-4-(prop-1-en-1-yl)furan-2(5*H*)-one (4j)

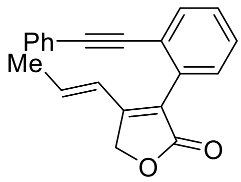

Yellow solid, 25.7 mg, 43% yield, mp: 95.3-95.6 °C. ^{1}H NMR (400 MHz, $CDCl_3$) (δ, ppm) δ 7.73-7.66 (m, 1H), 7.50-7.39 (m, 5H), 7.39-7.34 (m, 3H), 6.50 (d, *J* = 16.1 Hz, 1H), 6.28-6.16 (m, 1H), 5.13 (d, *J* = 6.8 Hz, 2H), 1.90 (dd, *J* = 6.8, 1.6 Hz, 3H); ^{13}C NMR (100 MHz, $CDCl_3$) (δ, ppm) δ 173.4, 156.3, 135.6, 132.9, 132.4, 131.5, 130.4, 128.7, 128.5, 128.49, 128.45, 124.40, 123.8, 123.5, 123.3, 93.5, 88.3, 69.4, 19.2. HRMS (TOF MS CI$^+$) calculated for $C_{21}H_{16}O_2$ [M + H]$^+$, 300.1229; found, 300.1230.

(*R*)-10-Chloro-8-(4-fluorophenyl)-1*H*-benzo[1,2]fluoreno[3,4-*c*]furan-3(8*H*)-one (4k)

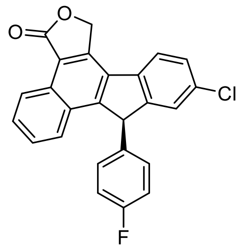

Yellow solid, 72.0 mg, 90% yield, 92% *ee*, mp: > 280 °C. ^{1}H NMR (400 MHz, $CDCl_3$) (δ, ppm) δ 9.10 (d, *J* = 8.4 Hz, 1H), 7.72 (d, *J* = 8.3 Hz, 1H), 7.65 (t, *J* = 7.6 Hz, 1H), 7.52 – 7.40 (m, 3H), 7.33 (s, 1H), 7.09 – 6.93 (m, 4H), 5.72 (q, *J* = 15.9 Hz, 2H), 5.39 (s, 1H); ^{13}C NMR (100 MHz, $CDCl_3$) (δ, ppm) δ 171.1, 163.5, 161.0, 150.6, 149.8, 142.4, 137.1, 135.4(d, *J* = 3.4 Hz), 134.1, 132.3, 129.8(d, *J* = 8.1 Hz), 129.5, 129.4, 128.5(d, *J* = 11.3 Hz), 128.0, 126.0, 125.1, 124.6, 122.1, 120.9, 116.5 (d, *J* = 21.6 Hz), 68.5, 54.2. HRMS (TOF MS CI$^+$) calculated for $C_{25}H_{15}ClFO_2$ [M + H]$^+$, 401.0745; found, 401.0748. HPLC conditions for determination of enantiomeric excess: Chiral IA-3, λ = 272 nm, hexane/2-propanol = 96:4, flow rate= 1.0 mL/min, t_{major} =23.9 min, t_{minor} = 28.5 min.

(*R*)-10-Chloro-8-(4-chlorophenyl)-1*H*-benzo[1,2]fluoreno[3,4-*c*]furan-3(8*H*)-one (4l)

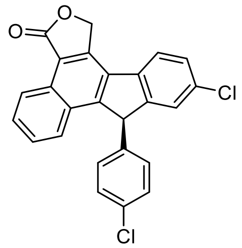

Yellow solid, 75.7 mg, 91% yield, 99% *ee*, $[\alpha]_D^{20}$ = 118.4° (c = 0.5, MeOH), mp: > 280 °C. ^{1}H NMR (600 MHz, $CDCl_3$) (δ, ppm) δ 9.11 (d, *J* = 8.4 Hz, 1H), 7.72 (d, *J* = 8.5 Hz, 1H), 7.65 (t, *J* = 7.6 Hz, 1H), 7.50 – 7.46 (m, 2H), 7.44 – 7.40 (m, 1H), 7.33 (s, 1H), 7.27 (s, 2H), 7.01 (d, *J* = 8.3 Hz, 2H), 5.75 (dd, *J* = 31.8 Hz,*J* = 15.7 Hz, 2H), 5.43 (s, 1H); ^{13}C NMR (150 MHz, $CDCl_3$) (δ, ppm) δ 171.1, 150.3, 149.5, 142.4, 138.3, 137.2, 134.2, 133.7, 132.5, 130.2, 129.8, 129.4, 129.0, 128.7, 128.57, 128.1, 126.0, 125.1, 124.7, 122.2, 121.0, 68.5, 54.4. HRMS (TOF MS CI$^+$) calculated for $C_{25}H_{15}ClFO_2$ [M + H]$^+$, 417.0449; found, 417.0441. HPLC conditions for determination of enantiomeric excess: Chiral IA-3, λ = 272 nm, hexane/2-propanol = 96:4, flow rate= 1.0 mL/min, t_{major} =16.5 min.

(*R*)-Methyl-4-(10-chloro-3-oxo-3,8-dihydro-1*H*-benzo[1,2]fluoreno[3,4-*c*]furan-8-yl)benzoate (4m)

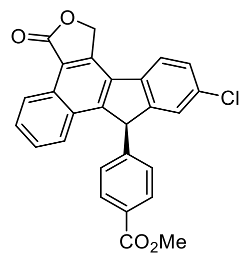

Yellow solid, 83.2 mg, 90% yield, 97 % *ee*, $[\alpha]_D^{20}$ = 134.8° (c = 0.5, MeOH), mp: > 280 °C. ^{1}H NMR (600 MHz, $CDCl_3$) (δ, ppm) δ 9.12 (d, *J* = 8.3 Hz, 1H), 7.97 (d, *J* = 8.3 Hz, 2H), 7.70 (d, *J* = 8.4 Hz, 1H), 7.65 (s, 1H), 7.50 (d, *J* = 8.1 Hz, 1H), 7.45 (d, *J* = 6.9 Hz, 2H), 7.34 (s, 1H), 7.17 (d, *J* = 8.0 Hz, 2H), 5.78 (q, *J* = 15.7 Hz, 2H), 5.53 (s, 1H), 3.89 (s, 3H); ^{13}C NMR (150 MHz, $CDCl_3$) (δ, ppm) δ 171.1, 166.7, 150.0, 149.4, 145.1, 142.4, 137.4, 134.2, 132.6, 130.9, 130.2, 130.1, 129.8, 129.4, 129.0, 128.7, 128.1, 126.0, 125.0, 124.7, 122.2, 121.1, 68.5, 54.9, 52.3. HRMS (TOF MS ESI$^+$) calculated for $C_{27}H_{17}ClNaO_4$ [M + Na]$^+$, 463.0713; found, 463.0719. HPLC conditions for determination of enantiomeric excess: Chiral IA-3, λ = 272 nm, hexane/2-propanol = 96:4, flow rate= 1.0 mL/min, t_{major} =36.6 min, t_{minor} = 33.4 min.

(*R*)-10-Chloro-8-(4-methoxyphenyl)-1*H*-benzo[1,2]fluoreno[3,4-*c*]furan-3(*8H*)-one (4n)

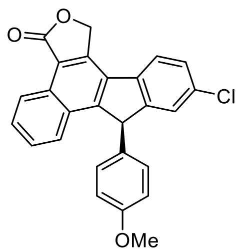

White solid, 70.9 mg, 86% yield, 90% *ee*, mp: 236.3-237.8 °C. ^{1}H NMR (400 MHz, $CDCl_3$) (δ, ppm) δ 9.03 (d, *J* = 8.4 Hz, 1H), 7.71 (d, *J* = 8.4 Hz, 1H), 7.62-7.58 (m, 1H), 7.47-7.43 (m, 1H), 7.41-7.36 (comp, 2H), 7.30 (s, 1H), 6.92 (d, *J* = 8.4 Hz, 2H), 6.79 (d, *J* = 8.4 Hz, 2H), 5.66 (d, *J* = 16.0 Hz, 1H), 5.52-5.48 (m, 1H), 5.14 (s, 1H), 3.76 (s, 3H); ^{13}C NMR (100 MHz, $CDCl_3$) (δ, ppm) δ 171.1, 159.1, 151.1, 150.2, 142.3, 137.0, 134.0, 132.0, 131.4, 130.1, 129.2, 129.0, 128.4, 128.2, 127.8, 125.8, 125.2, 124.3, 121.9, 120.5, 114.9, 68.4, 55.3, 54.2. HRMS (TOF MS CI$^+$) calculated for $C_{26}H_{18}ClO_3$ [M + H]$^+$, 413.0944; found, 413.0937. HPLC conditions for determination of enantiomeric excess: Chiral IB-3, λ = 272 nm, hexane/2-propanol /ethanol= 98.5:0.75:0.75, flow rate= 1.0 mL/min, t_{major} =41.9 min, t_{minor} = 44.7 min.

(*R*)-10-Chloro-8-(*p*-tolyl)-1*H*-benzo[1,2]fluoreno[3,4-*c*]furan-3(8*H*)-one (4o)

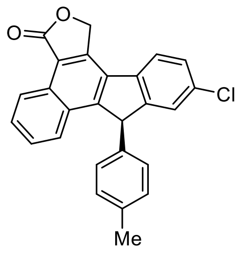

Yellow solid, 70.5 mg, 89% yield, 90% *ee*, mp: 219.3-220.5 °C. ^{1}H NMR (400 MHz, $CDCl_3$) (δ, ppm) δ 9.04 (d, *J* = 8.4 Hz, 1H), 7.71 (d, *J* = 8.4 Hz, 1H), 7.62-7.58 (m, 1H), 7.47-7.43 (m, 1H), 7.40-7.37 (comp, 2H), 7.32 (s, 1H), 7.07 (d, *J* = 8.0 Hz, 2H), 6.89 (d, *J* = 8.0 Hz, 2H), 5.68 (d, *J* = 16.0 Hz, 1H), 5.53 (d, *J* = 16.0 Hz, 1H), 5.19 (s, 1H), 2.30 (s, 3H); ^{13}C NMR (100 MHz, $CDCl_3$) (δ, ppm) δ 171.1, 151.0, 150.2, 142.3, 137.4, 137.1, 136.4, 134.0, 132.1, 130.2, 130.1, 129.2, 128.4, 128.2, 127.81, 127.79, 125.9, 125.2, 124.4, 121.9, 120.5, 68.4, 54.7, 21.2.

HRMS (TOF MS CI$^+$) calculated for $C_{26}H_{18}ClO_2$ [M + H]$^+$, 397.0995; found, 397.0993. HPLC conditions for determination of enantiomeric excess: Chiral IB-3, λ = 272 nm, hexane/2-propanol =98:1:1, flow rate= 1.0 mL/min, t_{major} =38.22 min, t_{minor} = 41.58 min.

(*R*)-10-Chloro-8-(*m*-tolyl)-1*H*-benzo[1,2]fluoreno[3,4-*c*]furan-3(8*H*)-one (**4p**)

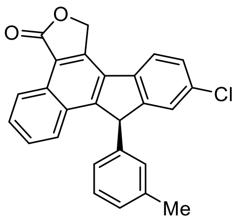

Yellow solid, 72.1 mg, 91% yield, 93% *ee*, mp: 210.5-211.7 ^{o}C. ^{1}H NMR (400 MHz, $CDCl_3$) (δ, ppm) δ 9.08 (d, *J* = 8.4 Hz, 1H), 7.75 (d, *J* = 8.4 Hz, 1H), 7.65-7.61 (m, 1H), 7.48-7.42 (comp, 3H), 7.34 (s, 1H), 7.18 (t, *J* = 7.2 Hz, 1H), 7.07 (d, *J* = 7.6 Hz, 1H), 6.88 (d, *J* = 7.6 Hz, 1H), 6.79 (s, 1H), 5.74 (d, *J* = 16.0 Hz, 1H), 5.62 (d, *J* = 16.0 Hz, 1H), 5.28 (s, 1H), 2.24 (s, 3H); ^{13}C NMR (100 MHz, $CDCl_3$) (δ, ppm) δ 171.2, 150.9, 150.2, 142.4, 139.5, 139.3, 137.2, 134.0, 132.3, 130.3, 129.33, 129.26, 128.6, 128.5, 128.3, 127.9, 126.0, 125.3, 125.2, 124.4, 122.0, 120.6, 68.5, 55.1, 21.6. HRMS (TOF MS CI$^+$) calculated for $C_{26}H_{18}ClO_2$ [M + H]$^+$, 397.0995; found, 397.0992. HPLC conditions for determination of enantiomeric excess: Chiral IA-3, λ = 272 nm, hexane/2-propanol = 93:7, flow rate= 1.0 mL/min, t_{major} =29.7 min, t_{minor} = 32.7 min.

(*R*)-10-Chloro-8-(*o*-tolyl)-1*H*-benzo[1,2]fluoreno[3,4-c]furan-3(8*H*)-one (**4q**)

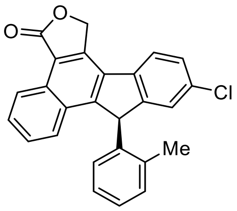

Yellow solid, 68.9 mg, 87% yield, 96% *ee*, $[\alpha]_D^{20}$ = 132.4^o (c = 0.5, MeOH), mp: 232.5-233.7 ^{o}C. ^{1}H NMR (400 MHz, $CDCl_3$) (δ, ppm) δ 9.07 (d, *J* = 8.4 Hz, 1H), 7.73-7.68 (m, 1H), 7.64-7.60 (m, 1H), 7.49-7.41 (comp, 3H), 7.36-7.31 (m, 2H), 7.04 (d, *J* = 7.6 Hz, 1H), 6.89-6.85 (m, 2H), 5.72-5.76 (m, 1H), 5.50-5.43 (m, 1H), 5.12-5.04 (m, 1H), 2.28 (s, 3H); ^{13}C NMR (100 MHz, $CDCl_3$) (δ, ppm) δ171.3, 150.3, 149.4, 142.7, 138.5, 137.3, 137.0, 133.1 (d, *J* = 3.2 Hz),130.2 (d, *J* = 2.5 Hz), 130.0, 129.1 (d, *J* = 1.9 Hz), 128.2, 128.0, 127.8, 127.6, 125.4, 125.33, 125.31, 124.3 (d, *J* = 1.4 Hz), 121.1, 120.2, 68.5, 54.6, 21.2. HRMS (TOF MS CI$^+$) calculated for $C_{26}H_{18}ClO_2$ [M + H]$^+$, 397.0995; found, 397.0999. HPLC conditions for determination of enantiomeric excess: Chiral IA-3, λ = 272 nm, hexane/2-propanol = 92:8, flow rate= 1.0 mL/min, t_{major} =13.4 min, t_{minor} = 12.6 min.

(*R*)-8-(4-Bromophenyl)-1*H*-benzo[1,2]fluoreno[3,4-*c*]furan-3(8*H*)-one (**4r**)

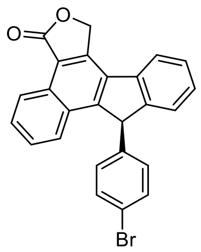

Yellow solid, 74.1 mg, 87% yield, 90% *ee*, mp: 253.5-254.8 °C. ^{1}H NMR (400 MHz, $CDCl_3$) (δ, ppm) δ 9.11 (d, *J* = 8.4 Hz, 1H), 7.73 (d, *J* = 8.4 Hz, 1H), 7.67-7.63 (m, 1H), 7.57-7.55 (m, 1H), 7.50-7.44 (m, 2H), 7.40-7.32 (comp, 3H), 6.95 (d, *J* = 8.0 Hz, 2H), 5.79 (d, *J* = 16.0 Hz, 1H), 5.72-5.68 (m, 1H), 5.35 (s, 1H); ^{13}C NMR (100 MHz, $CDCl_3$) (δ, ppm) δ 171.3, 149.5, 148.6, 142.6, 139.6, 138.7, 133.4, 132.5, 130.2, 129.7, 129.3, 128.4, 128.22, 128.19, 127.9, 125.4, 125.1, 124.6, 121.4, 121.3, 120.7, 68.6, 54.4. HRMS (TOF MS CI$^+$) calculated for $C_{25}H_{15}BrO_2$ [M + H]$^+$, 427.0334; found, 427.0327. HPLC conditions for determination of enantiomeric excess: Chiral IA-3, λ = 272 nm, hexane/2-propanol /ethanol= 98.5:0.75:0.75, flow rate= 1.0 mL/min, t_{major} =53.8 min, t_{minor} = 60.2 min.

(*R*)-8-(*tert*-Butyl)-10-chloro-1*H*-benzo[1,2]fluoreno[3,4-c]furan-3(8*H*)-one (**4s**)

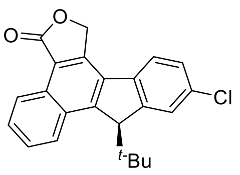

Yellow solid, 60.1 mg, 83% yield, 68% *ee*, mp: 183.4-184.6 °C. ^{1}H NMR (600 MHz, $CDCl_3$) (δ, ppm) δ 9.12 – 9.07 (m, 1H), 8.18 (d, *J* = 8.3 Hz, 1H), 7.69 – 7.65 (m, 1H), 7.64 – 7.60 (m, 2H), 7.42 – 7.38 (m, 1H), 7.35 (d, *J* = 8.0 Hz, 1H), 5.74 (d, *J* = 15.5 Hz, 1H), 5.60 (d, *J* = 15.5 Hz, 1H), 4.44 (s, 1H), 0.92 (s, 9H); ^{13}C NMR (150 MHz, $CDCl_3$) (δ, ppm) δ 171.2, 151.6, 149.5, 142.1, 139.0, 132.3, 131.3, 129.7, 129.2, 128.9, 128.1, 127.9, 126.8, 126.5, 124.1, 121.5, 120.1, 68.4, 58.8, 51.8, 29.3. HRMS (TOF MS CI$^+$) calculated for $C_{23}H_{20}ClO_2$ [M + H]$^+$, 363.1152; found, 363.1155. HPLC conditions for determination of enantiomeric excess: Chiral IA-3, λ = 272 nm, hexane/2-propanol = 92:8, flow rate= 1.0 mL/min, t_{major} = 18.9 min, t_{minor} = 18.2 min.

(*R*)-10-Chloro-8-(triisopropylsilyl)-1*H*-benzo[1,2]fluoreno[3,4-c]furan-3(8*H*)-one (4t)

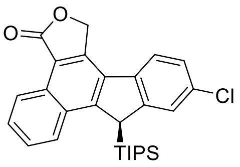

White solid**,** 75.5 mg, 78% yield, 84% *ee*, mp: 184.2-189.3 °C. ^{1}H NMR (400 MHz, $CDCl_3$) (δ, ppm) δ 9.08 (d, *J* = 8.3 Hz, 1H), 7.94 (d, *J* = 8.3 Hz, 1H), 7.71 (t, *J* = 7.2 Hz, 1H), 7.62 (t, *J* = 7.1 Hz, 1H), 7.54 (s, 1H), 7.40 (m, 1H), 7.34 (d, *J* = 8.1 Hz, 1H), 5.55 (d, *J* = 15.8 Hz, 1H), 5.32 (d, *J* = 15.8 Hz, 1H), 4.33 (s, 1H), 1.08 (m, 3H), 0.76 (d, *J* = 7.4 Hz, 9H), 0.65 (d, *J* = 7.5 Hz, 9H); ^{13}C NMR (100 MHz, $CDCl_3$) (δ, ppm) δ 171.4, 152.3, 148.4, 142.9, 137.6, 132.1, 129.7, 129.6, 128.2, 128.1, 126.3, 126.2, 125.8, 124.9, 124.3, 121.7, 118.1, 68.2, 41.0, 18.5, 18.4, 12.1. HRMS (TOF MS ESI$^+$) calculated for $C_{28}H_{31}ClNaO_2Si$ [M + Na]$^+$, 485.1680; found, 485.1678. HPLC conditions for determination of enantiomeric excess: Chiral IA-3, λ = 272 nm, hexane/2-propanol = 91:9, flow rate= 1.0 mL/min, t_{major} = 16.6 min, t_{minor} = 14.7 min.

1*H*-Benzo[1,2]fluoreno[3,4-*c*]furan-3(8*H*)-one (**4u**)

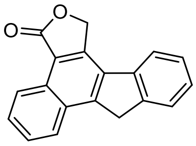

Yellow solid, 22.3 mg, 41% yield, mp: 163.4-164.8 °C. ^{1}H NMR (400 MHz, $CDCl_3$) (δ, ppm) δ 9.10-9.07 (m, 1H), 8.10-8.08 (m, 1H), 7.73-7.66 (comp, 3H), 7.54-7.40 (comp, 3H), 5.65 (s, 2H), 4.27 (s, 2H); ^{13}C NMR (100 MHz, $CDCl_3$) (δ, ppm) δ 171.5, 147.7, 143.2, 143.0, 140.4, 132.9, 130.6, 128.6, 128.4, 127.8, 127.6, 127.5, 125.5, 124.6, 124.5, 121.3, 119.6, 68.6, 36.9. HRMS (TOF MS CI$^+$) calculated for $C_{19}H_{12}O_2$ [M + H]$^+$, 273.0916; found, 273.0923.

3-Phenylprop-2-yn-1-yl 2-hydroxy-2-[2-(phenylethynyl)phenyl]acetate (**5a**)

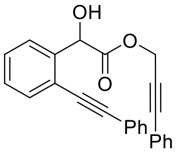

Colorless oil, ^{1}H NMR (400 MHz, $CDCl_3$) (δ, ppm) δ 7.62 – 7.53 (comp, 3H), 7.47 – 7.42 (m, 1H), 7.37 – 7.23 (comp, 10H), 5.70 (d, *J* = 5.7 Hz, 1H), 4.98 (dd, *J* = 76.8, 15.5 Hz, 2H), 3.65 (dd, *J* = 5.7, 2.2 Hz, 1H); ^{13}C NMR (100 MHz, $CDCl_3$) (δ, ppm) δ173.0, 139.5, 132.9, 132.0, 131.7, 128.9, 128.9, 128.7, 128.7, 128.5, 128.4, 127.7, 123.0, 122.6, 122.0, 94.6, 87.2, 86.7, 82.2, 72.2, 54.4. HRMS (TOF MS CI$^+$) calculated for $C_{25}H_{19}O_3$ [M + H]$^+$, 367.1334; found, 367.1339.

General Procedure of the Scale Up and Synthesis of 6c, 7c and 8c.

Gram scale reaction for the synthesis of 4c.

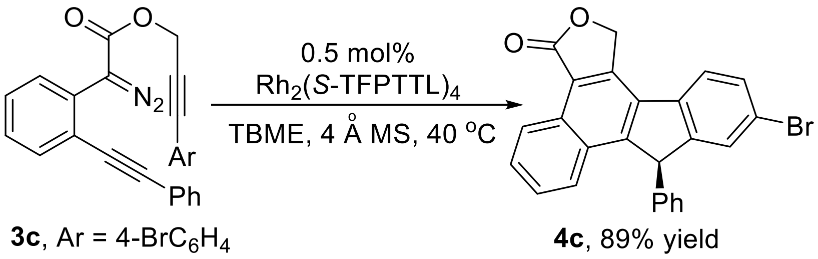

To a 50-mL oven-dried flask containing a magnetic stirring bar, diazo compound **3c** (1.82g, 4.0 mmol) and 4Å MS (1.3 g) in TBME (20 ml), Rh_2(S-TFPTTL)$_4$ (23.0 mg, 0.5 mol%) was added in TBME (5.0 ml) *via* a syringe pump over 2 h under argon at 40 °C, and the reaction mixture was stirred for additional 20 min. After evaporating the solvents, the residue was purified by column chromatography on silica gel without any additional treatment (Hexanes: DCM = 2:1 to 1:1) to give 1.52 g of **4c** (89% yield) with 94% *ee*.

Synthesis of 6c.[7]

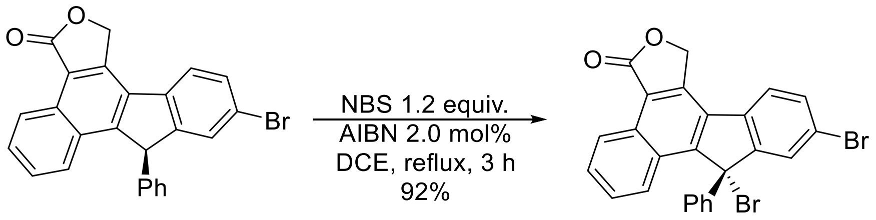

4c **6c**

To a 10 mL oven-dried glassware containing a magnetic stirring bar and **4c** (85.4 mg, 0.2 mmol) in DCE (2.0 mL), NBS (42 mg, 0.24 mmol) and AIBN (1.0 mg, 2.0 mmol%) was added under refluxing. After the reaction was completed (monitored by TLC), the reaction mixture was cooled to room temperature, and purified by column chromatography on silica gel (Hexanes:DCM = 2:1) to give **6c** as yellow solid (93.1 mg, 92% yield), mp: > 280 °C. ^{1}H NMR (600 MHz, $CDCl_3$) (δ, ppm) δ 9.18 (d, *J* = 8.4 Hz, 1H), 7.83 (d, *J* = 8.5 Hz, 1H), 7.69 (t, *J* = 7.6 Hz, 1H), 7.65 (s, 1H), 7.58 – 7.52 (m, 2H), 7.44 (d, *J* = 7.3 Hz, 2H), 7.36 (d, *J* = 8.0 Hz, 1H), 7.34 – 7.27 (m, 3H), 5.76 (dd, *J* = 20.4, 15.6 Hz, 2H); ^{13}C NMR (150 MHz, $CDCl_3$) (δ, ppm) δ 170.7, 153.6, 149.8, 142.0, 138.5, 134.3, 132.6, 130.3, 129.9, 129.4, 129.2, 129.0, 128.96, 128.9, 127.9, 127.2, 126.3, 124.6, 123.2, 122.9, 122.4, 68.4, 65.8. HRMS (TOF MS ESI) calculated for $C_{25}H_{14}Br_2NaO_2$ [M + Na]$^+$, 528.9238; found, 528.9243.

Synthesis of 7c.[8]

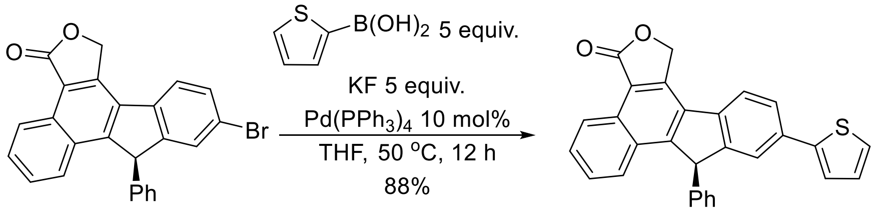

4c **7c**

To a 10-mL oven-dried vial containing a magnetic stirring bar, **4c** (85.4 mg, 0.2 mmol), KF (58.1 mg, 1.0 mmol), $Pd(PPh_3)_4$ (23.1 mg, 10 mol%) and thiophen-2-yl boronic acid (128.0 mg, 1.0 mmol), was added THF (5.0 mL) under argon atmosphere. Then the reaction mixture was stirred at 50 °C for 12 h. After the reaction was completed (monitored by TLC), the reaction mixture was cooled to room temperature and quenched with water (20 mL). The reaction mixture was extracted with EtOAc (20 mL). The organic layer was dried over anhydrous Na_2SO_4 and concentrated under reduced pressure after filtrations. The obtained residue was purified by flash column chromatography on silica gel (Hexanes: DCM = 2:1) to give 75.7 mg of coupling product **7c** as yellow solid (88% yield), mp: 233.3-234.8 °C. ^{1}H NMR (400 MHz, $CDCl_3$) (δ, ppm) δ 9.10 (d, *J* = 8.4 Hz, 1H), 7.78 (d, *J* = 8.4 Hz, 1H), 7.72 – 7.67 (m, 1H), 7.66 – 7.60 (m, 1H), 7.58 (s, 1H), 7.53

(d, J = 7.9 Hz, 1H), 7.49 – 7.42 (m, 1H), 7.34 – 7.25 (comp, 5H), 7.14 – 7.05 (comp, 3H), 5.74 (dd, J = 37.4, 15.9 Hz, 2H), 5.42 (s, 1H); ^{13}C NMR (100 MHz, $CDCl_3$) (δ, ppm) δ 171.3, 150.4, 150.0, 144.1, 142.6, 140.3, 137.9, 134.4, 132.9, 130.3, 129.4, 129.2, 128.3, 128.1, 127.8, 127.6, 125.9, 125.4, 125.3, 124.5, 123.7, 122.9, 121.6, 120.6, 68.6, 55.2. HRMS (TOF MS ESI) calculated for $C_{29}H_{18}NaO_2S$[M + Na]$^+$, 453.0925; found, 453.0929.

Synthesis of 8c.[9]

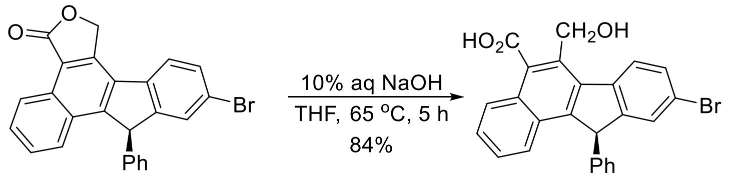

4c **8c**

To a 50-mL flask containing a magnetic stirring bar and **4c** (128.2 mg, 0.3 mmol) in THF (50 mL), was added 10% aqueous sodium hydroxide solution (6.0 mL). Then the reaction mixture was stirred at 65 °C for 5 h. The reaction mixture was cooled to room temperature and acidified with saturated aqueous $KHSO_4$ solution (to PH~3.0), and the resulting precipitate was collected by filtration, washed with water (5.0 mL) and dried under *vacuo* to give 112.2 mg pure product **8c** as yellow solid (84 % yield), mp: > 280 °C. ^{1}H NMR (400 MHz, DMSO) (δ, ppm) δ 8.92 (d, J = 8.3 Hz, 1H), 8.02 (d, J = 8.4 Hz, 1H), 7.74 – 7.68 (m, 1H), 7.66 (t, J = 4.0 Hz, 2H), 7.58 – 7.52 (m, 1H), 7.43 (s, 1H), 7.33 – 7.17 (m, 5H), 7.02 (s, 1H), 5.98 (dd, 2H); ^{13}C NMR (101 MHz, DMSO) (δ, ppm) δ 170.7, 155.3, 152.1, 143.7, 142.8, 135.8, 131.7, 130.62, 130.61, 129.3, 128.62, 128.61, 128.6, 127.4, 127.2, 125.9, 124.6, 124.2, 123.0, 121.6, 120.3, 83.8, 68.9. HRMS (TOF MS ESI) calculated for $C_{25}H_{17}BrNaO_3$[M + Na]$^+$, 467.0259; found, 467.0266.

Supplementary Figures

1D-Noe Study of 4g and 4g’

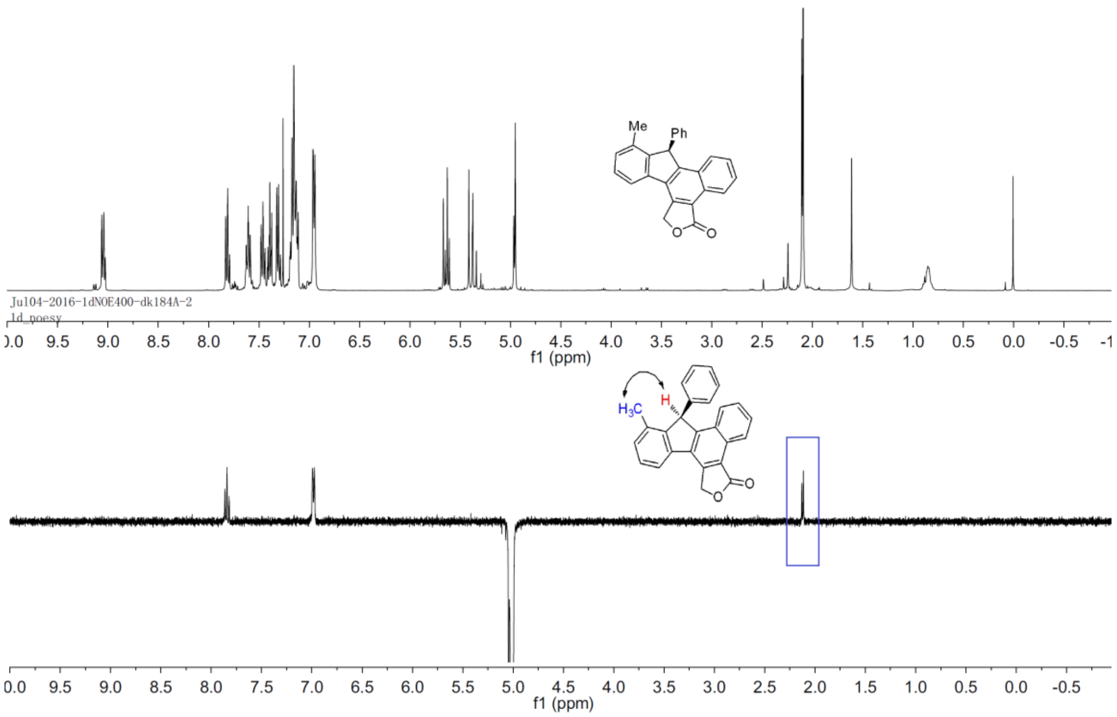

Supplementary Figure 1. 1D-Noe Study of 4g (400 MHz, $CDCl_3$)

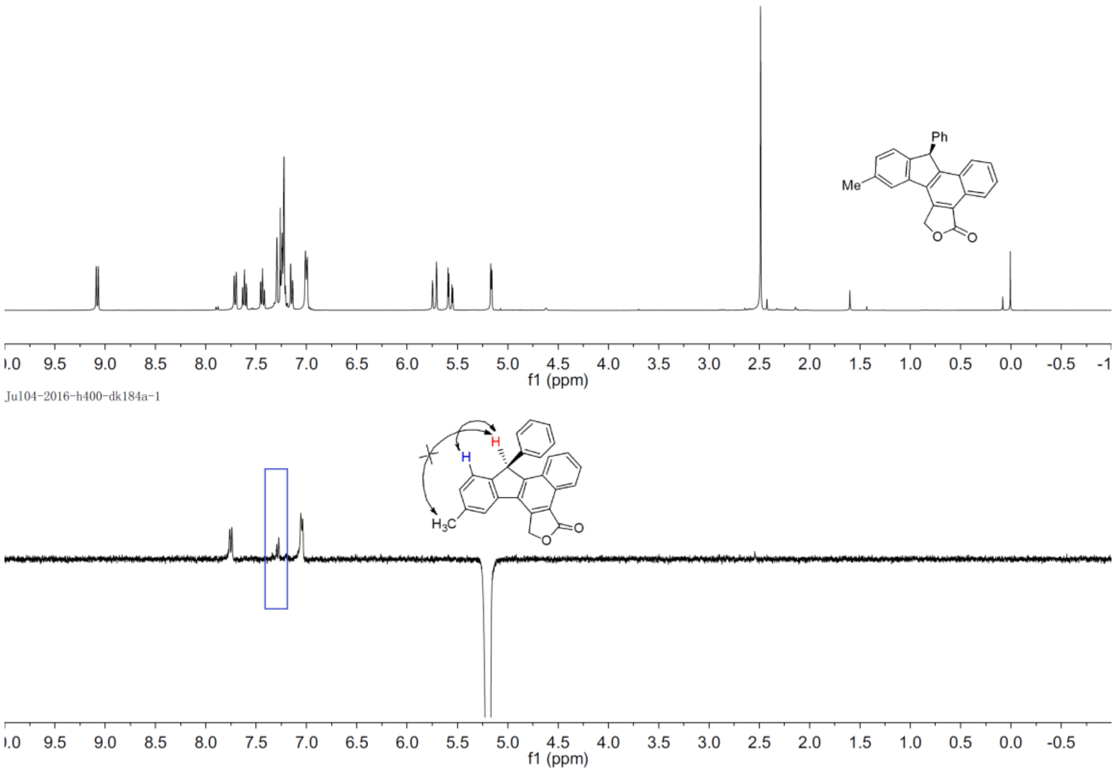

Supplementary Figure 2. 1D-Noe Study of 4g’ (400 MHz, $CDCl_3$)

Control Reaction in NMR Tube

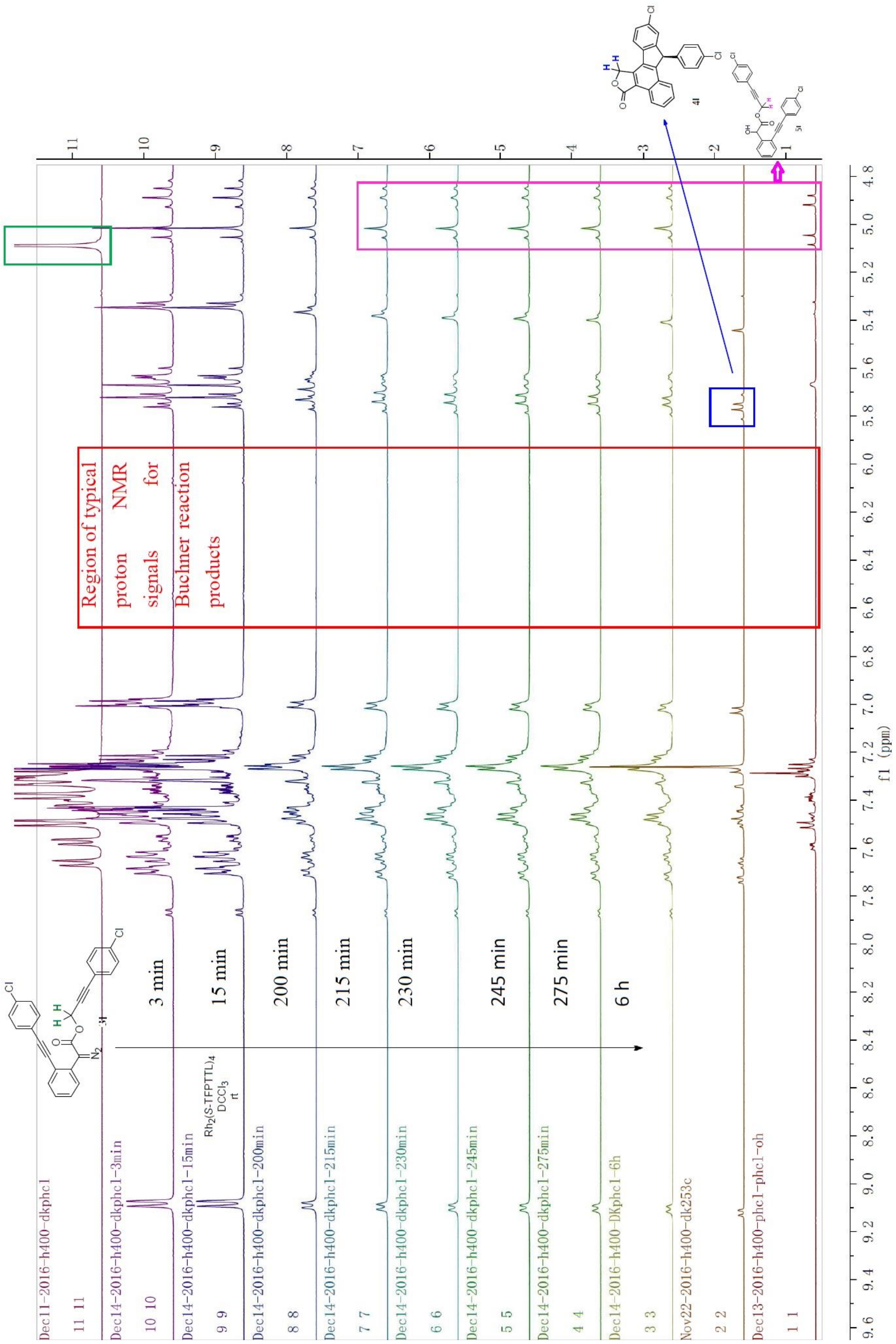

Supplementary Figure 3. Control Reaction in NMR Tube (400 MHz, CDCl₃)

NMR Spectra of New Compounds

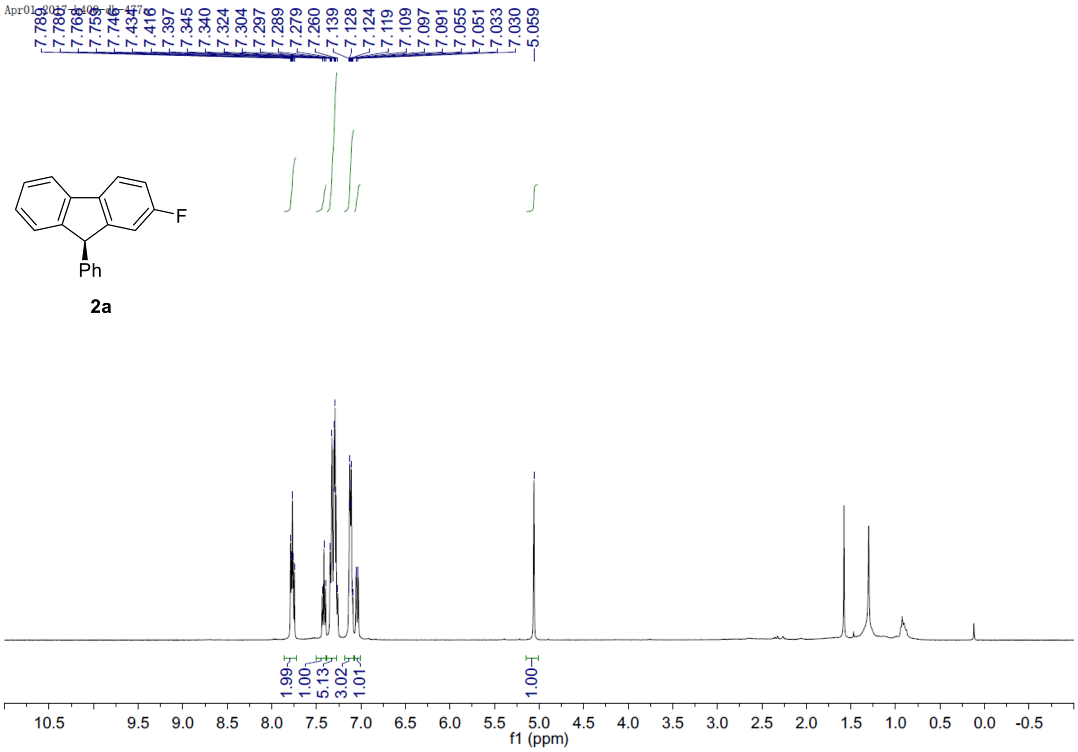

Supplementary Figure 4. ^{1}H NMR (400 MHz, CDCl$_3$) spectrum for 2a

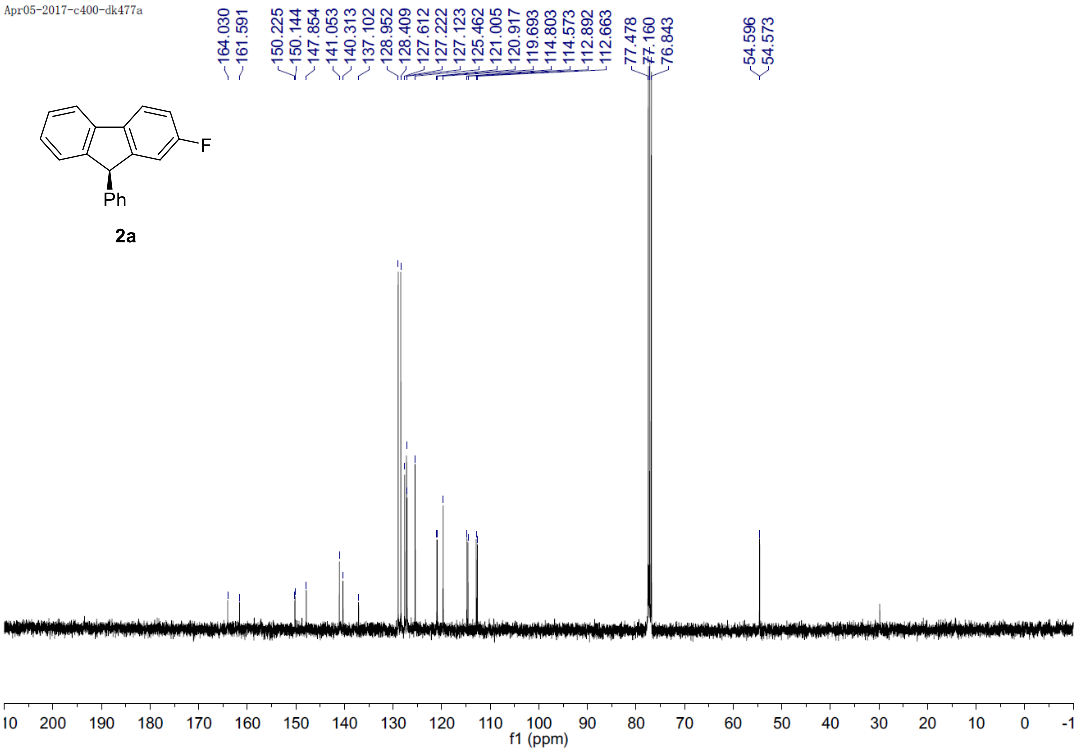

Supplementary Figure 5. ^{13}C NMR (100 MHz, CDCl$_3$) spectrum for 2a

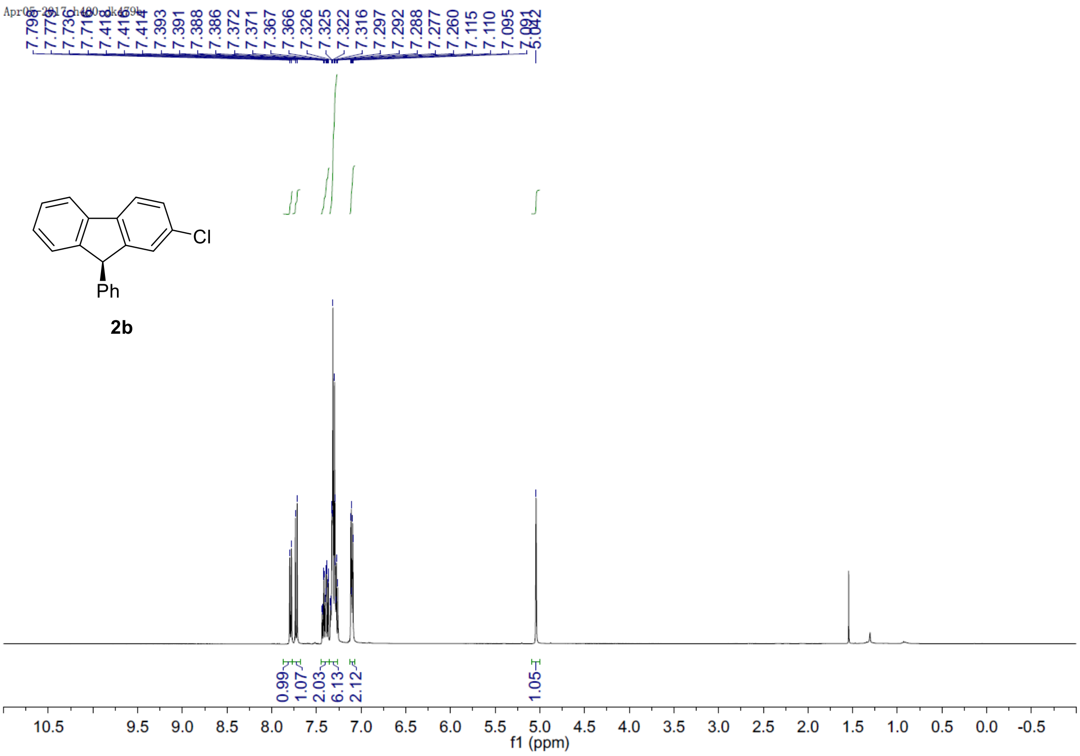

Supplementary Figure 6. ^{1}H NMR (400 MHz, $CDCl_3$) spectrum for 2b

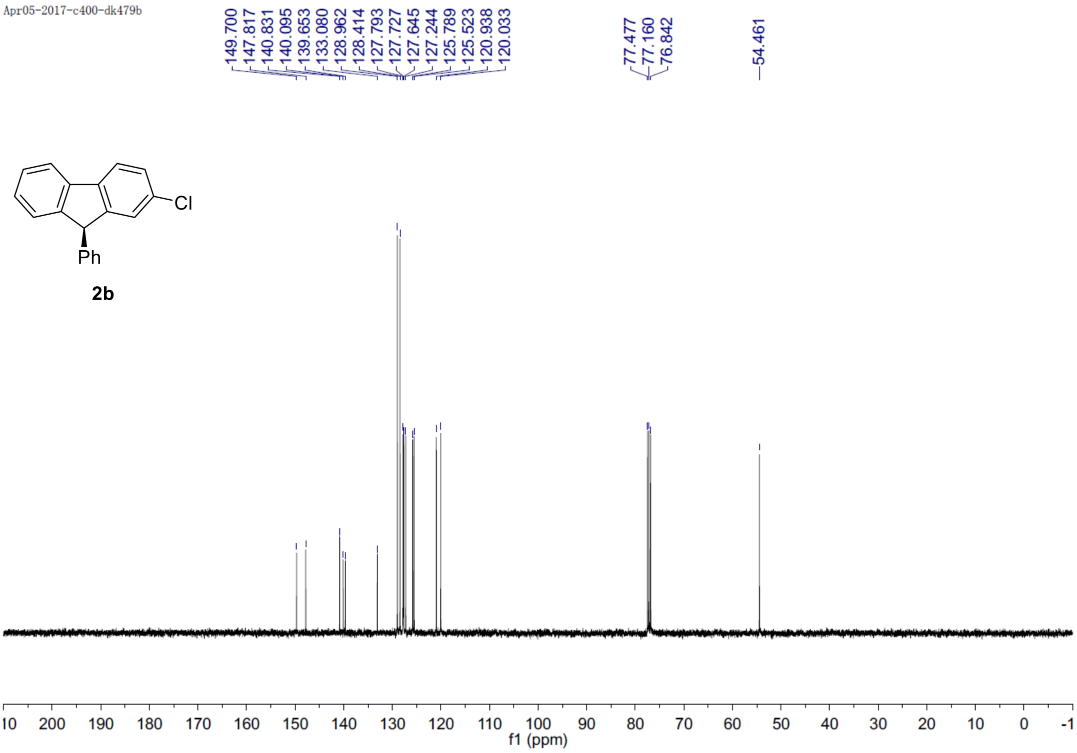

Supplementary Figure 7. ^{13}C NMR (100 MHz, $CDCl_3$) spectrum for 2b

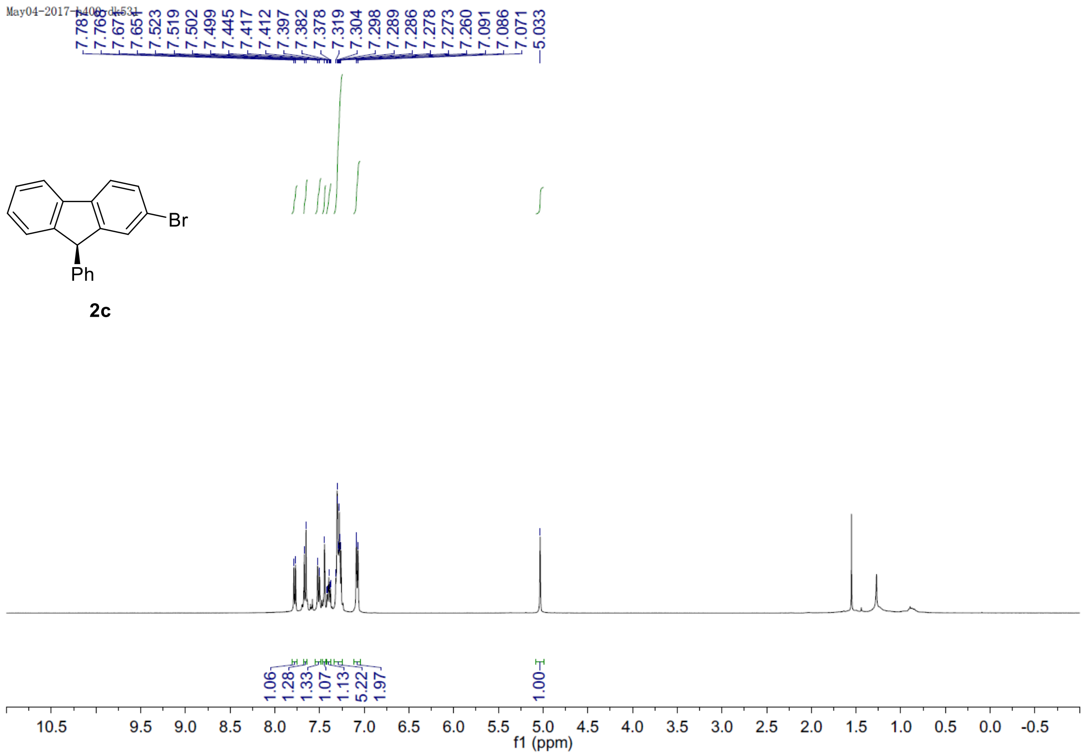

Supplementary Figure 8. ^{1}H NMR (400 MHz, $CDCl_3$) spectrum for 2c

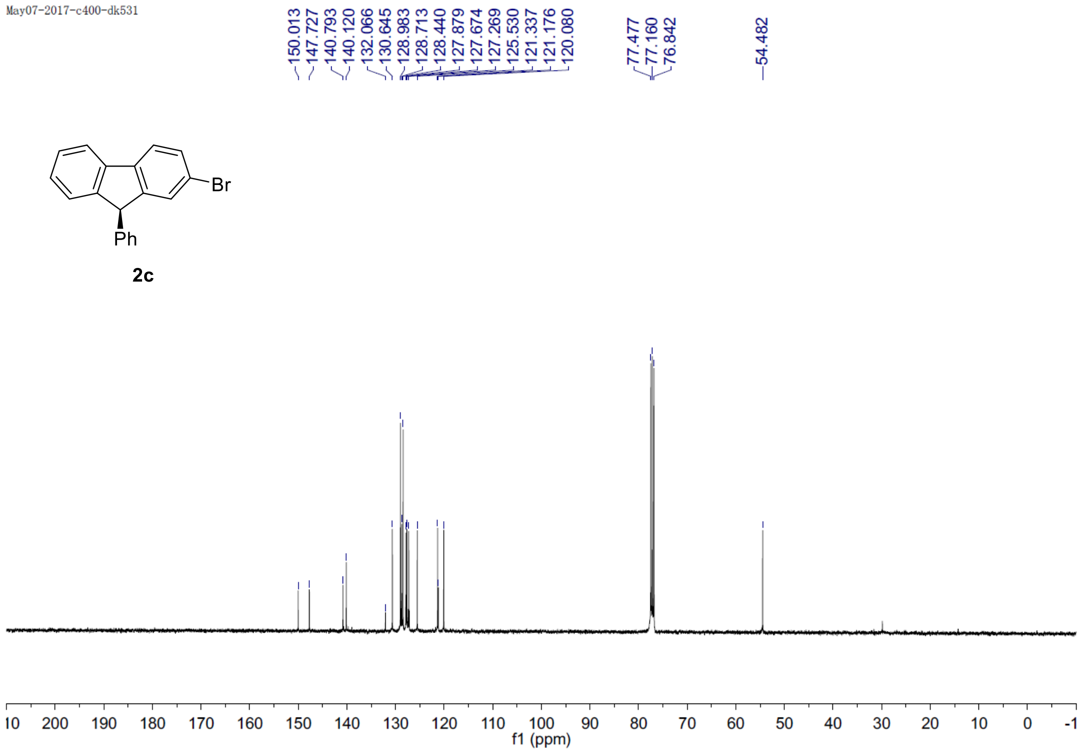

Supplementary Figure 9. ^{13}C NMR (100 MHz, $CDCl_3$) spectrum for 2c

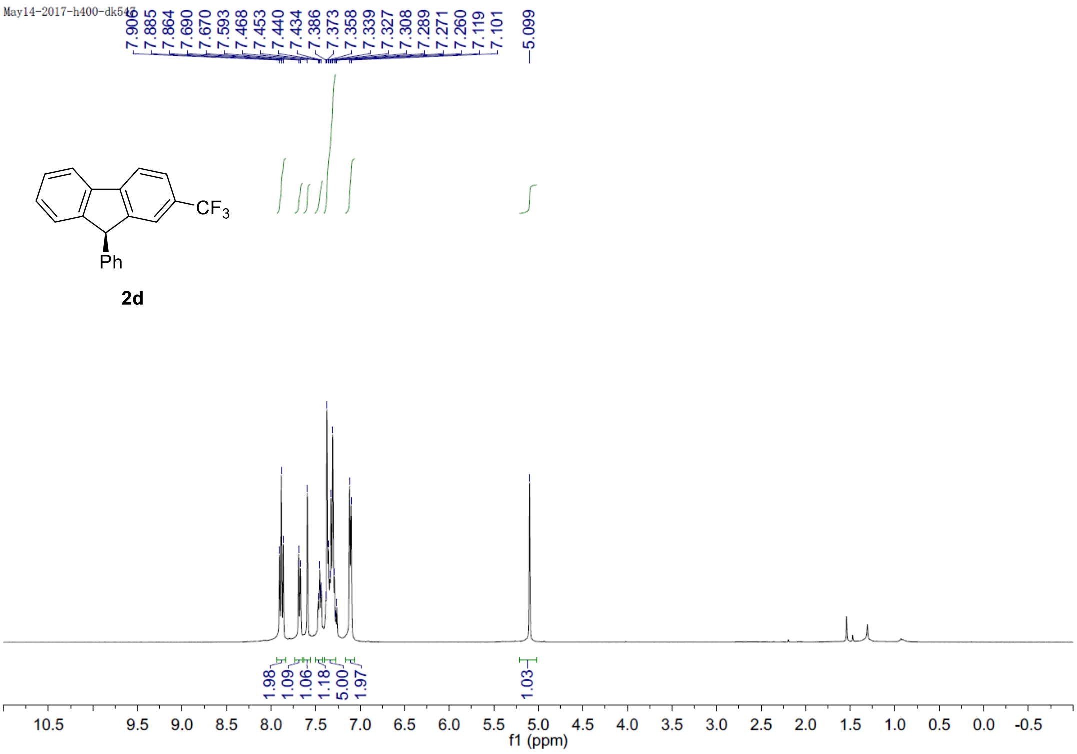

Supplementary Figure 10. **^{1}H NMR (400 MHz, $CDCl_3$) spectrum for 2d**

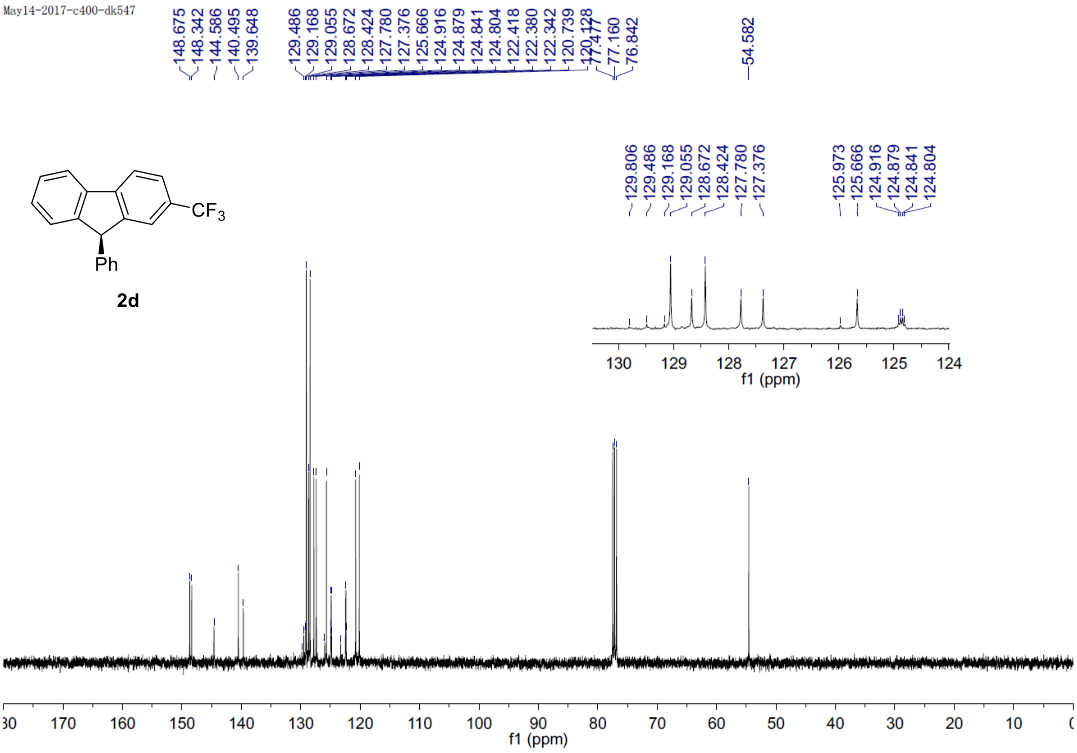

Supplementary Figure 11. **^{13}C NMR (100 MHz, $CDCl_3$) spectrum for 2d**

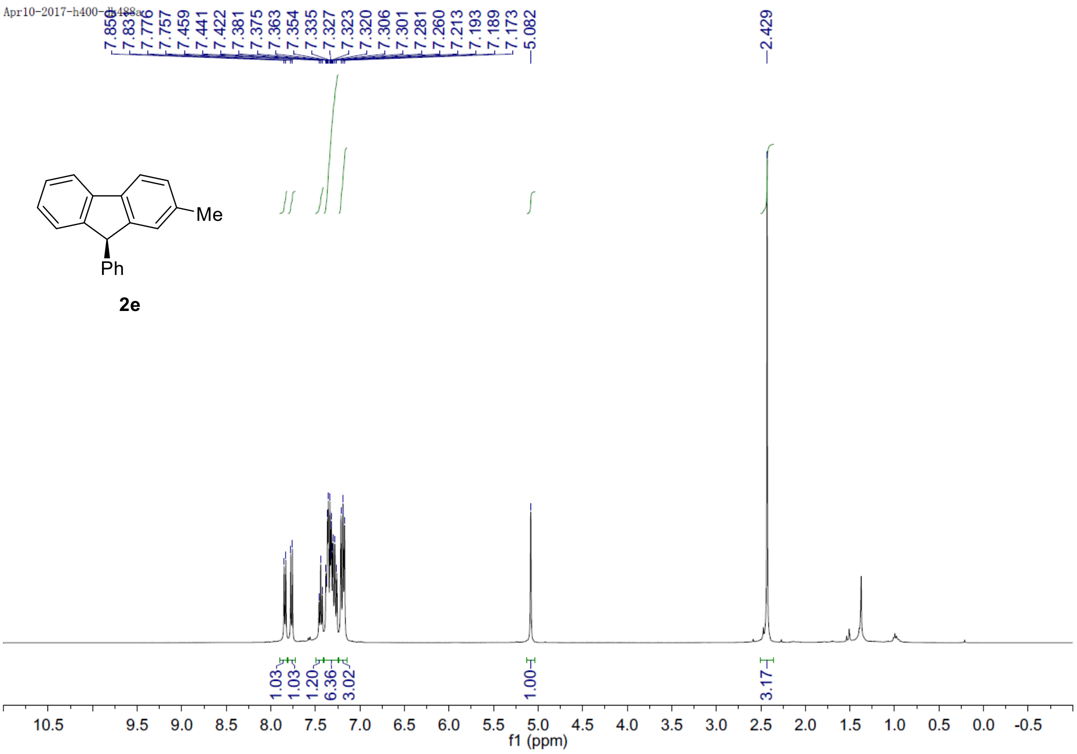

Supplementary Figure 12. ^{1}H NMR (400 MHz, $CDCl_3$) spectrum for 2e

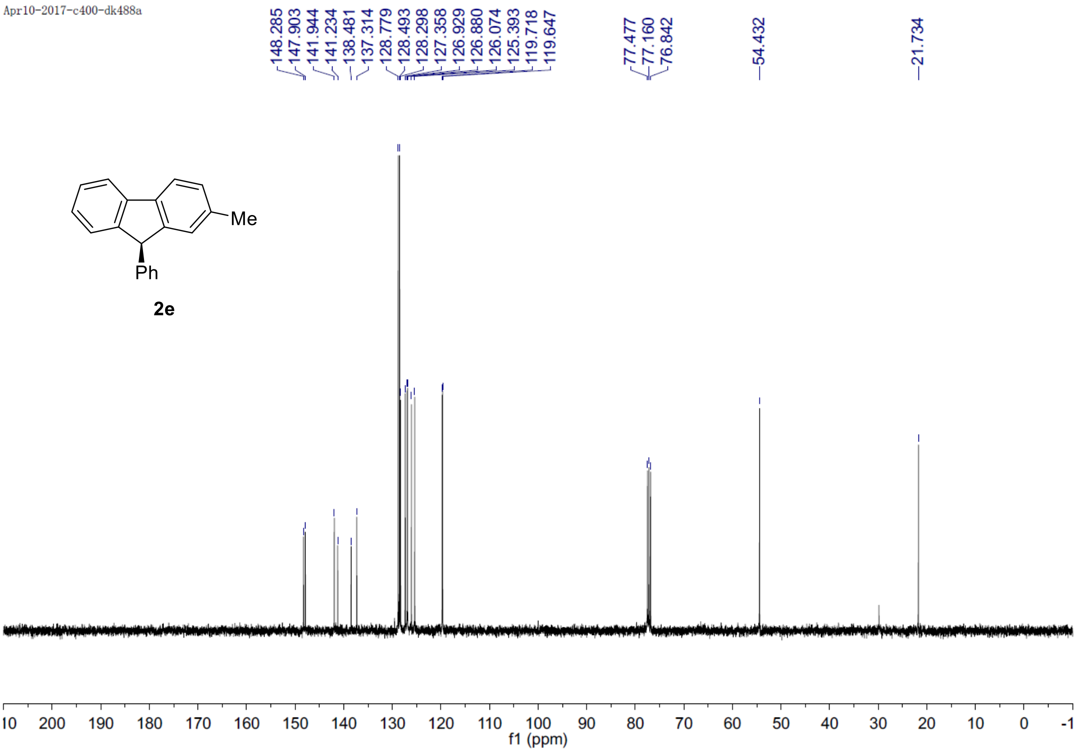

Supplementary Figure 13. ^{13}C NMR (100 MHz, $CDCl_3$) spectrum for 2e

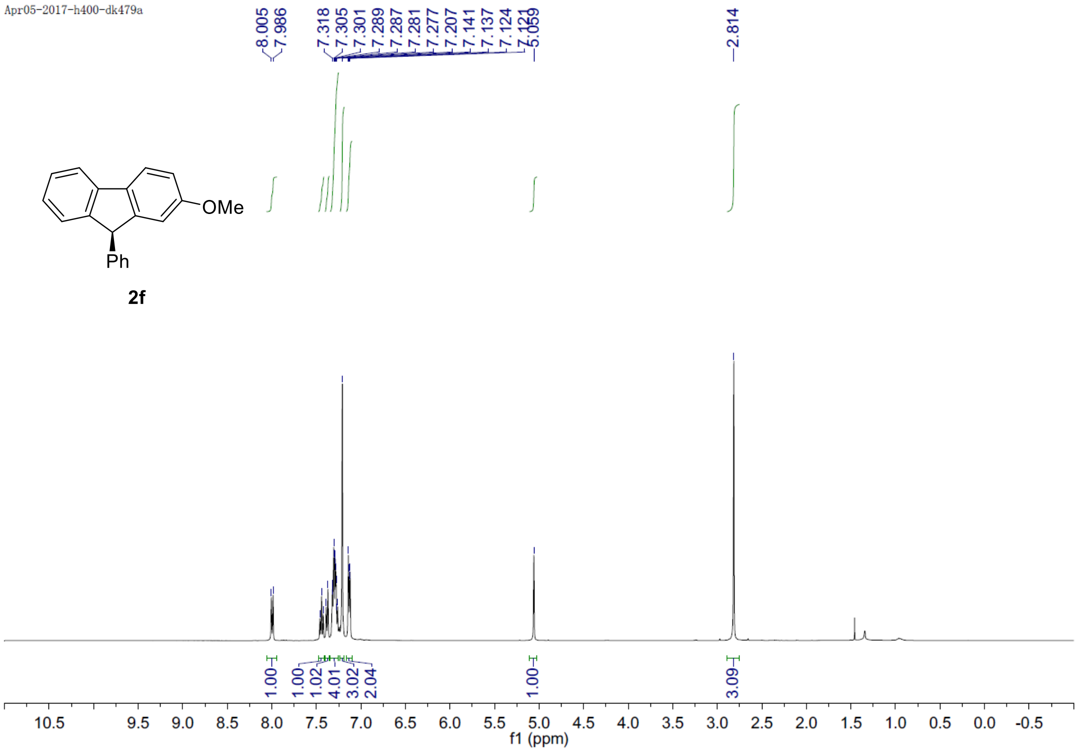

Supplementary Figure 14. ^{1}H NMR (400 MHz, $CDCl_3$) spectrum for 2f

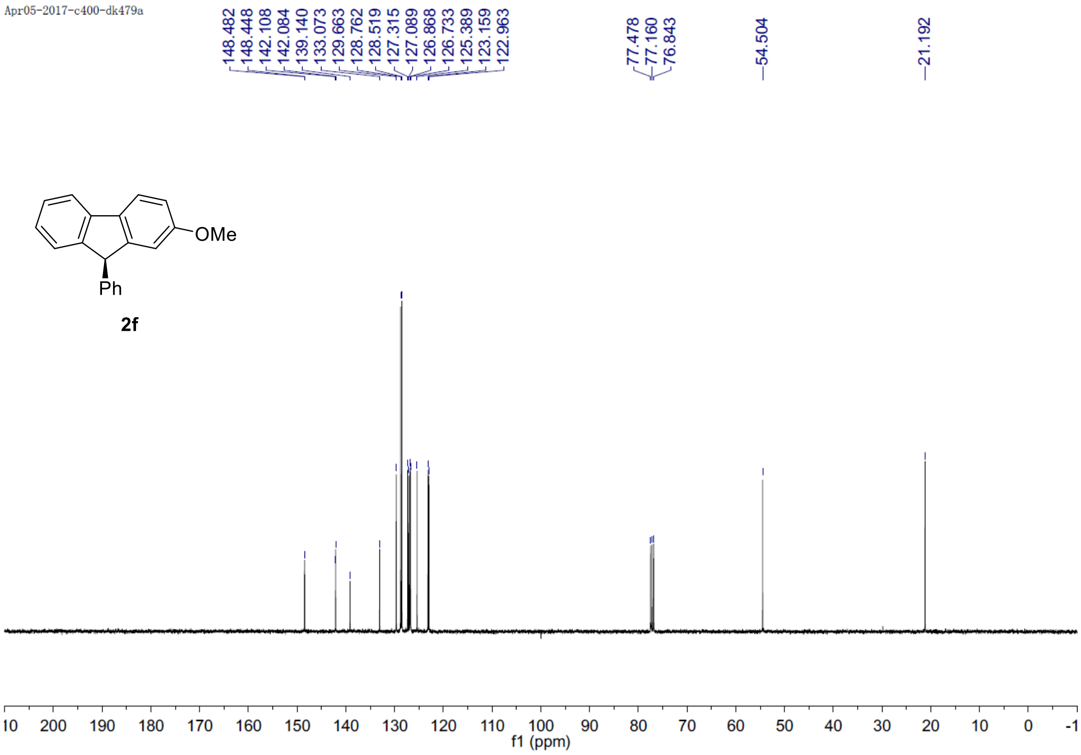

Supplementary Figure 15. ^{13}C NMR (100 MHz, $CDCl_3$) spectrum for 2f

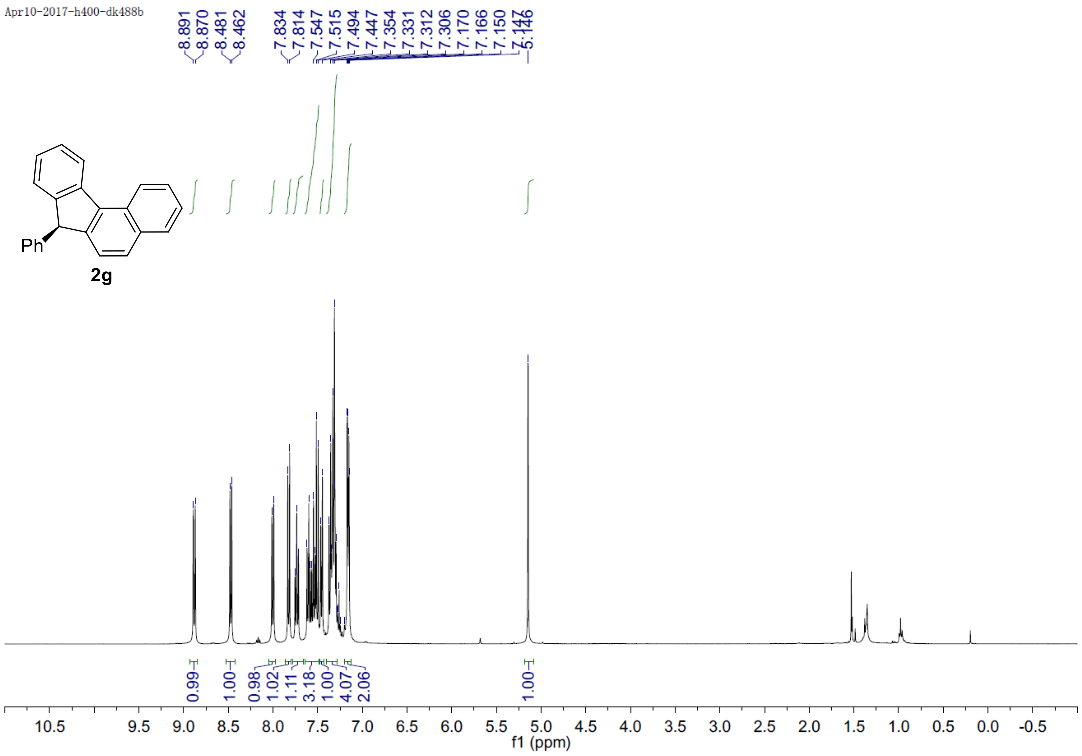

Supplementary Figure 16. ^{1}H NMR (400 MHz, $CDCl_3$) spectrum for 2g

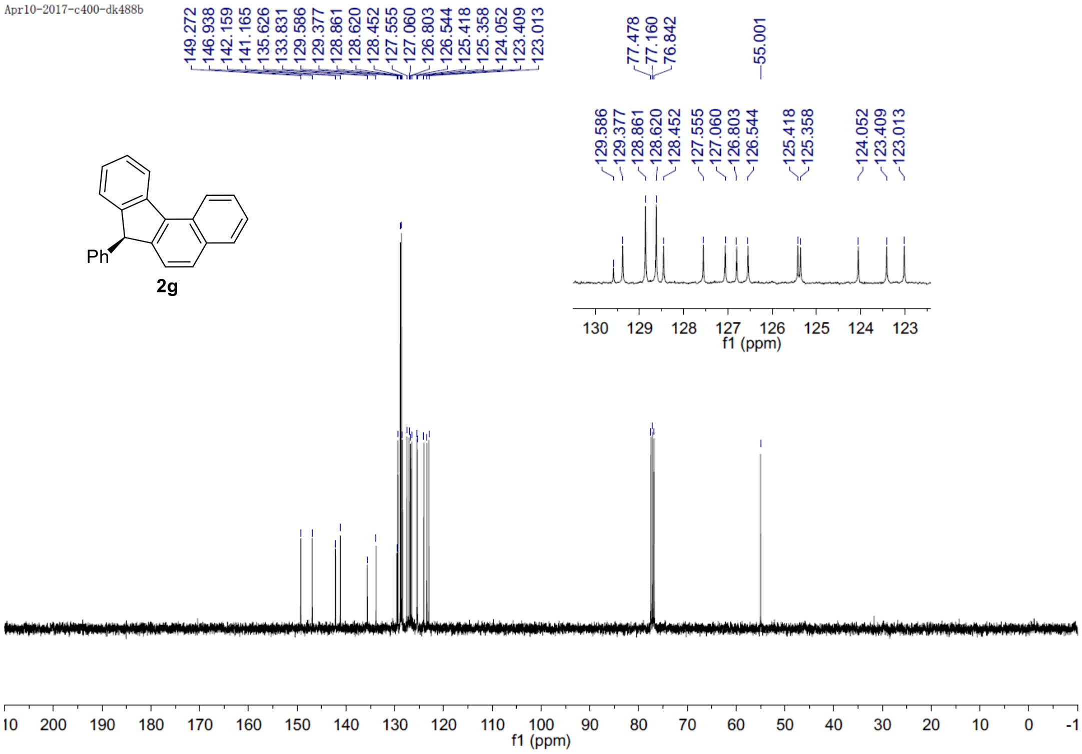

Supplementary Figure 17. ^{13}C NMR (100 MHz, $CDCl_3$) spectrum for 2g

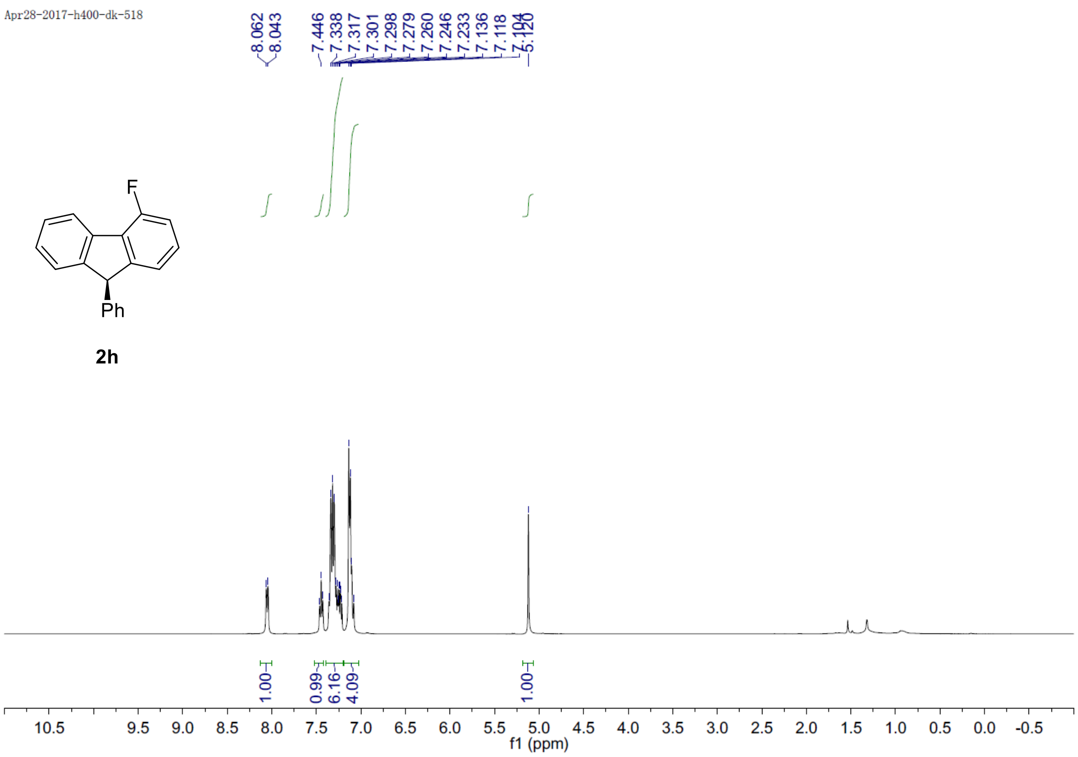

Supplementary Figure 18. ^{1}H NMR (400 MHz, $CDCl_3$) spectrum for 2h

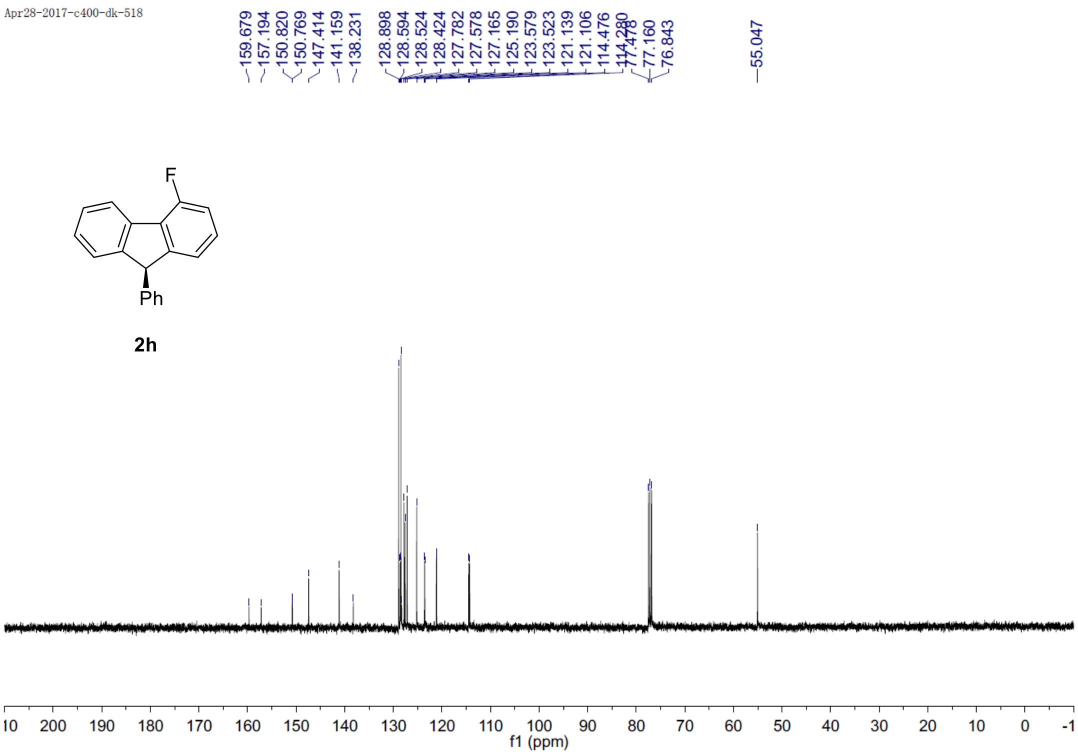

Supplementary Figure 19. ^{13}C NMR (100 MHz, $CDCl_3$) spectrum for 2h

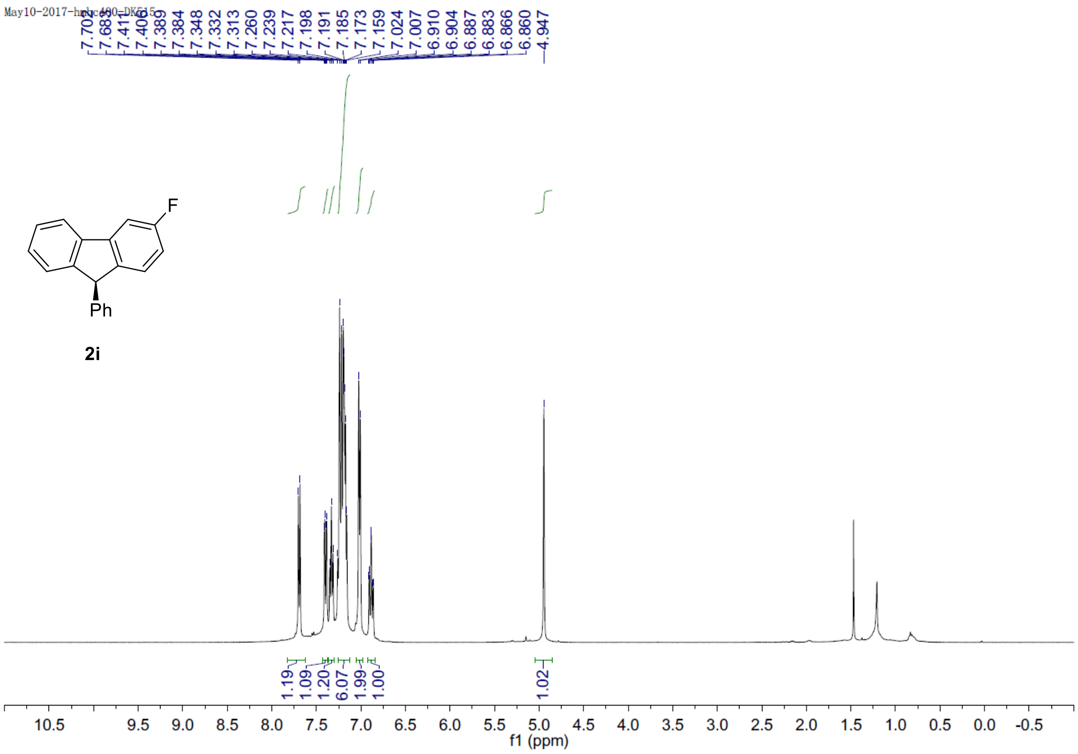

Supplementary Figure 20. ^{1}H **NMR (400 MHz,** $CDCl_3$**) spectrum for 2i**

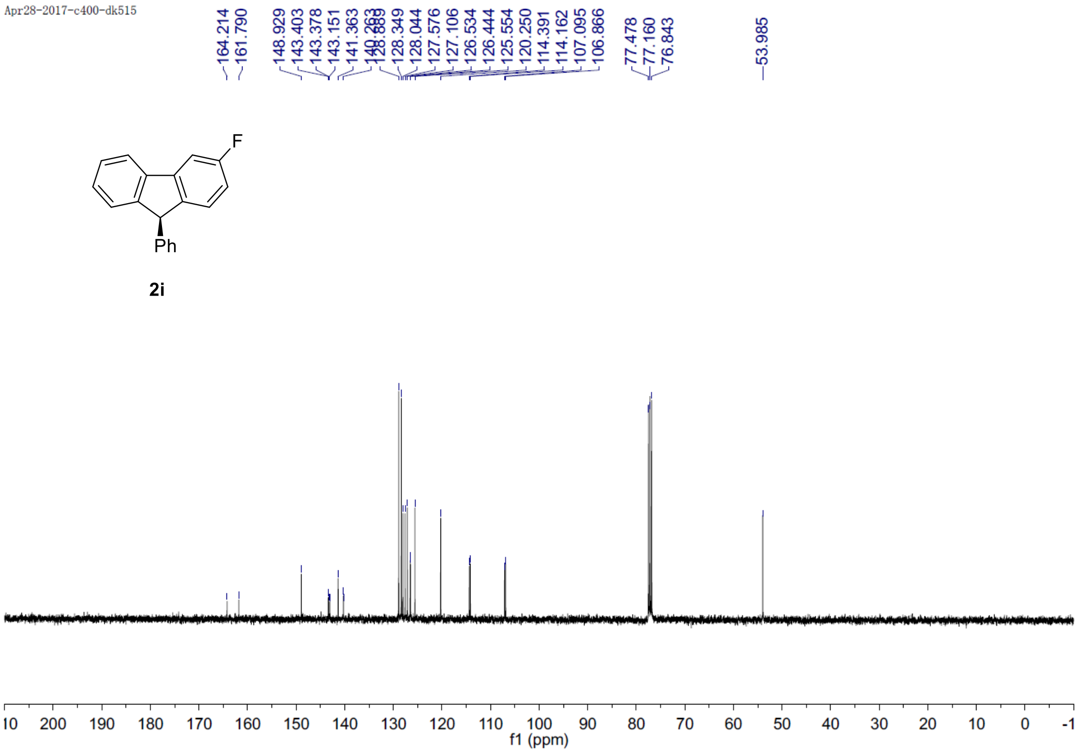

Supplementary Figure 21. ^{13}C **NMR (100 MHz,** $CDCl_3$**) spectrum for 2i**

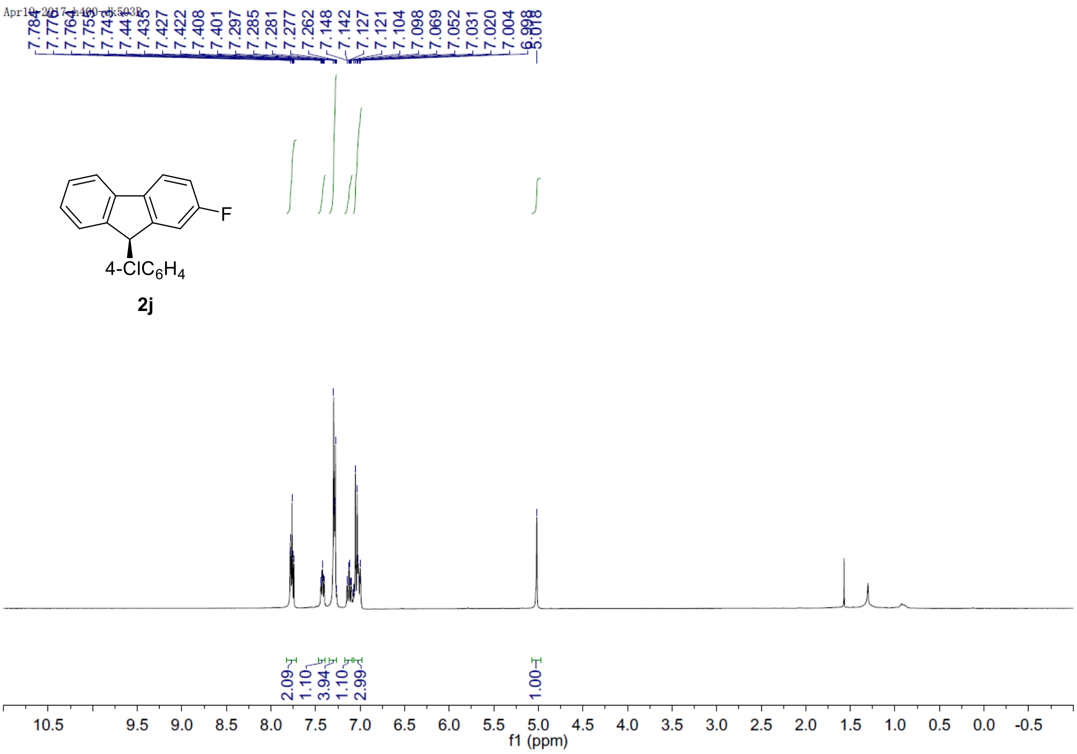

Supplementary Figure 22. ^{1}H **NMR (400 MHz,** $CDCl_3$**) spectrum for 2j**

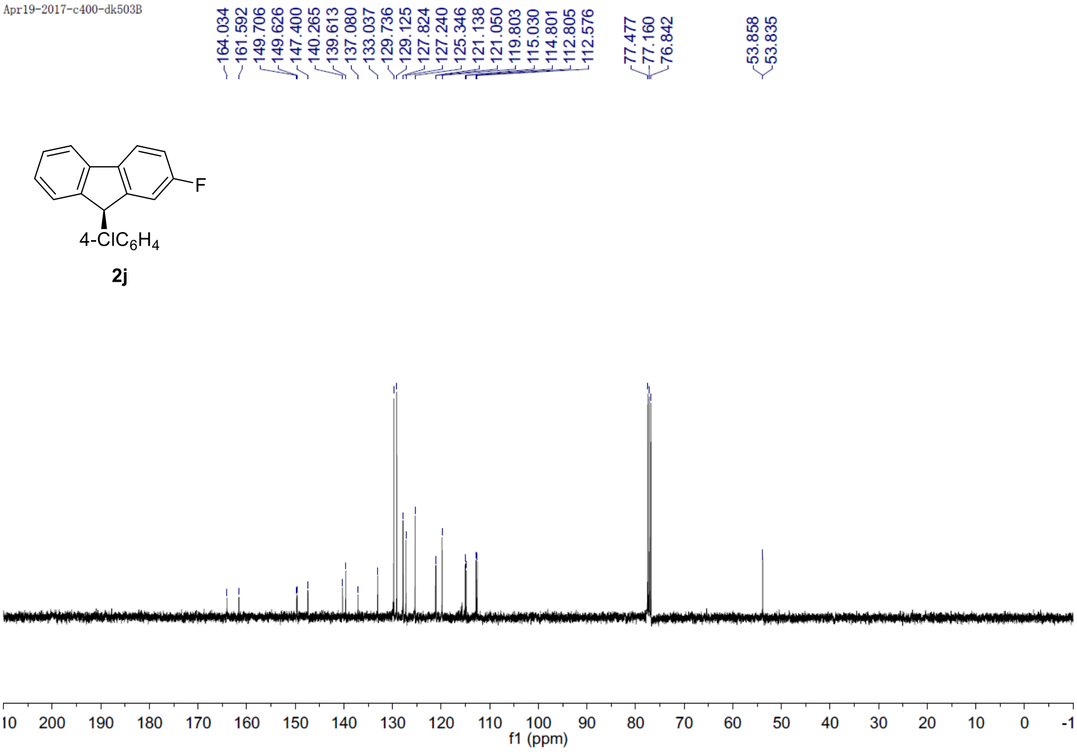

Supplementary Figure 23. ^{13}C **NMR (100 MHz,** $CDCl_3$**) spectrum for 2j**

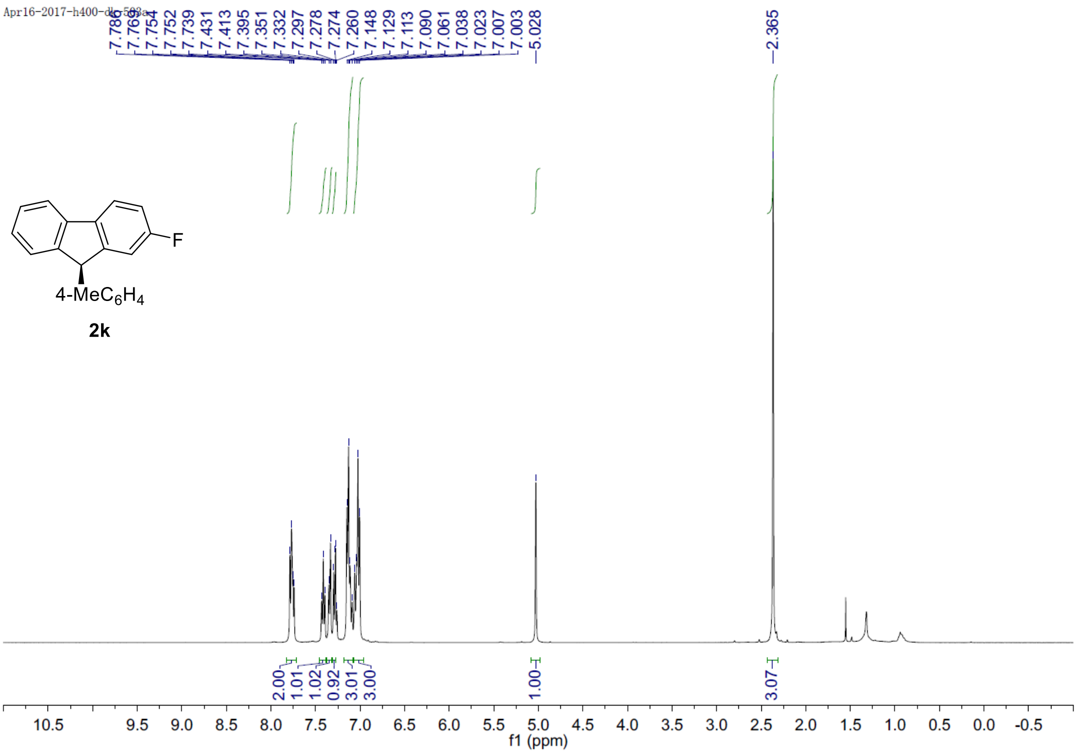

Supplementary Figure 24. ^{1}H **NMR (400 MHz, $CDCl_3$) spectrum for 2k**

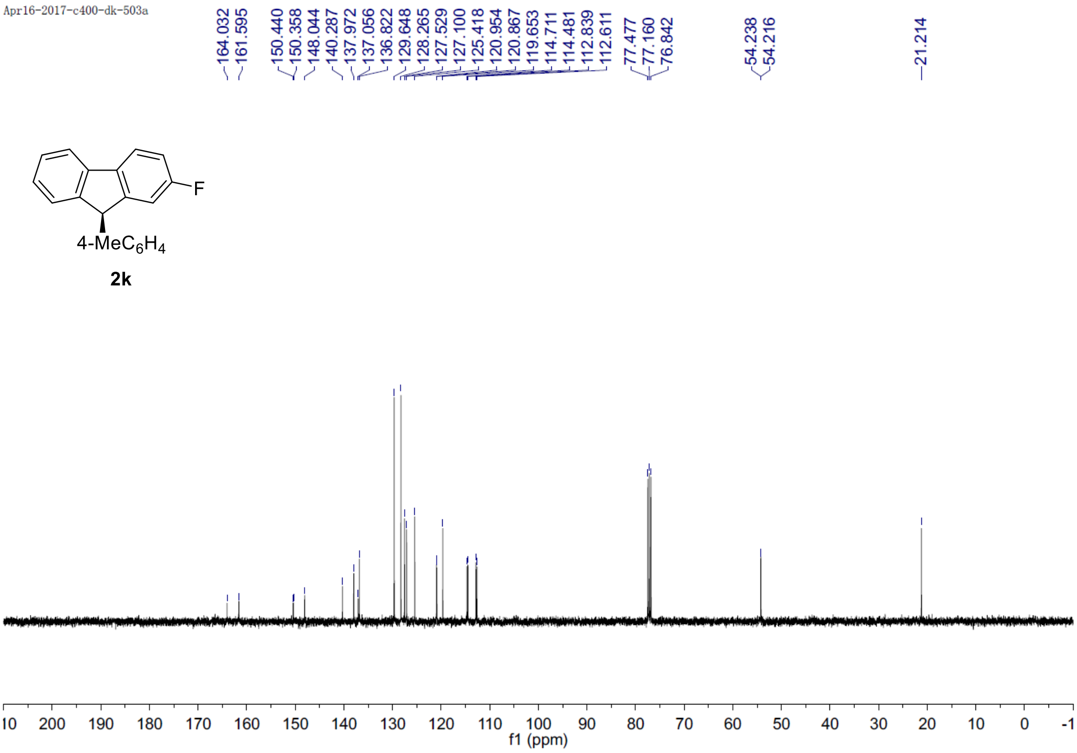

Supplementary Figure 25. ^{13}C **NMR (100 MHz, $CDCl_3$) spectrum for 2k**

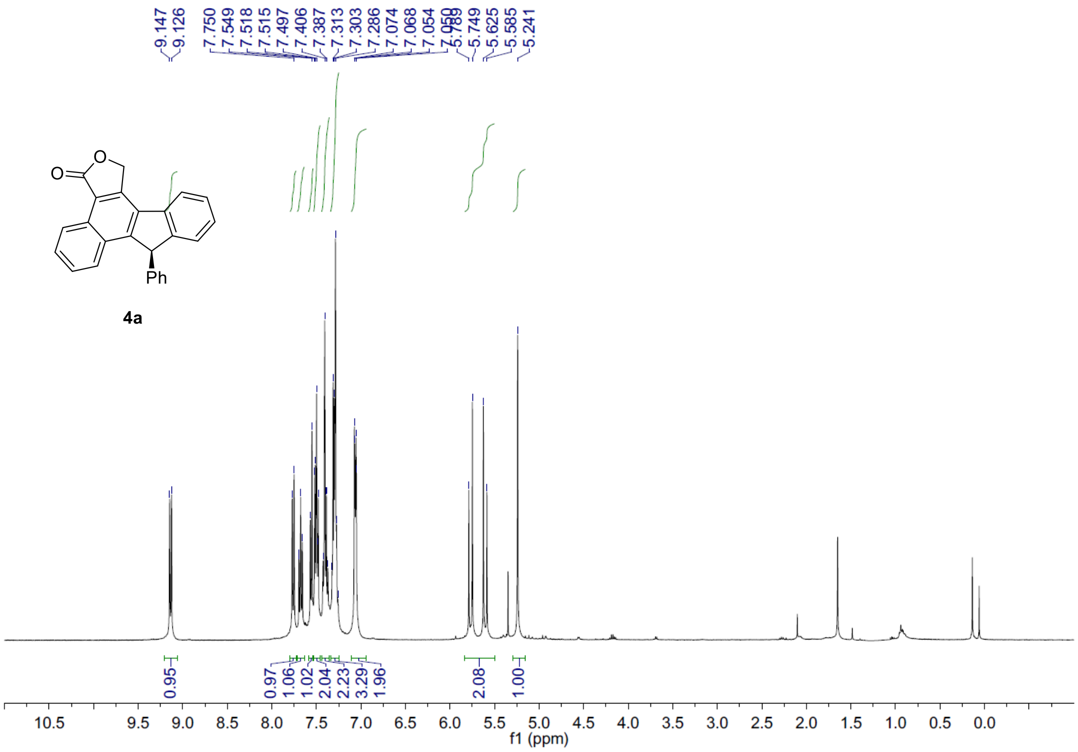

Supplementary Figure 26. ^{1}H NMR (400 MHz, $CDCl_3$) spectrum for 4a

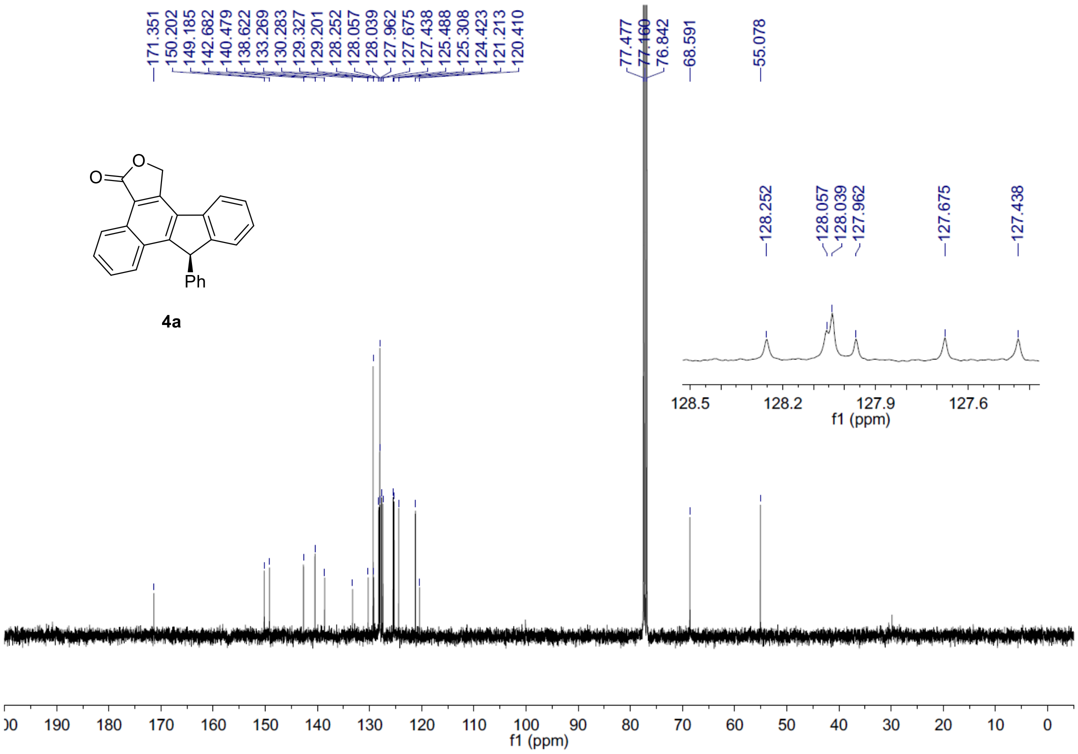

Supplementary Figure 27. ^{13}C NMR (100 MHz, $CDCl_3$) spectrum for 4a

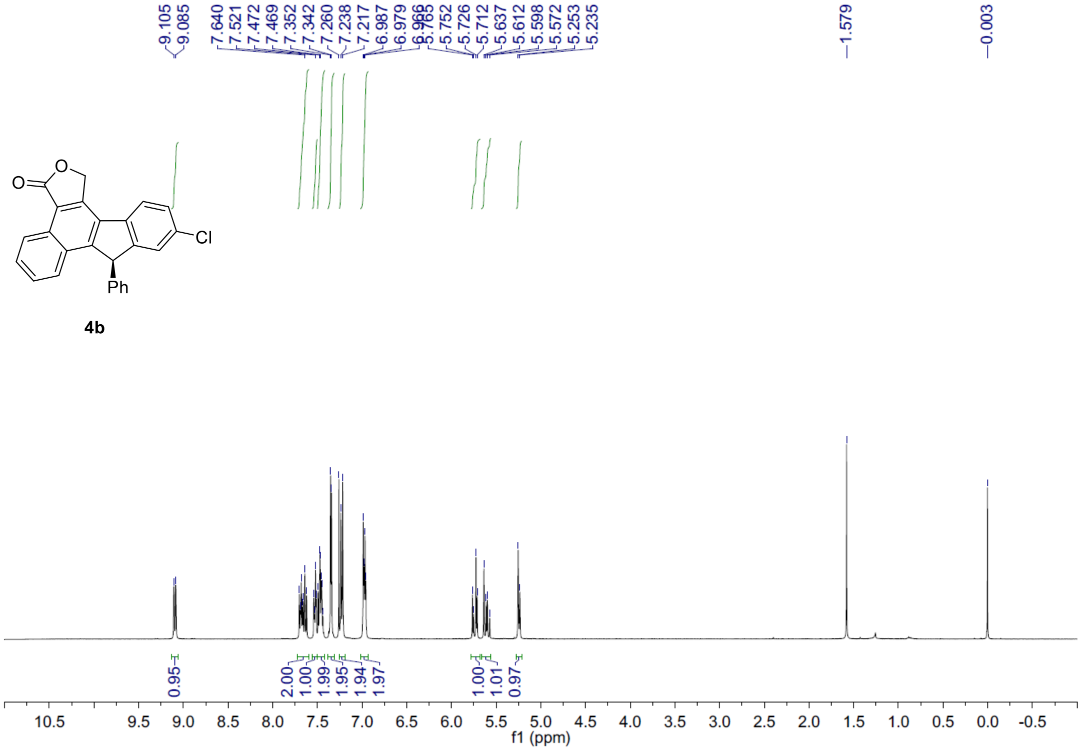

Supplementary Figure 28. ^{1}H NMR (400 MHz, $CDCl_3$) spectrum for 4b

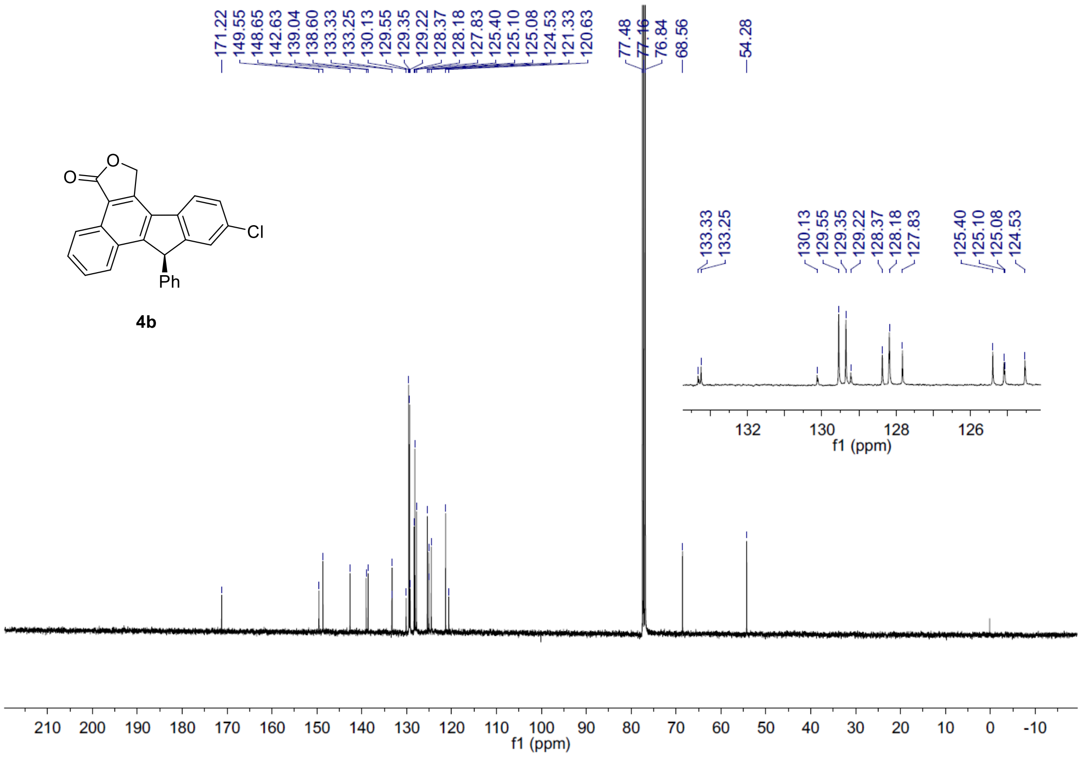

Supplementary Figure 29. ^{13}C NMR (100 MHz, $CDCl_3$) spectrum for 4b

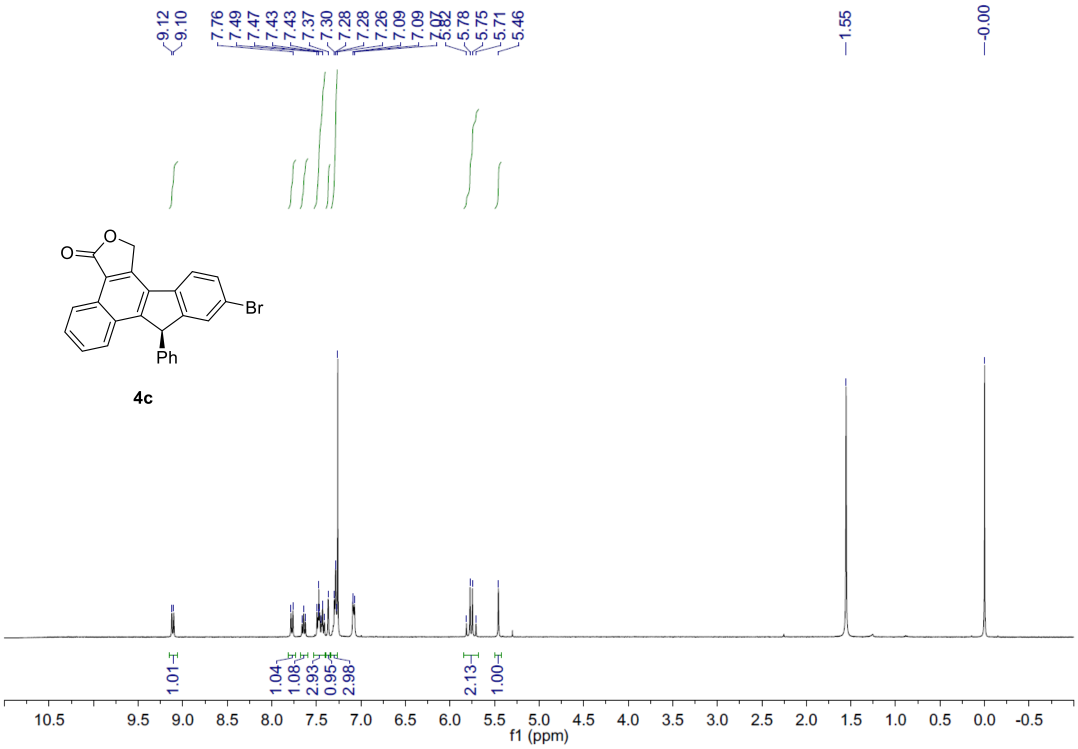

Supplementary Figure 30. ^{1}H NMR (400 MHz, $CDCl_3$) spectrum for 4c

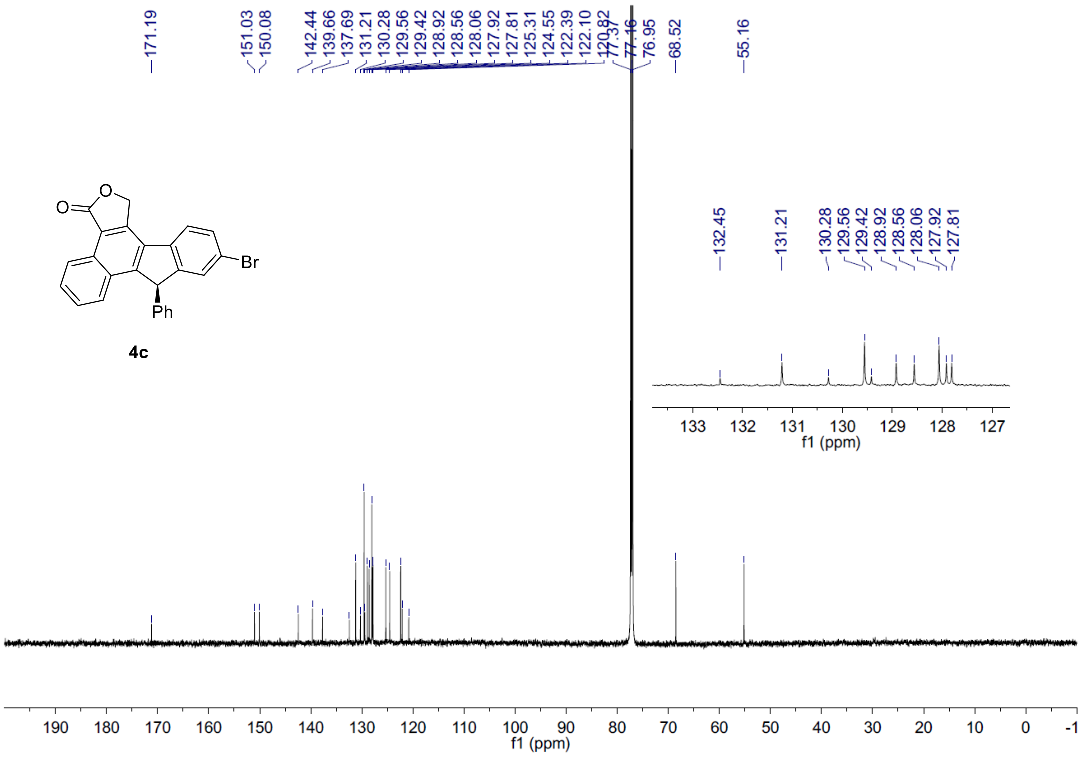

Supplementary Figure 31. ^{13}C NMR (100 MHz, $CDCl_3$) spectrum for 4c

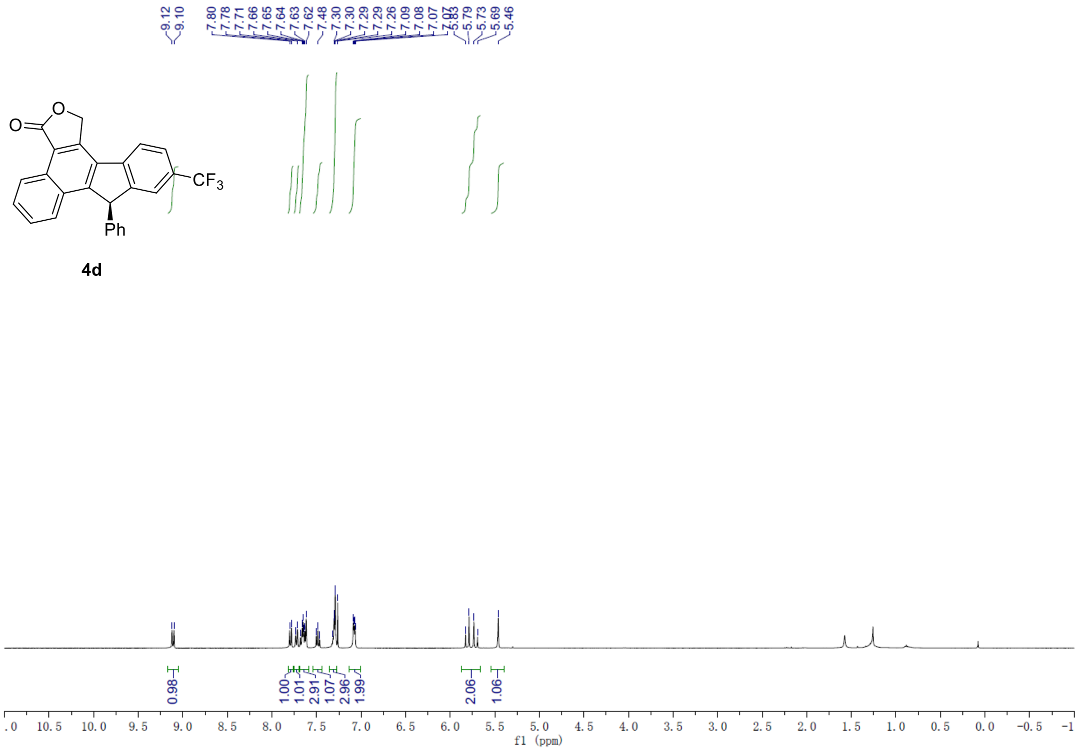

Supplementary Figure 32. ^{1}H NMR (400 MHz, $CDCl_3$) spectrum for 4d

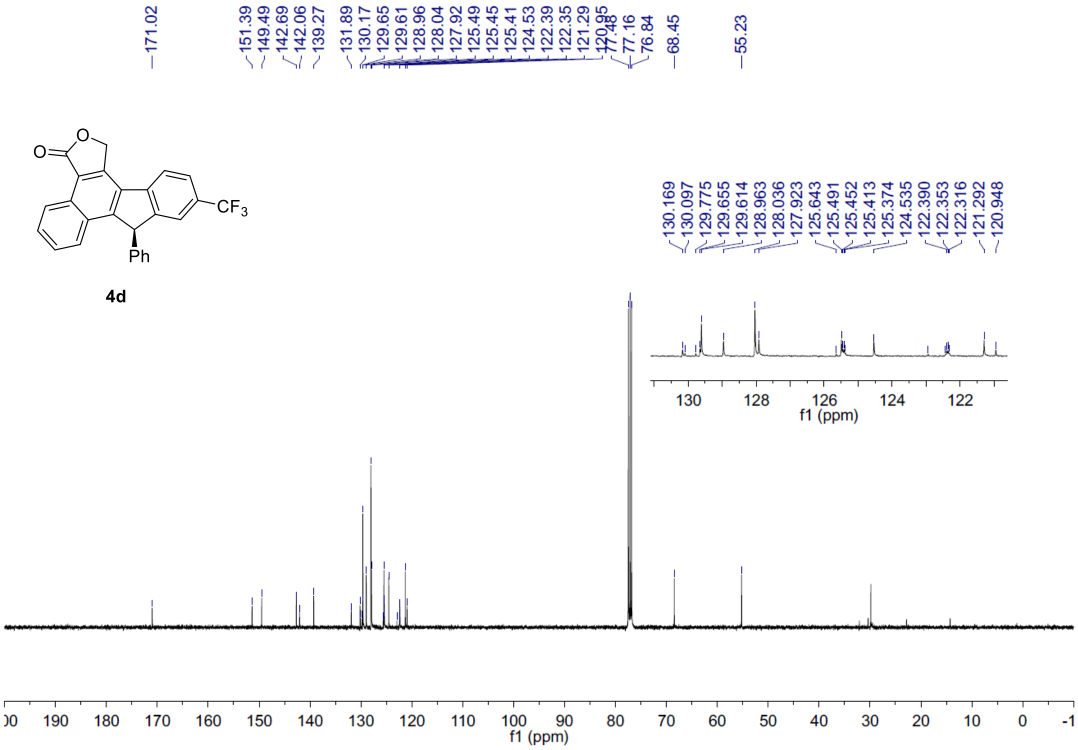

Supplementary Figure 33. ^{13}C NMR (100 MHz, $CDCl_3$) spectrum for 4d

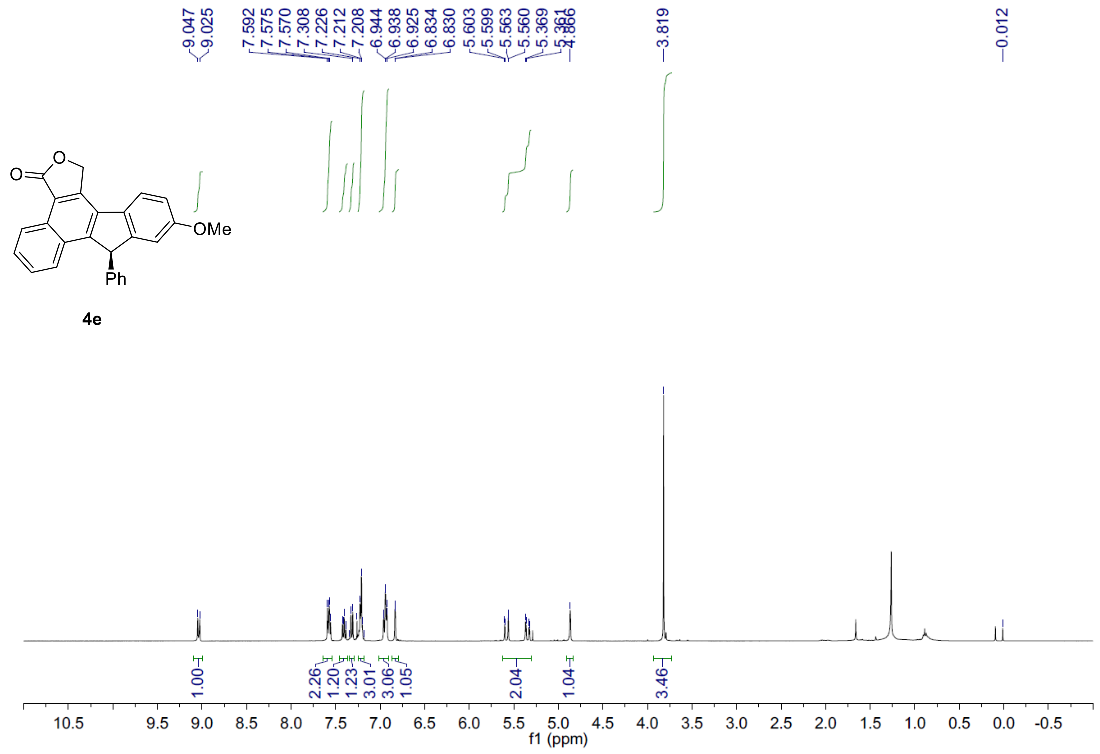

Supplementary Figure 34. ^{1}H NMR (400 MHz, $CDCl_3$) spectrum for 4e

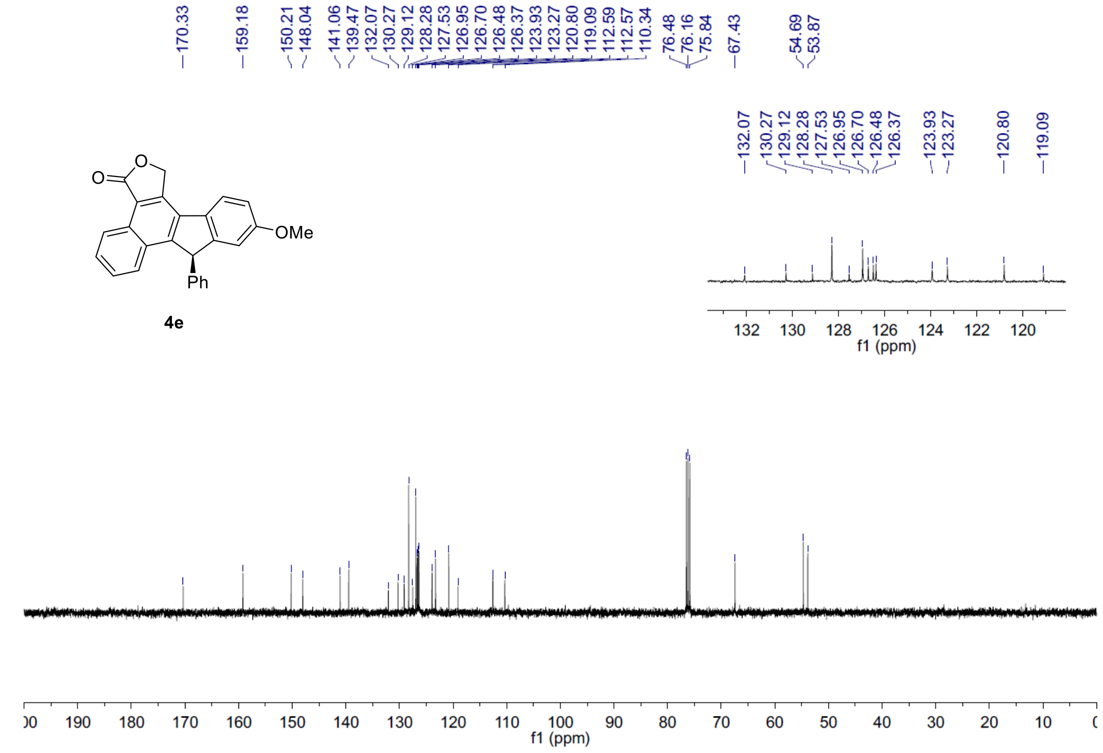

Supplementary Figure 35. ^{13}C NMR (100 MHz, $CDCl_3$) spectrum for 4e

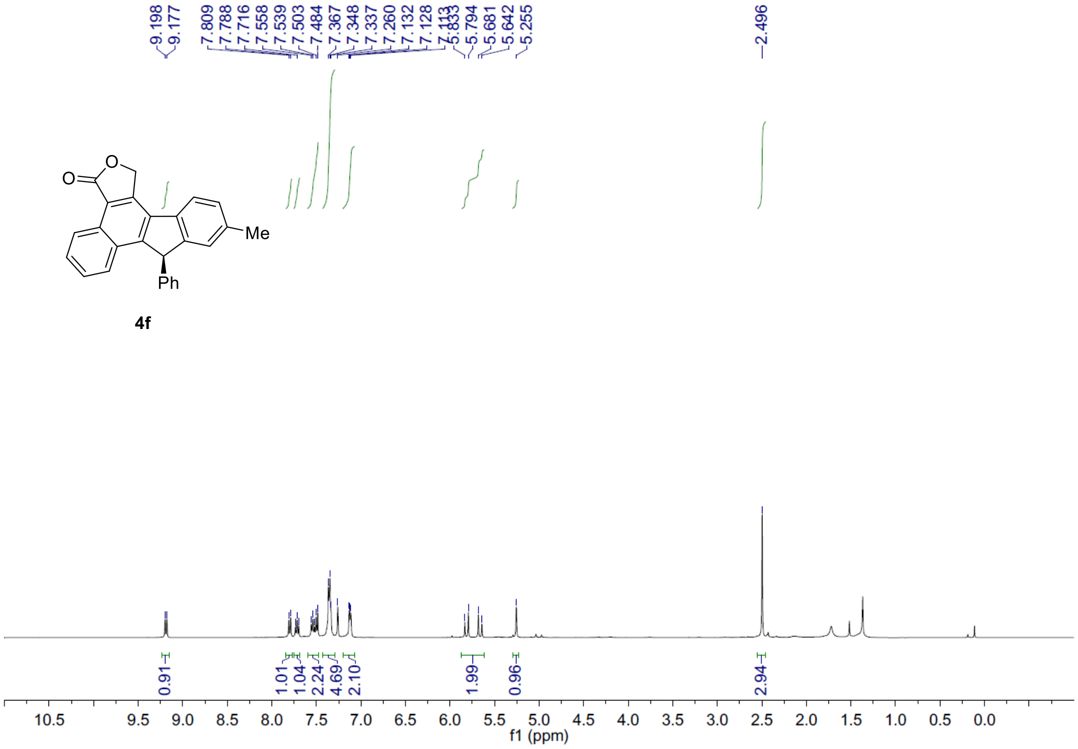

Supplementary Figure 36. ^{1}H NMR (400 MHz, $CDCl_3$) spectrum for 4f

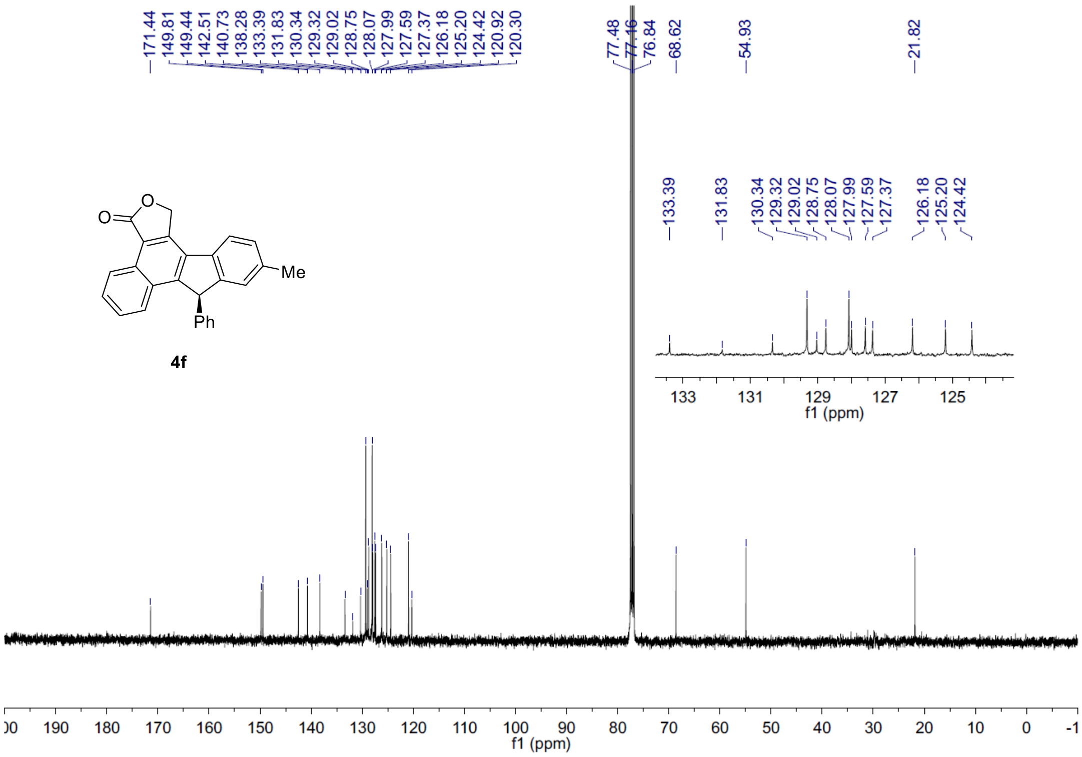

Supplementary Figure 37. ^{13}C NMR (100 MHz, $CDCl_3$) spectrum for 4f

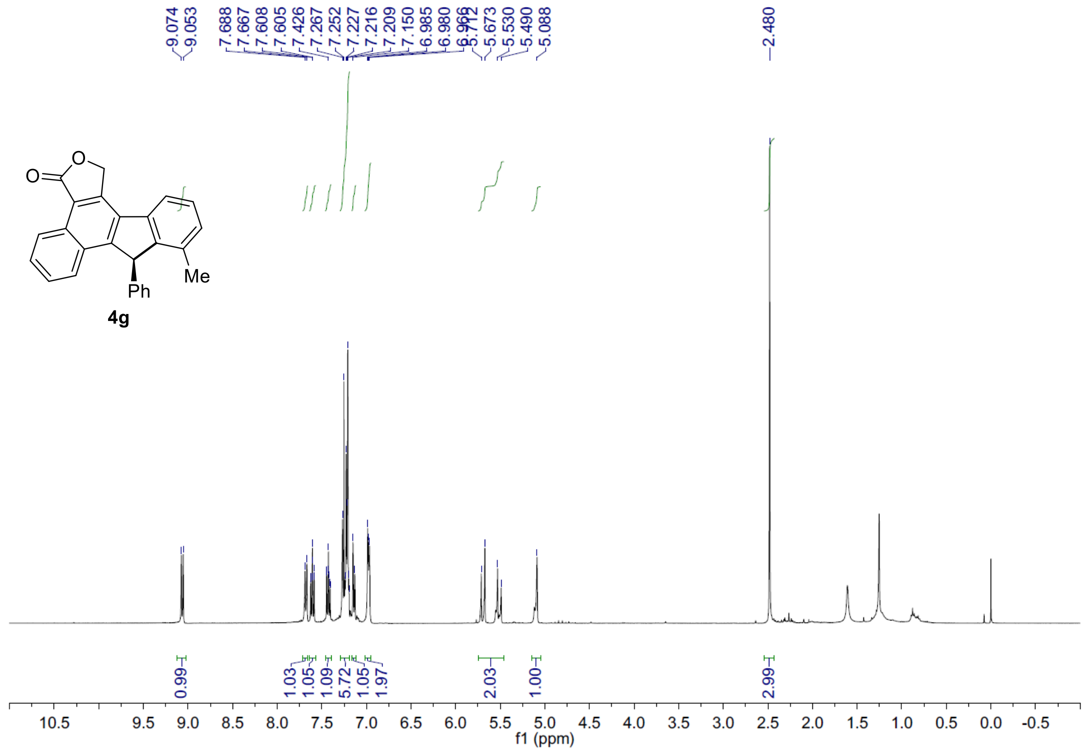

Supplementary Figure 38. ^{1}H NMR (400 MHz, $CDCl_3$) spectrum for 4g

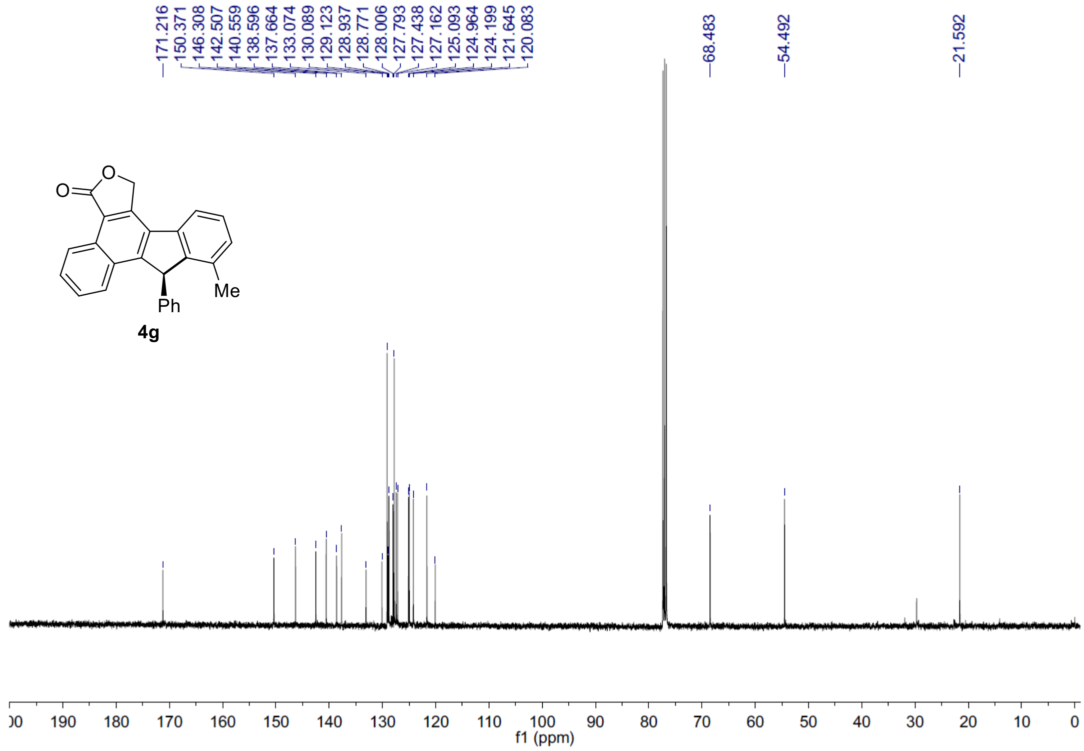

Supplementary Figure 39. ^{13}C NMR (100 MHz, $CDCl_3$) spectrum for 4g

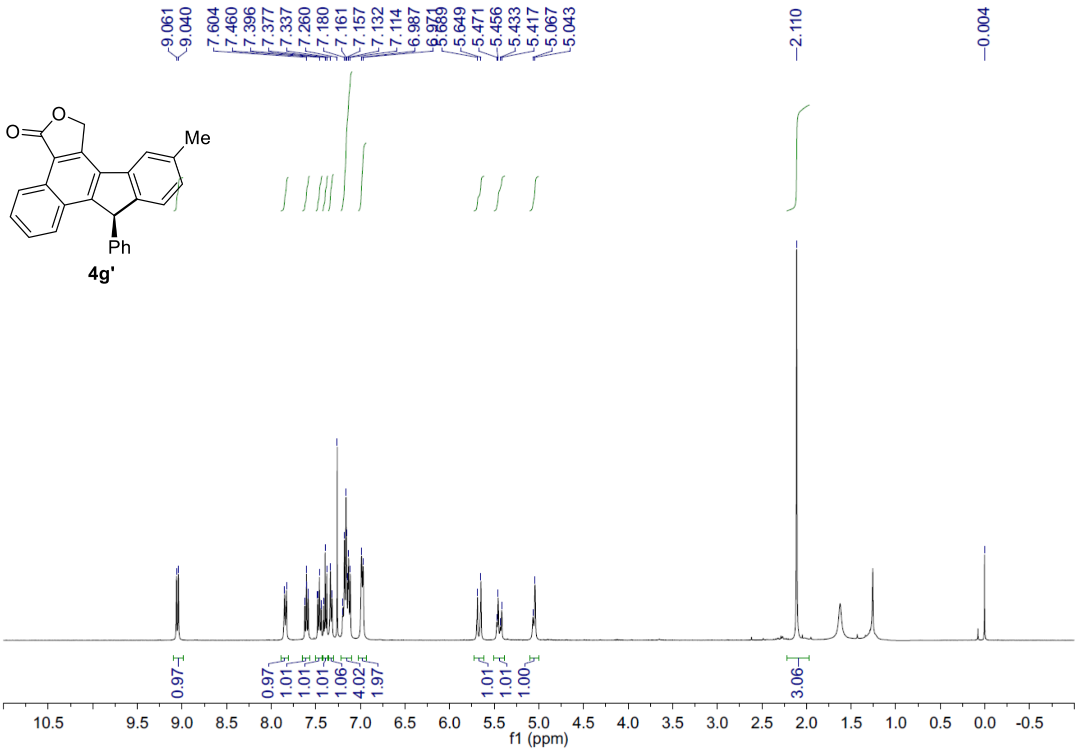

Supplementary Figure 40. ^{1}H **NMR (400 MHz, $CDCl_3$) spectrum for 4g'**

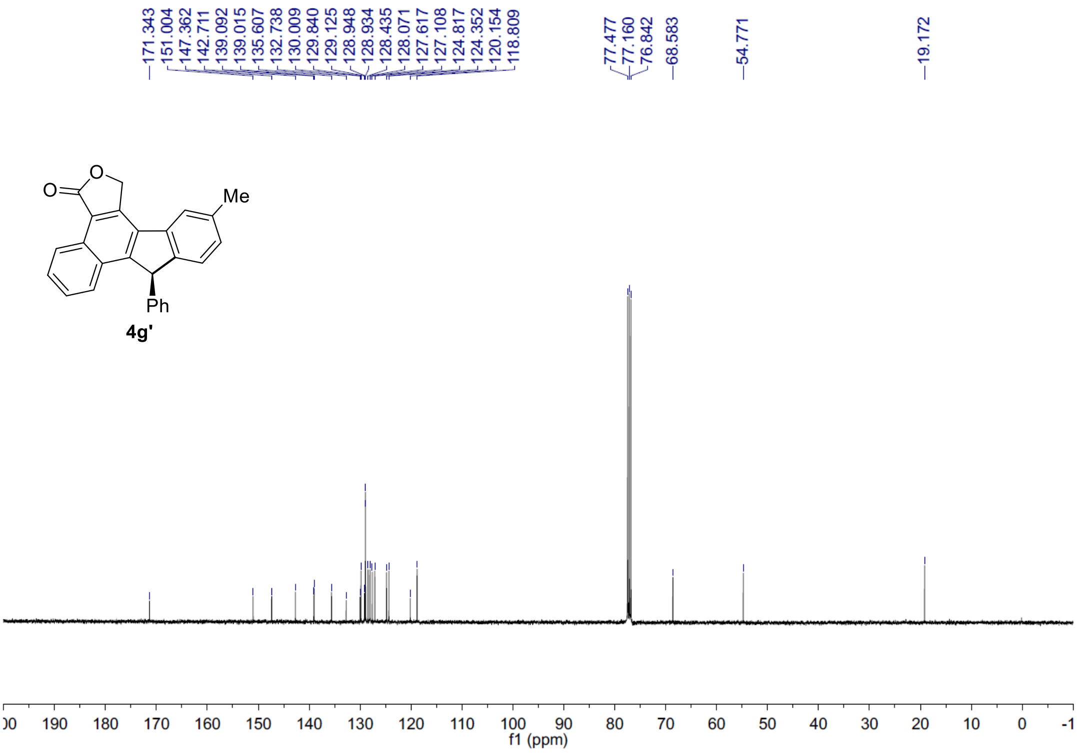

Supplementary Figure 41. ^{13}C **NMR (100 MHz, $CDCl_3$) spectrum for 4g'**

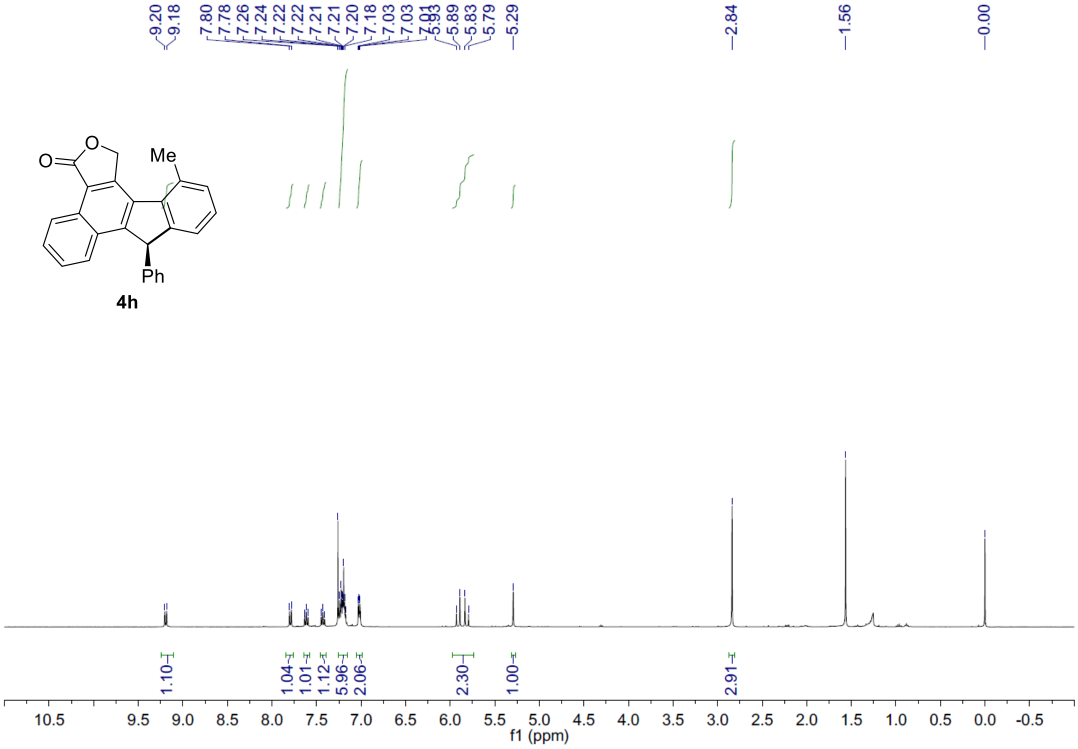

Supplementary Figure 42. ^{1}H NMR (400 MHz, $CDCl_3$) spectrum for 4h

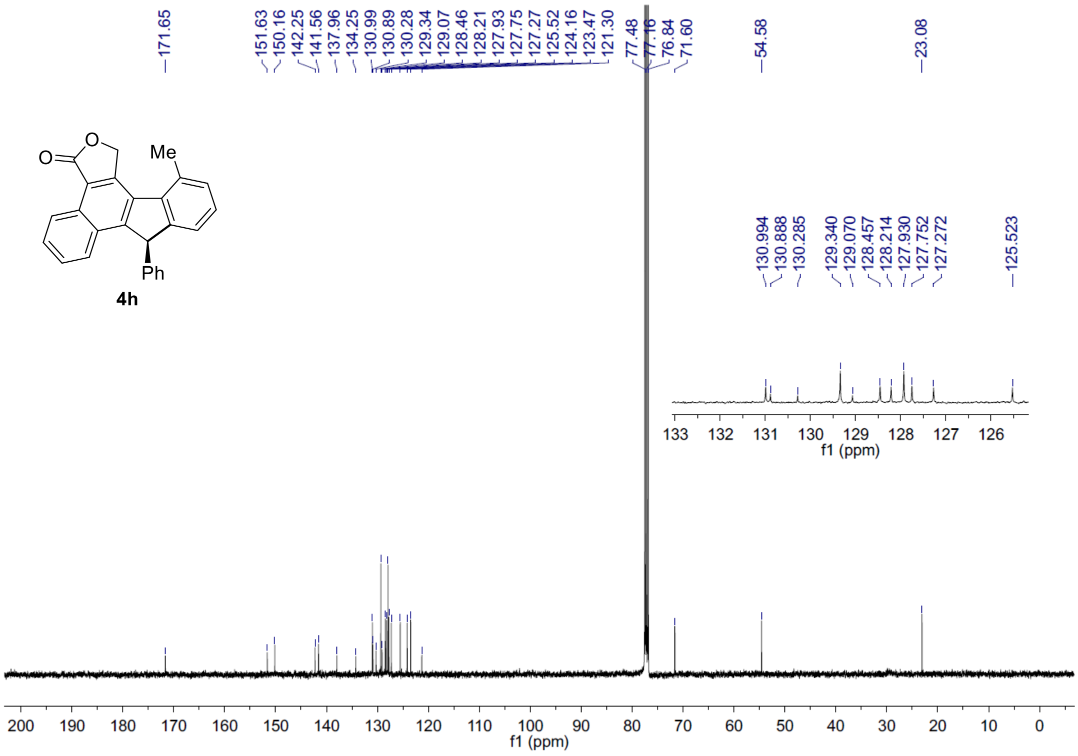

Supplementary Figure 43. ^{13}C NMR (100 MHz, $CDCl_3$) spectrum for 4h

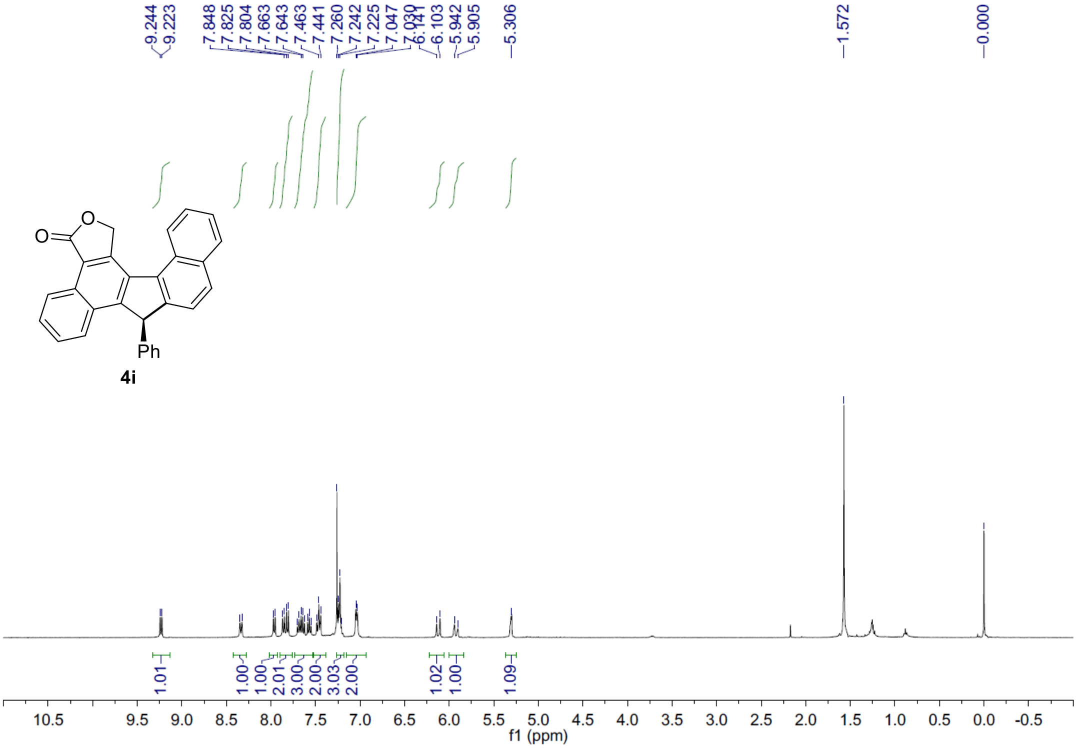

Supplementary Figure 44. ^{1}H NMR (400 MHz, $CDCl_3$) spectrum for 4i

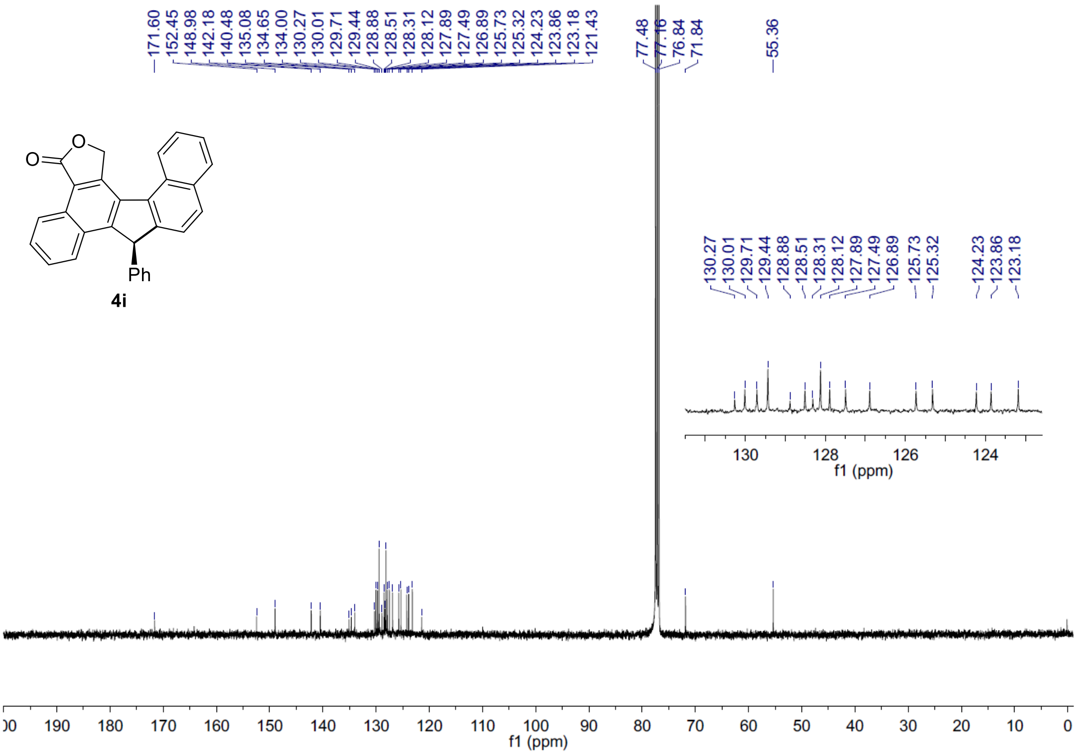

Supplementary Figure 45. ^{13}C NMR (100 MHz, $CDCl_3$) spectrum for 4i

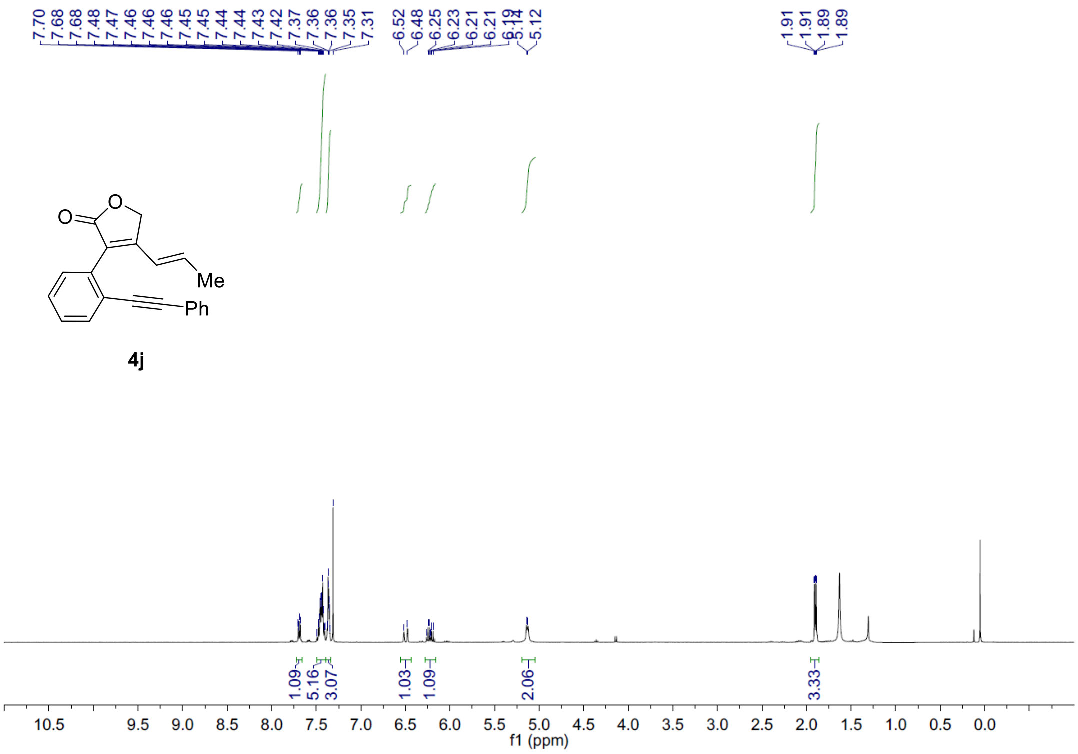

Supplementary Figure 46. ^{1}H NMR (400 MHz, $CDCl_3$) spectrum for 4j

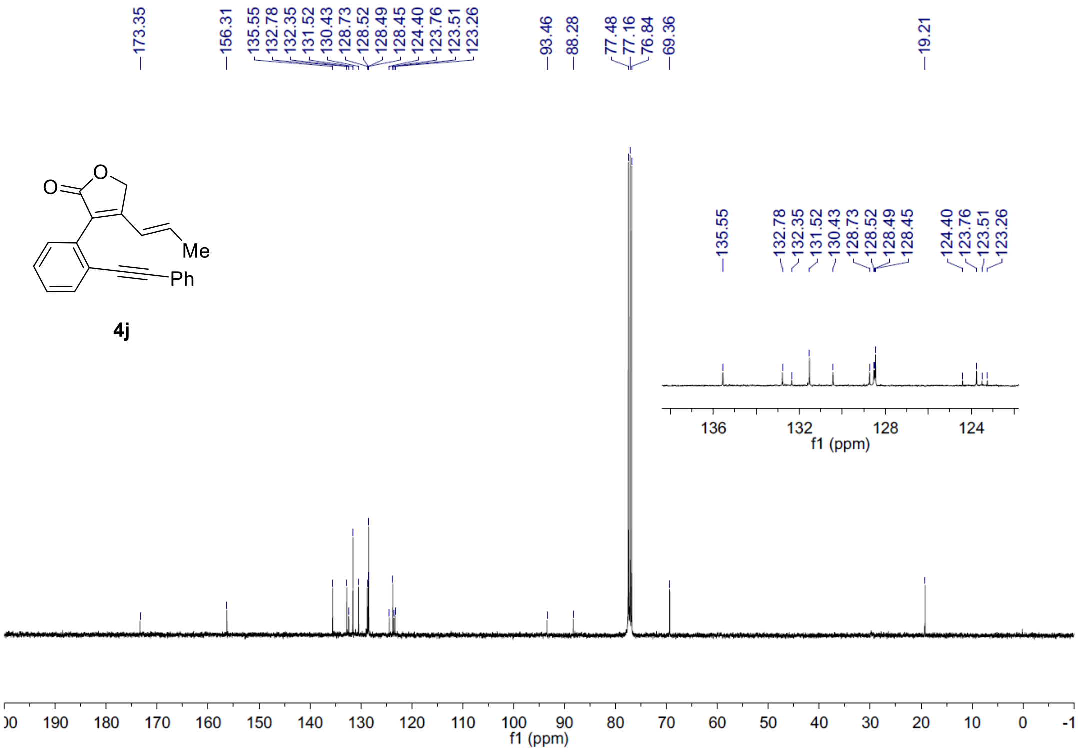

Supplementary Figure 47. ^{13}C NMR (100 MHz, $CDCl_3$) spectrum for 4j

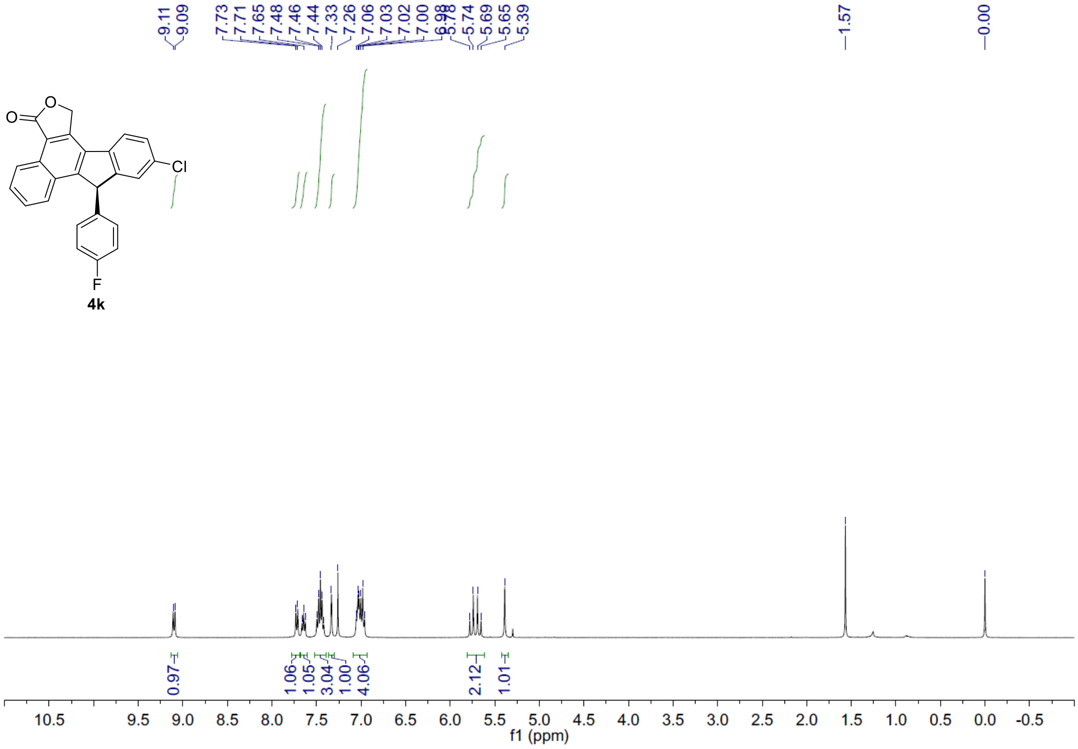

Supplementary Figure 48. ^{1}H NMR (400 MHz, $CDCl_3$) spectrum for 4k

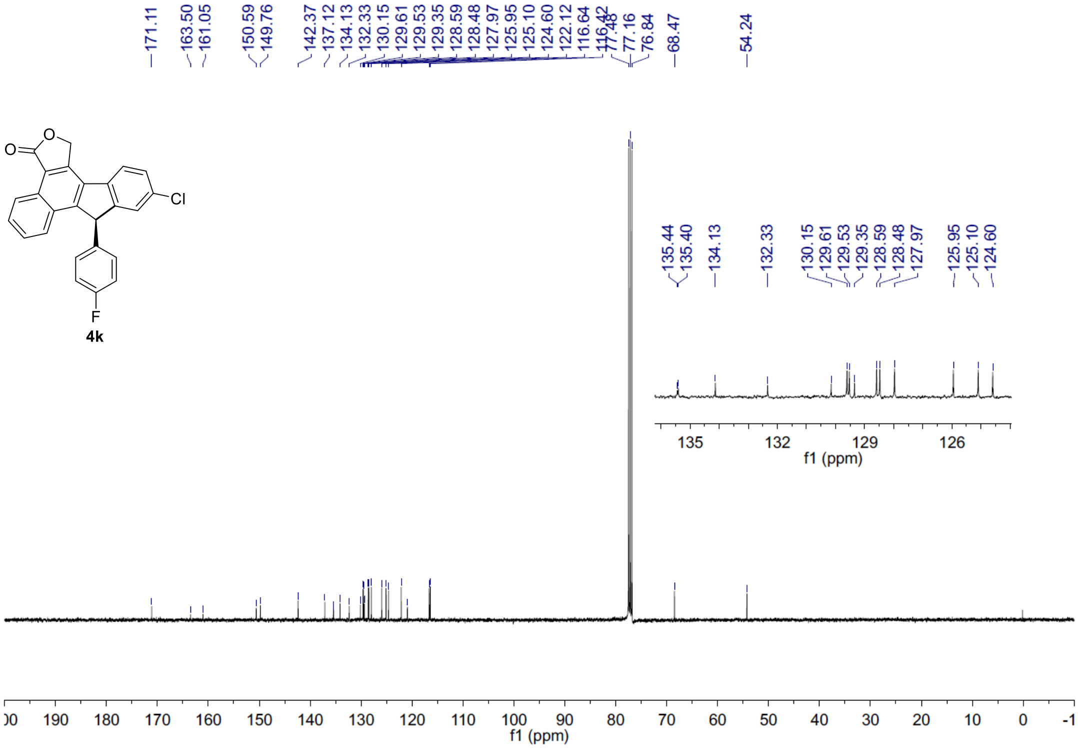

Supplementary Figure 49. ^{13}C NMR (100 MHz, $CDCl_3$) spectrum for 4k

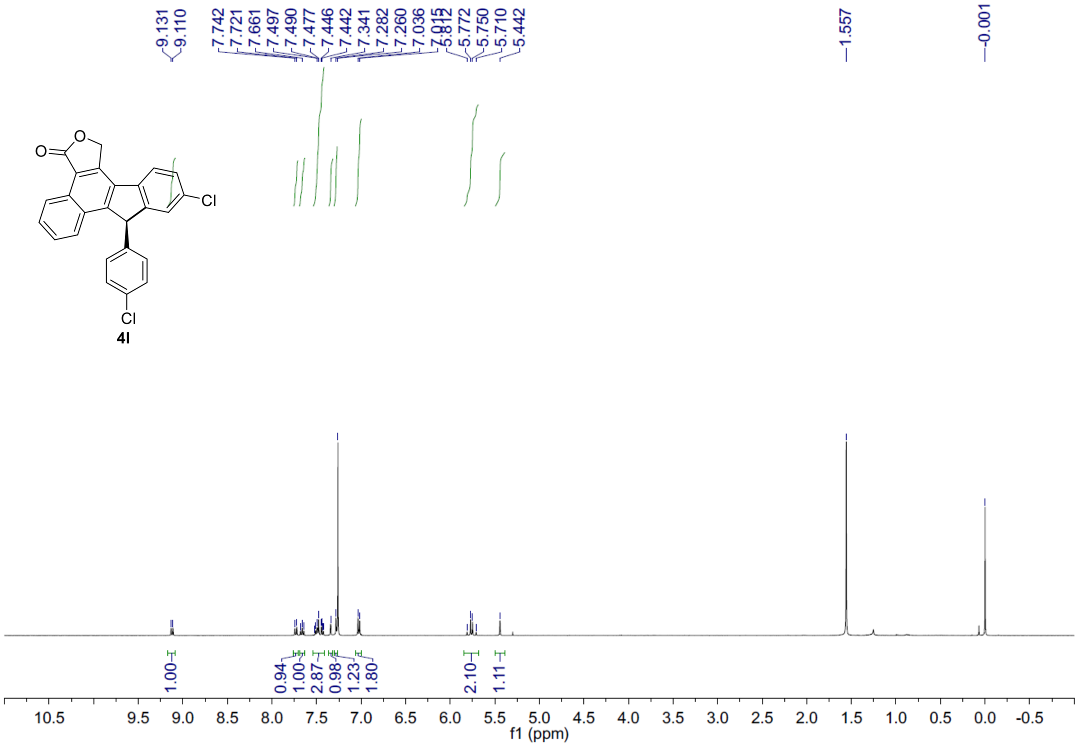

Supplementary Figure 50. ^{1}H NMR (600 MHz, $CDCl_3$) spectrum for 4l

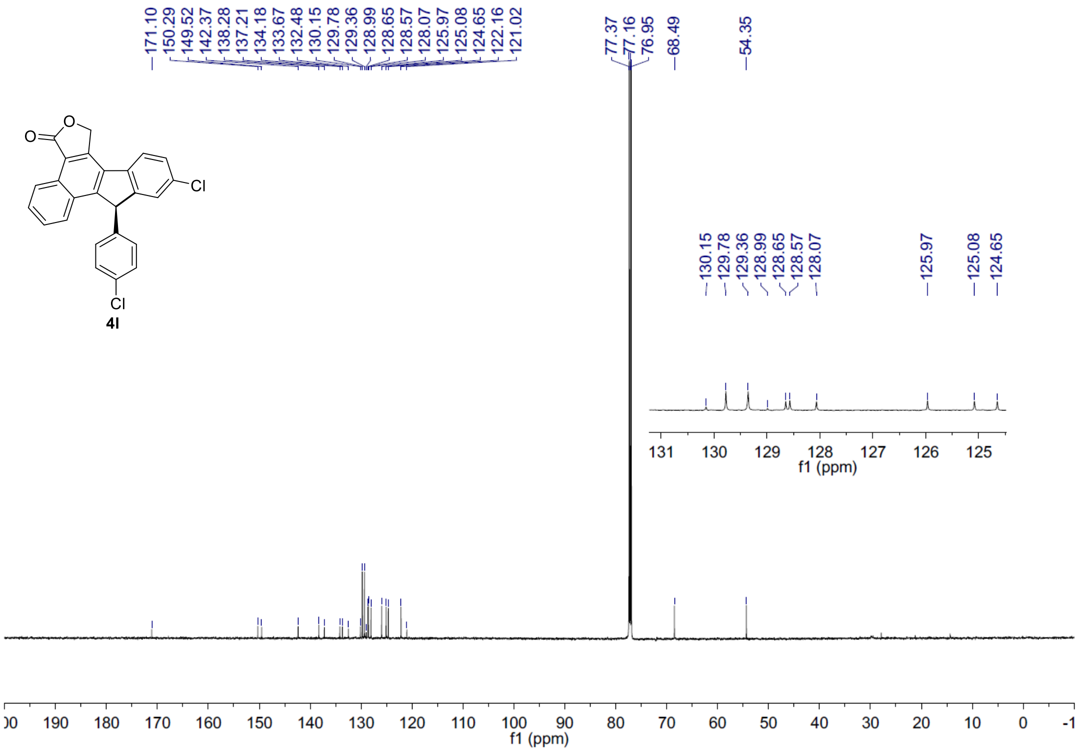

Supplementary Figure 51. ^{13}C NMR (150 MHz, $CDCl_3$) spectrum for 4l

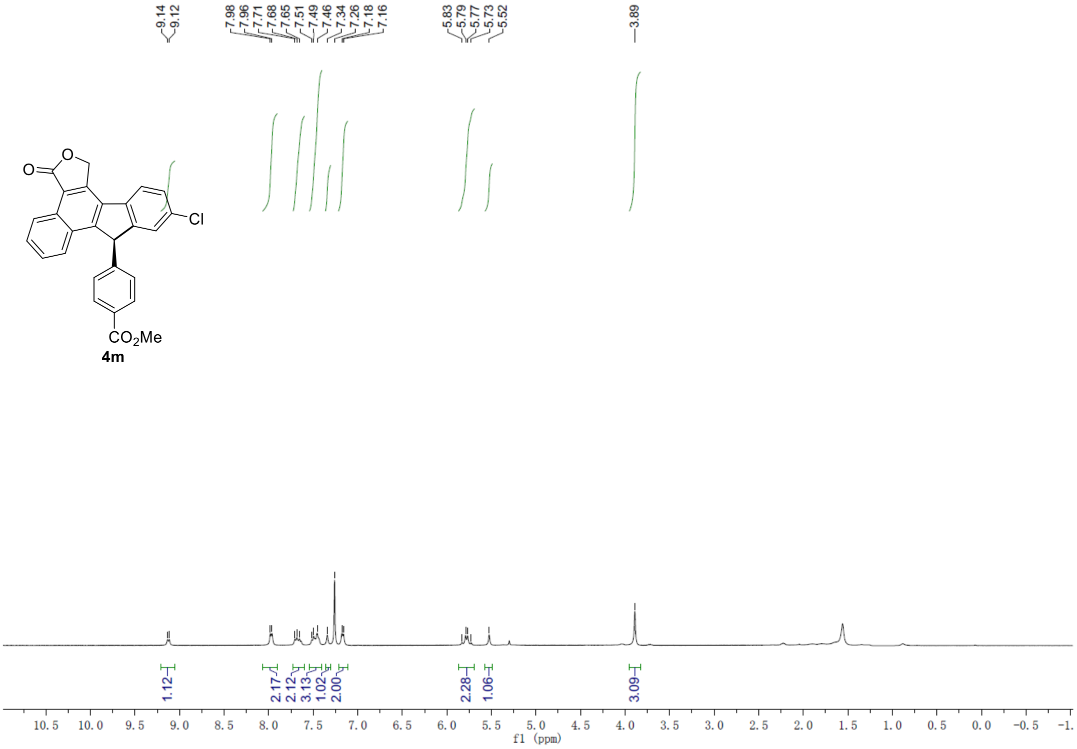

Supplementary Figure 52. ^{1}**H NMR (600 MHz, CDCl**$_3$**) spectrum for 4m**

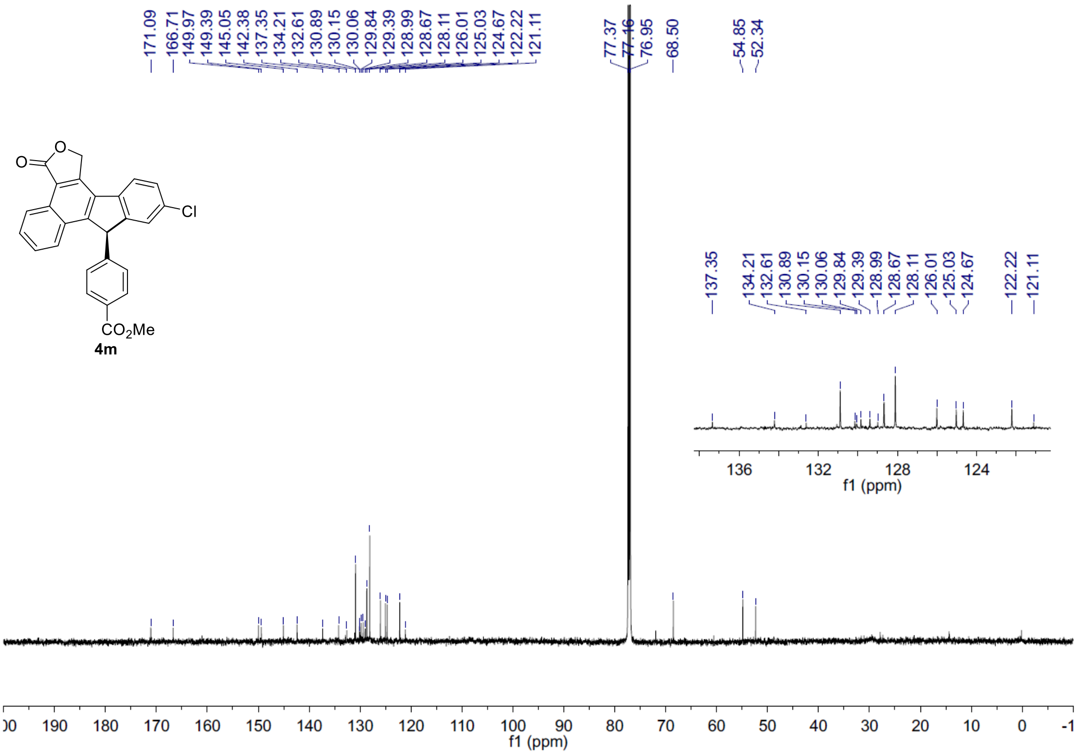

Supplementary Figure 53. ^{13}**C NMR (150 MHz, CDCl**$_3$**) spectrum for 4m**

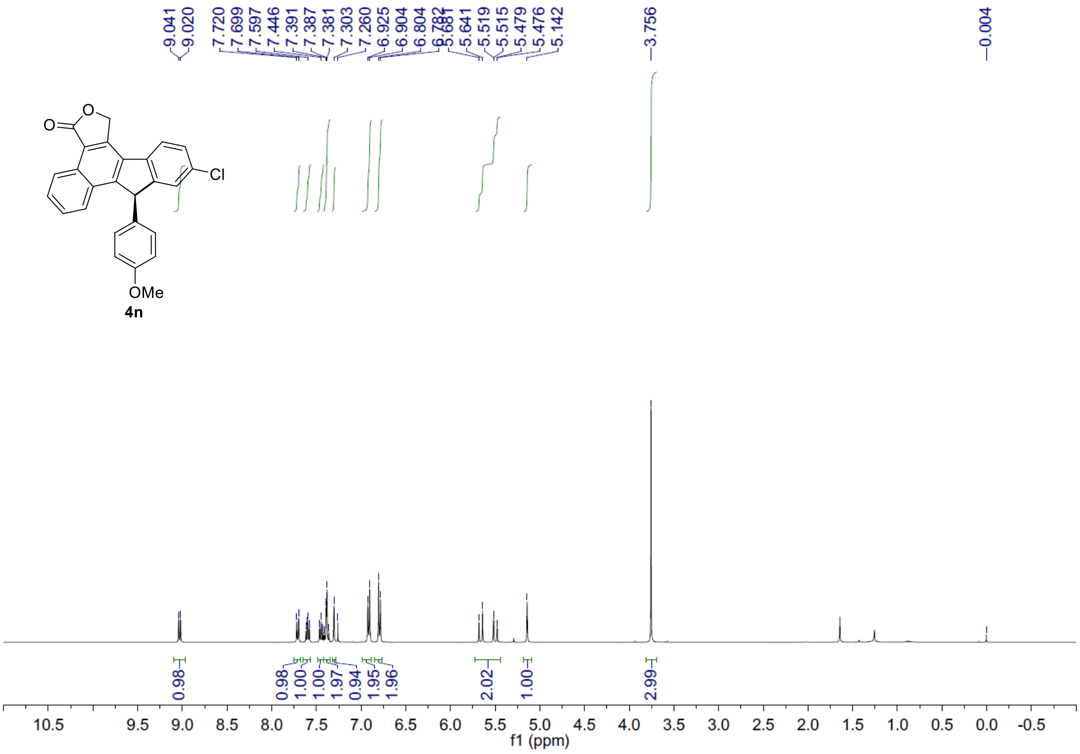

Supplementary Figure 54. ^{1}H NMR (400 MHz, $CDCl_3$) spectrum for 4n

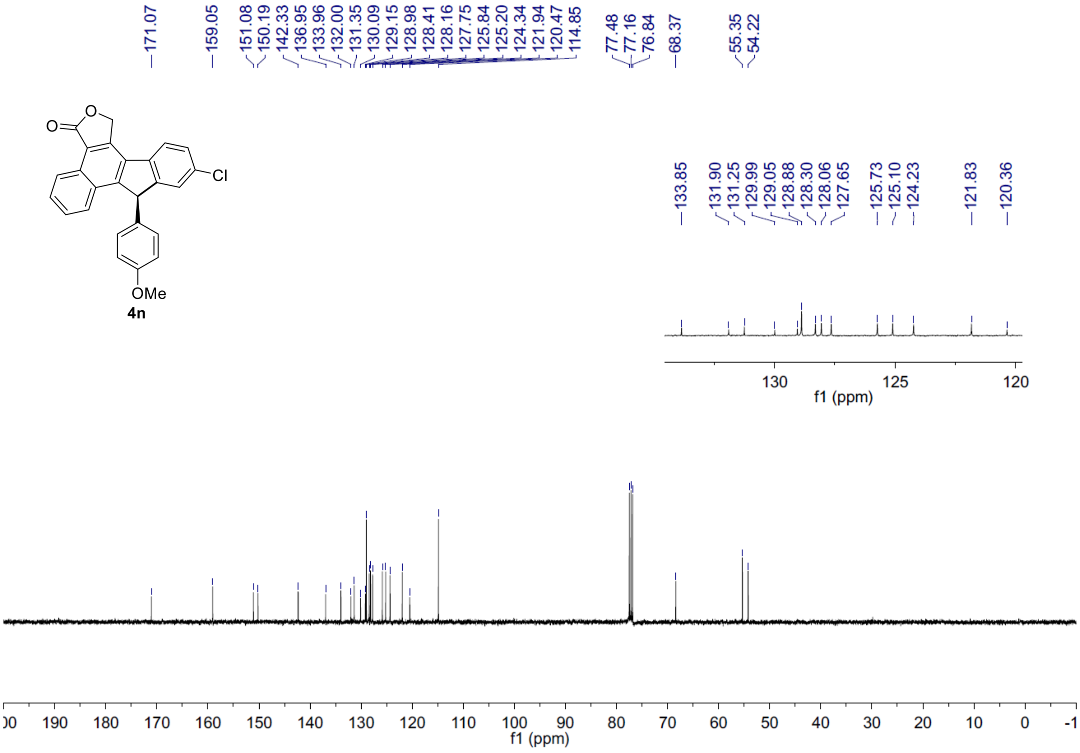

Supplementary Figure 55. ^{13}C NMR (100 MHz, $CDCl_3$) spectrum for 4n

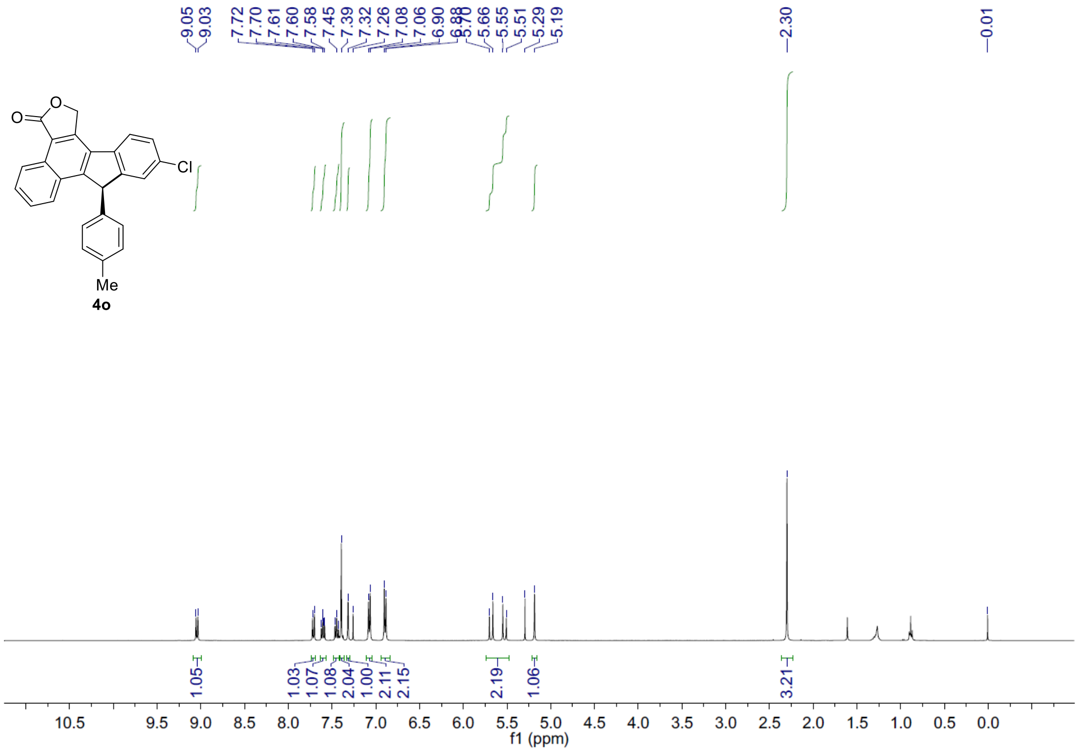

Supplementary Figure 56 ^{1}H NMR (400 MHz, $CDCl_3$) spectrum for 4o

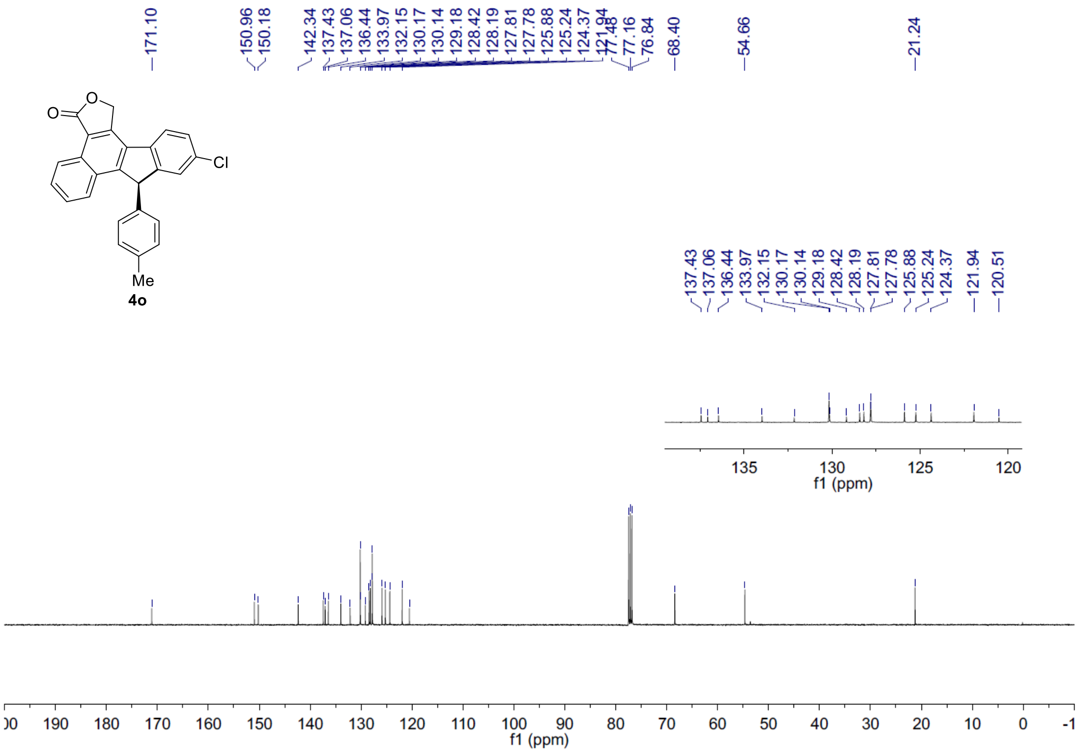

Supplementary Figure 57. ^{13}C NMR (100 MHz, $CDCl_3$) spectrum for 4o

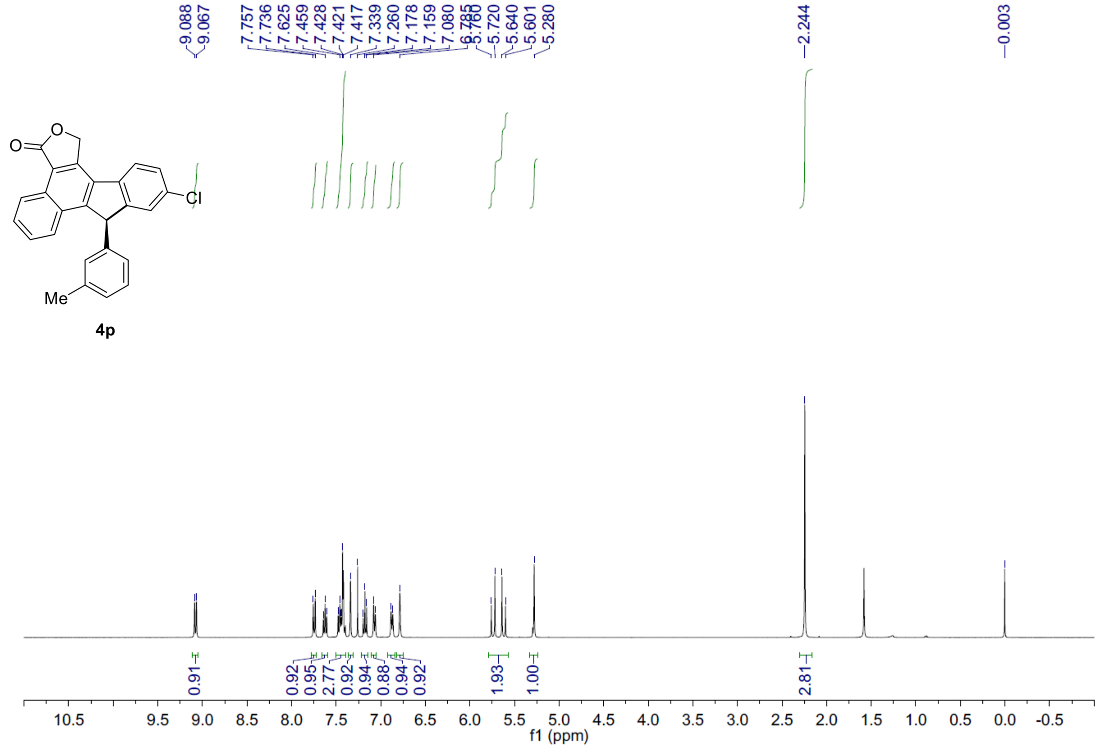

Supplementary Figure 58. ^{1}**H NMR (400 MHz,** $CDCl_3$**) spectrum for 4p**

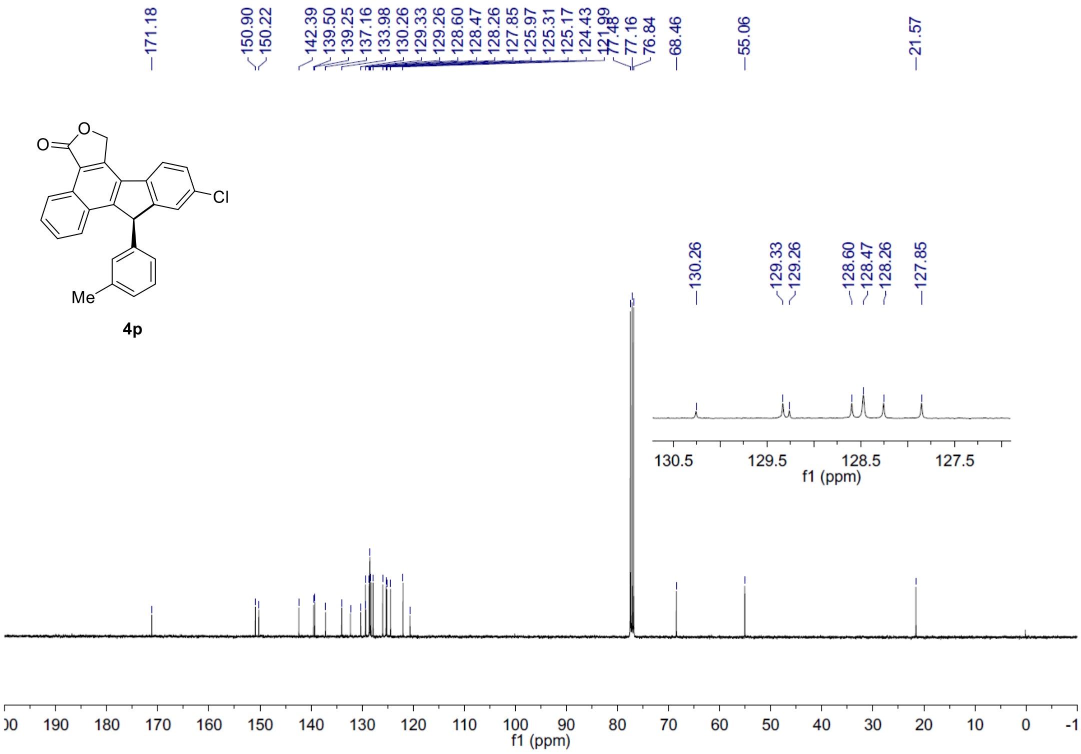

Supplementary Figure 59. ^{13}**C NMR (100 MHz,** $CDCl_3$**) spectrum for 4p**

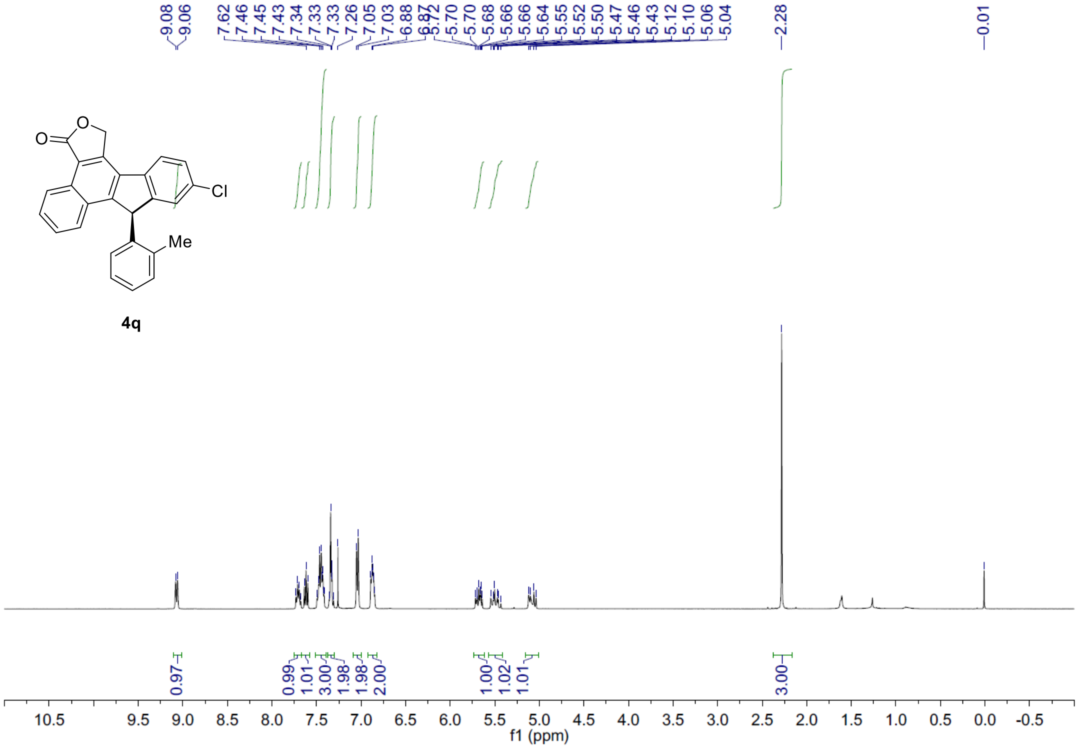

Supplementary Figure 60. ^{1}H NMR (400 MHz, $CDCl_3$) spectrum for 4q

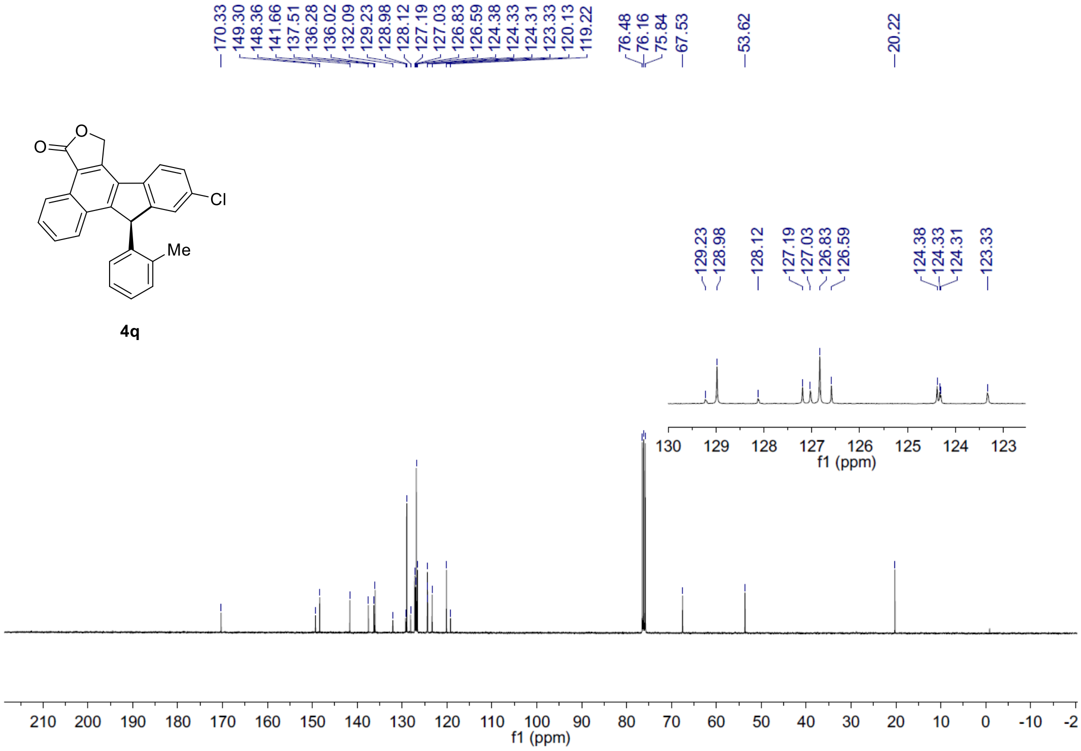

Supplementary Figure 61. ^{13}C NMR (100 MHz, $CDCl_3$) spectrum for 4q

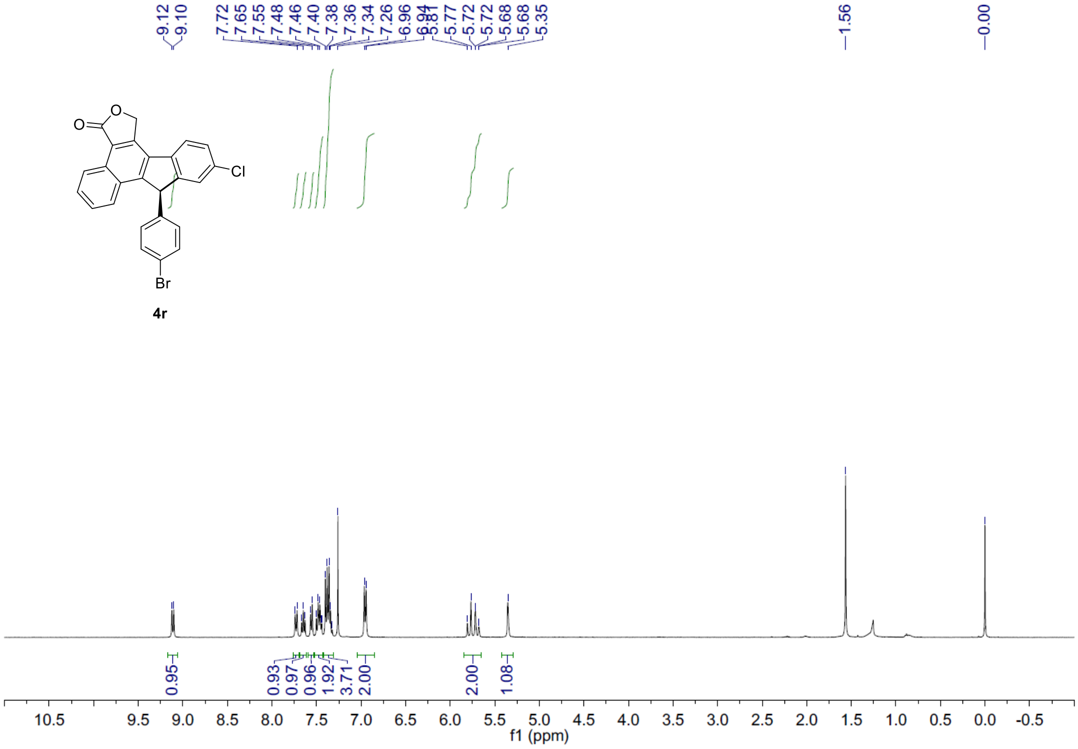

Supplementary Figure 62. ^{1}H NMR (400 MHz, $CDCl_3$) spectrum for 4r

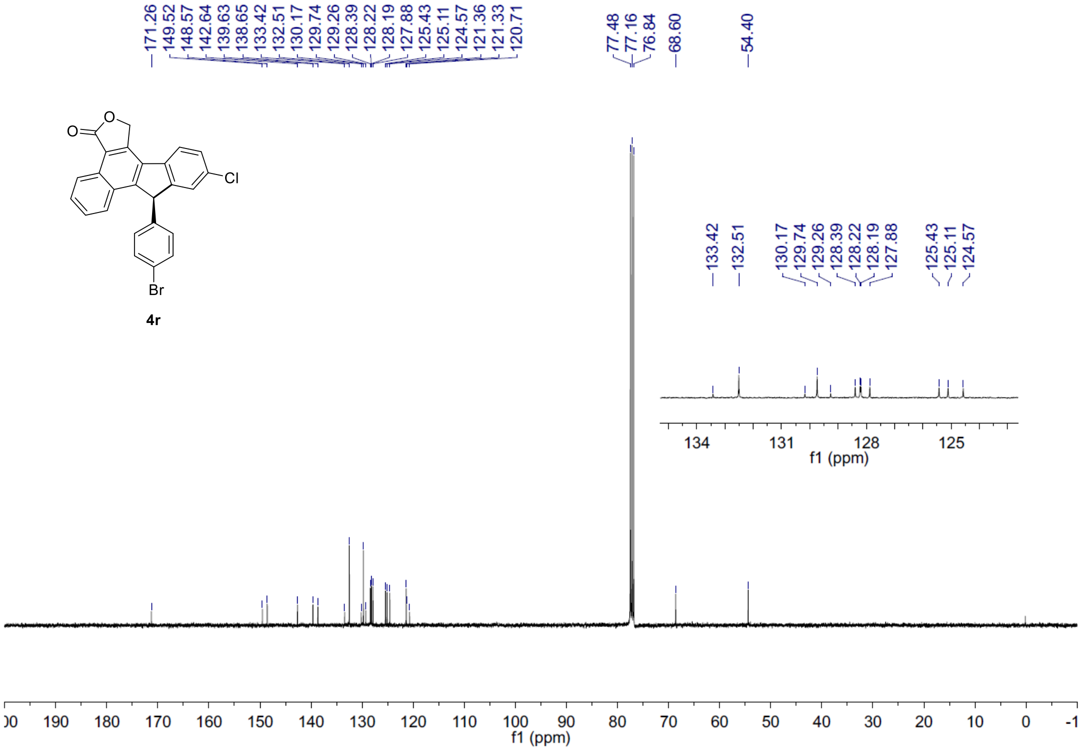

Supplementary Figure 63. ^{13}C NMR (100 MHz, $CDCl_3$) spectrum for 4r

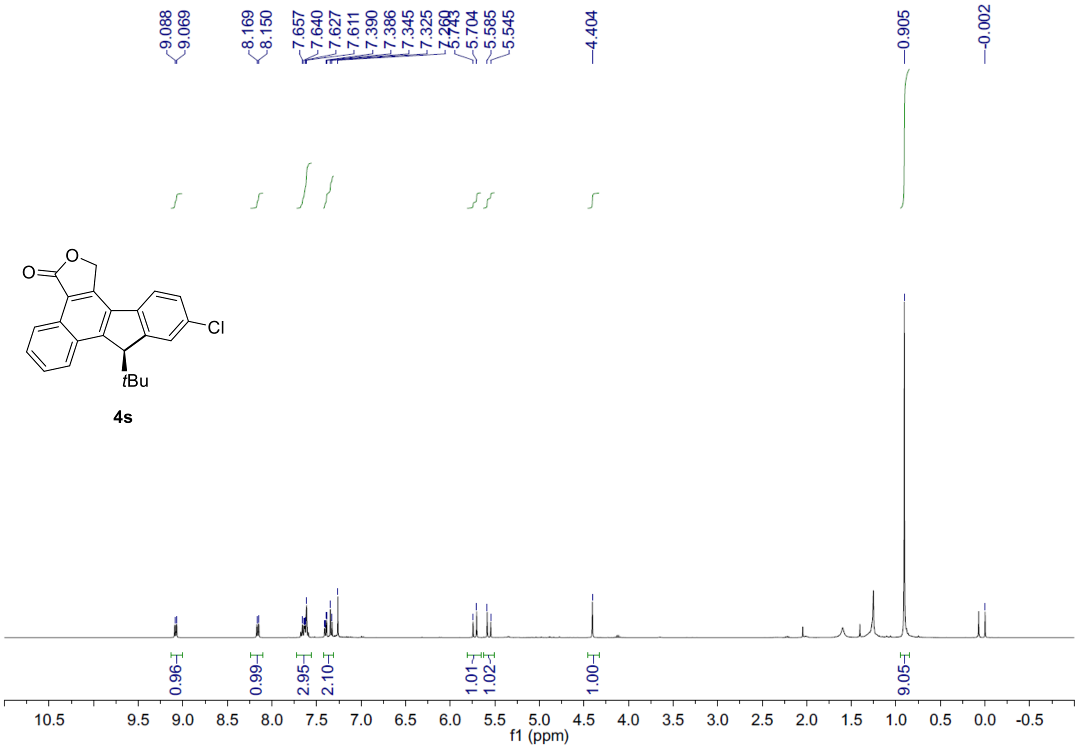

Supplementary Figure 64. **^{1}H NMR (600 MHz, $CDCl_3$) spectrum for 4s**

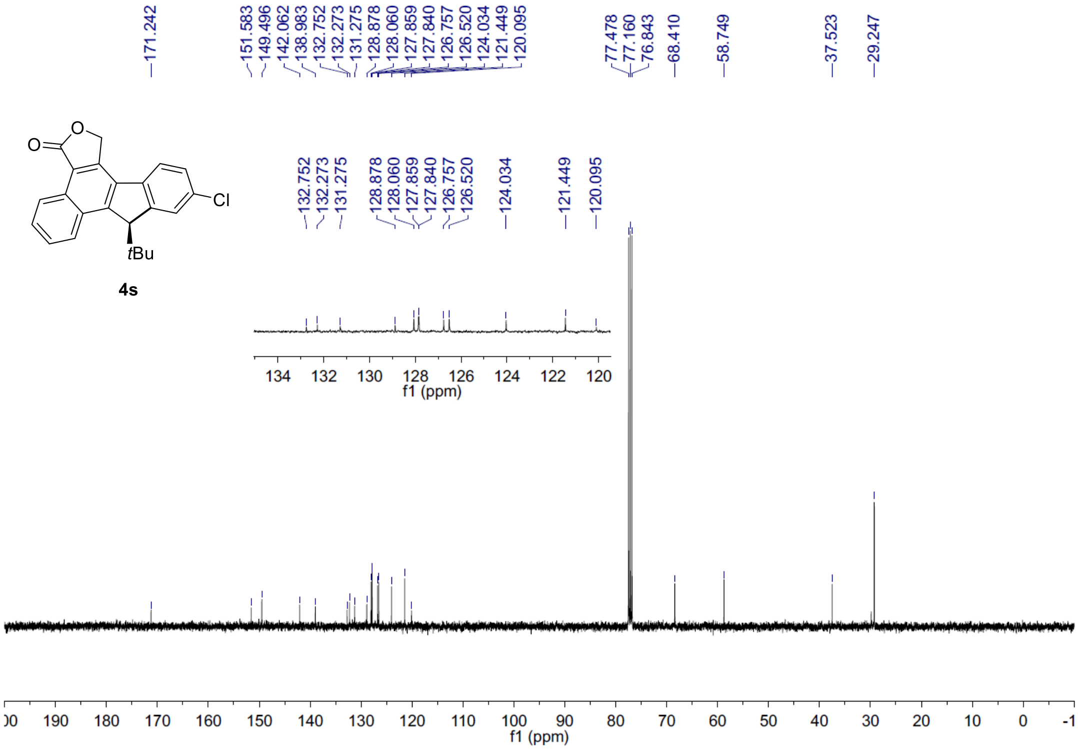

Supplementary Figure 65. **^{13}C NMR (150 MHz, $CDCl_3$) spectrum for 4s**

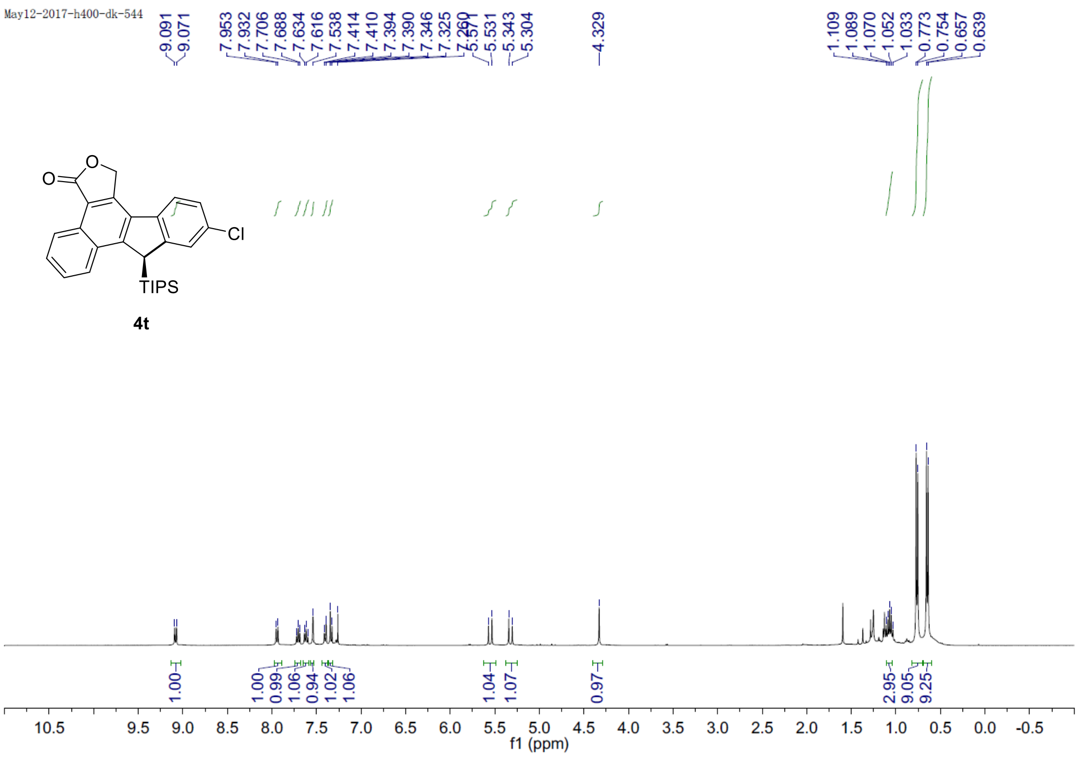

Supplementary Figure 66. ^{1}H NMR (400 MHz, $CDCl_3$) spectrum for 4t

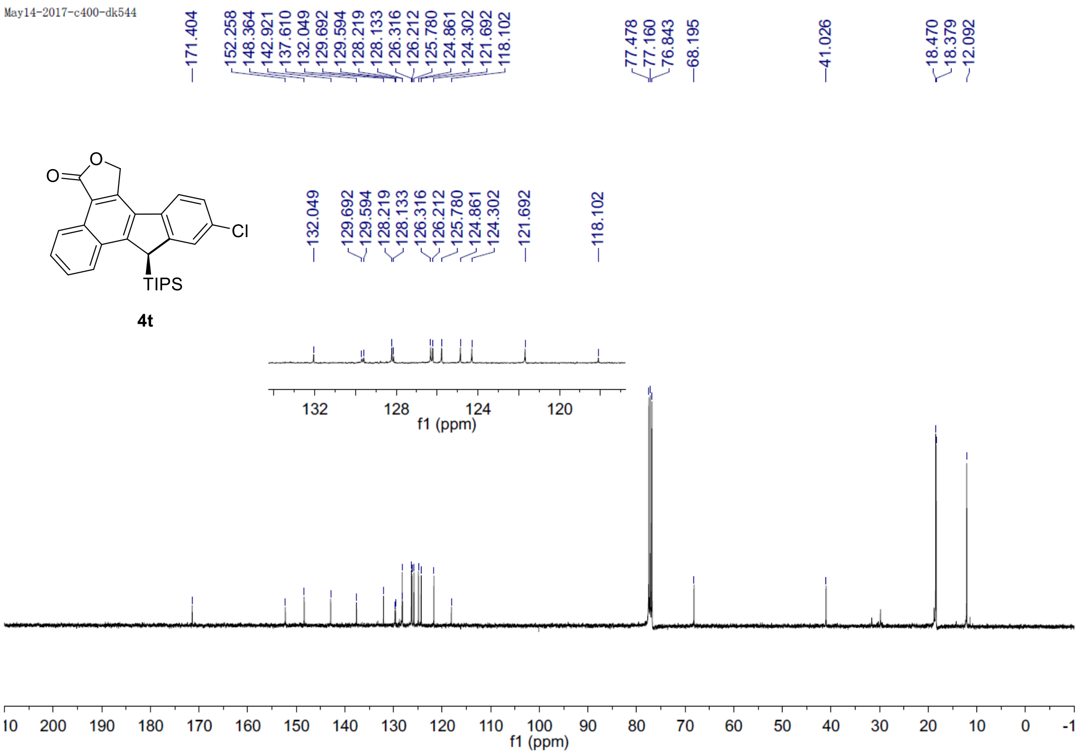

Supplementary Figure 67. ^{13}C NMR (100 MHz, $CDCl_3$) spectrum for 4t

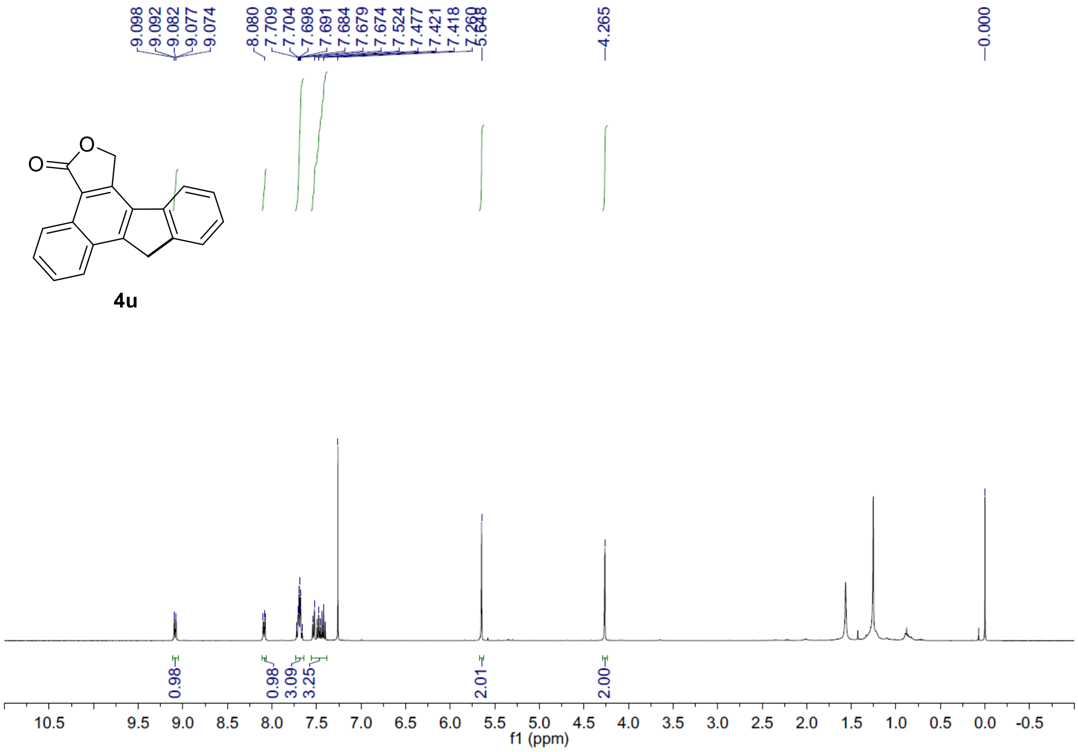

Supplementary Figure 68. ^{1}H NMR (400 MHz, $CDCl_3$) spectrum for 4u

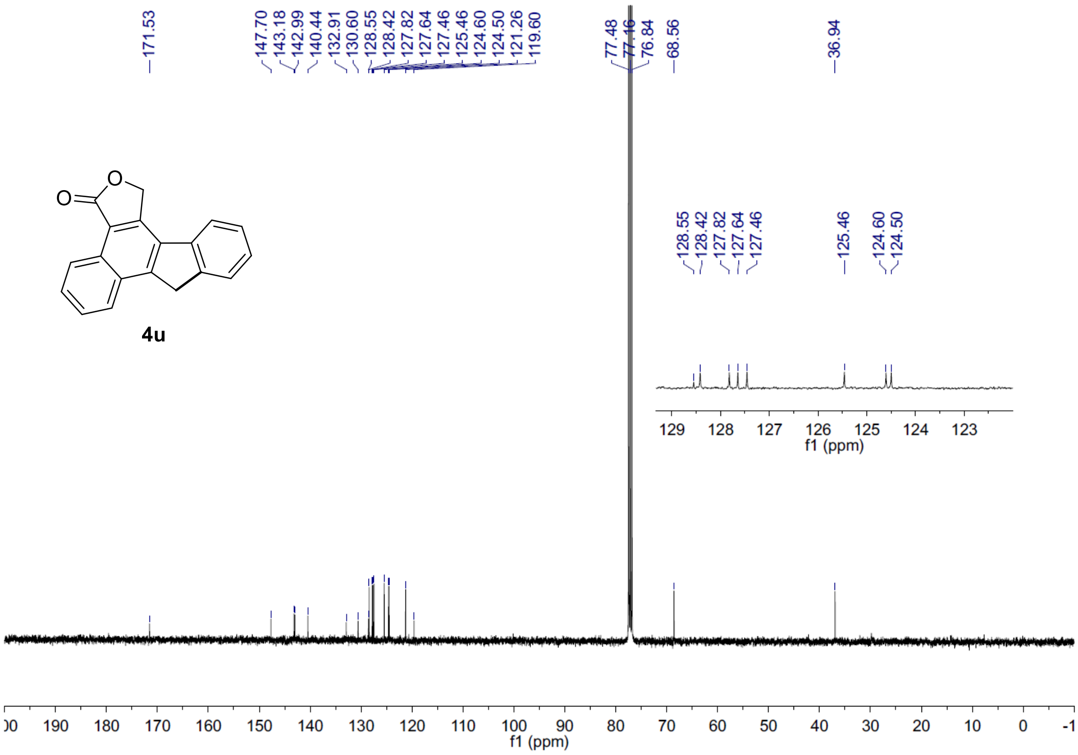

Supplementary Figure 69. ^{13}C NMR (100 MHz, $CDCl_3$) spectrum for 4u

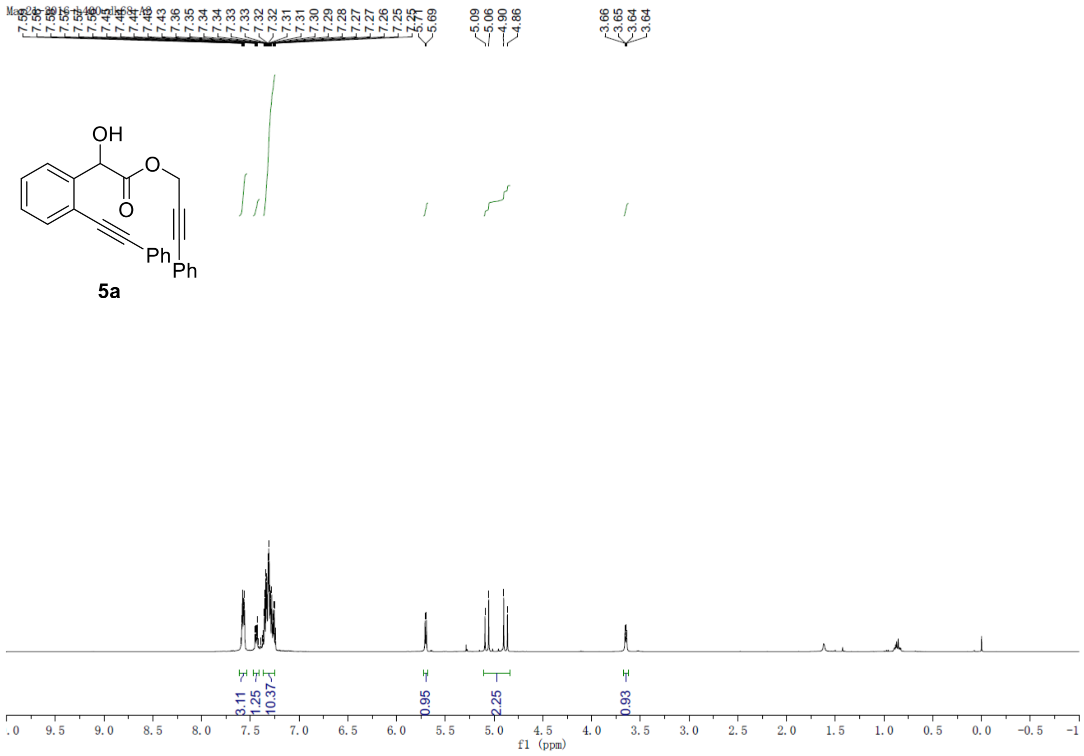

Supplementary Figure 70. **^{1}H NMR (400 MHz, $CDCl_3$) spectrum for 5a**

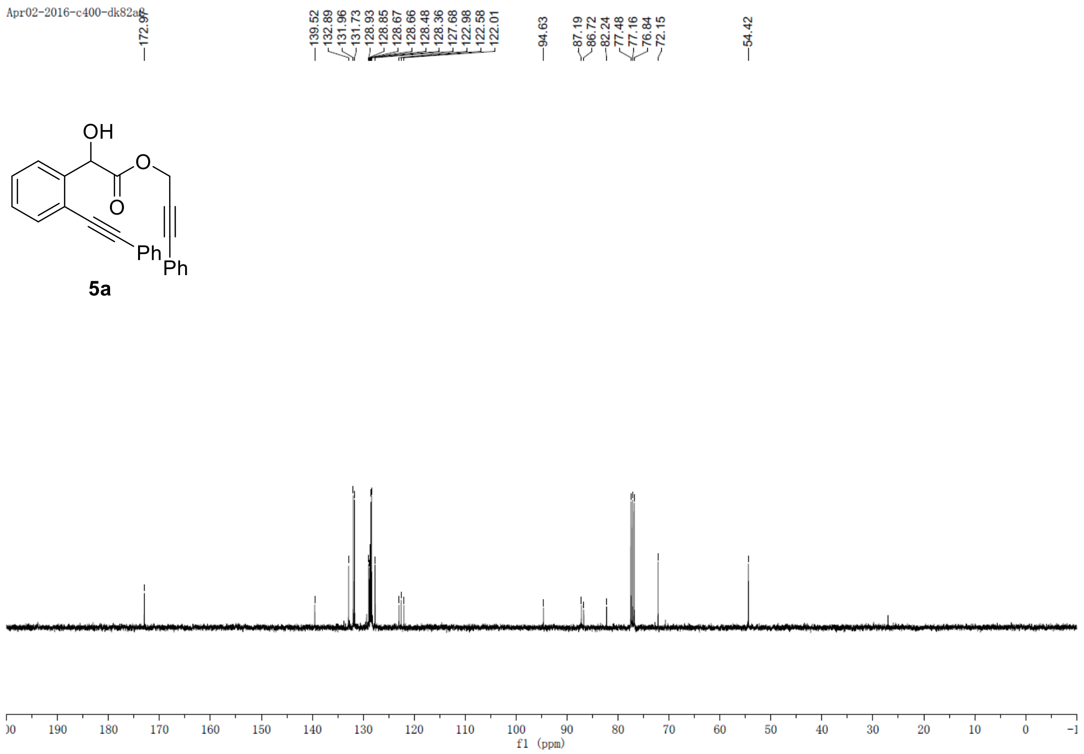

Supplementary Figure 71. **^{13}C NMR (100 MHz, $CDCl_3$) spectrum for 5a**

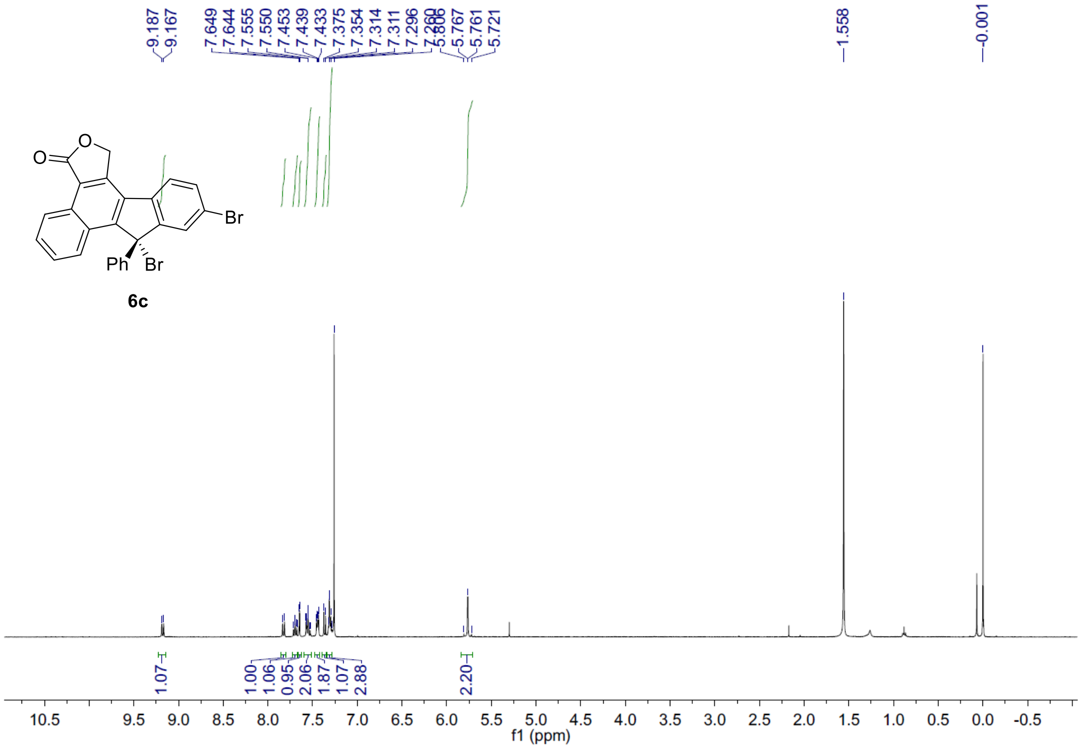

Supplementary Figure 72. **^{1}H NMR (400 MHz, $CDCl_3$) spectrum for 6c**

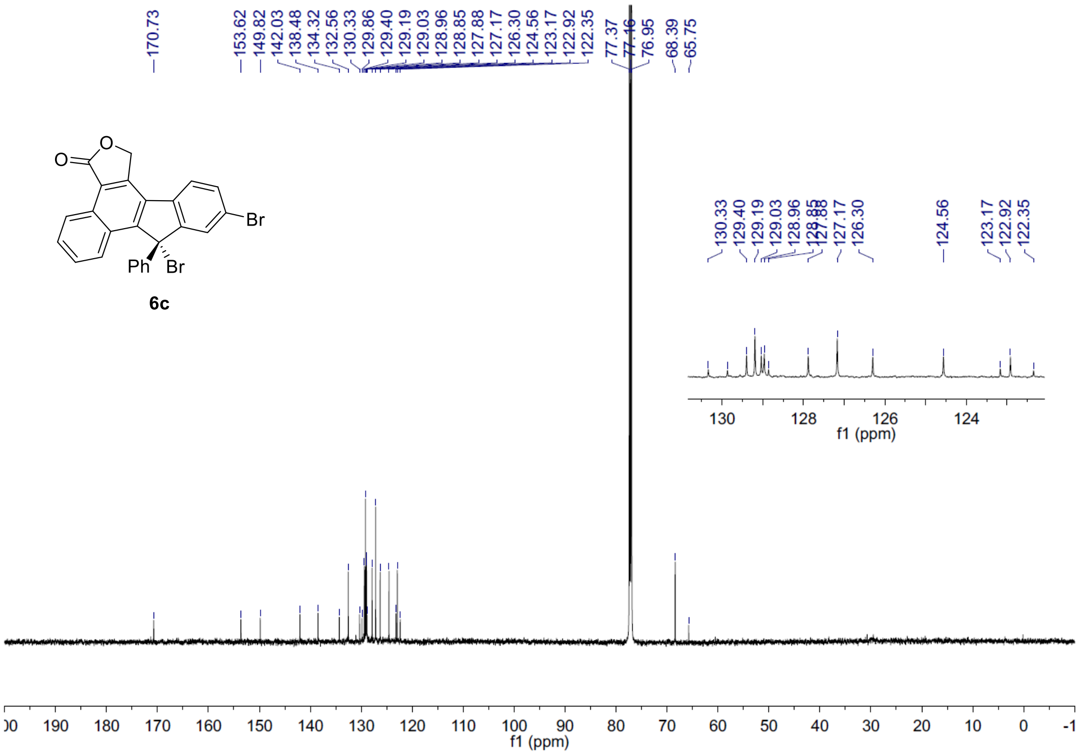

Supplementary Figure 73. **^{13}C NMR (100 MHz, $CDCl_3$) spectrum for 6c**

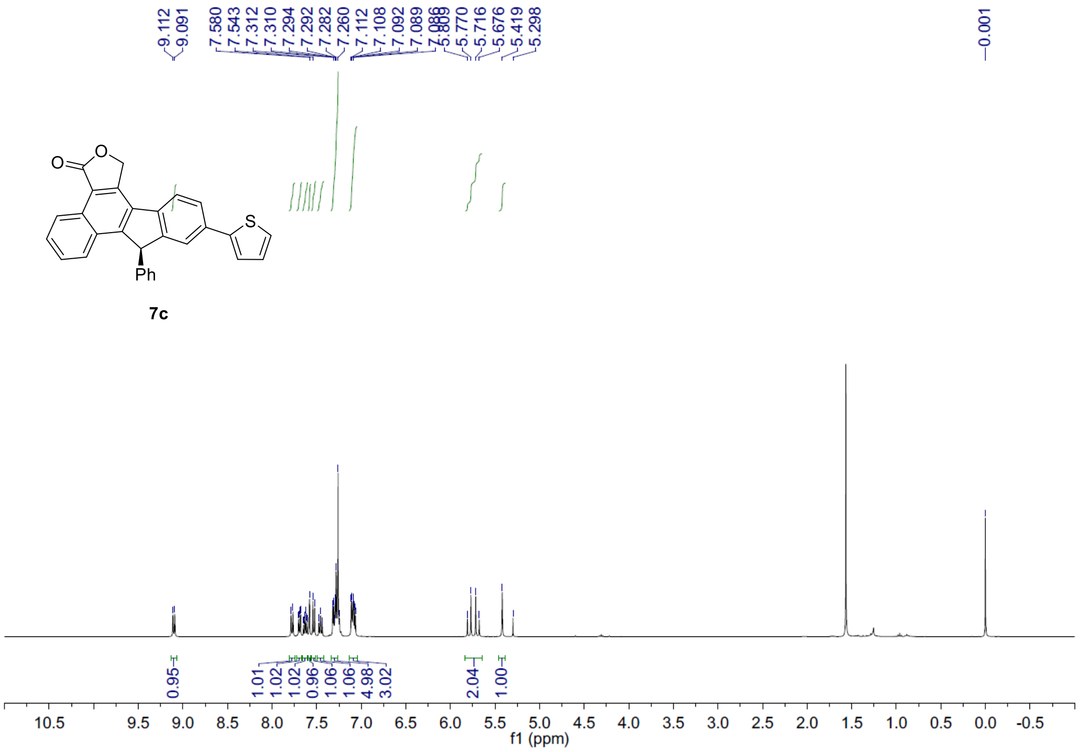

Supplementary Figure 74. ^{1}H **NMR (400 MHz,** $CDCl_3$**) spectrum for 7c**

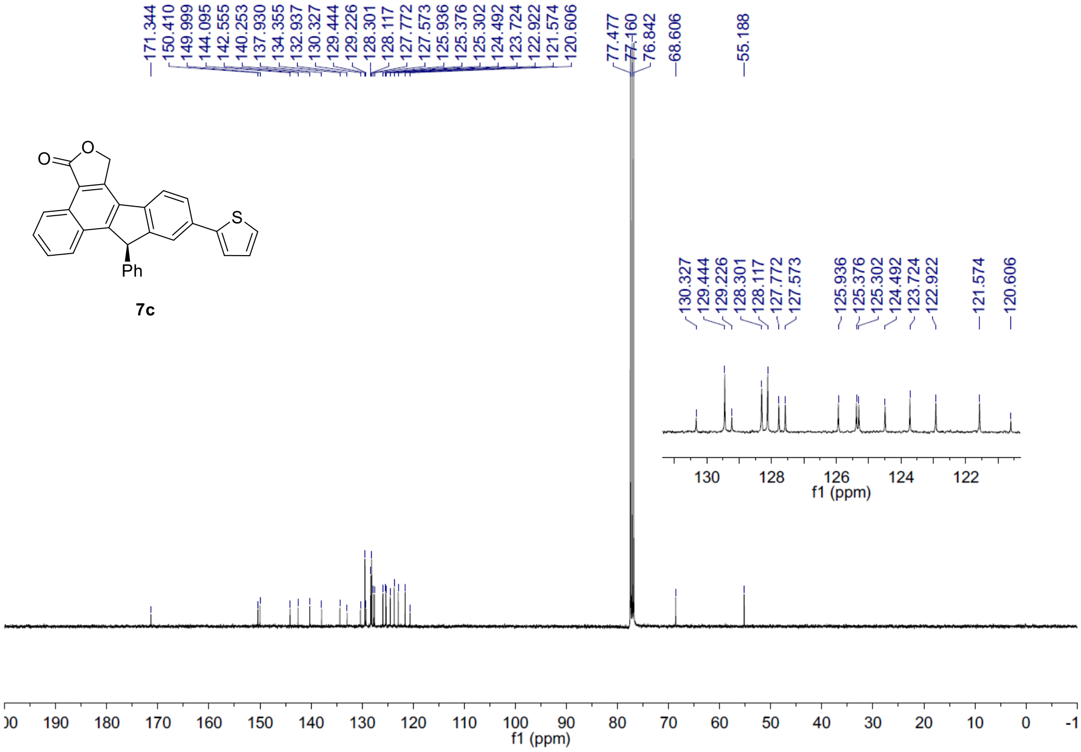

Supplementary Figure 75. ^{13}C **NMR (100 MHz,** $CDCl_3$**) spectrum for 7c**

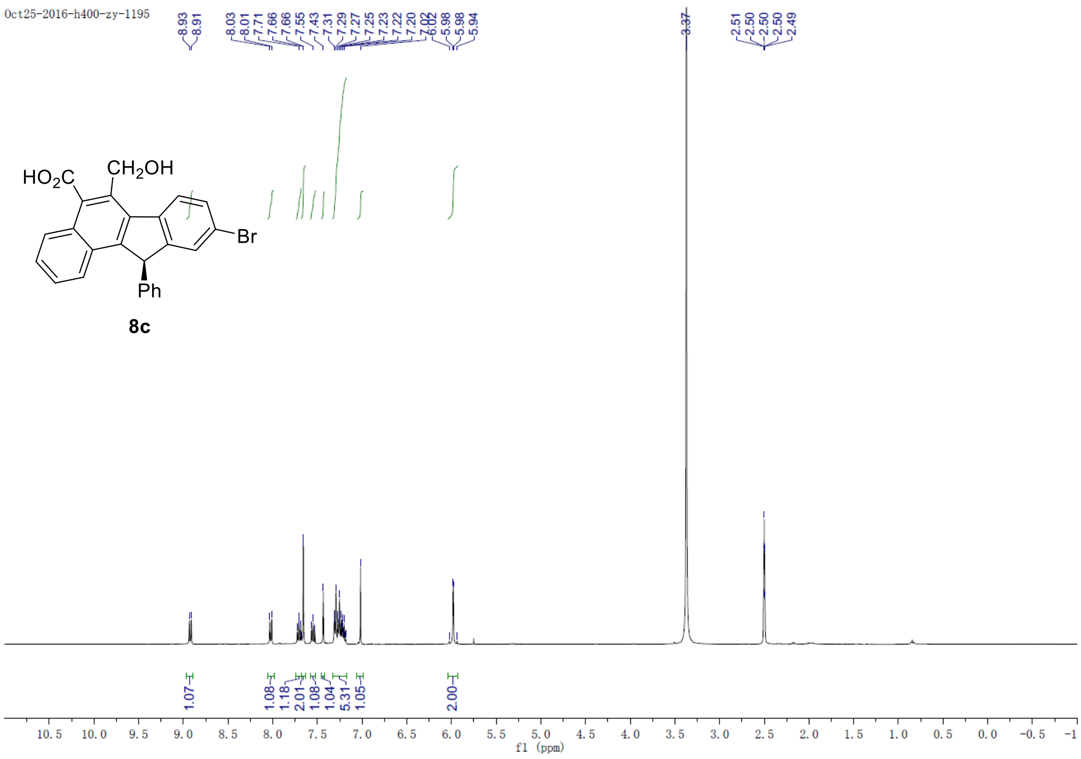

Supplementary Figure 76. ^{1}H NMR (400 MHz, $CDCl_3$) spectrum for 8c

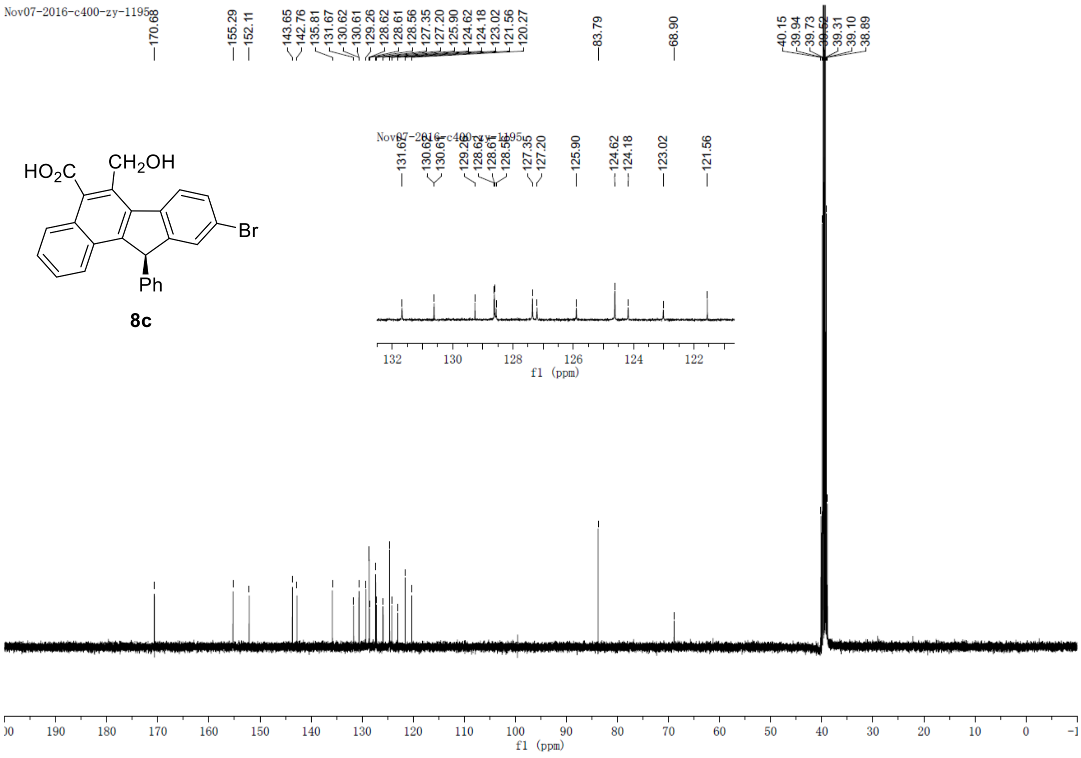

Supplementary Figure 77. ^{13}C NMR (100 MHz, $CDCl_3$) spectrum for 8c

HPLC Traces of Racemic and Chiral 2, 4.

Conditions: hexane/2-propanol/ethanol = 99.2:0.4:0.4

Flow rate = 1.0 mL/min, λ = 272 nm, Chiral IB-3

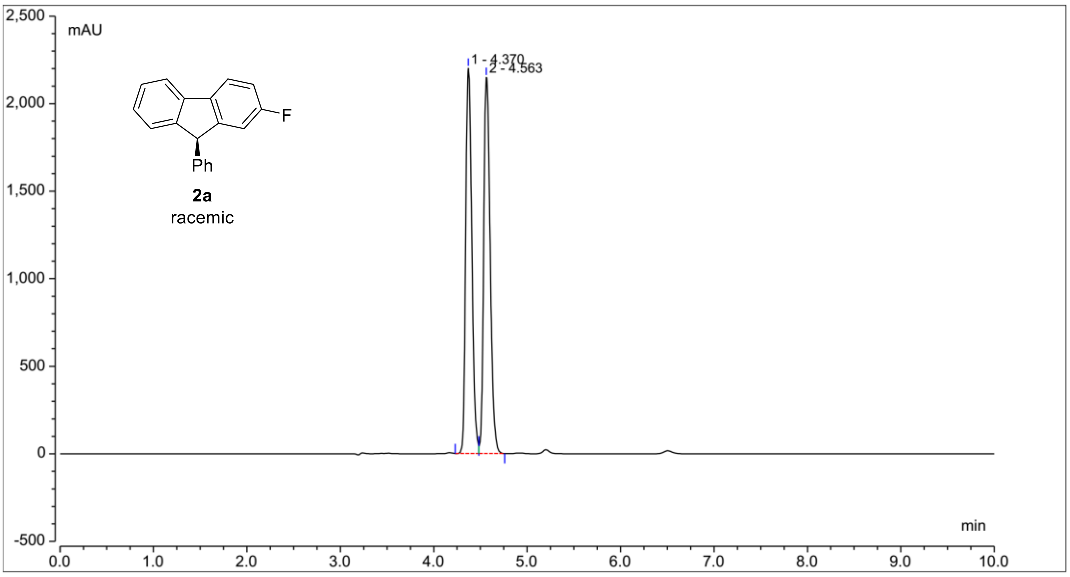

Entry	RT min	Area mAU*min	Height mAU	% Area %	% Height %
1	4.370	168.037	2200.228	49.38	50.59
2	4.563	172.259	2148.612	50.62	49.41

Supplementary Figure 78. HPLC trace for 2-Fluoro-9-phenyl-9*H*-fluorene (*rac*-2a)

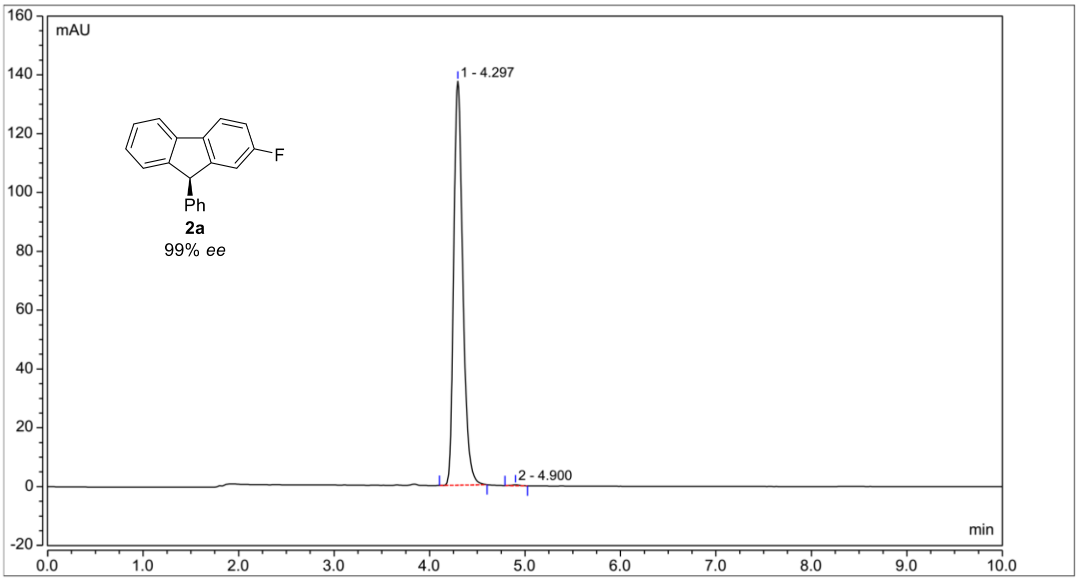

Entry	RT min	Area mAU*min	Height mAU	% Area %	% Height %
1	4.297	15.225	137.312	99.82	99.79
2	4.900	0.027	0.287	0.18	0.21

Supplementary Figure 79. HPLC trace for (*S*)-2-Fluoro-9-phenyl-9*H*-fluorene (2a)

Conditions: hexane/2-propanol/ethanol = 99.6:0.2:0.2

Flow rate = 1.0 mL/min, λ = 272 nm, Chiral IB-3

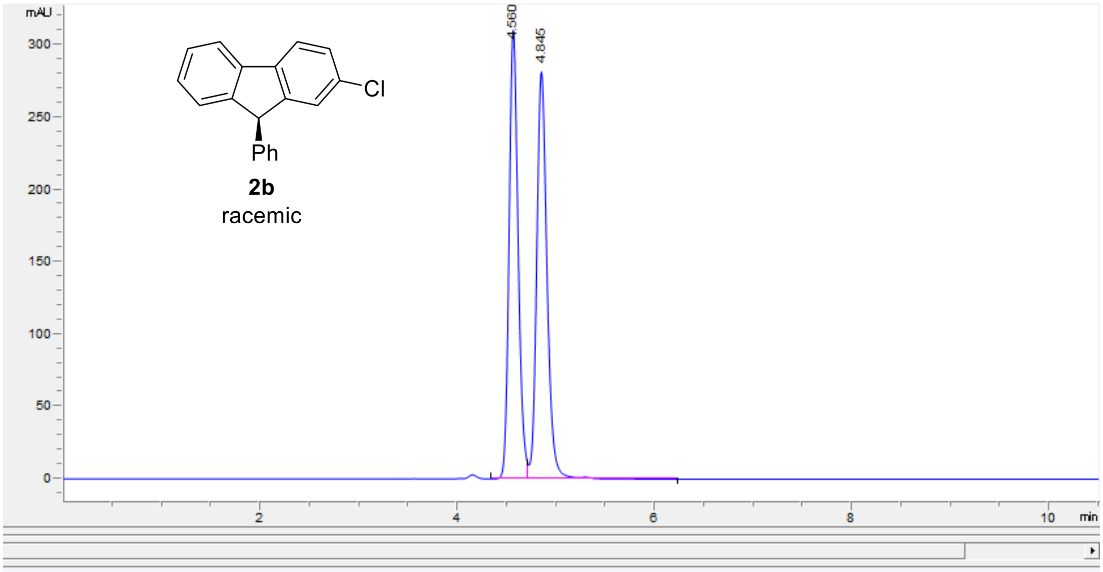

#	Time	Area	Height	Width	Area%	Symmetry
1	4.56	1999.4	311.9	0.0988	49.274	0.818
2	4.845	2058.3	283	0.1104	50.726	0.771

Supplementary Figure 80. HPLC trace for 2-Chloro -9-phenyl-9*H*-fluorene (*rac*-2b)

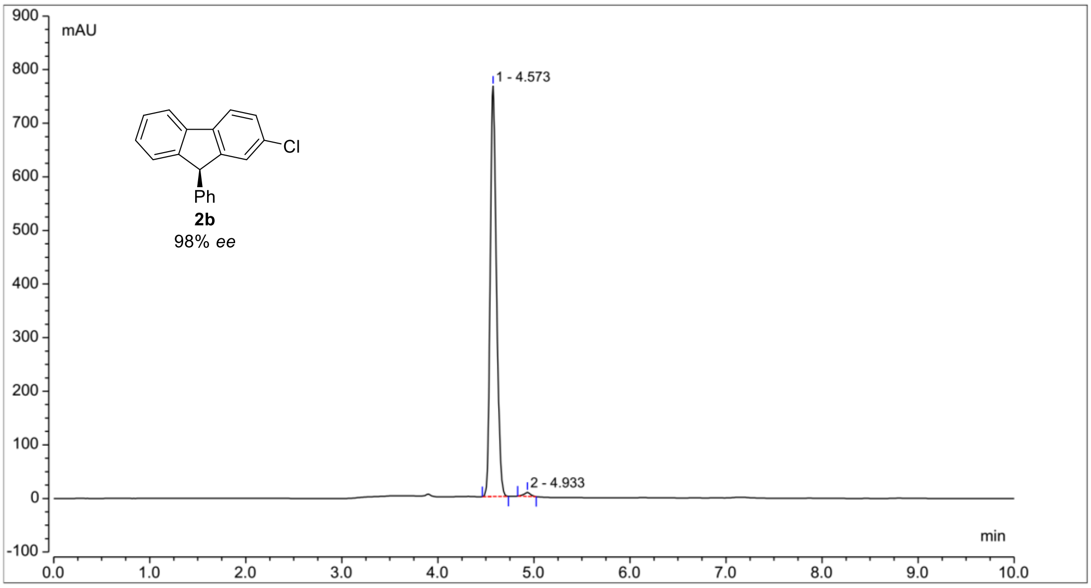

Entry	RT min	Area mAU*min	Height mAU	% Area %	% Height %
1	4.573	57.248	765.635	98.99	99.00
2	4.933	0.585	7.716	1.01	1.00

Supplementary Figure 81. HPLC trace for 2-Chloro -9-phenyl-9*H*-fluorene (2b)

Conditions: hexane/2-propanol/ethanol = 99.2:0.4:0.4

Flow rate = 1.0 mL/min, λ = 272 nm, Chiral IB-3

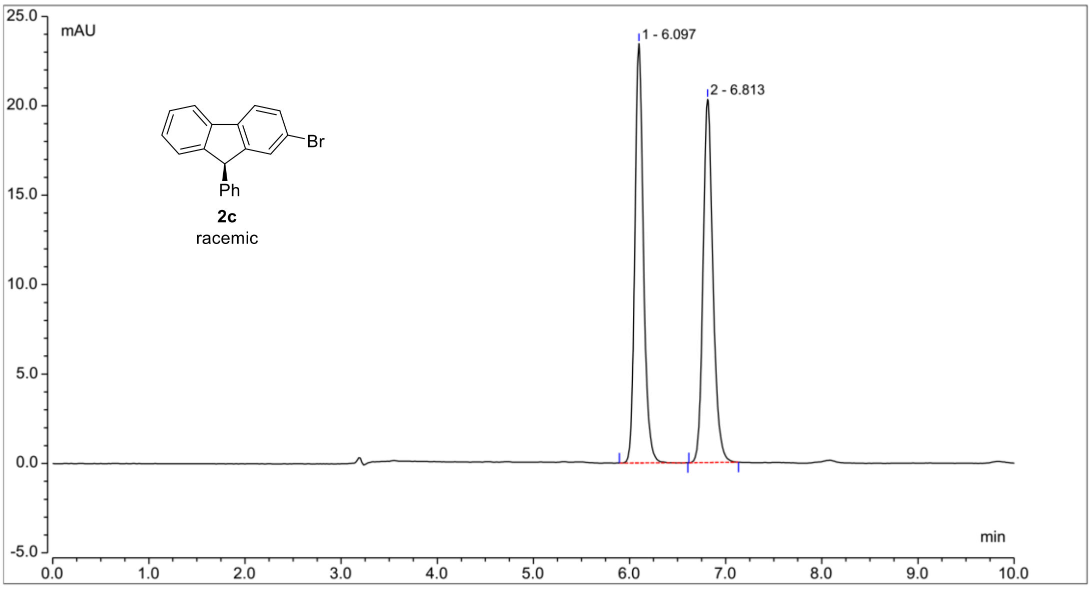

Entry	RT min	Area mAU*min	Height mAU	% Area %	% Height %
1	6.097	2.417	23.452	50.05	53.59
2	6.813	2.412	20.311	49.95	46.41

Supplementary Figure 82. HPLC trace for 2-Bromo -9-phenyl-9*H*-fluorene (*rac*-2c)

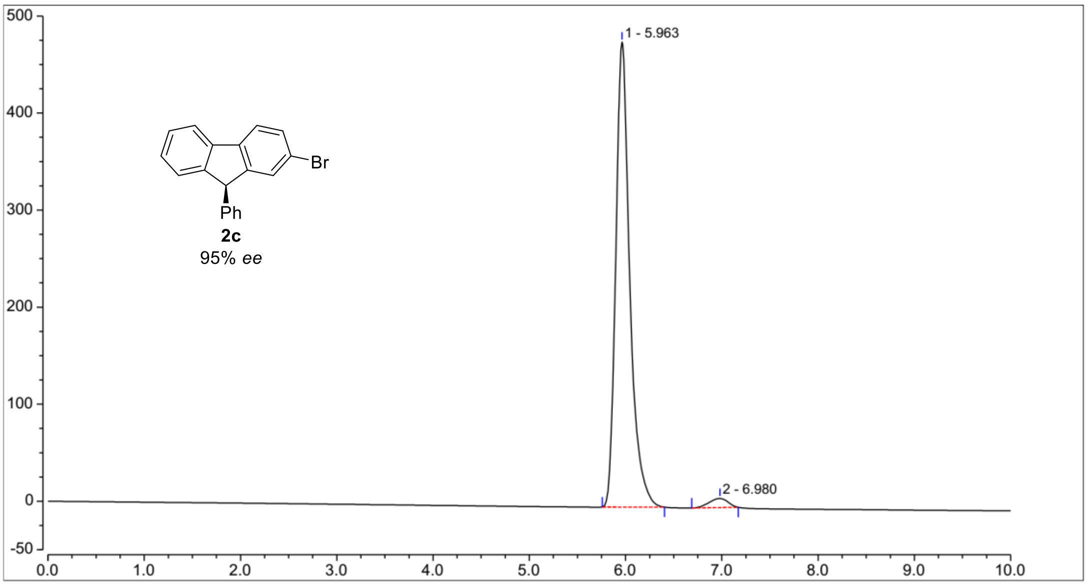

Entry	RT min	Area mAU*min	Height mAU	% Area %	% Height %
1	5.963	80.494	479.149	97.45	98.08
2	6.980	2.108	9.375	2.55	1.92

Supplementary Figure 83. HPLC trace for 2-Bromo -9-phenyl-9*H*-fluorene (2c)

Conditions: hexane/2-propanol/ethanol = 99.2:0.4:0.4

Flow rate = 1.0 mL/min, λ = 272 nm, Chiral IB-3

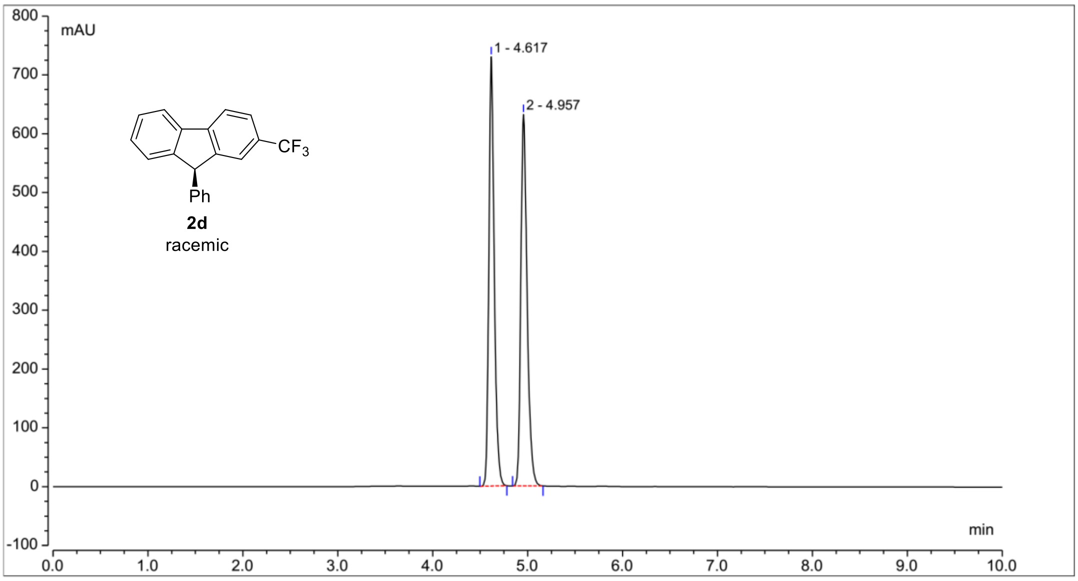

Entry	RT min	Area mAU*min	Height mAU	% Area %	% Height %
1	4.617	48.245	729.719	49.94	53.61
2	4.957	48.354	631.506	50.06	46.39

Supplementary Figure 84. HPLC trace for 9-Phenyl-2-(trifluoromethyl)-9*H*-fluorene (*rac*-2d)

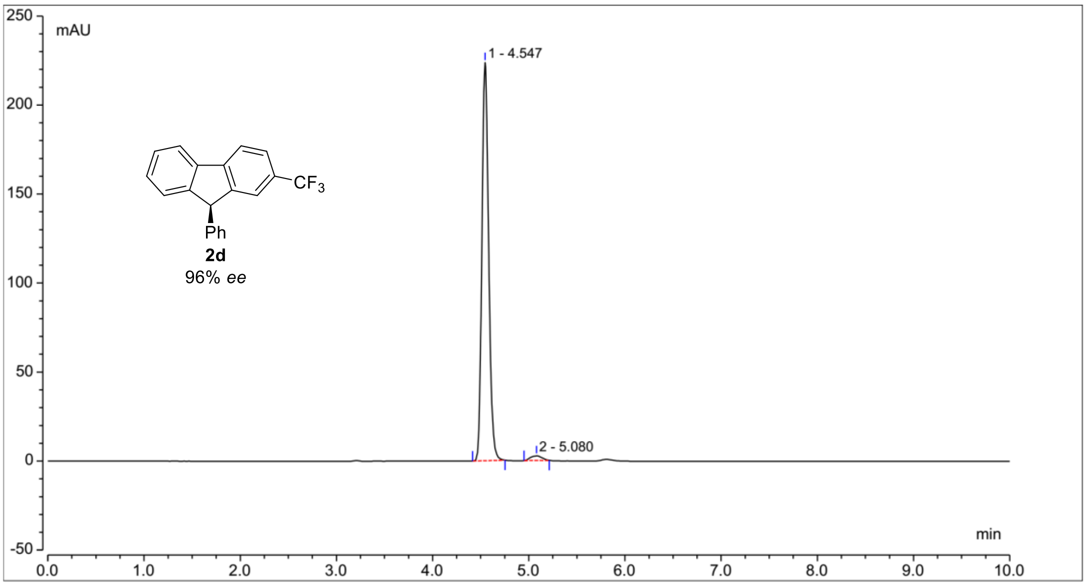

Entry	RT min	Area mAU*min	Height mAU	% Area %	% Height %
1	4.547	18.088	223.612	98.07	98.86
2	5.080	0.357	2.575	1.93	1.14

Supplementary Figure 85. HPLC trace for 9-Phenyl-2-(trifluoromethyl)-9*H*-fluorene (2d)

Conditions: hexane/2-propanol/ethanol = 99.2:0.4:0.4

Flow rate = 1.0 mL/min, λ = 272 nm, Chiral IB-3

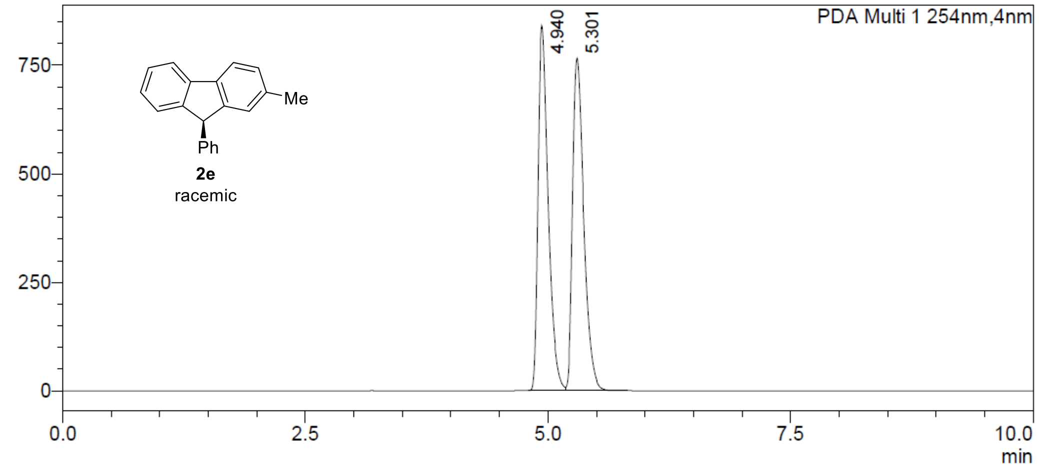

<Peak Table>

PDA Ch1 254nm

Peak#	Ret. Time	Area	Height	Area%	Height%
1	4.940	6148404	841354	49.889	52.326
2	5.301	6175733	766552	50.111	47.674
Total		12324138	1607906	100.000	100.000

Supplementary Figure 86. HPLC trace for 2-Methyl-9-phenyl-9*H*-fluorene (*rac*-2e)

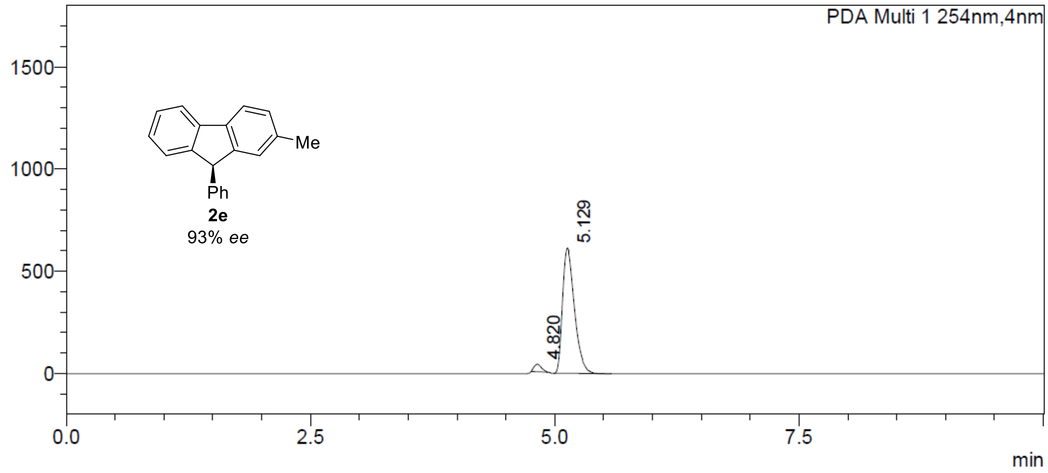

<Peak Table>

PDA Ch1 254nm

Peak#	Ret. Time	Area	Height	Area%	Height%
1	4.820	191688	36409	3.548	5.601
2	5.129	5210422	613587	96.452	94.399

Supplementary Figure 87. HPLC trace for (*S*)-2-Methyl-9-phenyl-9*H*-fluorene (2e)

Conditions: hexane/2-propanol/ethanol = 99.6:0.2:0.2

Flow rate = 1.0 mL/min, λ = 272 nm, Chiral IB-3

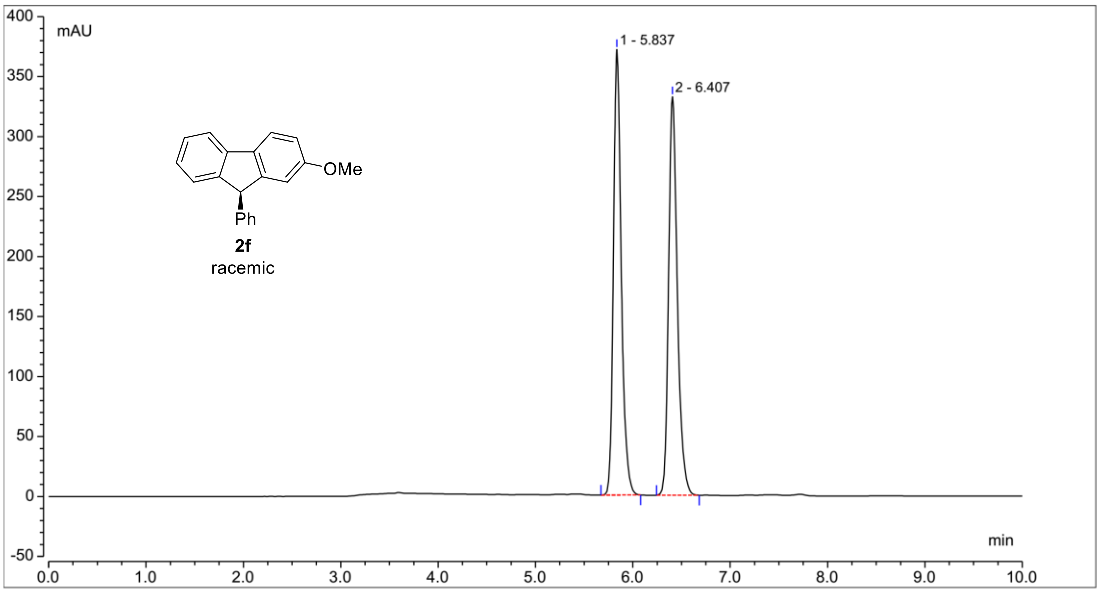

Entry	RT min	Area mAU*min	Height mAU	% Area %	% Height %
1	5.837	34.750	371.328	50.07	52.78
2	6.407	34.647	332.172	49.93	47.22

Supplementary Figure 88. HPLC trace for 2- Methoxy -9-phenyl-9*H*-fluorene (*rac*-2f)

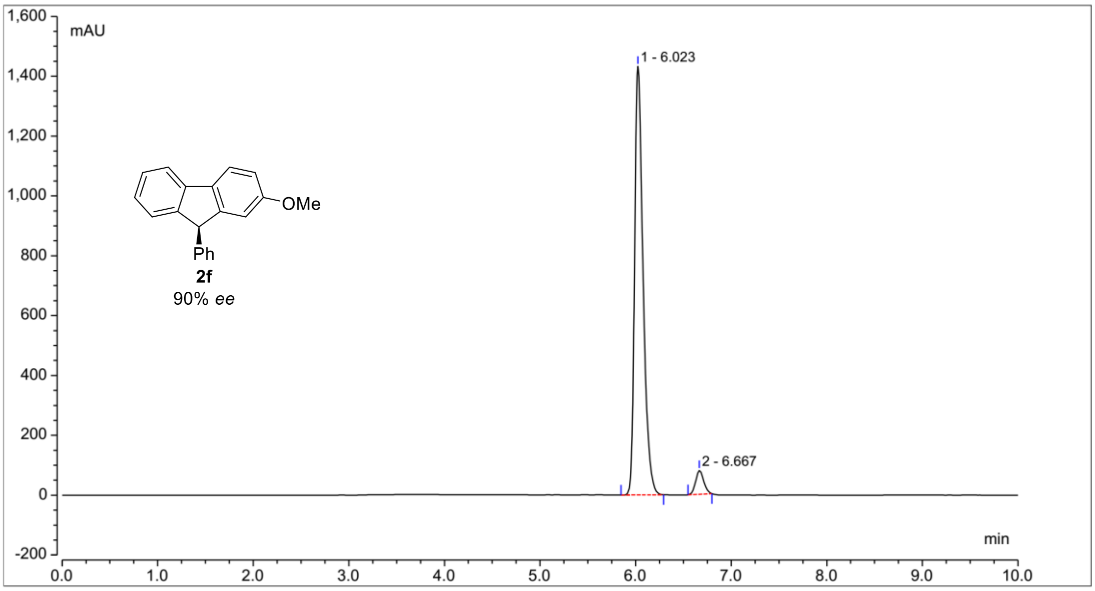

Entry	RT min	Area mAU*min	Height mAU	% Area %	% Height %
1	6.023	147.288	1431.892	94.96	94.75
2	6.667	7.818	79.380	5.04	5.25

Supplementary Figure 89. HPLC trace for 2- Methoxy -9-phenyl-9*H*-fluorene (2f)

Conditions: hexane/2-propanol/ethanol = 99.1:0.45:0.45

Flow rate = 1.0 mL/min, λ = 272 nm, Chiral IB-3

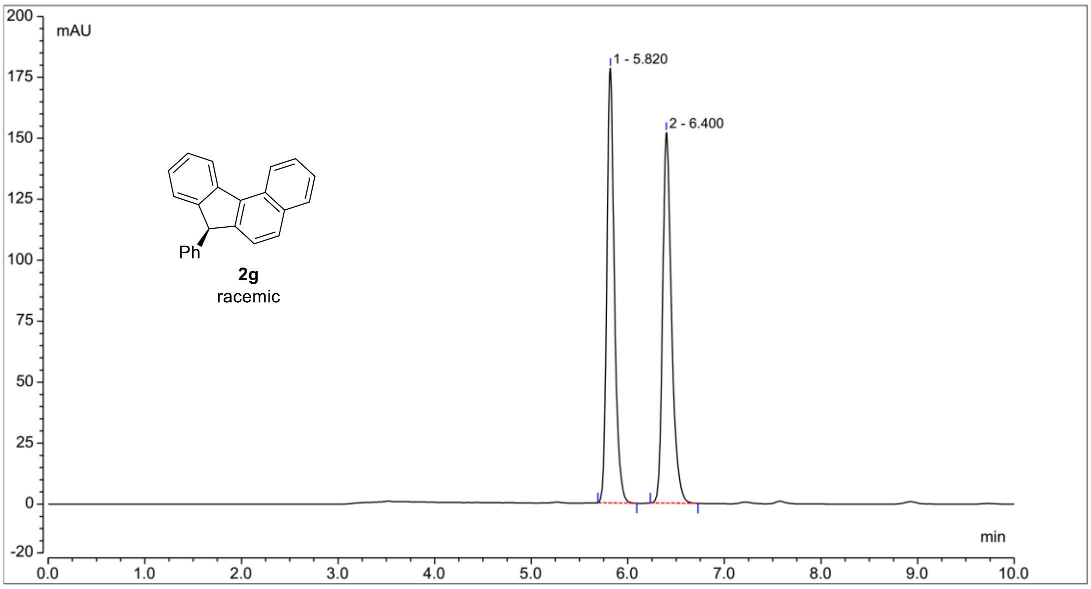

Entry	RT<br>min	Area<br>mAU*min	Height<br>mAU	% Area<br>%	% Height<br>%
1	5.820	16.161	178.185	49.90	53.98
2	6.400	16.226	151.906	50.10	46.02

Supplementary Figure 90. HPLC trace for 7-Phenyl-7*H*-benzo[*c*]fluorine (*rac*-2g)

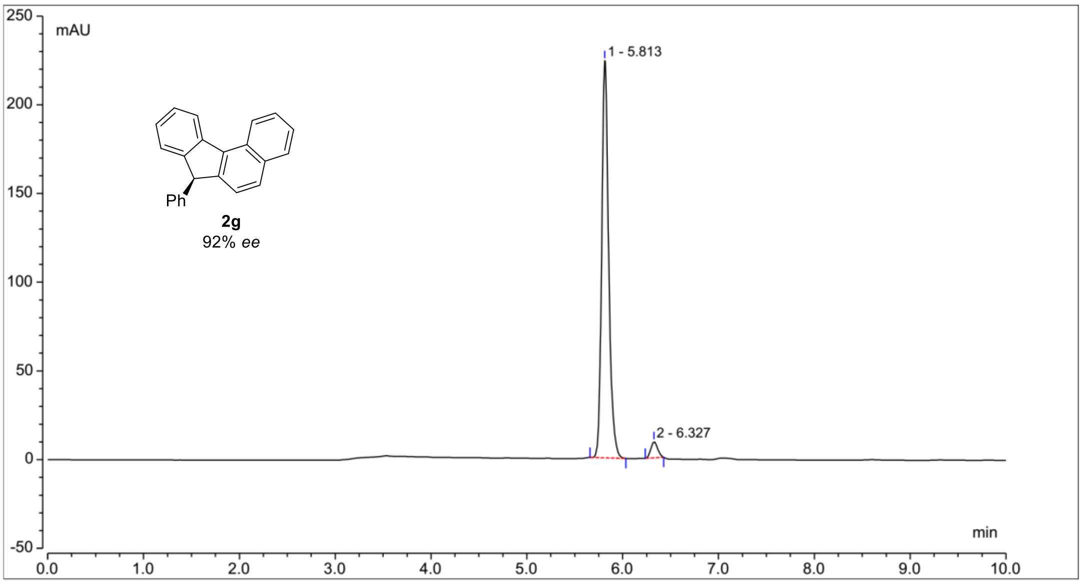

Entry	RT<br>min	Area<br>mAU*min	Height<br>mAU	% Area<br>%	% Height<br>%
1	5.813	18.873	223.801	96.18	96.11
2	6.327	0.749	9.061	3.82	3.89

Supplementary Figure 91. HPLC trace for (*S*)-7-Phenyl-7*H*-benzo[*c*]fluorine (2g)

Conditions: hexane/2-propanol/ethanol = 99.6:0.2:0.2

Flow rate = 1.0 mL/min, λ = 272 nm, Chiral IB-3

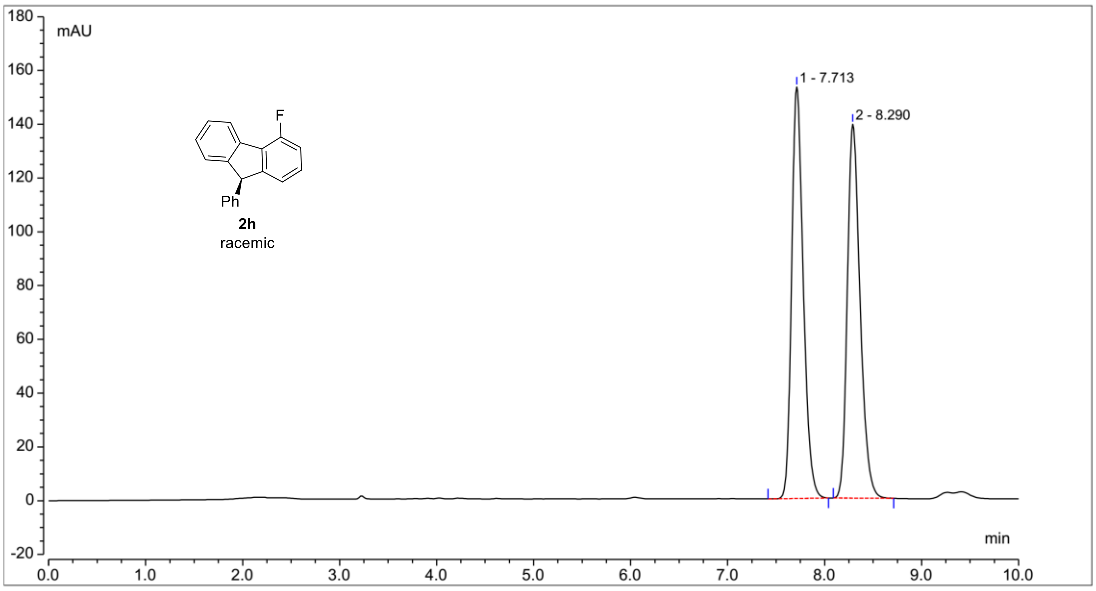

Entry	RT<br>min	Area<br>mAU*min	Height<br>mAU	% Area<br>%	% Height<br>%
1	7.713	21.239	153.067	50.02	52.40
2	8.290	21.219	139.019	49.98	47.60

Supplementary Figure 92. HPLC trace for 4-Fluoro-9-phenyl-9*H*-fluorene (*rac*-2h)

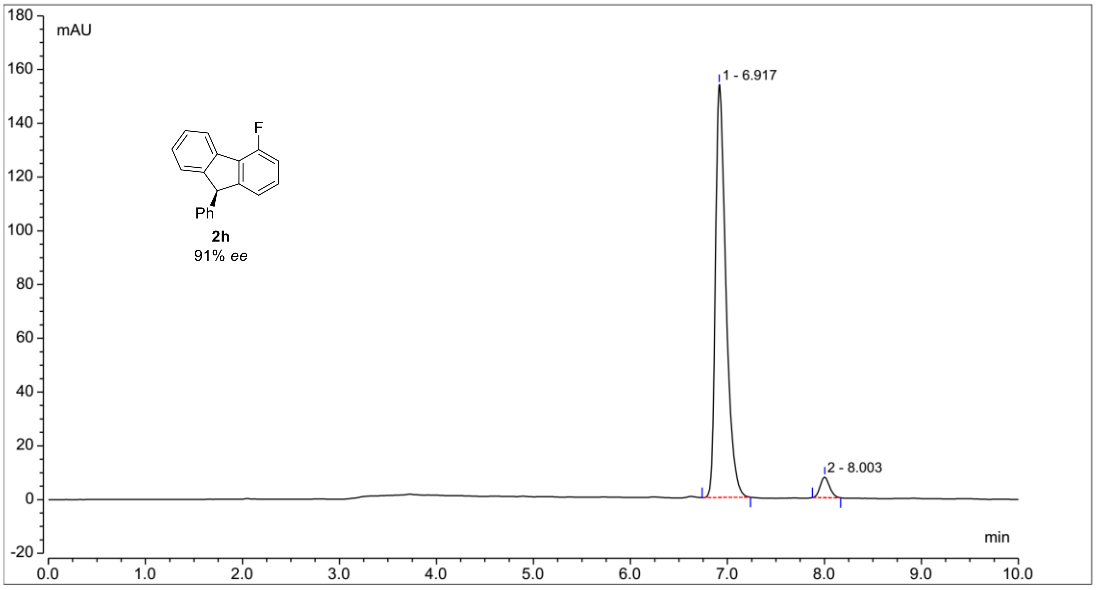

Entry	RT<br>min	Area<br>mAU*min	Height<br>mAU	% Area<br>%	% Height<br>%
1	6.917	19.209	153.648	95.71	95.21
2	8.003	0.861	7.722	4.29	4.79

Supplementary Figure 93. HPLC trace for (*S*)-4-Fluoro-9-phenyl-9*H*-fluorene (2h)

Conditions: hexane/2-propanol = 100:0

Flow rate = 1.0 mL/min, λ = 272 nm, Chiral IB-3

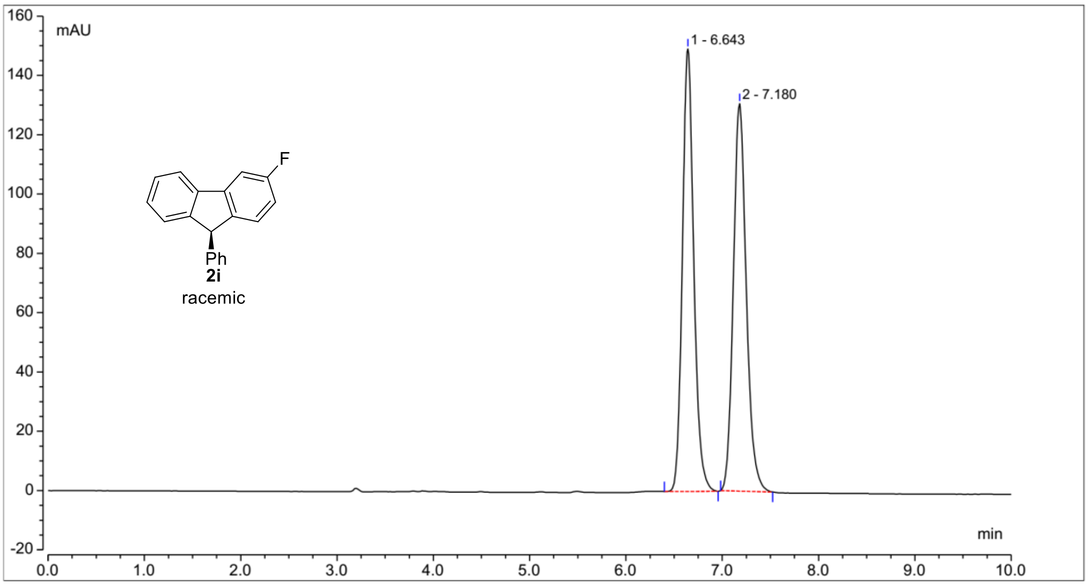

Entry	RT<br>min	Area<br>mAU*min	Height<br>mAU	% Area<br>%	% Height<br>%
1	6.643	20.456	149.203	50.16	53.31
2	7.180	20.329	130.656	49.84	46.69

Supplementary Figure 94. HPLC trace for 3-Fluoro-9-phenyl-*9H*-fluorene (*rac*-2i)

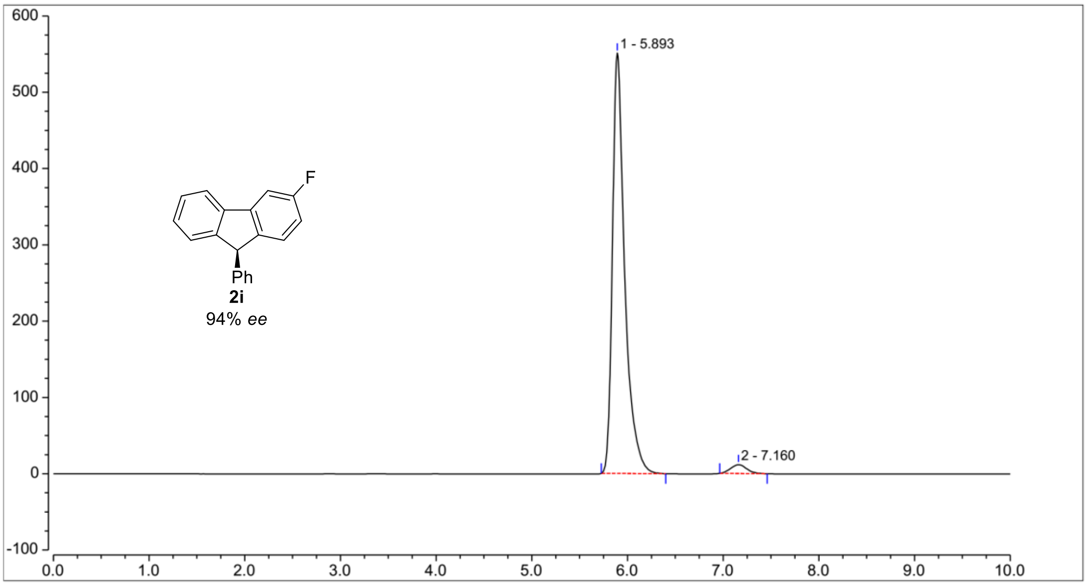

Entry	RT<br>min	Area<br>mAU*min	Height<br>mAU	% Area<br>%	% Height<br>%
1	5.893	81.862	550.835	97.29	97.93
2	7.160	2.277	11.638	2.71	2.07

Supplementary Figure 95. HPLC trace for (*S*)-3-Fluoro-9-phenyl-*9H*-fluorene (2i)

Conditions: hexane/2-propanol/ethanol = 99:0.5:0.5

Flow rate = 1.0 mL/min, λ = 272 nm, Chiral IB-3

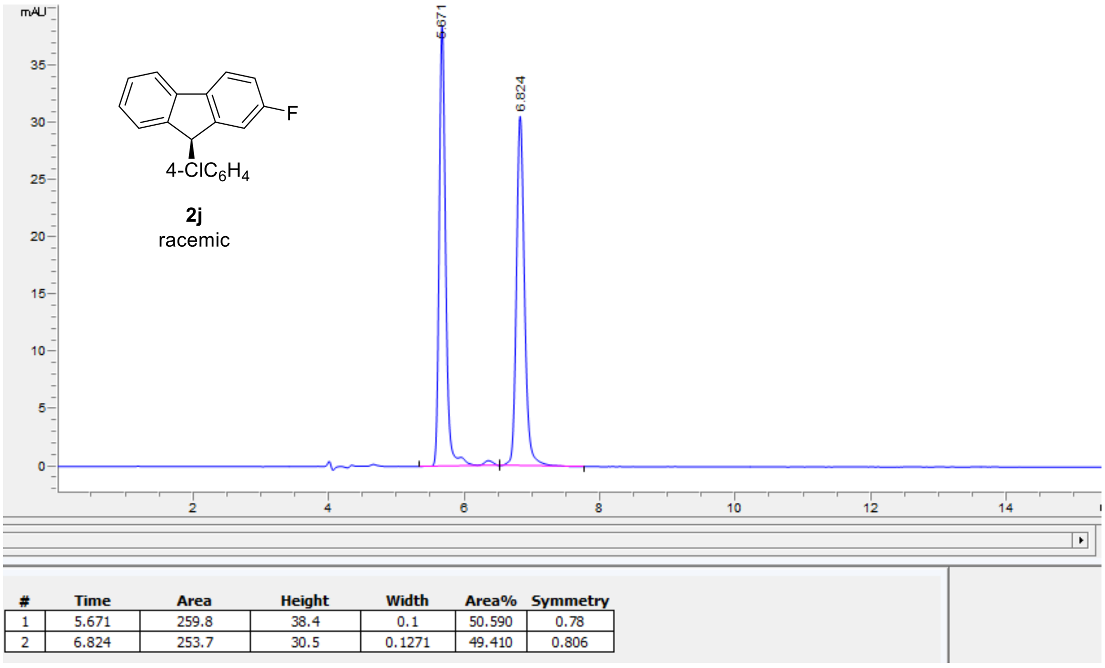

#	Time	Area	Height	Width	Area%	Symmetry
1	5.671	259.8	38.4	0.1	50.590	0.78
2	6.824	253.7	30.5	0.1271	49.410	0.806

Supplementary Figure 96. HPLC trace for 9-(4-Chlorophenyl)-2-fluoro-9*H*-fluorene (*rac*-2j)

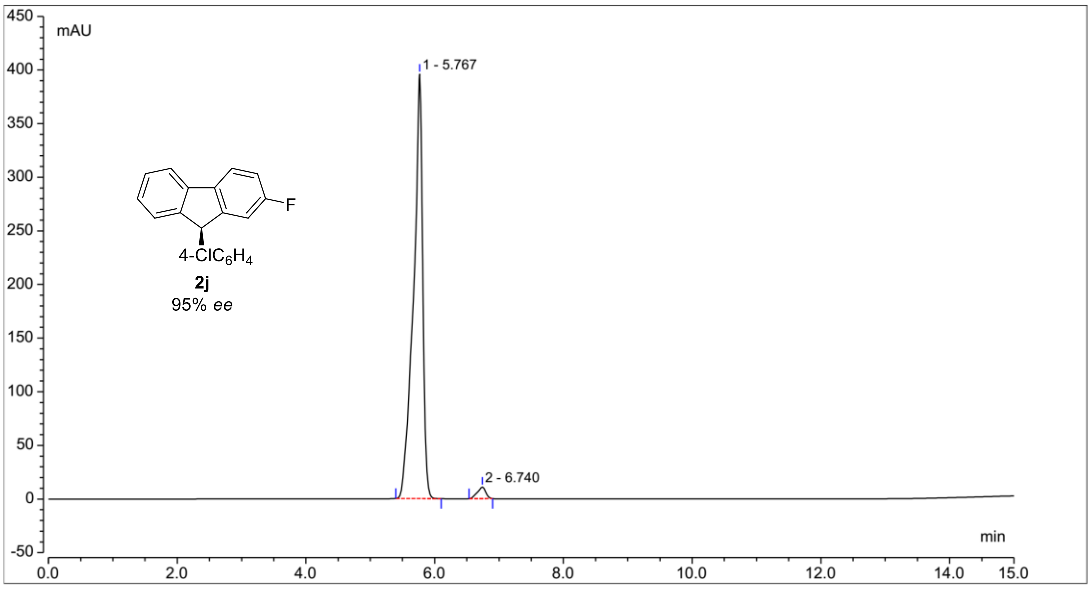

Entry	RT min	Area mAU*min	Height mAU	% Area %	% Height %
1	5.767	65.403	395.782	97.57	97.34
2	6.740	1.628	10.835	2.43	2.66

Supplementary Figure 97. HPLC trace for (*S*)-9-(4-Chlorophenyl)-2-fluoro-9*H*-fluorene (2j)

Conditions: hexane/2-propanol/ethanol = 99.2:0.4:0.4

Flow rate = 1.0 mL/min, λ = 272 nm, Chiral IB-3

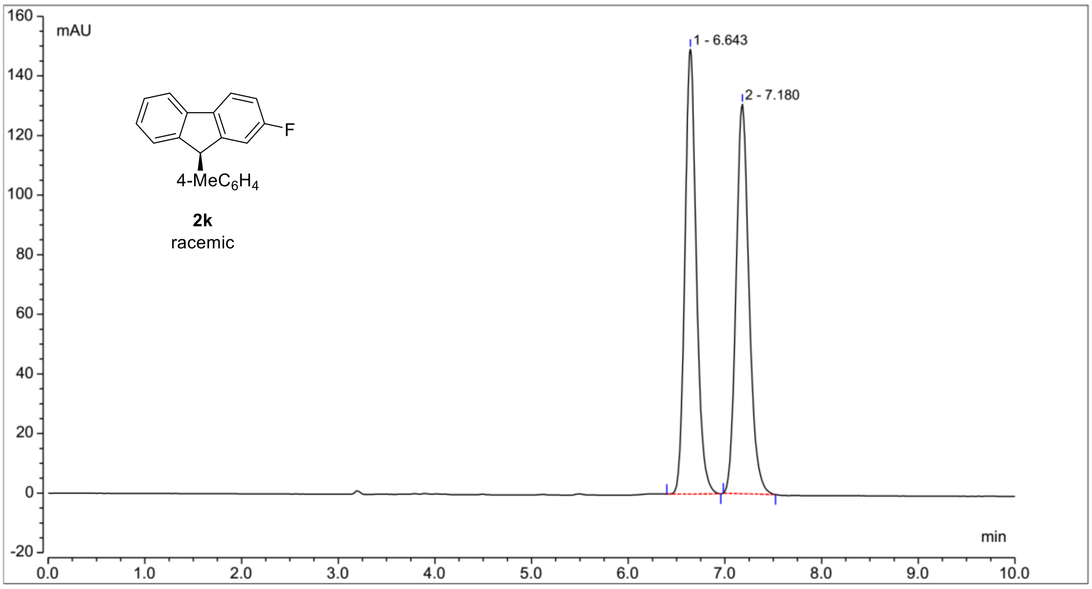

Entry	RT<br>min	Area<br>mAU*min	Height<br>mAU	% Area<br>%	% Height<br>%
1	6.643	20.456	149.203	50.16	53.31
2	7.180	20.329	130.656	49.84	46.69

Supplementary Figure 98. HPLC trace for 2-Fluoro-9-(*p*-tolyl)-9*H*-fluorene (*rac*-2k)

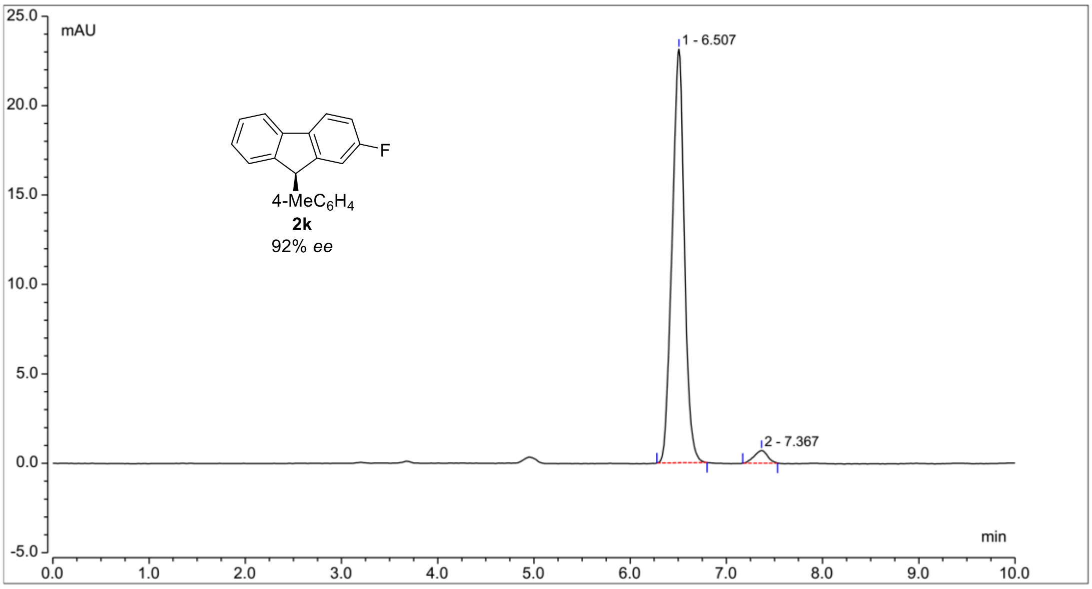

Entry	RT<br>min	Area<br>mAU*min	Height<br>mAU	% Area<br>%	% Height<br>%
1	6.507	3.411	23.124	96.78	97.04
2	7.367	0.114	0.706	3.22	2.96

Supplementary Figure 99. HPLC trace for 2-Fluoro-9-(*p*-tolyl)-9*H*-fluorene (2k)

Conditions: hexane/2-propanol/ethanol= 97.5:1.25:1.25

Flow rate = 1.0 mL/min, λ = 272 nm, Chiral IB-3

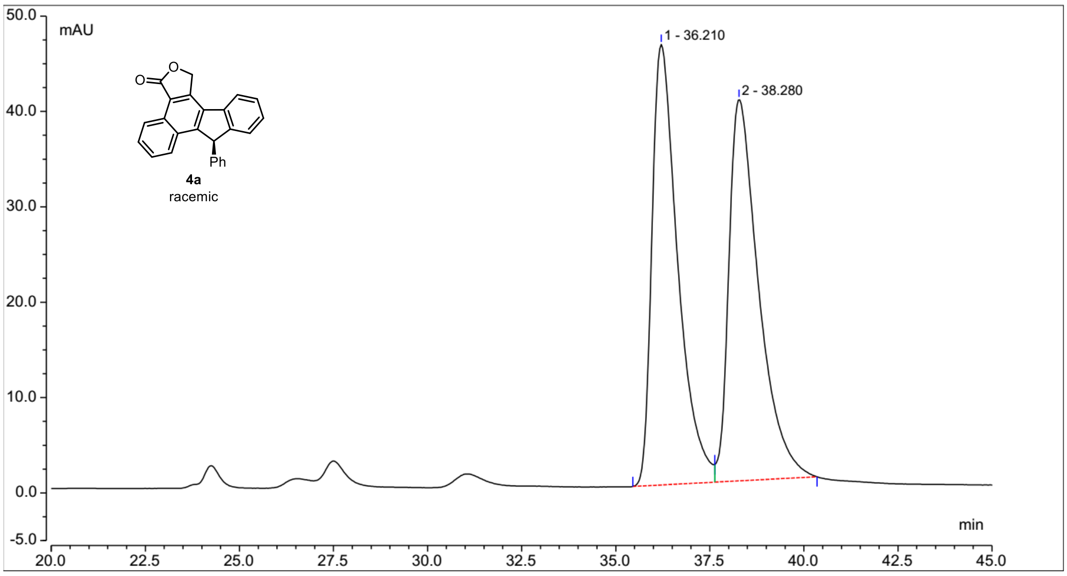

Entry	RT<br>min	Area<br>mAU*min	Height<br>mAU	% Area<br>%	% Height<br>%
1	36.210	36.731	46.183	49.98	53.58
2	38.280	36.757	40.009	50.02	46.42

Supplementary Figure 100. HPLC trace for 8-Phenyl-1*H*-benzo[1,2]fluoreno[3,4-*c*] furan-3(8*H*)-one (*rac*-4a)

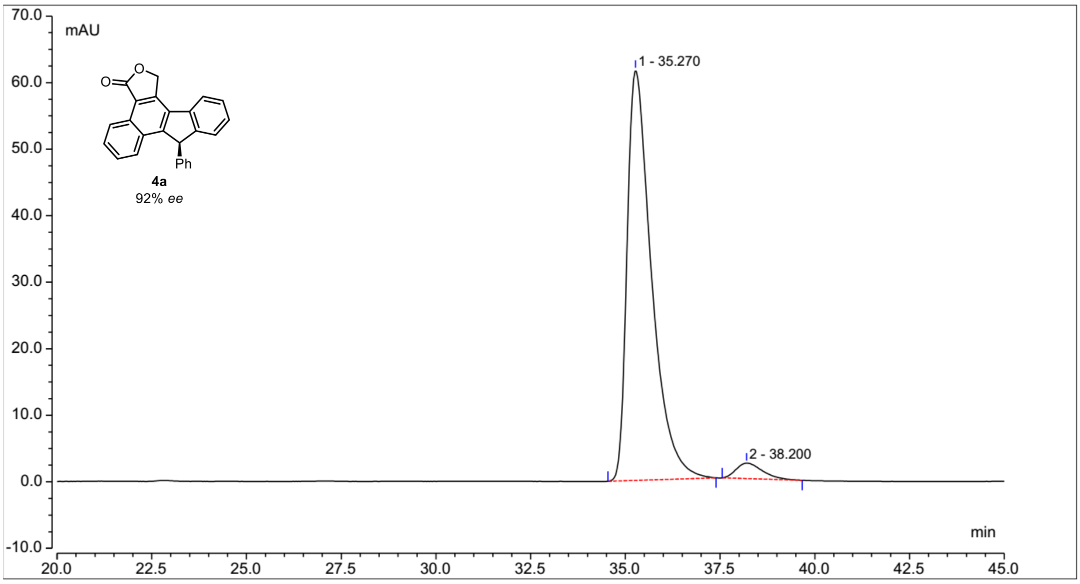

Entry	RT<br>min	Area<br>mAU*min	Height<br>mAU	% Area<br>%	% Height<br>%
1	35.270	46.075	61.649	96.09	96.36
2	38.200	1.877	2.332	3.91	3.64

Supplementary Figure 101. HPLC trace for (*R*)-8-Phenyl-1*H*-benzo[1,2]fluoreno[3,4-*c*] furan-3(8*H*)-one (4a)

Conditions: hexane/2-propanol/ethanol= 97.5:1.25:1.25

Flow rate = 1.0 mL/min, λ = 272 nm, Chiral IB-3

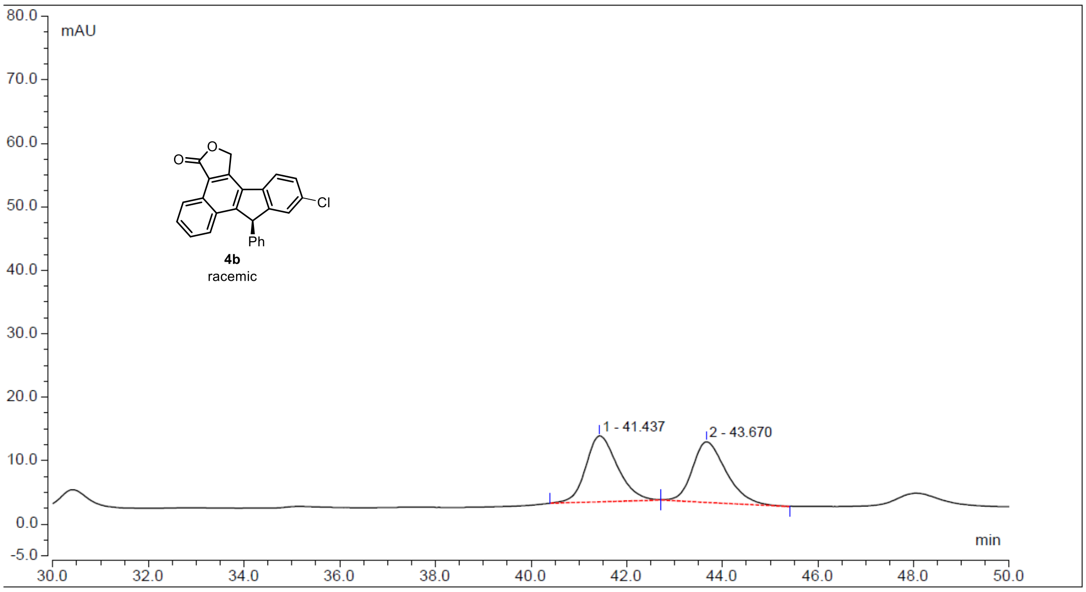

Entry	RT min	Area mAU*min	Height mAU	% Area %	% Height %
1	41.437	8.003	10.389	50.50	52.12
2	43.670	7.843	9.545	49.50	47.88

Supplementary Figure 102. HPLC trace for 0-Chloro-8-phenyl-1*H*-benzo[1,2]fluoreno[3,4-*c*] furan-3(8*H*)-one (*rac*-4b)

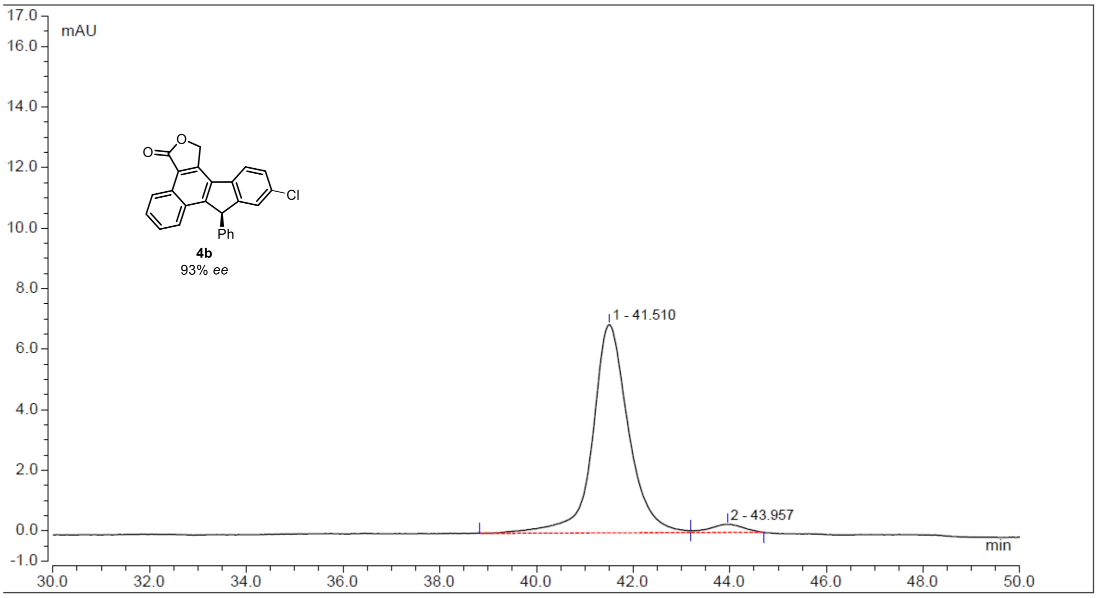

Entry	RT min	Area mAU*min	Height mAU	% Area %	% Height %
1	41.510	5.914	6.875	96.52	96.24
2	43.957	0.213	0.269	3.48	3.76

Supplementary Figure 103. HPLC trace for (*R*)-10-Chloro-8-phenyl-1*H*-benzo[1,2] fluoreno[3,4-*c*]furan-3(8*H*)-one (4b)

Conditions: hexane/2-propanol = 92:08

Flow rate = 1.0 mL/min, λ = 272 nm, Chiral IA-3

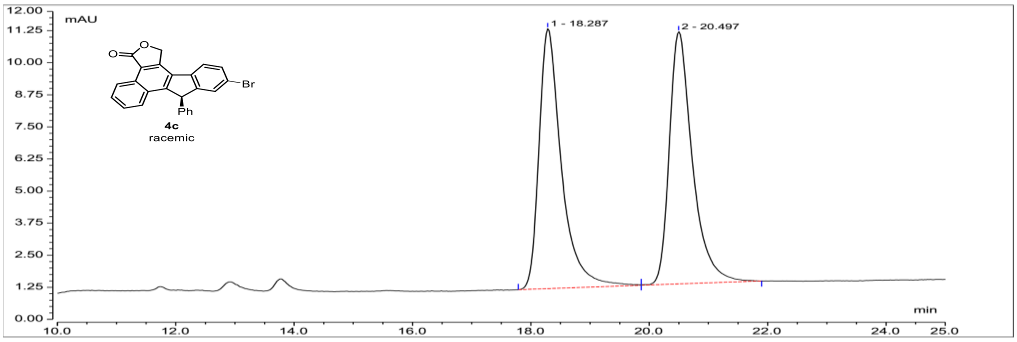

Entry	RT min	Area mAU*min	Height mAU	% Area %	% Height %
1	18.287	4.448	10.128	50.09	50.77
2	20.497	4.432	9.822	49.91	49.23

Supplementary Figure 104. HPLC trace for 10-Bromo-8-phenyl-1*H*-benzo[1,2]fluoreno[3,4-c] furan-3(8*H*)-one (*rac*-4c)

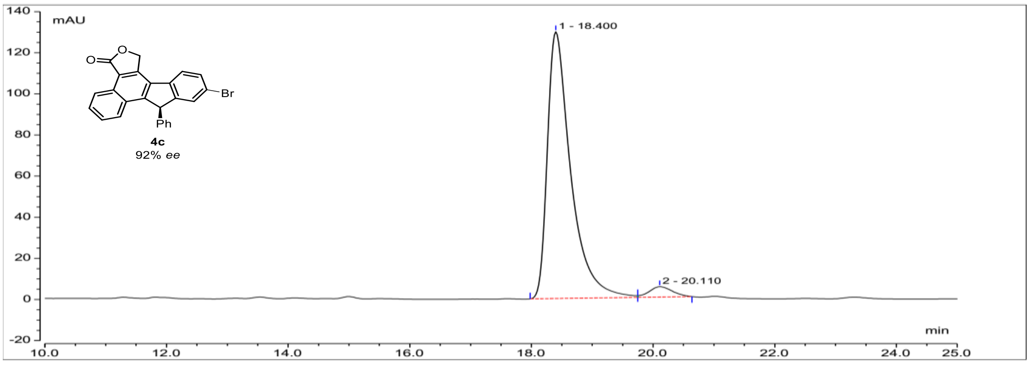

Entry	RT min	Area mAU*min	Height mAU	% Area %	% Height %
1	18.400	57.677	129.721	96.28	96.27
2	20.110	2.229	5.026	3.72	3.73

Supplementary Figure 105. HPLC trace for (*R*)-10-Bromo-8-phenyl-1*H*-benzo[1,2] fluoreno[3,4-c]furan-3(8*H*)-one (4c)

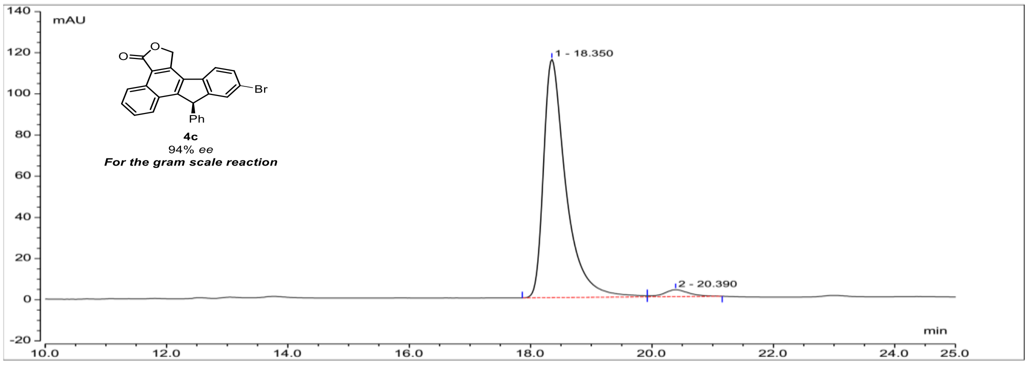

Entry	RT min	Area mAU*min	Height mAU	% Area %	% Height %
1	18.350	49.530	115.738	96.91	97.20
2	20.390	1.580	3.329	3.09	2.80

Supplementary Figure 106. HPLC trace for (*R*)-10-Bromo-8-phenyl-1*H*-benzo[1,2] fluoreno[3,4-c]furan-3(8*H*)-one (4c) (gram scale)

Conditions: hexane/2-propanol = 95:05

Flow rate = 1.0 mL/min, λ = 272 nm, Chiral IA-3

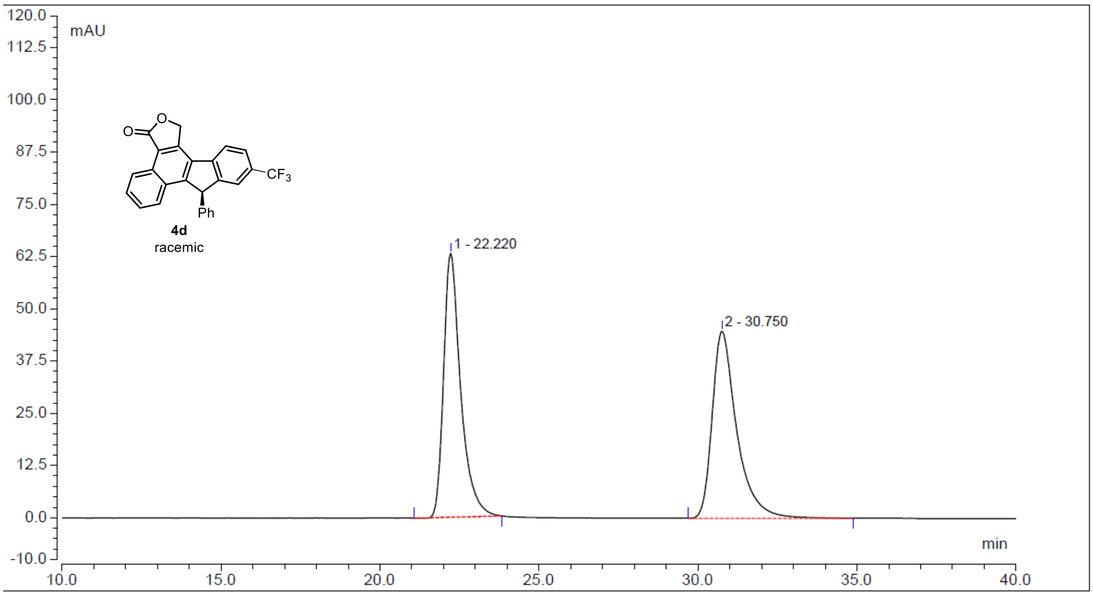

Entry	RT min	Area mAU*min	Height mAU	% Area %	% Height %
1	22.220	39.240	63.172	49.42	58.52
2	30.750	40.165	44.782	50.58	41.48

Supplementary Figure 107. HPLC trace for 8-Phenyl-10-(trifluoromethyl)-1*H*-benzo[1,2] fluoreno[3,4-*c*]furan-3(8*H*)-one (*rac*-4d)

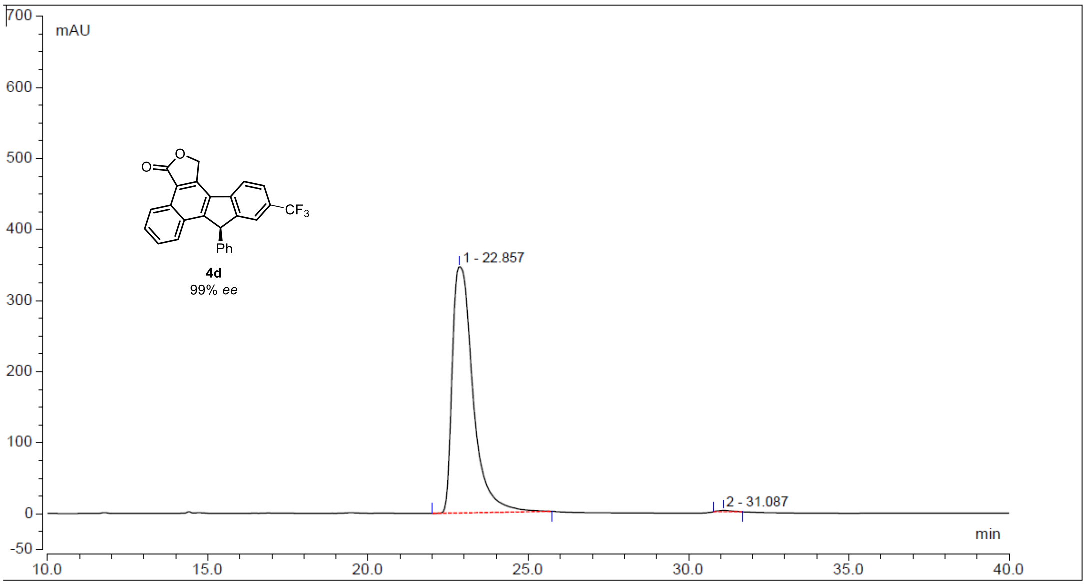

Entry	RT min	Area mAU*min	Height mAU	% Area %	% Height %
1	22.857	260.500	346.368	99.65	99.49
2	31.087	0.925	1.787	0.35	0.51

Supplementary Figure 108. HPLC trace for (*R*)-8-Phenyl-10-(trifluoromethyl)-1*H*-benzo[1,2] fluoreno[3,4-*c*]furan-3(8*H*)-one (4d)

Conditions: hexane/2-propanol = 96:04

Flow rate = 1.0 mL/min, λ = 272 nm, Chiral IA-3

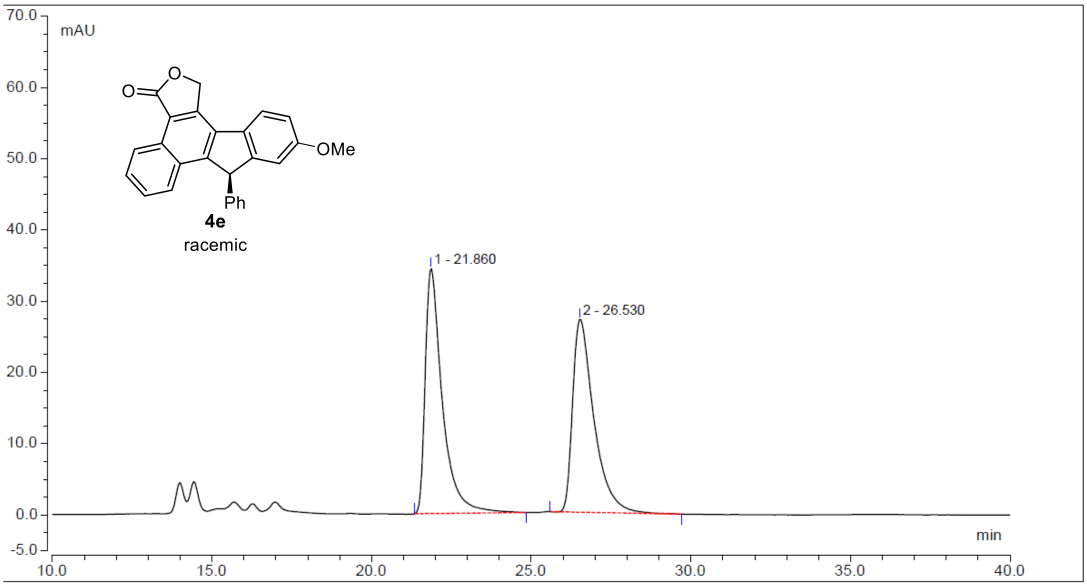

Entry	RT min	Area mAU*min	Height mAU	% Area %	% Height %
1	21.860	21.349	34.412	50.54	55.94
2	26.530	20.892	27.100	49.46	44.06

Supplementary Figure 109. HPLC trace for 10-Methoxy-8-phenyl-1*H*-benzo[1,2] fluoreno[3,4-*c*]furan-3(8*H*)-one (*rac*-4e)

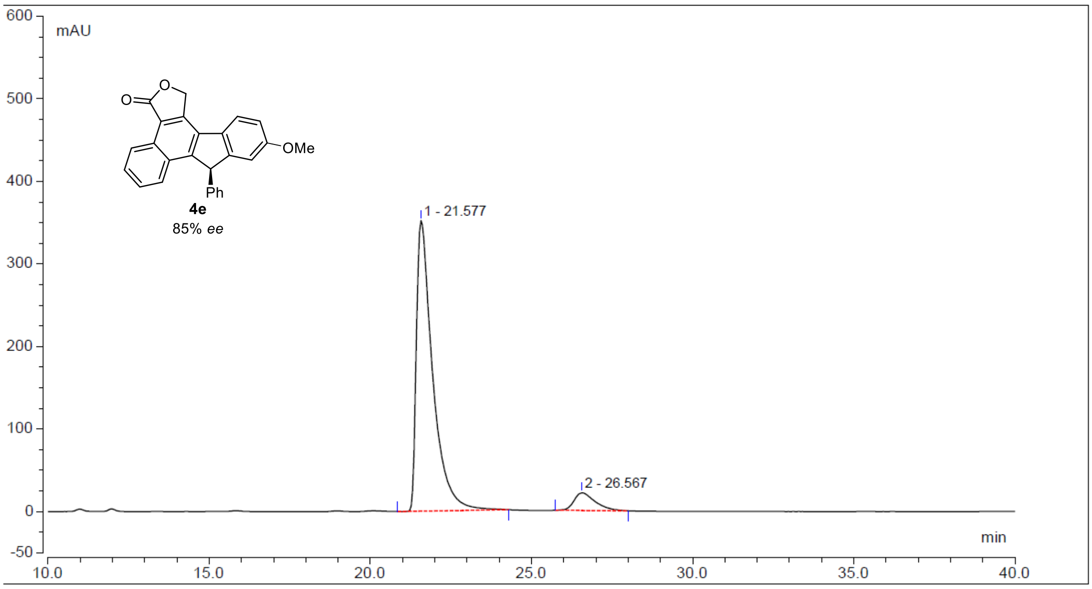

Entry	RT min	Area mAU*min	Height mAU	% Area %	% Height %
1	21.577	205.865	352.246	92.69	94.30
2	26.567	16.233	21.300	7.31	5.70

Supplementary Figure 110. HPLC trace for (*R*)-10-Methoxy-8-phenyl-1*H*-benzo[1,2] fluoreno[3,4-*c*]furan-3(8*H*)-one (4e)

Conditions: hexane/2-propanol = 93:07

Flow rate = 0.5 mL/min, λ = 272 nm, Chiral IA-3

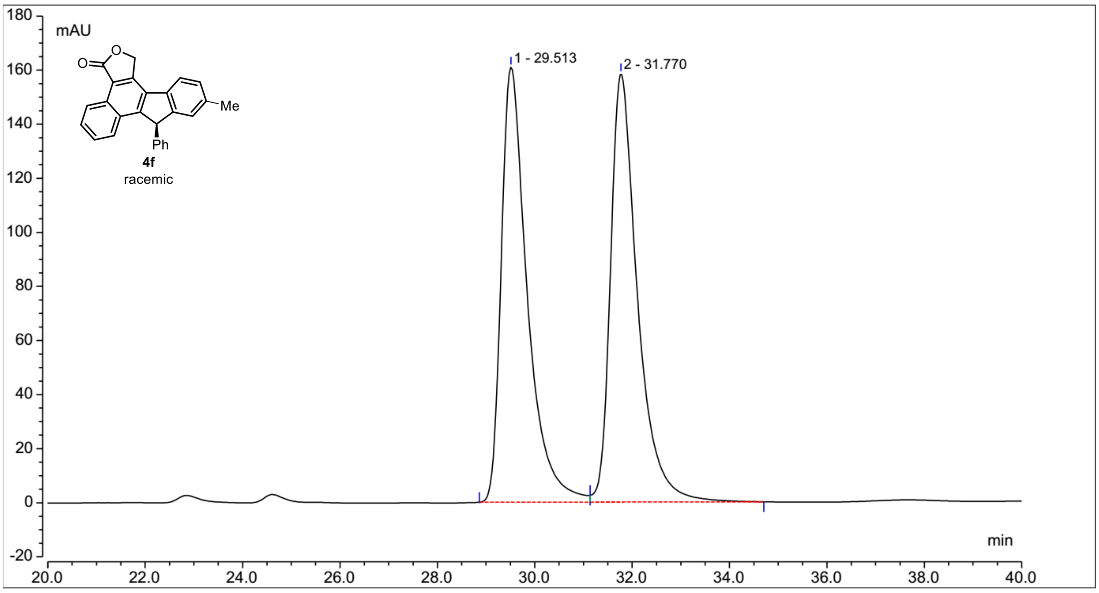

Entry	RT min	Area mAU*min	Height mAU	% Area %	% Height %
1	29.513	99.704	160.936	49.51	50.39
2	31.770	101.661	158.438	50.49	49.61

Supplementary Figure 111. HPLC trace for (*R*)-10-methyl-8-phenyl-1*H*-benzo[1,2] fluoreno[3,4-*c*]furan-3(8*H*)-one (*rac*-4f)

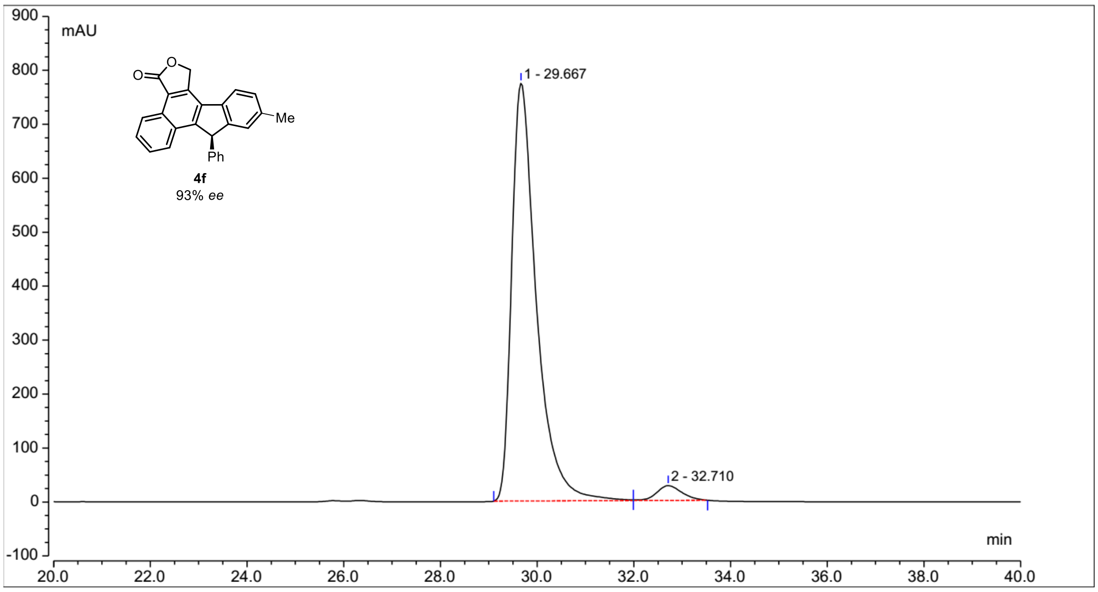

Entry	RT min	Area mAU*min	Height mAU	% Area %	% Height %
1	29.667	457.605	774.713	96.51	96.59
2	32.710	16.567	27.386	3.49	3.41

Supplementary Figure 112. HPLC trace for (*R*)-10-methyl-8-phenyl-1*H*-benzo[1,2] fluoreno[3,4-*c*]furan-3(8*H*)-one (4f)

Conditions: hexane/2-propanol /ethanol= 98.5:0.75:0.75

Flow rate = 1.0 mL/min, λ = 272 nm, Chiral IB-3

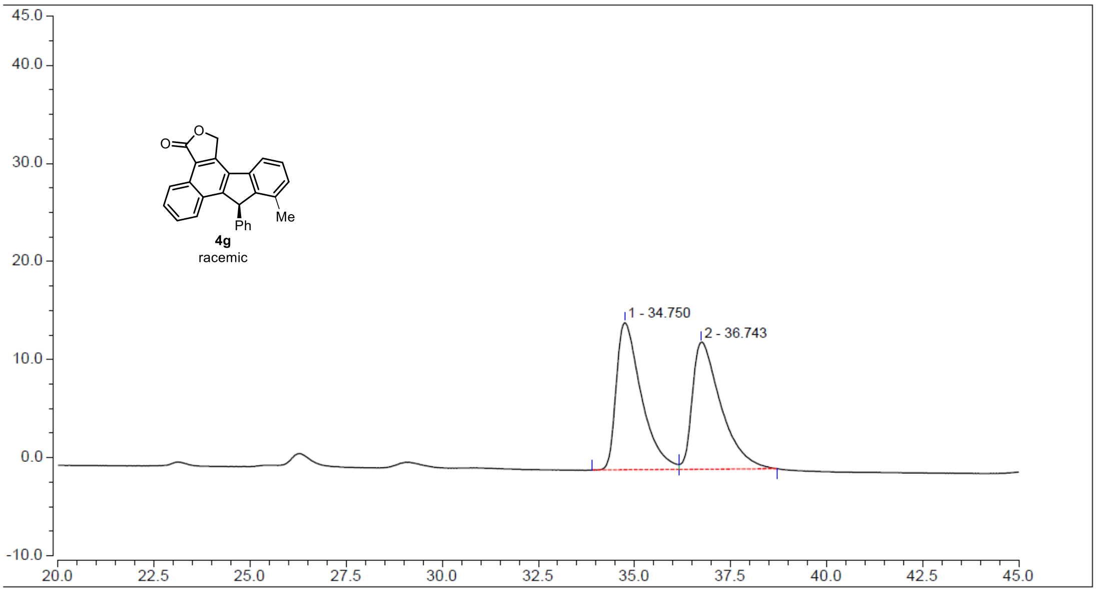

Entry	RT<br>min	Area<br>mAU*min	Height<br>mAU	% Area<br>%	% Height<br>%
1	34.750	11.412	14.997	50.40	53.65
2	36.743	11.229	12.958	49.60	46.35

Supplementary Figure 113. HPLC trace for (*R*)-9-Methyl-8-phenyl-1*H*-benzo[1,2] fluoreno[3,4-*c*]furan-3(8*H*)-one (*rac*-4g)

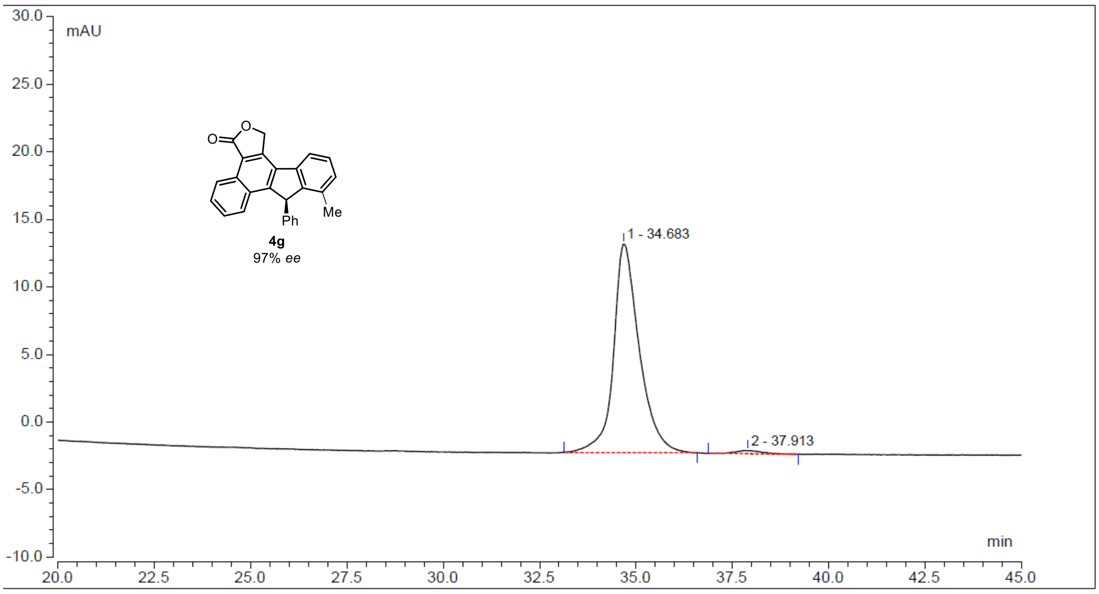

Entry	RT<br>min	Area<br>mAU*min	Height<br>mAU	% Area<br>%	% Height<br>%
1	34.683	11.985	15.463	98.50	98.40
2	37.913	0.182	0.252	1.50	1.60

Supplementary Figure 114. HPLC trace for (*R*)-9-Methyl-8-phenyl-1*H*-benzo[1,2] fluoreno[3,4-*c*]furan-3(8*H*)-one (4g)

Conditions: hexane/2-propanol /ethanol= 97.5:1.25:1.25

Flow rate = 1.0 mL/min, λ = 272 nm, Chiral IB-3

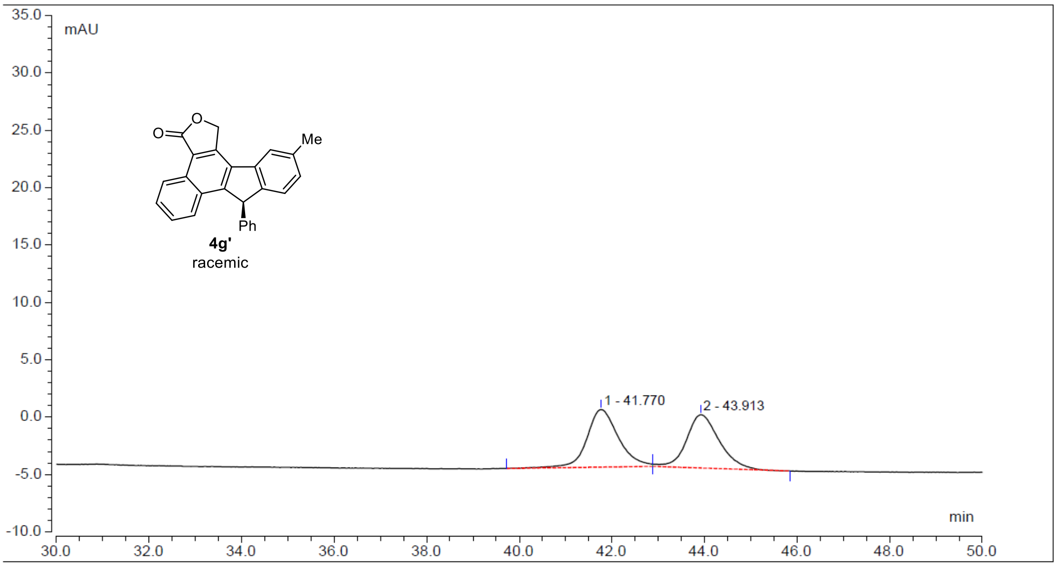

Entry	RT min	Area mAU*min	Height mAU	% Area %	% Height %
1	41.770	3.964	5.026	50.97	51.98
2	43.913	3.812	4.643	49.03	48.02

Supplementary Figure 115. HPLC trace for 12-Methyl-8-phenyl-1*H*-benzo[1,2] fluoreno[3,4-*c*]furan-3(8*H*)-one (*rac*-4g')

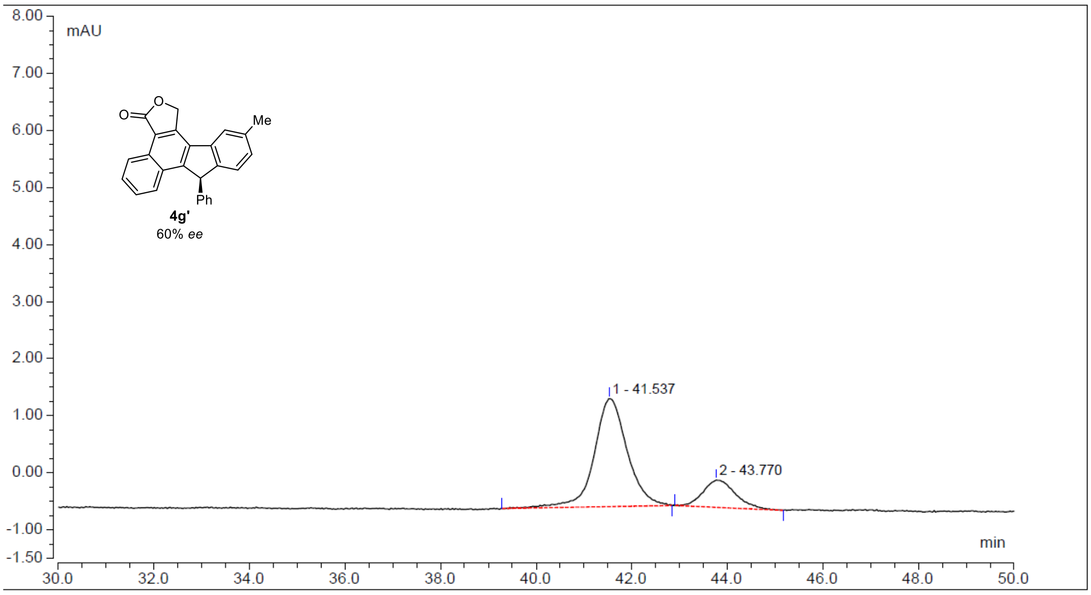

Entry	RT min	Area mAU*min	Height mAU	% Area %	% Height %
1	41.537	1.518	1.900	80.09	79.84
2	43.770	0.378	0.480	19.91	20.16

Supplementary Figure 116. HPLC trace for (*R*)-12-Methyl-8-phenyl-1*H*-benzo[1,2] fluoreno[3,4-*c*]furan-3(8*H*)-one (4g')

Conditions: hexane/2-propanol = 95:05

Flow rate = 1.0 mL/min, λ = 272 nm, Chiral IB-3

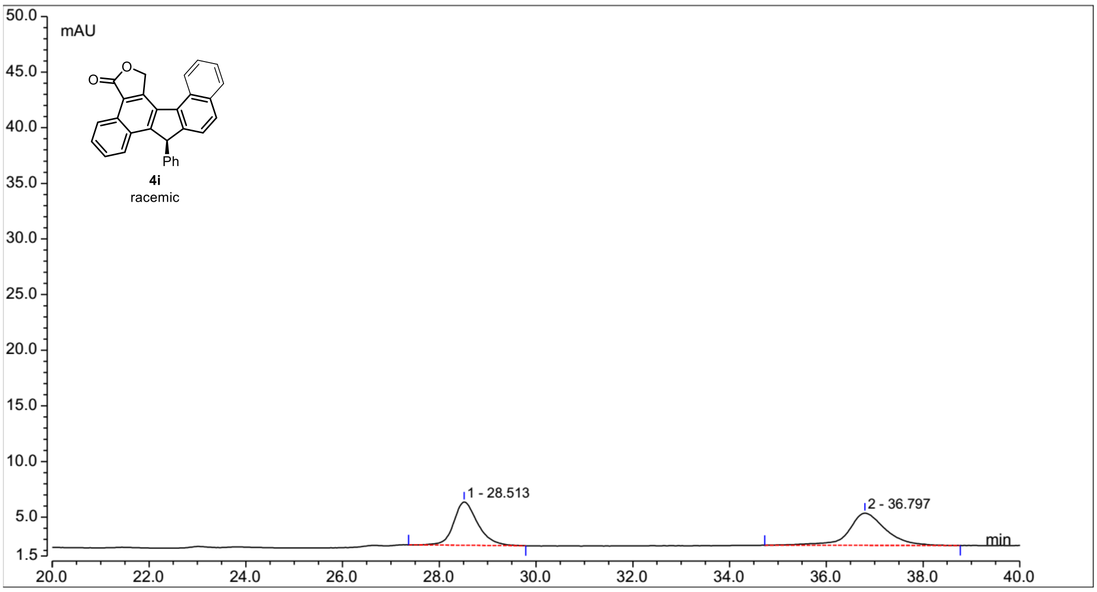

Entry	RT<br>min	Area<br>mAU*min	Height<br>mAU	% Area<br>%	% Height<br>%
1	28.513	2.240	3.888	46.68	57.28
2	36.797	2.558	2.900	53.32	42.72

Supplementary Figure 117. HPLC trace for 8-Phenyl-1*H*-dibenzo[1,2:5,6]fluoreno[3,4-*c*] furan-3(8*H*)-one (*rac*-4i)

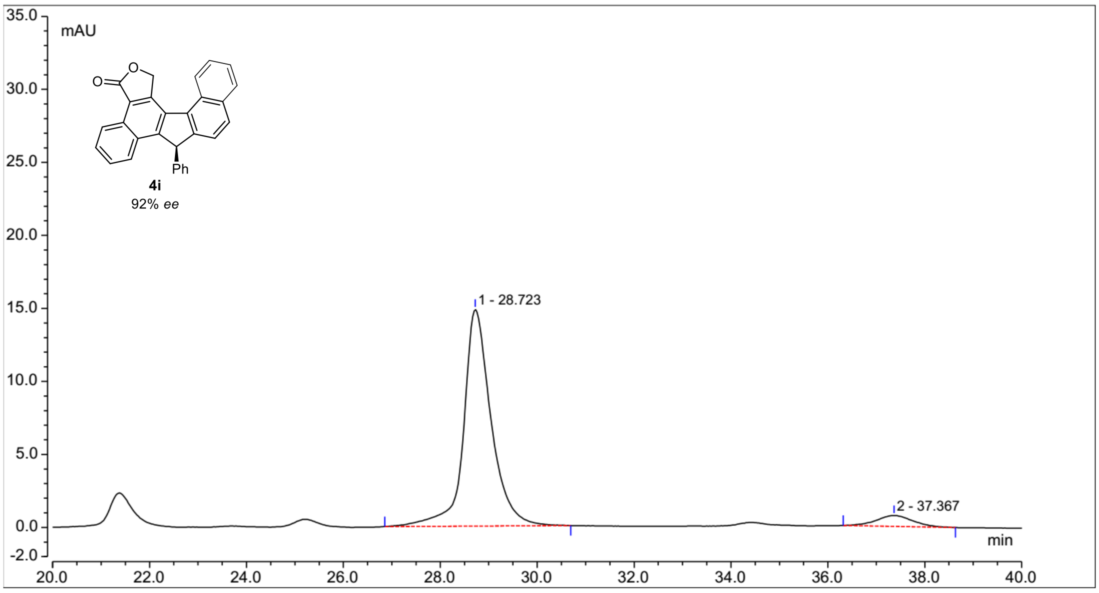

Entry	RT<br>min	Area<br>mAU*min	Height<br>mAU	% Area<br>%	% Height<br>%
1	28.723	9.809	14.834	96.24	95.22
2	37.367	0.655	0.745	3.76	4.78

Supplementary Figure 118. HPLC trace for (*R*)-8-Phenyl-1*H*-dibenzo[1,2:5,6]Fluoreno [3,4-*c*]furan-3(8*H*)-one (4i)

Conditions: hexane/2-propanol = 96:04

Flow rate = 1.0 mL/min, λ = 272 nm, Chiral IA-3

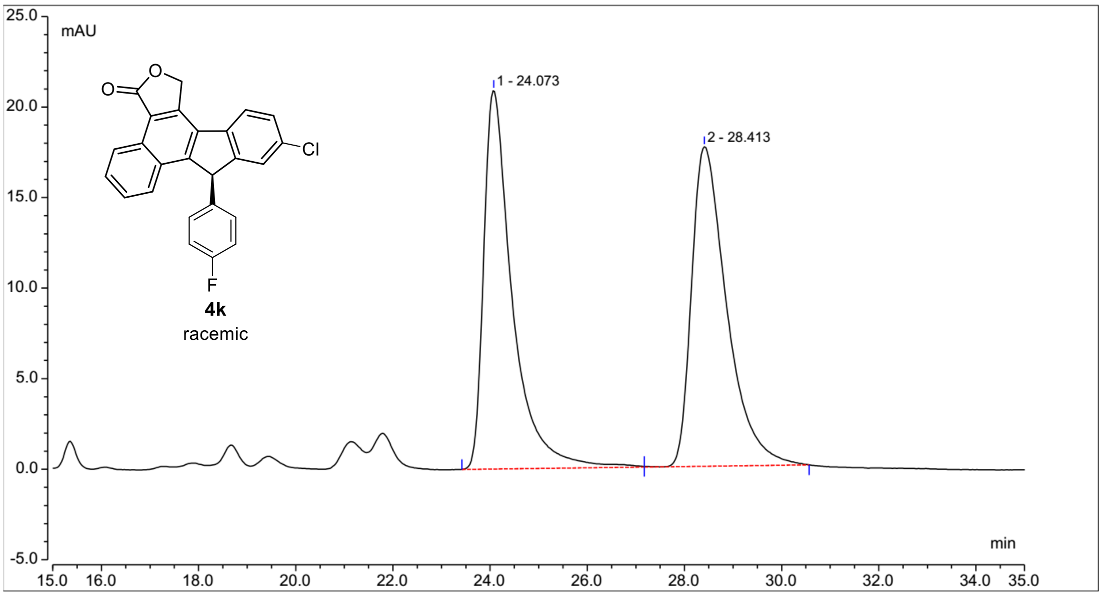

Entry	RT<br>min	Area<br>mAU*min	Height<br>mAU	% Area<br>%	% Height<br>%
1	24.073	14.262	20.914	49.11	54.23
2	28.413	14.780	17.650	50.89	45.77

Supplementary Figure 119. HPLC trace for 10-Chloro-8-(4-fluorophenyl)-1*H*-benzo[1,2] fluoreno[3,4-*c*]furan-3(8*H*)-one (*rac*-4k)

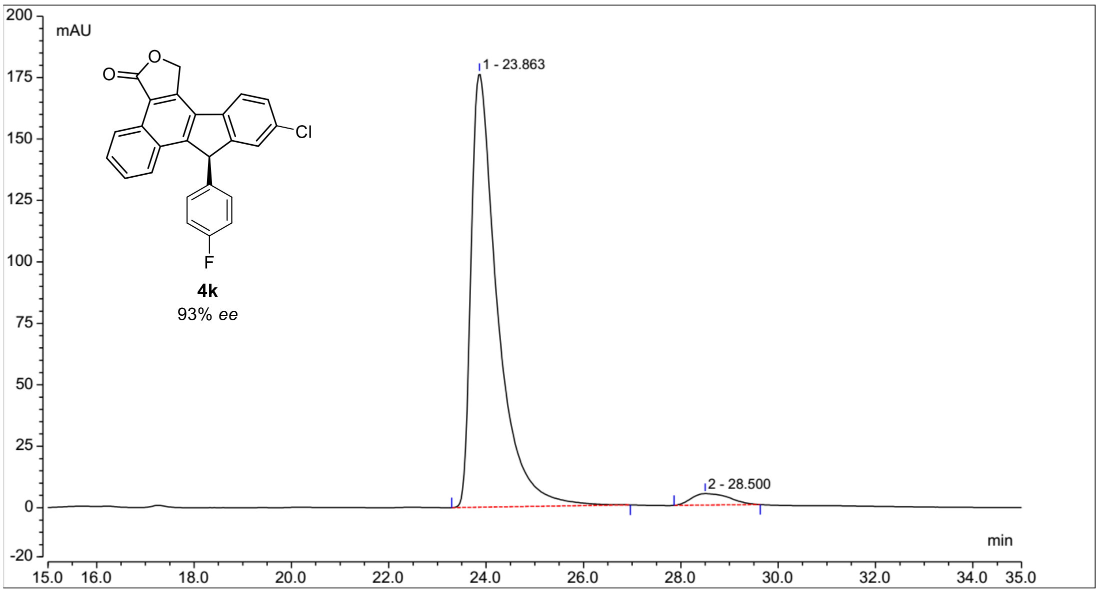

Entry	RT<br>min	Area<br>mAU*min	Height<br>mAU	% Area<br>%	% Height<br>%
1	23.863	110.650	176.502	96.35	97.44
2	28.500	4.191	4.637	3.65	2.56

Supplementary Figure 120. HPLC trace for (*R*)-10-Chloro-8-(4-fluorophenyl)-1*H*-benzo[1,2] fluoreno[3,4-*c*]furan-3(8*H*)-one (4k)

Conditions: hexane/2-propanol = 96:04

Flow rate = 1.0 mL/min, λ = 272 nm, Chiral IA-3

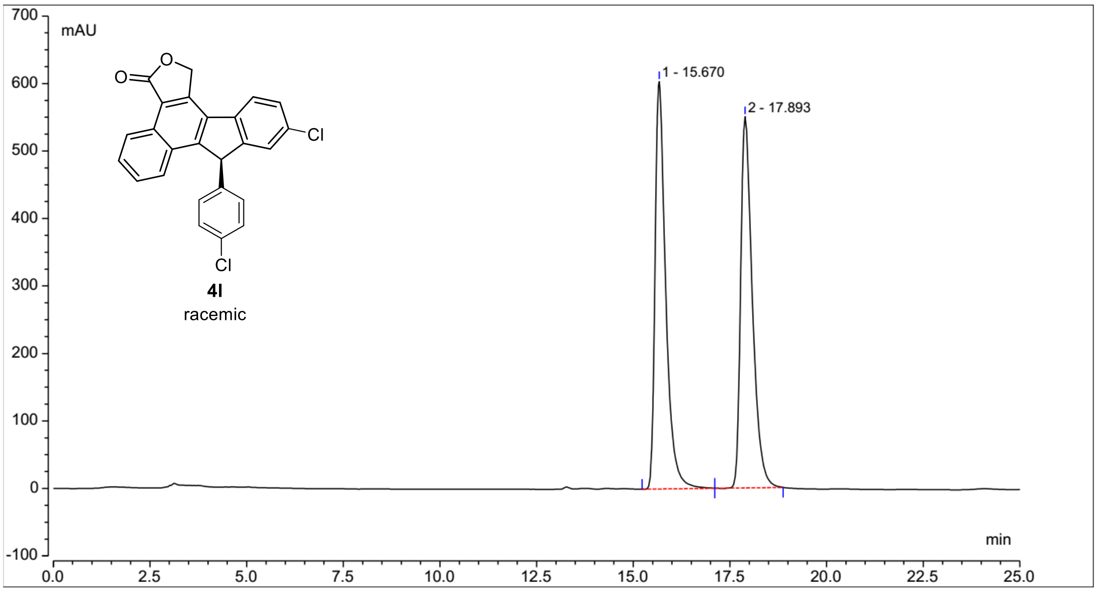

Entry	RT<br>min	Area<br>mAU*min	Height<br>mAU	% Area<br>%	% Height<br>%
1	15.670	193.235	603.753	50.09	52.32
2	17.893	192.524	550.118	49.91	47.68

Supplementary Figure 121. HPLC trace for (*R*)-10-Chloro-8-(4-chlorophenyl)-1*H*-benzo[1,2] fluoreno[3,4-*c*]furan-3(8*H*)-one e (*rac*-4l)

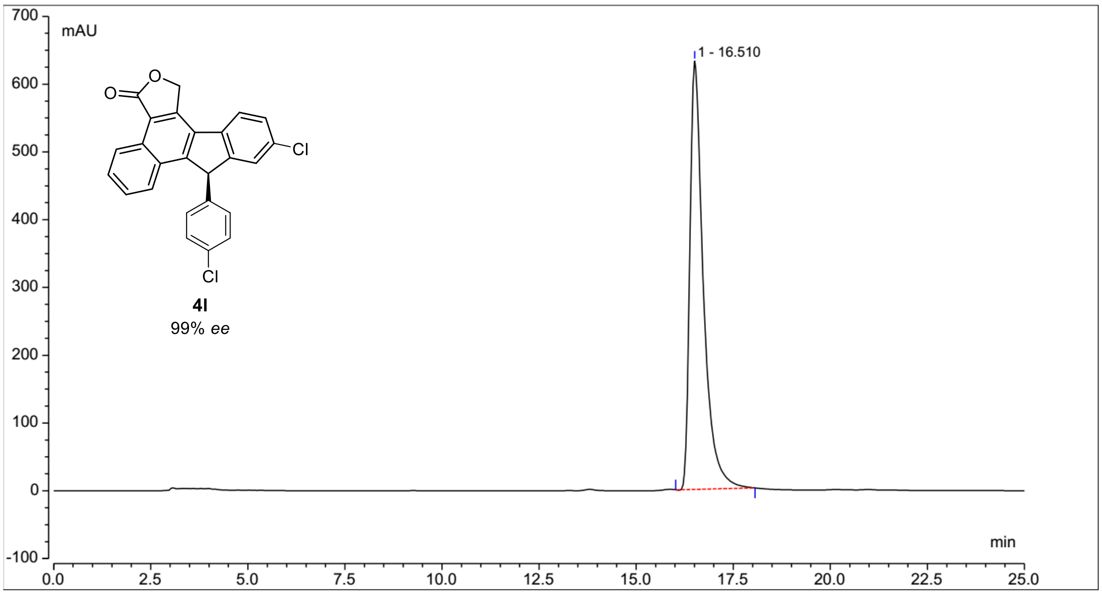

Entry	RT<br>min	Area<br>mAU*min	Height<br>mAU	% Area<br>%	% Height<br>%
1	16.510	255.614	631.931	100.00	100.00

Supplementary Figure 122. HPLC trace for (*R*)-10-Chloro-8-(4-chlorophenyl)-1*H*-benzo[1,2] fluoreno[3,4-*c*]furan-3(8*H*)-one (4l)

Conditions: hexane/2-propanol = 96:04

Flow rate = 1.0 mL/min, λ = 272 nm, Chiral IA-3

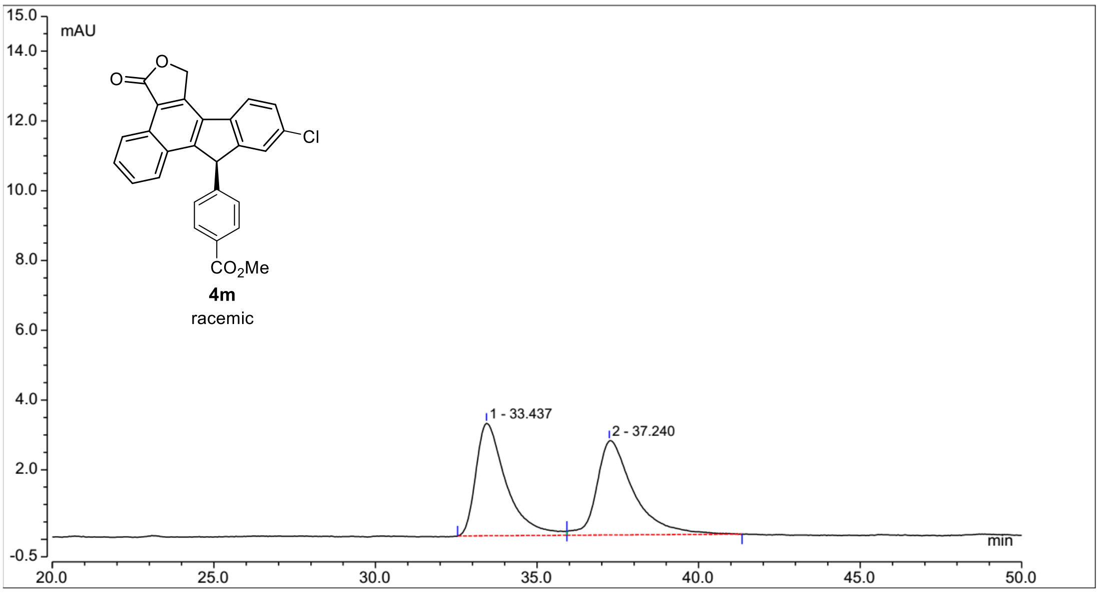

Entry	RT min	Area mAU*min	Height mAU	% Area %	% Height %
1	33.437	3.505	3.223	49.53	54.38
2	37.240	3.572	2.703	50.47	45.62

Supplementary Figure 123. HPLC trace for (*R*)-Methyl-4-(10-chloro-3-oxo-3,8-dihydro-1*H*-benzo[1,2]fluoreno[3,4-*c*]furan-8-yl)benzoate(*rac*-4m)

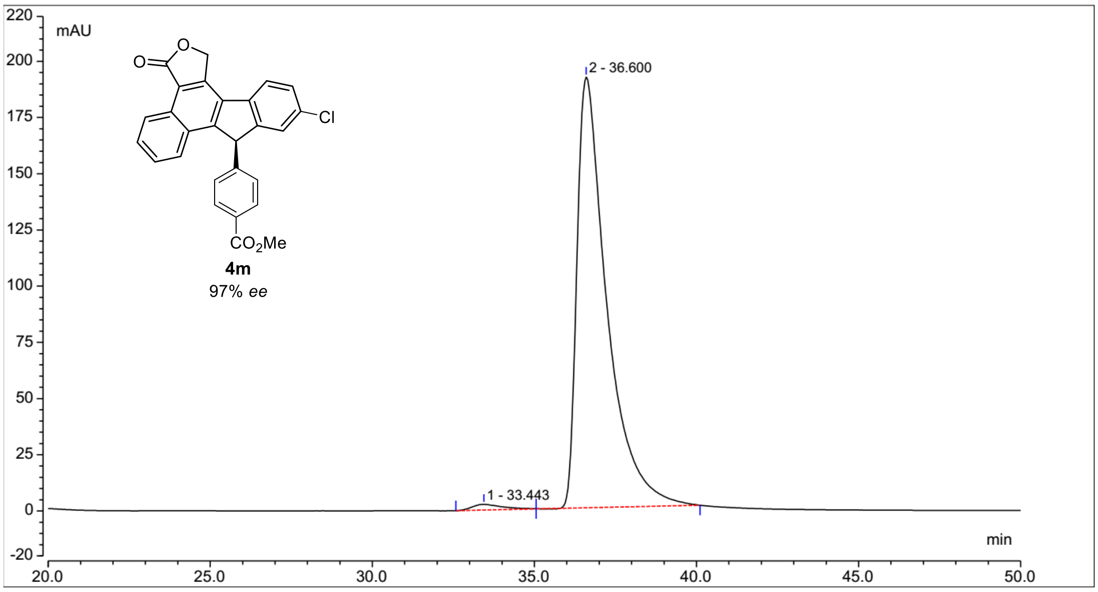

Entry	RT min	Area mAU*min	Height mAU	% Area %	% Height %
1	33.443	2.719	2.510	1.31	1.29
2	36.600	204.612	191.614	98.69	98.71

Supplementary Figure 124. HPLC trace for (*R*)-Methyl-4-(10-chloro-3-oxo-3,8-dihydro-1*H*-benzo[1,2]fluoreno[3,4-*c*]furan-8-yl)benzoate (4m)

Conditions: hexane/2-propanol /ethanol= 98.5:0.75:0.75

Flow rate = 1.0 mL/min, λ = 272 nm, Chiral IB-3

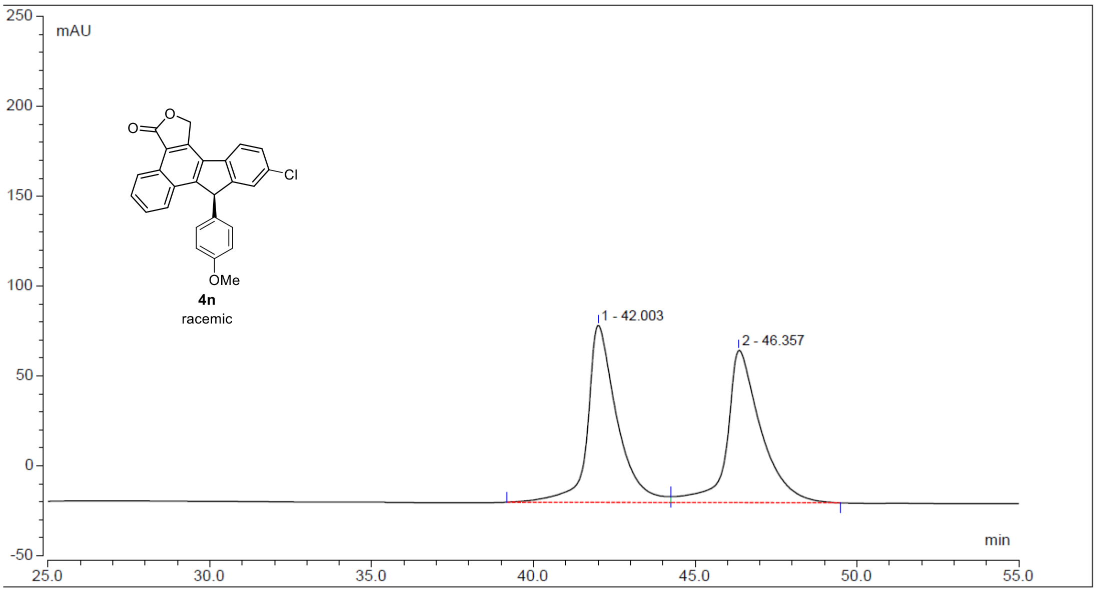

Entry	RT min	Area mAU*min	Height mAU	% Area %	% Height %
1	42.003	104.195	98.566	50.20	53.77
2	46.357	103.366	84.754	49.80	46.23

Supplementary Figure 125. HPLC trace for (*R*)-10-Chloro-8-(4-methoxyphenyl)-1*H*-benzo[1,2]fluoreno[3,4-*c*]furan-3(*8H*)-one (*rac*-4n)

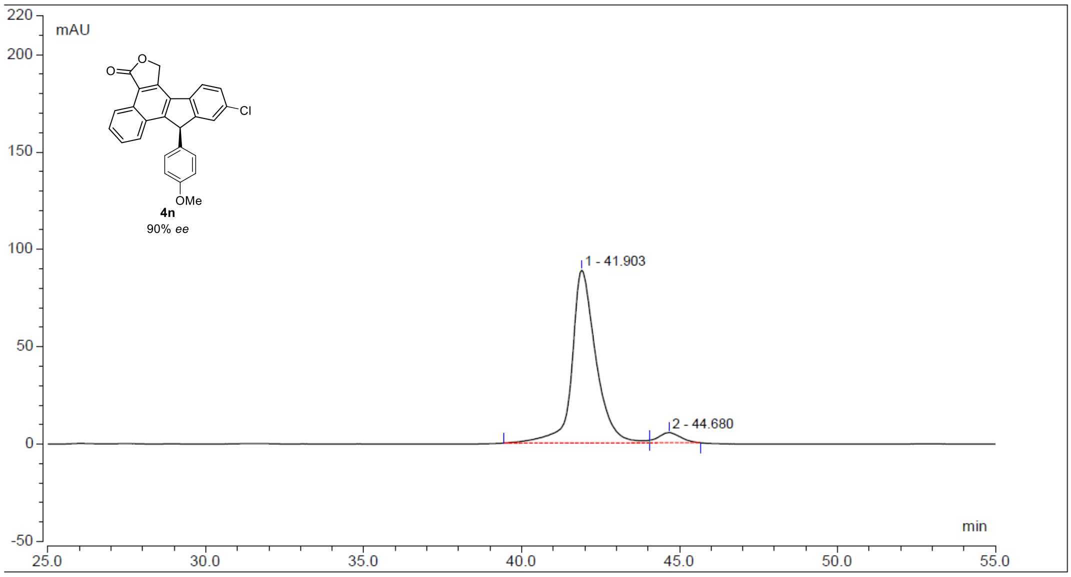

Entry	RT min	Area mAU*min	Height mAU	% Area %	% Height %
1	41.903	78.326	88.662	94.89	94.47
2	44.680	4.222	5.193	5.11	5.53

Supplementary Figure 126. HPLC trace for (*R*)-10-Chloro-8-(4-methoxyphenyl)-1*H*-benzo[1,2]fluoreno[3,4-*c*]furan-3(*8H*)-one (4n)

Conditions: hexane/2-propanol = 98:1:1

Flow rate = 1.0 mL/min, λ = 272 nm, Chiral IA-3

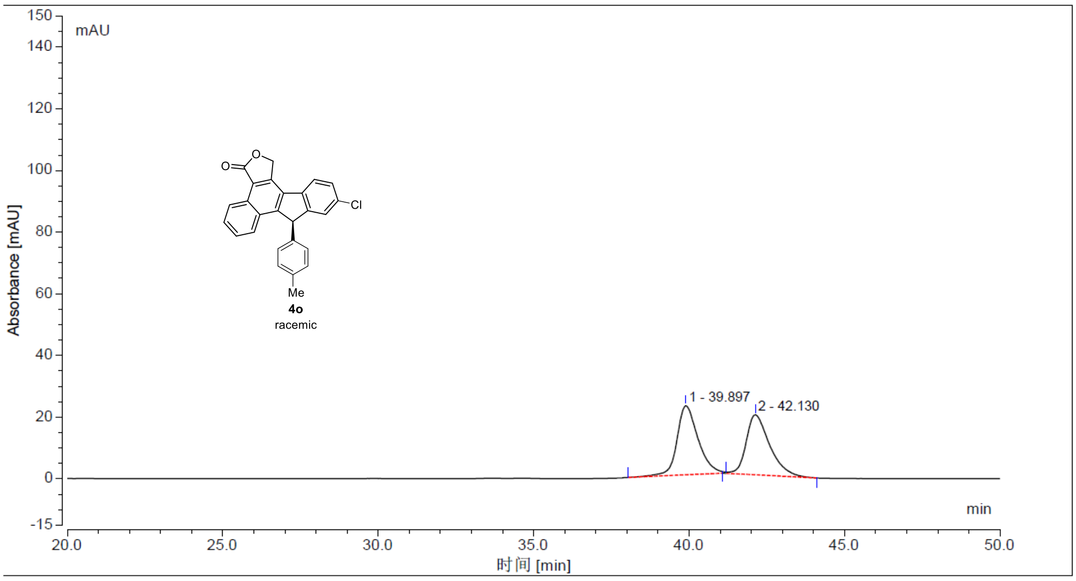

Entry	RT<br>min	Area<br>mAU*min	Height<br>mAU	% Area<br>%	% Height<br>%
1	39.897	17.585	22.390	50.78	53.45
2	42.130	17.046	19.497	49.22	46.55

Supplementary Figure 127. HPLC trace for (*R*)-10-Chloro-8-(*p*-tolyl)-1*H*-benzo[1,2]fluoreno [3,4-*c*]furan-3(8*H*)-one (*rac*-4o)

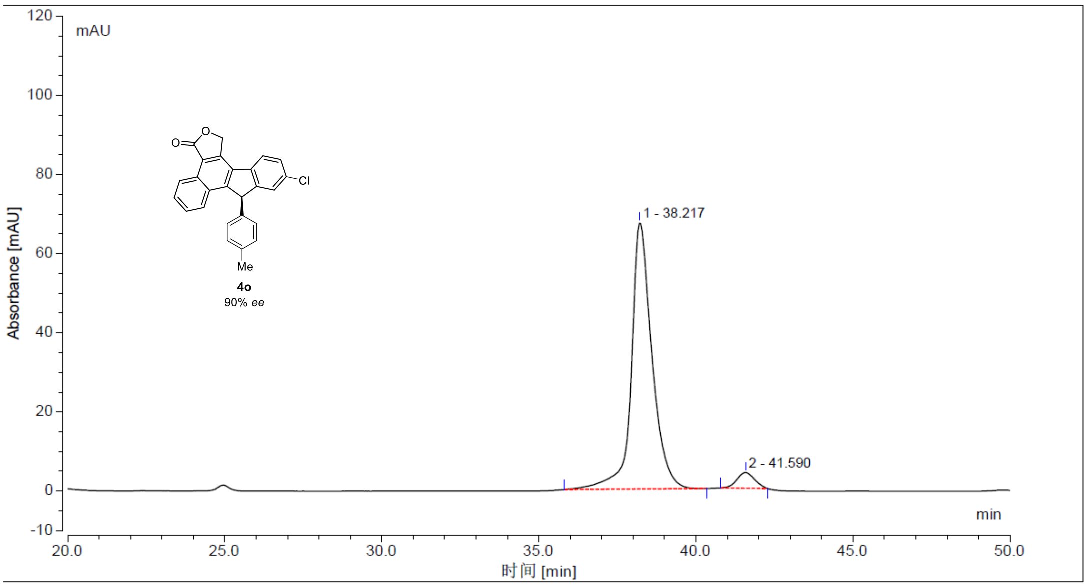

Entry	RT<br>min	Area<br>mAU*min	Height<br>mAU	% Area<br>%	% Height<br>%
1	38.217	51.350	67.370	95.19	94.41
2	41.590	2.595	3.986	4.81	5.59

Supplementary Figure 128. HPLC trace for (*R*)-10-Chloro-8-(*p*-tolyl)-1*H*-benzo[1,2]fluoreno [3,4-*c*]furan-3(8*H*)-one (4o)

Conditions: hexane/2-propanol = 93:07

Flow rate = 1.0 mL/min, λ = 272 nm, Chiral IA-3

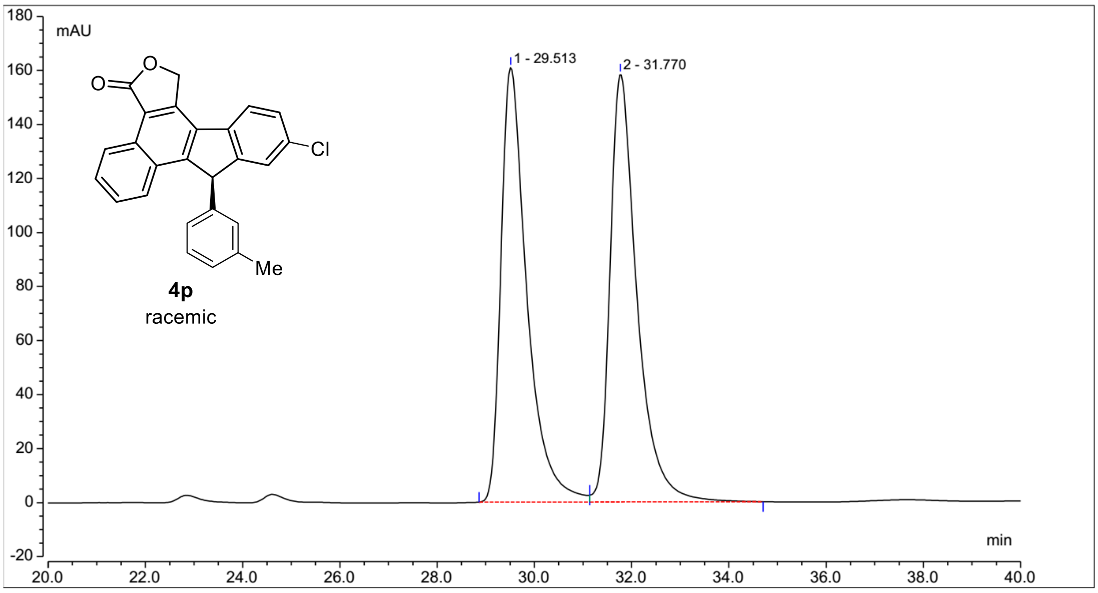

Entry	RT<br>min	Area<br>mAU*min	Height<br>mAU	% Area<br>%	% Height<br>%
1	29.513	99.704	160.936	49.51	50.39
2	31.770	101.661	158.438	50.49	49.61

Supplementary Figure 129. HPLC trace for (*R*)-10-Chloro-8-(*m*-tolyl)-1*H*-benzo[1,2] fluoreno[3,4-*c*]furan-3(8*H*)-one (*rac*-4p)

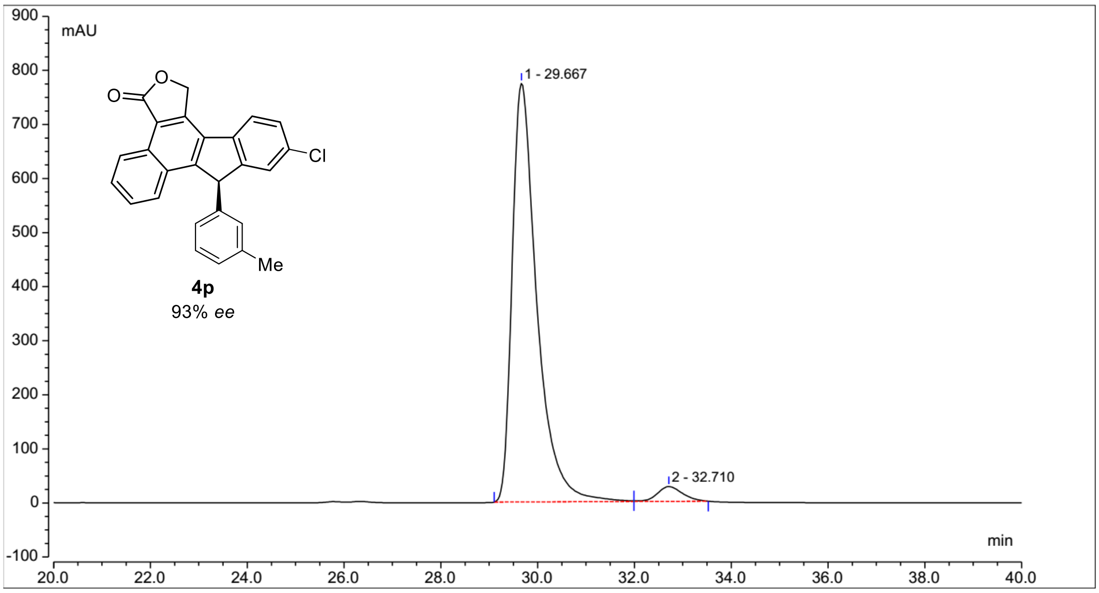

Entry	RT<br>min	Area<br>mAU*min	Height<br>mAU	% Area<br>%	% Height<br>%
1	29.667	457.605	774.713	96.51	96.59
2	32.710	16.567	27.386	3.49	3.41

Supplementary Figure 130. HPLC trace for (*R*)-10-Chloro-8-(*m*-tolyl)-1*H*-benzo[1,2] fluoreno[3,4-*c*]furan-3(8*H*)-one (4p)

Conditions: hexane/2-propanol = 92:08

Flow rate = 1.0 mL/min, λ = 272 nm, Chiral IA-3

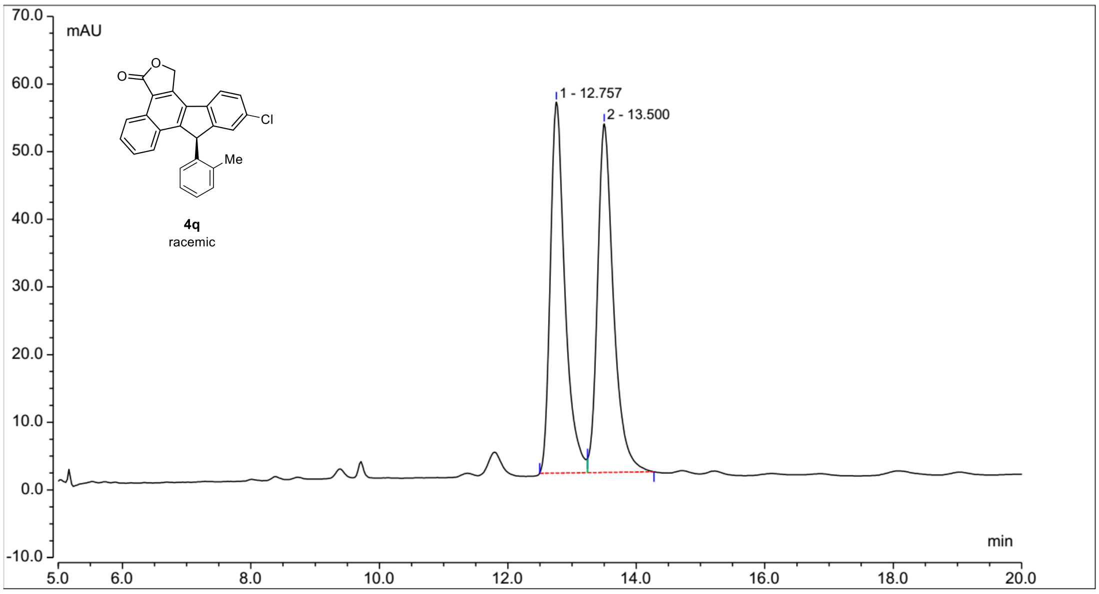

Entry	RT min	Area mAU*min	Height mAU	% Area %	% Height %
1	12.757	14.503	54.827	49.40	51.56
2	13.500	14.854	51.505	50.60	48.44

Supplementary Figure 131. HPLC trace for (*R*)-10-Chloro-8-(*o*-tolyl)-1*H*-benzo[1,2] fluoreno[3,4-c]furan-3(8*H*)-one (*rac*-4q)

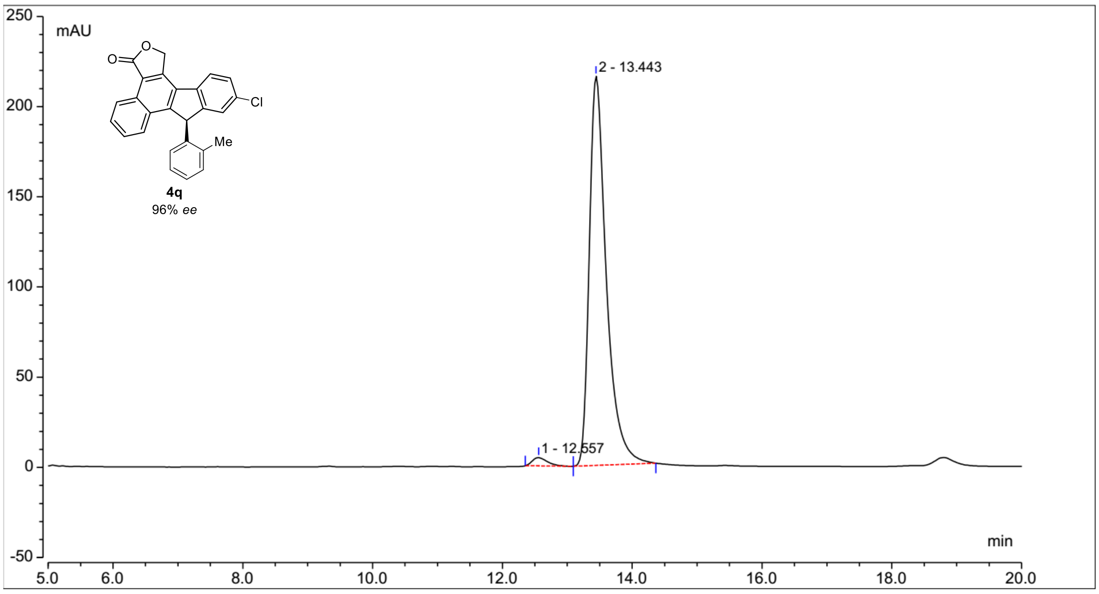

Entry	RT min	Area mAU*min	Height mAU	% Area %	% Height %
1	12.557	1.237	4.575	1.85	2.08
2	13.443	65.467	215.780	98.15	97.92

Supplementary Figure 132. HPLC trace for (*R*)-10-Chloro-8-(*o*-tolyl)-1*H*-benzo [1,2]fluoreno[3,4-c]furan-3(8*H*)-one (4q)

Conditions: hexane/2-propanol/ethanol=98.5:0.75:0.75

Flow rate = 1.0 mL/min, λ = 272 nm, Chiral IB-3

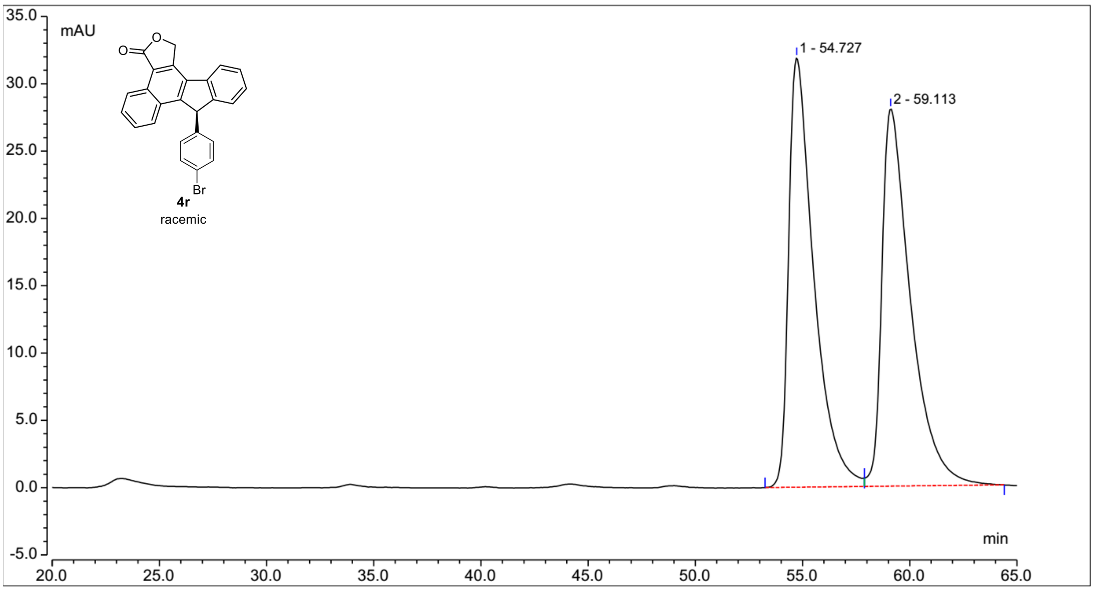

Entry	RT min	Area mAU*min	Height mAU	% Area %	% Height %
1	54.727	43.470	31.906	49.96	53.24
2	59.113	43.533	28.027	50.04	46.76

Supplementary Figure 133. HPLC trace for (*R*)-8-(4-Bromophenyl)-1*H*-benzo[1,2]fluoreno[3,4-*c*]furan-3(8*H*)-one (*rac*-4r)

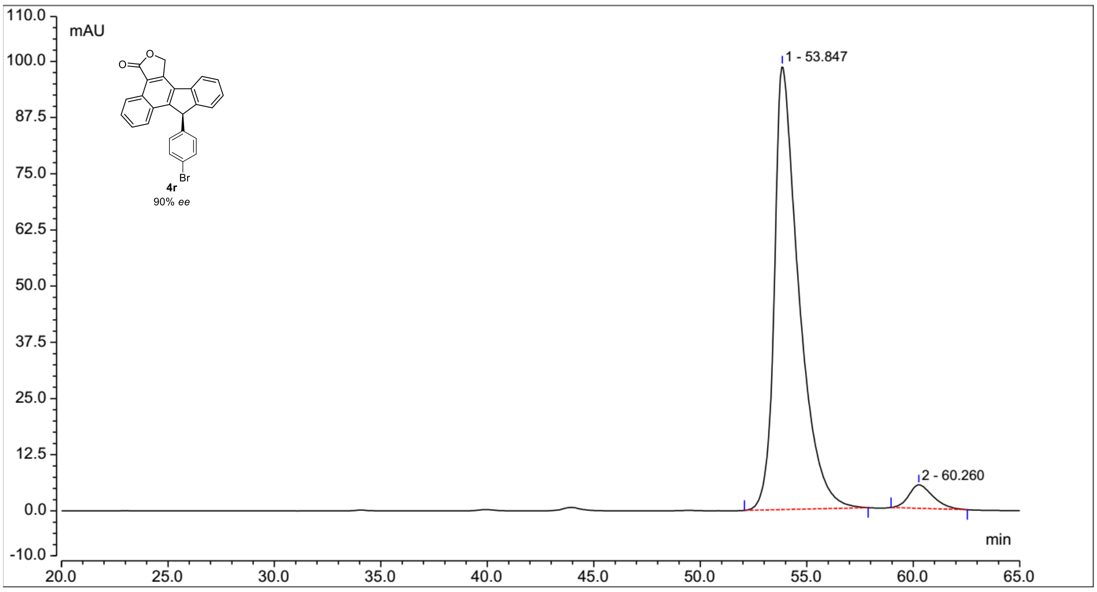

Entry	RT min	Area mAU*min	Height mAU	% Area %	% Height %
1	53.847	131.100	98.664	95.01	94.99
2	60.260	6.881	5.209	4.99	5.01

Supplementary Figure 134. HPLC trace for (*R*)-8-(4-Bromophenyl)-1*H*-benzo[1,2]fluoreno[3,4-*c*]furan-3(8*H*)-one (4r)

Conditions: hexane/2-propanol/ethanol= 92:08

Flow rate = 1.0 mL/min, λ = 272 nm, Chiral IA-3

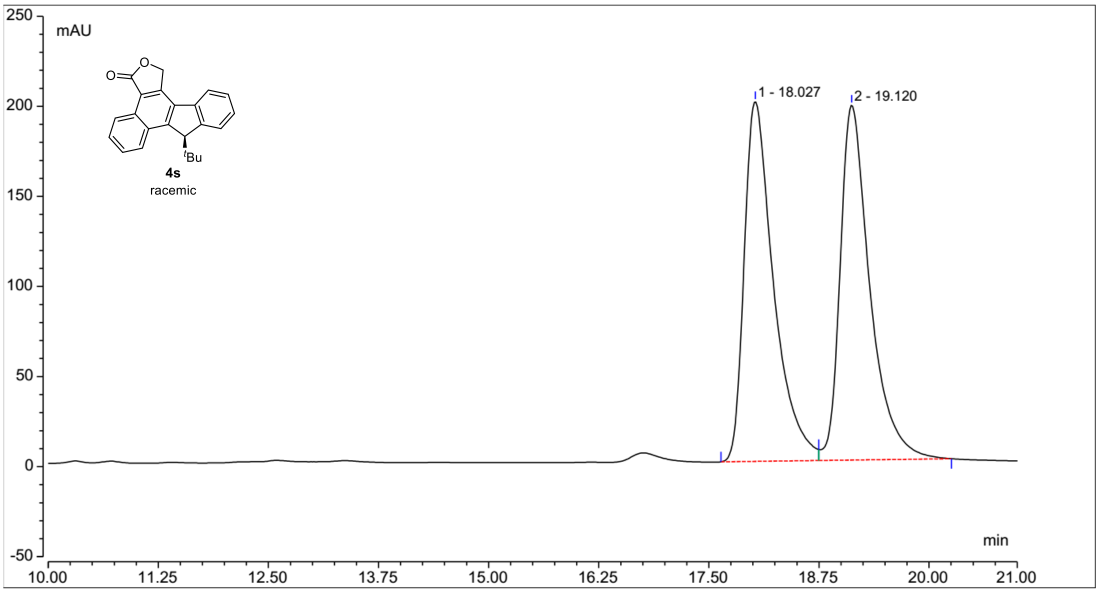

Entry	RT<br>min	Area<br>mAU*min	Height<br>mAU	% Area<br>%	% Height<br>%
1	18.027	77.129	199.738	49.99	50.34
2	19.120	77.167	197.007	50.01	49.66

Supplementary Figure 135. HPLC trace for 8-(*tert*-Butyl)-10-chloro-1*H*-benzo[1,2] fluoreno[3,4-c]furan-3(8*H*)-one (*rac*-4s)

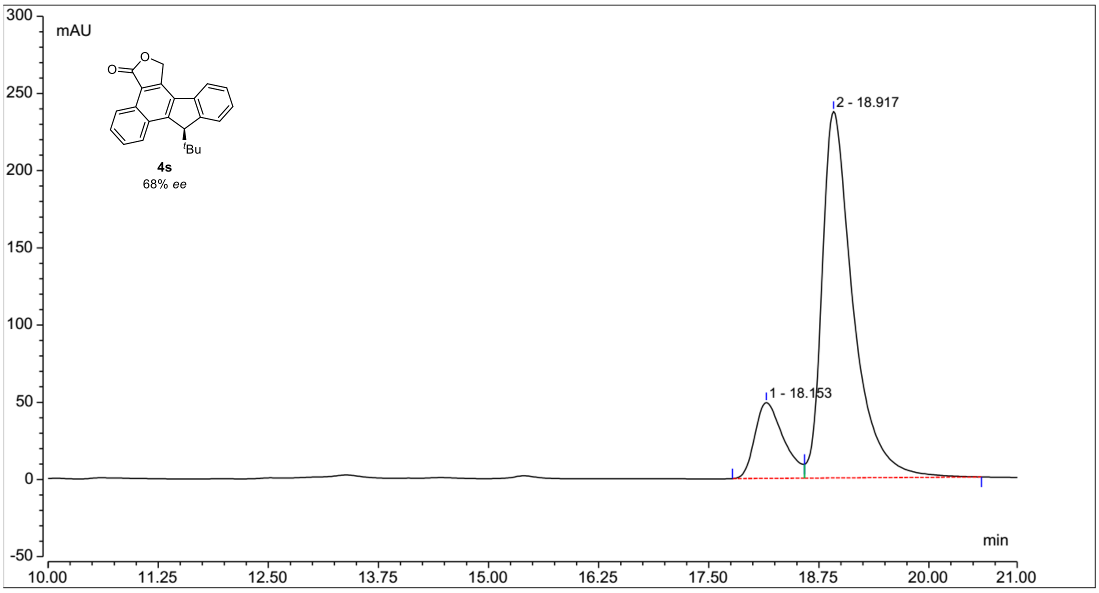

Entry	RT<br>min	Area<br>mAU*min	Height<br>mAU	% Area<br>%	% Height<br>%
1	18.153	18.540	49.111	16.09	17.14
2	18.917	96.697	237.409	83.91	82.86

Supplementary Figure 136. HPLC trace for (*R*)-8-(*tert*-Butyl)-10-chloro-1*H*-benzo[1,2] fluoreno[3,4-c]furan-3(8*H*)-one (4s)

Conditions: hexane/2-propanol = 91:09

Flow rate = 1.0 mL/min, λ = 272 nm, Chiral IA-3

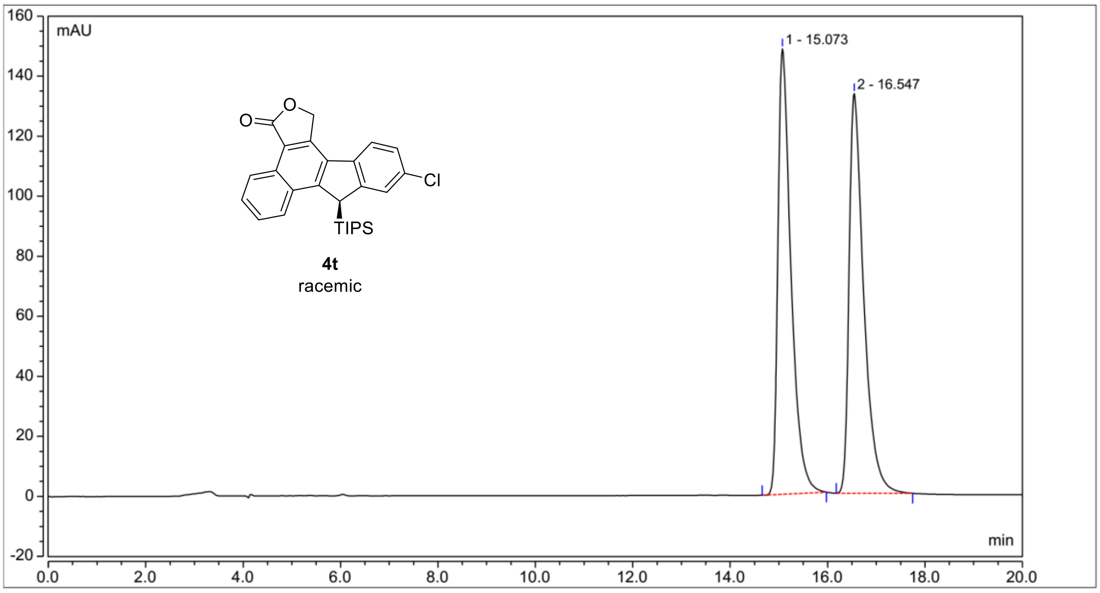

Entry	RT min	Area mAU*min	Height mAU	% Area %	% Height %
1	15.073	46.192	148.434	49.90	52.71
2	16.547	46.373	133.154	50.10	47.29

Supplementary Figure 137. HPLC trace for 10-Chloro-8-(triisopropylsilyl)-1*H*-benzo[1,2] fluoreno[3,4-c]furan-3(8*H*)-one (*rac*-4t)

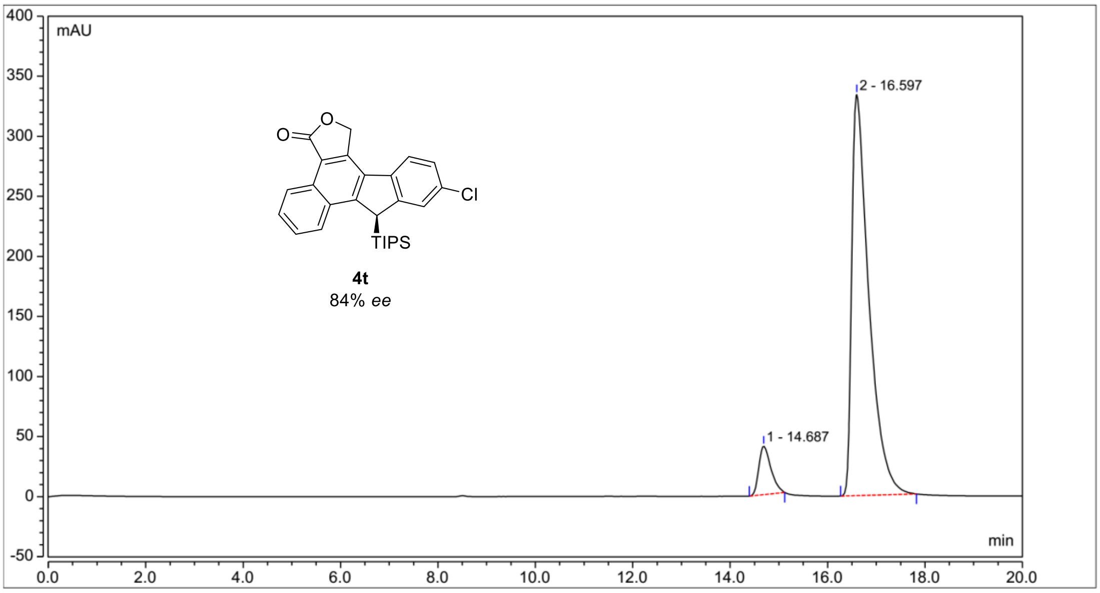

Entry	RT min	Area mAU*min	Height mAU	% Area %	% Height %
1	14.687	11.609	40.394	7.87	10.80
2	16.597	135.928	333.789	92.13	89.20

Supplementary Figure 138. HPLC trace for (*R*)-10-Chloro-8-(triisopropylsilyl)-1*H*-benzo[1,2] fluoreno[3,4-c]furan-3(8*H*)-one (4t)

Stability Study of 4c (Solid stored at 0 ºC)

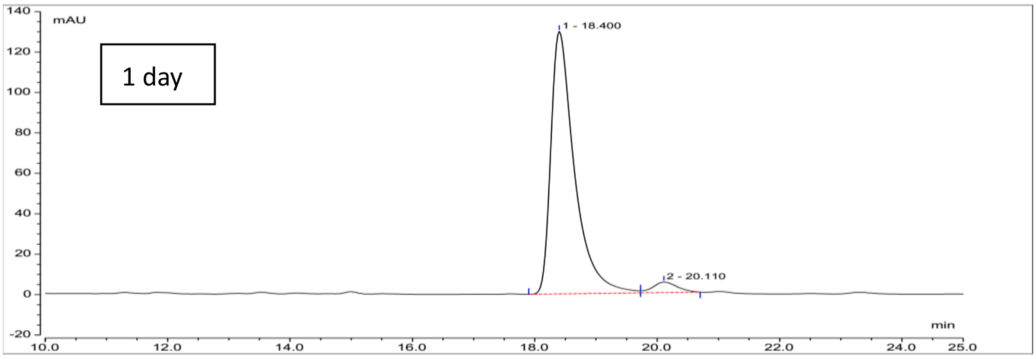

Entry	RT min	Area mAU*min	Height mAU	% Area %	% Height %
1	18.400	57.907	129.849	96.00	96.15
2	20.110	2.414	5.202	4.00	3.85

Supplementary Figure 139. HPLC trace for (*R*)-10-Bromo-8-phenyl-1*H*-benzo[1,2] fluoreno[3,4-c]furan-3(8*H*)-one (4c) stored at 0 ºC for 1 day

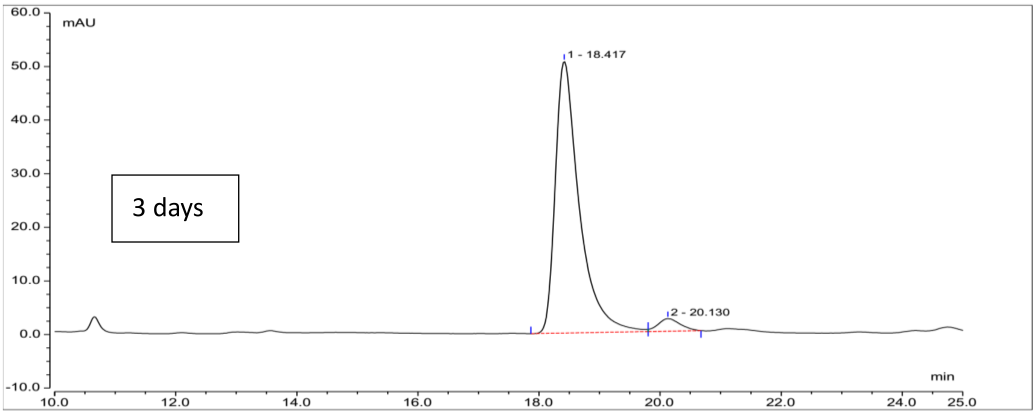

Entry	RT min	Area mAU*min	Height mAU	% Area %	% Height %
1	18.417	23.275	50.709	95.87	95.57
2	20.130	1.002	2.350	4.13	4.43

Supplementary Figure 140. HPLC trace for (*R*)-10-Bromo-8-phenyl-1*H*-benzo[1,2] fluoreno[3,4-c]furan-3(8*H*)-one (4c) stored at 0 ºC for 3 days

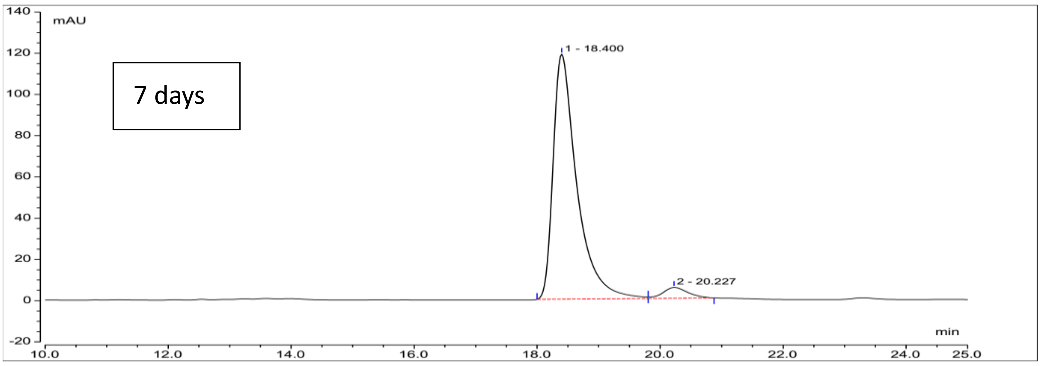

Entry	RT min	Area mAU*min	Height mAU	% Area %	% Height %
1	18.400	53.041	118.815	95.53	95.77
2	20.227	2.484	5.250	4.47	4.23

Supplementary Figure 141. HPLC trace for (*R*)-10-Bromo-8-phenyl-1*H*-benzo[1,2] fluoreno[3,4-c]furan-3(8*H*)-one (4c) stored at 0 ºC for 7 day

Crystallographic Data for 4b

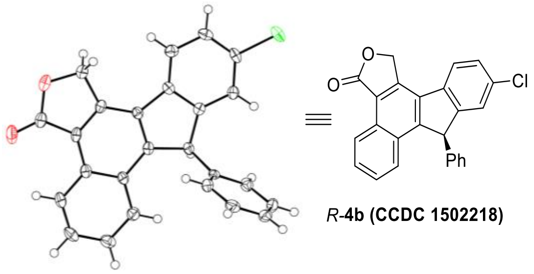

Bond precision: C-C = 0.0039 A Wavelength=0.71073

Cell: a=6.2562(16) b=7.8851(19) c=9.446(2)
alpha=77.776(8) beta=76.728(8) gamma=80.556(8)

Temperature: 296 K

	Calculated	Reported
Volume	439.97(18)	439.96(19)
Space group	P 1	P 1
Hall group	P 1	P 1
Moiety formula	C25 H15 Cl O2	C25 H15 Cl O2
Sum formula	C25 H15 Cl O2	C25 H15 Cl O2
Mr	382.82	382.82
Dx,g cm-3	1.445	1.445
Z	1	1
Mu (mm-1)	0.236	0.236
F000	198.0	198.0
F000'	198.23	
h,k,lmax	7,9,11	7,9,11
Nref	3588[1794]	3386
Tmin,Tmax	0.956,0.972	0.954,0.972
Tmin'	0.954	

Correction method= # Reported T Limits: Tmin=0.954 Tmax=0.972
AbsCorr = NONE

Data completeness= 1.89/0.94 Theta(max)= 26.330

R(reflections)= 0.0381(3043) wR2(reflections)= 0.0887(3386)

S = 1.051 Npar= 253

Supplementary Figure 142. Crystallographic Data for 4b

Computational Study

a) Computational details.

All DFT calculations were performed with the Gaussian 09 software package.[10] For racemic reaction, geometry optimizations of all the minima and transition states involved were carried out using the pure functional PBE.[11,12] The SDD basis set[13] (Stuttgart/Dresden ECP) was used for rhodium and the 6-31G(d) basis set[14] for the other atoms. Frequency calculations at the same level were performed to validate each structure as either a minimum or a transition state and to evaluate its zero-point energy and thermal corrections at 298 K. Solvation free energy of all the minima and transition states involved were carried out using SMD solvation model[15] in CH_2Cl_2. Based on the optimized structures, single-point energy calculations were carried out at the M06L[16]/6-311G(d,p)[14]&SDD level. Minimum-energy crossing points (MECPs) between singlet−triplet potential energy surfaces (PESs) were located with the MECP program developed by Harvey and co-workers.[17] The keyword "5D" was used to specify that five d-type orbitals were used for all elements in the calculations. For enantioselectivity calculations, geometry optimizations of all the minima and transition states involved were carried out using the pure functional PBE together with Grimme's D3 correction.[18] The Def2-SVP[19] and corresponding fitting basis set W06[20] was used for geometry optimizations. Frequency calculations at the same level were performed to validate each structure as either a minimum or a transition state and to evaluate its zero-point energy and thermal corrections at 298 K. Based on the optimized structures, single-point energy calculations were carried out at the SMD(toluene)-M06L-D3[18]/Def2-TZVP[19] level. We used standard state of 1.0 mol/L at 298 K for all species and therefore a 1.89 kcal/mol correction was used for processes involving two molecules to a complex/intermediate or a transition state or vice versa. All discussed energies were Gibbs free energies unless otherwise specified. 3D structure was prepared with CYLview.[21]

b) Computed energies for the stationary points

Supplementary Table 2 Thermal corrections to Gibbs energies (TCGs) and single-point energies (SPEs) of asymmetric reaction 1.

	**TCG[a] (a.u.)**	**SPE[b] (a.u.)**
TS-*S*	1.009466	-6342.535193
TS-*R*	1.008674	-6342.530138

[a]Computed at the PBE-D3/Def2-SVP/W06 level.

[b]Computed at the SMD(toluene)-M06L-D3/Def2-TZVP level.

Supplementary Table 3, Thermal corrections to Gibbs energies (TCGs) and single-point energies (SPEs) of racemic tandem reaction 2.

	TCG[a] (a.u.)	SPE[b] (a.u.)
N_2	-0.01309	-109.5365303
3a	0.269452	-1222.269567
$Rh_2(HCOO)_4$	0.057691	-978.1930657
4a	0.279876	-1112.943758
IN1	0.349185	-2200.477255
IN2	0.344224	-2090.948716
IN3	0.34807	-2090.966361
^{3}IN3	0.345651	-2090.96155
IN4	0.350272	-2091.017276
IN5	0.35233	-2091.026607
^{3}IN5	0.350947	-2091.042874
IN6	0.357107	-2091.089551
TS1	0.347239	-2200.460962
TS2	0.346035	-2090.942823
TS3	0.346459	-2090.947218
^{3}TS3	0.346365	-2090.963745
TS4	0.352621	-2091.003573
TS5	0.352387	-2091.029307
^{3}TS5	0.351058	-2091.041684
TS6	0.355555	-2091.053977

[a]Computed at the PBE/6-31G(d)&SDD level.

[b]Computed at the SMD(DCM)-M06L/6-311G(d,p)&SDD level.

Supplementary Table 4 Single-point energies (SPEs) of MECP

	SPE[a] (a.u.)
IN3	-2088.431784
MECP-1	-2088.424028
IN5	-2088.498593
MECP-2	-2088.498507

[a]Computed at the PBE/6-31G(d)&SDD level.

Supplementary Table 5 Thermal corrections to Gibbs energies (TCGs) and single-point energies (SPEs) of asymmetric reaction 2

	TCG[a] (a.u.)	SPE[b] (a.u.)
TS-*pro-S*	1.069216	-6623.566934
TS-*pro-R*	1.069114	-6623.564693

c-INT3	1.074701	-6514.185712
c-TS-INT3-CH	1.073512	-6514.146712
c-TS-INT3-Rotation	1.079397	-6514.131254

[a]Computed at the PBE-D3/Def2-SVP/W06 level.

[b]Computed at the SMD(toluene)-M06L-D3/Def2-TZVP level.

c) 3D structures of key intermediates for racemic reaction 2

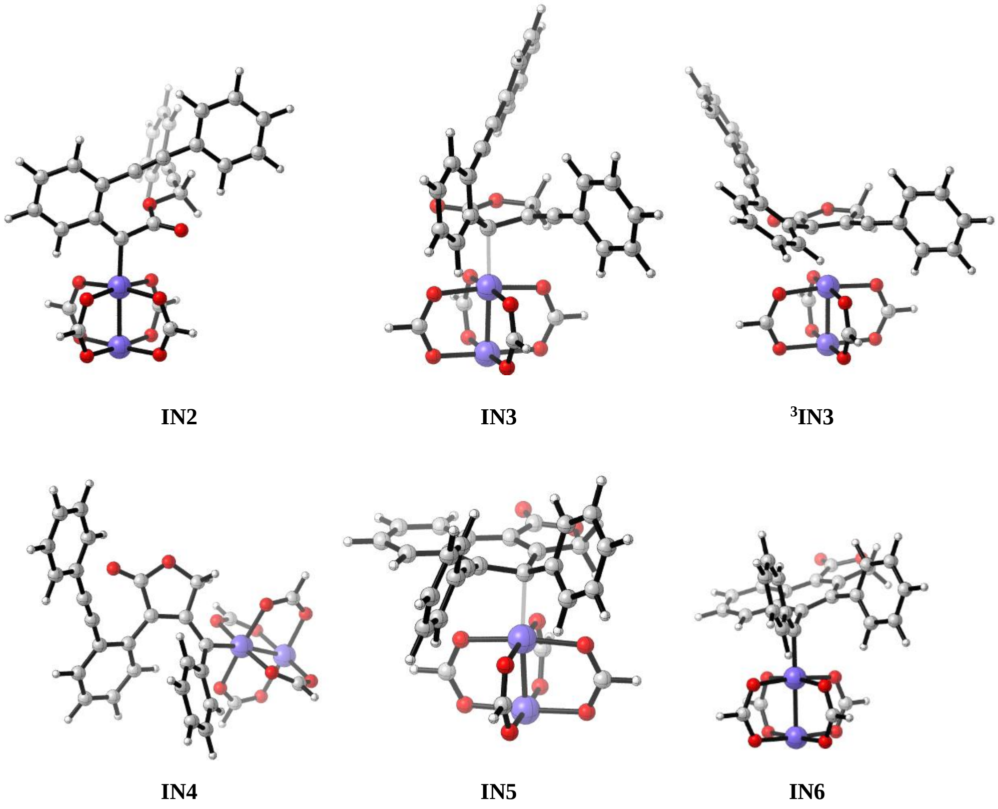

Supplementary Figure 143. 3D structures of key intermediates for reaction 2.

d) Orbital analysis of triplet intermediate ^{3}IN3

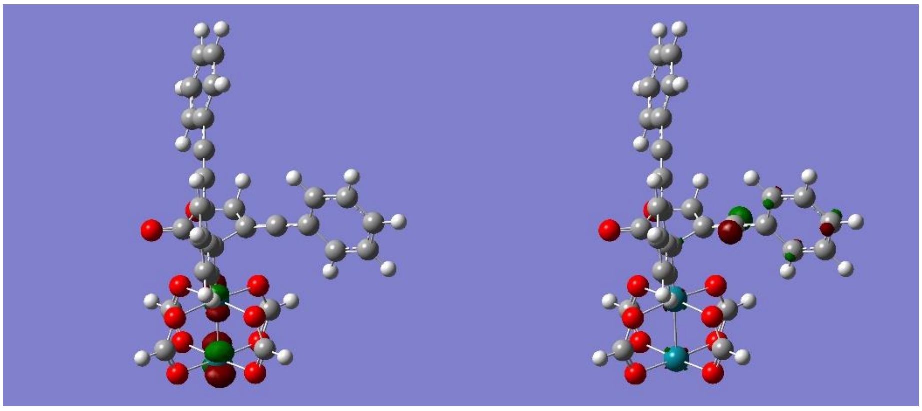

Supplementary Figure 144. HOMO and LUMO of IN3.

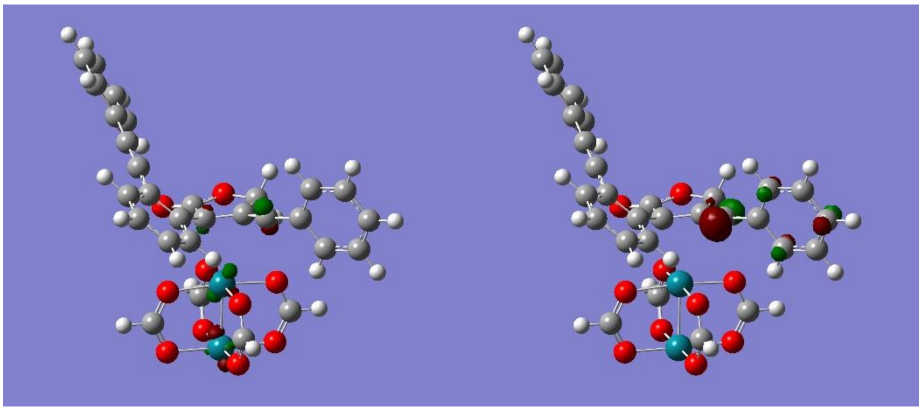

Supplementary Figure 145. SOMOs of ^{3}IN3.

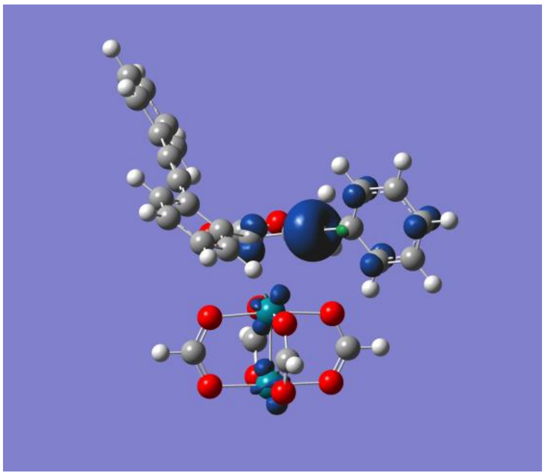

Supplementary Figure 146. Spin density of ^{3}IN3.

e) Regioselectivity discussion for reactions of substrates 1i and 3g

DFT calculation result show that for substrate **1i**, the para-position C-H insertion TS-*p*F-DDC-CH is 3.5 kcal/mol favored than the ortho-position C-H insertion TS-*o*F-DDC-CH, so only product **2i** can be obtained. For substrate **3g**, the C-H insertion barriers only differs by 0.7 kcal/mol, suggesting that a mixture of **4g** and **4g'** should be obtained. Both of these calculations agreed with experiments.

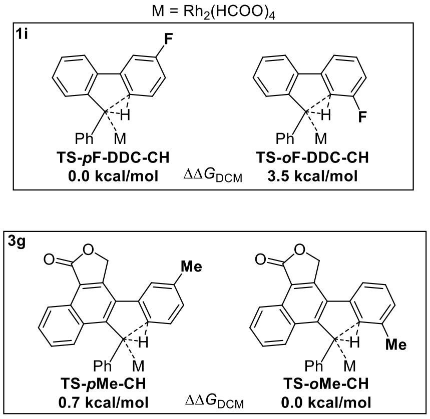

Supplementary Figure 147. Regioselectivity calculation results of substrates 1i and 3g.

Supplementary Table 6 Thermal corrections to Gibbs energies (TCGs) and single-point energies (SPEs) of regioselectivity calculation of substrates 1i and 3g

	TCG[a] (a.u.)	SPE[b] (a.u.)
TS-*p*F-DDC-CH	0.286346	-1810.160774
TS-*o*F-DDC-CH	0.286882	-1810.155733
TS-*p*Me-CH	0.379838	-2130.644121
TS-*o*Me-CH	0.381254	-2130.644393

[a]Computed at the PBE/6-31G(d)&SDD level.

[b]Computed at the SMD(toluene)-M06L/Def2-TZVP level.

f) Reactivity discussion of substrate 3h:

For the substrate 3h, a higher temperature is need for C-H insertion reaction due to the steric repulsion between the methyl group and lactone ring in the C-H insertion transition state, as suggested by DFT calculations shown below. The C-H insertion step has activation barrier about 5 kcal/mol than that for substrate 3a when we used model catalyst (we speculated that, if real catalyst was used, this energy difference should become lower about 2 to 3 kcal/mol). We hypothesized that the reaction of 3h initially gave higher *ee*, because this was set up in the first step of the catalytic cycle and there was no difference from substrate 3a. But the product could then undergo racemization at 60 °C. One support for this hypothesis is that product 4c heating at 80 °C for 12 hours underwent racemization from 90% *ee* to 30% *ee*.

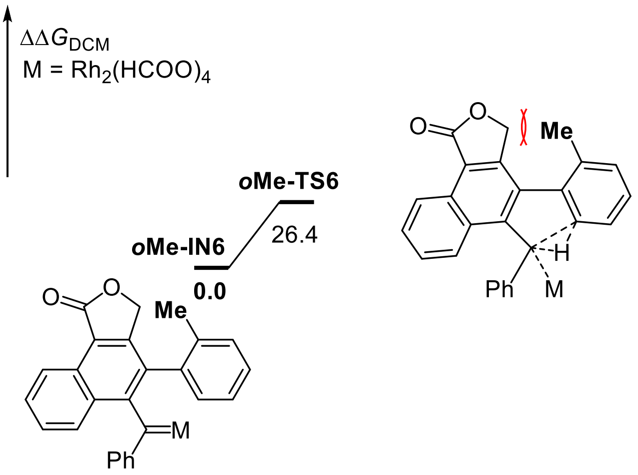

Supplementary Figure 148. C-H Insertion reaction step for substrate 3h.

Supplementary Table 7 Thermal corrections to Gibbs energies (TCGs) and single-point energies (SPEs) of C-H insertion reaction of substrate 3h

	TCG[a] (a.u.)	SPE[b] (a.u.)
*o*Me-IN6	0.38275	-2130.410753
*o*Me-TS6	0.382142	-2130.368067

[a]Computed at the PBE/6-31G(d)&SDD level.

[b]Computed at the SMD(DCM)-M06L/6-311G(d,p)&SDD level.

g) Estimation of the rotation barrier of axial chiral intermediate *c*-INT3

Considering that the C-H insertion step is the rate-determine step in this tandem reaction, we calculated the barriers of C-H insertion and rotation of *c*-INT3 in the real system (simplifying the chiral catalyst may cause underestimation of rotation barrier). The barrier of final C-H insertion reaction is 23.7 kcal/mol. The rotation barrier shown below is 13.4 kcal/mol higher than that required for the C-H insertion reaction, so the chiral transfer can be realized from the traisiant axial chirality in intermediate *c*-INT3.

In the rotation transition state structure for intermediate *c*-INT3, remarkable distortion of chiral dirhodium catalyst and substrate part can be observed due to the strong repulsion of two aryl rings, which is shown below.

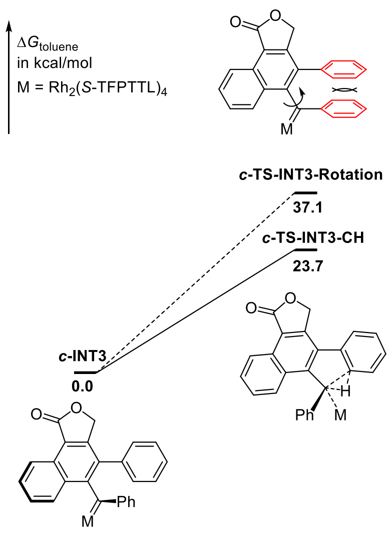

Supplementary Figure 149. Rotation vs Csp^2-H insertion.

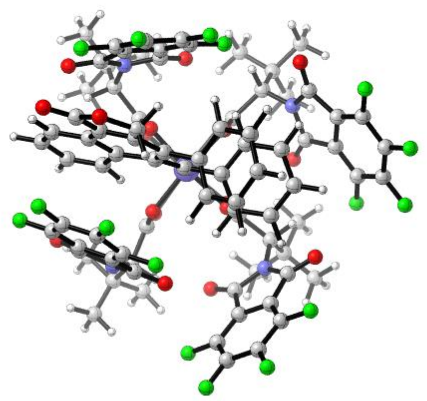

Supplementary Figure 150. 3D structure of Rotation transition state of *c*-INT3.

h) Possibility of Catalyst dissociation from intermediates

We have considered that catalyst could dissociate from some intermediates and then rebound back to form these complexes again for further transformations, which could decrease the enantioselectivity. **IN2**, **IN4**, and **IN6** are strong complexes of carbenes with catalyst, and catalyst dissociations from these intermediates are not possible. Other intermediates such as **IN3** and **IN5** are not possible too, because they can quickly be converted to **IN4** and **IN6** with almost no barriers (see Figure 3). Actually we have computed these possibilities for these two intermediates. **IN3** cannot dissociate to carbene and catalyst because our calculations by elongating Rh-C bond in **IN3** leads to **IN4**, same as the MECP process in Figure 3. For **IN5**, dissociation of catalyst is slower than MECP shown in Figure 3.

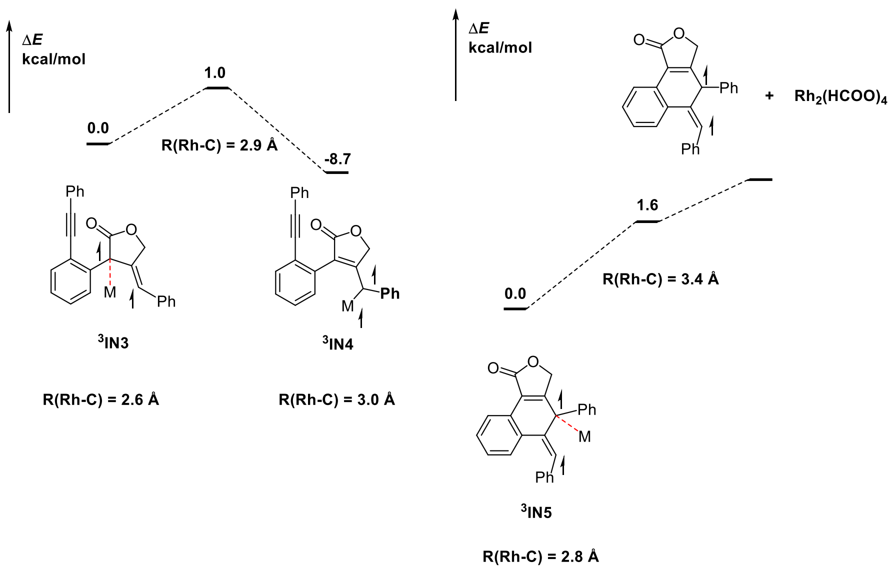

Supplementary Figure 151. Scan result of potential energy surface

i) 3D Structures of axial chiral intermediates in real system

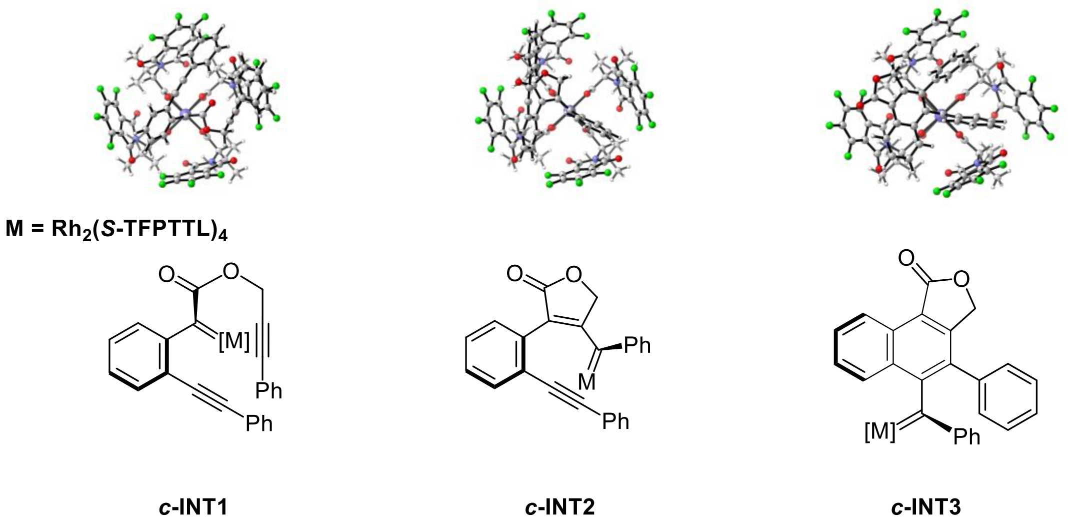

Supplementary Figure 152. 3D structures of key chiral intermediates.

Supplementary References

1. Zhao, Z., He, B., Nie, H., Chen, B., Lu, P., Qin, A. & Tang, B. Stereoselective synthesis of folded luminogens with arene–arene stacking interactions and aggregation-enhanced emission. *Chem. Commun.* **50**, 1131–1133 (2014).
2. Ageshina, A., Chesnokov, G., Topchiy, M., Alabugin, I., Nechaev M. S. & Asachenko. A. F. Making endo-cyclizations favorable again: a conceptually new synthetic approach to benzotriazoles via azide group directed lithiation/cyclization of 2-azidoaryl bromides. *Org. Biomol. Chem.* **17**, 4523–4534 (2019).
3. Gazis, T., Dasgupta, A., Hill, M., Rawson, J., Wirth. T. & Melen, R. Reactions of hydrazones and hydrazides with Lewis acidic boranes. *Dalton Trans.* **48**, 12391–12395 (2019).
4. Peng, C., Cheng, J. & Wang, J. Sequential copper(I)-catalyzed reaction of amines with *o*-acetylenyl-substituted phenyldiazoacetates. *Adv. Synth. Catal.* **350**, 2359–2364 (2008).
5. Badry, M., Kariuki, B., Knight, D. W. & Mohammed, F. K. 6-Exoversus 7-endoiodolactonizations of 2-(alkynyl)phenylacetic acids. *Tetrahedron Lett.* **50**, 1385–1388 (2009).
6. Zheng, Y., Mao, J., Weng, Y., Zhang, X. & X. Xu, Cyclopentadiene construction *via* Rh-catalyzed carbene/alkyne metathesis terminated with intramolecular formal [3 + 2] cycloaddition. *Org. Lett.* **17**, 5638–5641 (2015).
7. Li, C., Brideau, C., Chan, C., Savoie, C., Claveau, D., Charleson, S., Gordon, R., Greig, G., Gauthier, J., Lau, C. K., Riendeau, D., Terien, M., Wong, E. & Prasit, P. Pyridazinones as selective cyclooxygenase-2 inhibitors. *Bioorg. Med. Chem Lett.* **13**, 597–600 (2003).
8. Meng, C., Ni, L., Worsencroft, K. J., Ye, Z., Weingarten, M. D., Simpson, J. E., Skudlarek, J. W., Marino, E. M., Suen, K., Kunsch, C., Souder, A., Howard, R. B., Sundell, C. L., Wasserman, M. A. & Sikorski, J. A. Carboxylated, heteroaryl-substituted chalcones as inhibitors of vascular cell adhesion molecule-1 expression for use in chronic inflammatory diseases. *J. Med. Chem.* **50**, 1304–1315 (2007).
9. Yao, R., Rong, G., Yan, B., Qiu, L. & Xu, X. Dual-Functionalization of alkynes *via* copper-catalyzed carbene/alkyne metathesis: a direct access to the 4-carboxyl quinolines. *ACS Catal.* **6**, 1024–1027 (2016).
10. Frisch, M. J.; Trucks, G. W.; Schlegel, H. B.; Scuseria, G. E.; Robb, M. A.; Cheeseman, J. R.; Scalmani, G.; Barone, V.; Mennucci, B.; Petersson, G. A.; Nakatsuji, H.; Caricato, M.; Li, X.; Hratchian, H. P.; Izmaylov, A. F.; Bloino, J.; Zheng, G.; Sonnenberg, J. L.; Hada, M.; Ehara, M.; Toyota, K.; Fukuda, R.; Hasegawa, J.; Ishida, M.; Nakajima, T.; Honda, Y.; Kitao, O.; Nakai, H.; Vreven, T.; Montgomery, J. A., Jr.; Peralta, J. E.; Ogliaro, F.; Bearpark, M.; Heyd, J. J.; Brothers, E.; Kudin, K. N.; Staroverov, V. N.; Kobayashi, R.; Normand, J.; Raghavachari, K.; Rendell, A.; Burant, J. C.; Iyengar, S. S.; Tomasi, J.; Cossi, M.; Rega, N.; Millam, J. M.; Klene, M.; Knox, J. E.; Cross, J. B.; Bakken, V.; Adamo, C.; Jaramillo, J.; Gomperts, R.; Stratmann, R. E.; Yazyev, O.; Austin, A. J.; Cammi, R.; Pomelli, C.; Ochterski, J. W.; Martin, R. L.; Morokuma, K.; Zakrzewski, V. G.; Voth, G. A.; Salvador, P.; Dannenberg, J. J.; Dapprich, S.; Daniels, A. D.; Farkas, Ö.; Foresman, J. B.; Ortiz, J. V.; Cioslowski, J.; Fox, D. J. *Gaussian 09*, *Revision D.01*; Gaussian, Inc.: Wallingford, CT, 2009.
11. Perdew, J. P., Burke, K. & Ernzerhof, M. Generalized gradient approximation made simple. *Phys. Rev. Lett.* **77**, 3865–3868 (1996).

12. Perdew, J. P., Burke, K. & Ernzerhof, M. Generalized gradient approximation made simple. *Phys. Rev. Lett.* **78**, 1396 (1997).
13. Andrae, D., Haeussermann, U., Dolg, M., Stoll, H. & Preuss, H. Energy−adjusted *ab initio* pseudopotentials for the second and third row transition elements. *Theor. Chem. Acc*. **77**, 123–141 (1990).
14. Hehre, W. J., Radom, L., Schleyer, P. R. & J. A. Pople, Ab Initio molecular orbital theory; Wiley: New York, pp. 379 (1986).
15. Marenich, A. V., Cramer, C. J. & Truhlar, D. G. Universal solvation model based on solute electron density and a continuum model of the solvent defined by the bulk dielectric constant and atomic surface tensions. *J. Phys. Chem. B.* **113**, 6378−6396 (2009).
16. Zhao, Y. & Truhlar, D. G. A new local density functional for main−group thermochemistry, transition metal bonding, thermochemical kinetics, and noncovalent interactions. *J. Chem. Phys*. **125**, 194101−194118 (2006).
17. Harvey, J. N., Aschi, M., Schwarz, H. & Koch, W. The singlet and triplet states of phenyl cation. A hybrid approach for locating minimum energy crossing points between non−interacting potential energy surfaces. *Theor. Chem. Acc*. **99**. 95−99 (1998).
18. Grimme, S., Antony, J., Ehrlich, S. & Krieg, H. A consistent and accurate *ab initio* parametrization of density functional dispersion correction (DFT−D) for the 94 elements H−Pu. *J. Chem. Phys*. **132**, 154104−154122 (2010).
19. Weigend, F. & Ahlrichs, R. Balanced basis sets of split valence, triple zeta valence and quadruple zeta valence quality for H to Rn: Design and assessment of accuracy. *Phys. Chem. Chem. Phys*. **7**, 3297−3305 (2005).
20. Weigend, F. Accurate coulomb−fitting basis sets for H to Rn. *Phys. Chem. Chem. Phys*. **8**, 1057−1065 (2006).
21. Legault, C. Y. CYLview, 1.0b; Universitéde Sherbrooke, (2009). http://www.cylview.org
