# Supplementary material for: Transient-axial-chirality controlled asymmetric rhodium-carbene C(sp2)-H functionalization for the synthesis of chiral fluorenes
Source: Nat Commun. 2020 May 12;11:2363. doi: 10.1038/s41467-020-16098-8 (PMC7217916; doi:10.1038/s41467-020-16098-8)
Supplement: Supplementary file 3 — Description of Additional Supplementary Files [file 41467_2020_16098_MOESM3_ESM.pdf]

Description of Additional Supplementary Files

File Name: Supplementary Data 1
Description: The Cartesian coordinates for the stationary points
