# Supplementary material for: Transient-axial-chirality controlled asymmetric rhodium-carbene C(sp2)-H functionalization for the synthesis of chiral fluorenes
Source: Nat Commun. 2020 May 12;11:2363. doi: 10.1038/s41467-020-16098-8 (PMC7217916; doi:10.1038/s41467-020-16098-8)
Supplement: Supplementary file 4 — Supplementary Data 1 [file 41467_2020_16098_MOESM4_ESM.zip › 228470_2_data_set_4528927_q8906k.doc]

**Cartesian coordinates for the stationary points**

**N_2_**

N	0.00000000	0.00000000	0.55831700

N	0.00000000	0.00000000	-0.55831700

**Sub**

C	0.00234500	-2.30174400	0.50720700

O -0.05420600	-3.34550000	1.15008900

O	1.14022500	-1.55942900	0.35313100

C	2.30169800	-2.12369300	1.03561400

H	2.10486000	-2.12544700	2.12363500

H	2.41540400	-3.17919900	0.72804200

C	3.47198000	-1.33189200	0.70058300

C	4.48096100	-0.68828800	0.43989100

C -1.13854000	-1.68395400	-0.19744100

C	5.65444600	0.06091400	0.12864700

C	6.45100300	-0.28494000	-0.99039500

C	6.04076700	1.16162200	0.93201200

C	7.59996300	0.45233000	-1.29338800

H	6.15304000	-1.13416900	-1.61218900

C	7.19169500	1.89248700	0.62063400

H	5.42590500	1.43036100	1.79593100

C	7.97406800	1.54160300	-0.49083800

H	8.20743300	0.17597200	-2.16129300

H	7.48015800	2.74163600	1.24864300

H	8.87413500	2.11655800	-0.73140100

C -2.50612000	-2.24238900	-0.20940200

C -3.66624500	-1.40306700	-0.22453000

C -2.67722600	-3.64240500	-0.20669700

C -4.94882200	-2.00734300	-0.27264700

C -3.95374500	-4.21266300	-0.22834000

H -1.79268200	-4.28072500	-0.17035100

C -5.09427400	-3.39456000	-0.27230700

H -5.82570300	-1.35380000	-0.29100300

H -4.05553300	-5.30247000	-0.22199200

H -6.09452100	-3.83805900	-0.29977000

C -3.58132700	0.01329600	-0.15218800

C -3.55122200	1.24121700	-0.07609800

C -3.50316000	2.65928700	0.02708700

C -2.30841200	3.36437200	-0.26779000

C -4.64924100	3.39073300	0.43010100

C -2.26695500	4.75702500	-0.15436500

H -1.42708500	2.80174400	-0.58971600

C -4.59567800	4.78358700	0.53698100

H -5.57176000	2.84903800	0.65883500

C -3.40683300	5.47188500	0.24711500

H -1.33854400	5.28970200	-0.38494300

H -5.48750100	5.33643100	0.84990200

H -3.36959900	6.56277000	0.33166000

N -0.60525800	0.34928300	-1.49853100

N -0.86801600	-0.59091600	-0.88952900

**Rh_2_(HCOO)_4_**

Rh 0.00342800	0.00332800	-1.19979500

Rh -0.00335600	-0.00325400	1.19981700

O	1.45221000	1.44984900	1.15877700

O -1.45706500	1.45175900	1.15160700

O -1.45875300	-1.45656700	1.14463100

O	1.45012700	-1.45854200	1.15175000

O -1.45194200	-1.44998100	-1.15876400

O -1.44991700	1.45853200	-1.15177400

O	1.45684700	-1.45192500	-1.15164500

O	1.45863000	1.45670000	-1.14461000

C -1.84713000	1.84889600	-0.00013000

C	1.84931100	1.84674800	0.00932600

C	1.84673900	-1.84931600	0.00004700

C -1.84942100	-1.84655000	-0.00933800

H -2.62913600	2.63099800	-0.00028200

H -2.63232700	-2.62774700	-0.01389400

H	2.62812700	-2.63205000	0.00014000

H	2.63198200	2.62817500	0.01387200

**Pro**

C -1.46535400	0.07221700	-0.84552400

C -0.43223200	2.17252300	-0.24502200

C -0.36407100	3.55506100	-0.00683100

C -1.65411500	1.57364600	-0.63923200

C -1.52741100	4.32437300	-0.16234200

H	0.57299100	4.03365100	0.29643100

C -2.80994500	2.34076400	-0.78485300

H -1.62040300	-0.16738000	-1.91767600

C -2.73934700	3.72497000	-0.54606100

H -1.49038000	5.40312400	0.02065700

H -3.75814700	1.87263100	-1.07074000

H -3.63759900	4.34074500	-0.65746300

C	0.99260500	-3.72881300	-0.93213200

C	0.20681100	-2.58672100	-0.90303700

C	0.76941300	-1.32644100	-0.54684900

C	2.17847700	-1.27057600	-0.21641600

C	2.95747600	-2.45834400	-0.25252500

C	2.37149400	-3.66539500	-0.60596300

H	0.54409800	-4.68913300	-1.20689400

H -0.85865500	-2.64698900	-1.14319800

C	0.01107300	-0.11764400	-0.50871400

C	2.72288400	0.00415300	0.12922600

H	4.01861100	-2.38873300	0.00180400

H	2.97731900	-4.57695800	-0.63328200

C	1.96328700	1.16299900	0.15105800

C	0.58680400	1.11994700	-0.17124000

C -2.45280700	-0.76659500	-0.03869500

C -2.33835600	-0.86521300	1.36142000

C -3.52034600	-1.42159700	-0.67942900

C -3.26878100	-1.60552000	2.10039300

H -1.51021700	-0.36033100	1.87042400

C -4.45516600	-2.16218700	0.06041900

H -3.61829200	-1.35347000	-1.76969900

C -4.33107200	-2.25665500	1.45292700

H -3.16370300	-1.67610300	3.18810200

H -5.27825600	-2.66772700	-0.45547800

H -5.05637700	-2.83666400	2.03263800

C	4.11459700	0.34993600	0.50421300

O	5.10817600	-0.34701300	0.61312900

O	4.15942800	1.72115100	0.74550200

C	2.85947500	2.30461300	0.54603700

H	2.54633300	2.79615400	1.48668600

H	2.93949900	3.08307300	-0.23633300

**IN1**

C	0.78273500	-0.82362300	-0.55705800

O	0.77214400	-1.53793200	-1.55119500

O	1.48182000	0.32322100	-0.41507600

C	2.23555000	0.70152300	-1.61266300

H	2.76173900	-0.19365300	-1.98985700

H	1.50419300	1.01007000	-2.38083100

C -0.01581200	-1.07344400	0.70746700

C	0.60748700	-0.76135100	2.05885600

C -0.00344300	0.09777400	2.98113400

C	1.84608200	-1.39443600	2.39839000

C	0.60130800	0.36559500	4.21798700

C	2.43843800	-1.11256500	3.65322300

C	1.82163900	-0.23892500	4.55201500

H	0.11261400	1.04777000	4.92062500

H	3.38659500	-1.59630400	3.90454000

H	2.29377400	-0.03247200	5.51789000

N -0.96858800	-3.38887100	0.69537100

N -0.49384500	-2.35594800	0.69420200

H -0.94985500	0.56945300	2.71836700

Rh -2.03536600	-0.02514200	0.03748900

Rh -4.16569000	0.99349100	-0.59996700

O -4.94629100	0.31385100	1.18431600

O -3.57076700	2.73984700	0.30457000

O -3.31040600	1.62383200	-2.35731800

O -4.68878700	-0.78286700	-1.49828600

O -1.28584000	0.69324600	-1.76590300

O -1.55194200	1.80387500	0.90451300

O -2.68537500	-1.74659500	-0.90373200

O -2.94889100	-0.67004600	1.77944000

C -2.41100300	2.74908100	0.83535100

C -4.18135500	-0.35960400	1.94864900

C -3.84263600	-1.73437900	-1.45080200

C -2.07992600	1.33587400	-2.53503200

H -2.10177900	3.70461500	1.30183900

H -1.63635800	1.68943000	-3.48605300

H -4.14158900	-2.67831500	-1.94524300

H -4.63555300	-0.72721300	2.88906200

C	3.15704400	1.76879000	-1.27173500

C	3.95847600	2.66107600	-1.02263000

C	4.90142400	3.68882200	-0.72383100

C	6.17830800	3.35981900	-0.20580800

C	4.57887700	5.05100300	-0.93951500

C	7.10198100	4.36794500	0.08731200

H	6.42778500	2.30804000	-0.03812500

C	5.51035100	6.05168500	-0.64471200

H	3.59270000	5.30661300	-1.33798500

C	6.77265300	5.71497800	-0.13108900

H	8.08535600	4.10145300	0.48817300

H	5.24984900	7.10123200	-0.81573300

H	7.49877100	6.50136700	0.09899800

C	2.47424900	-2.30138600	1.50366200

C	2.99223000	-3.09718700	0.72049100

C	3.57659900	-4.01359600	-0.19694300

C	4.68725400	-4.80806900	0.18417100

C	3.04754600	-4.14521500	-1.50689900

C	5.24905000	-5.71139200	-0.72265300

H	5.09411200	-4.70579600	1.19455900

C	3.62058800	-5.05269300	-2.40275700

H	2.19530100	-3.52445600	-1.80013700

C	4.71894700	-5.83784300	-2.01674700

H	6.10566700	-6.32158000	-0.41829800

H	3.20600800	-5.14807100	-3.41161400

H	5.16204700	-6.54713700	-2.72329900

**IN2**

C	0.79873300	-0.81907600	-0.28464600

O	0.78929700	-1.43172300	-1.34867200

O	1.74610600	0.08768900	0.07750200

C	2.76200900	0.32694000	-0.94829600

H	3.39872100	-0.57456600	-1.01992000

H	2.24974800	0.44863300	-1.92019600

C -0.28400900	-0.86704200	0.73807400

C -0.05624500	-1.47195900	2.01705400

C -0.91735200	-1.12661700	3.10388600

C	1.04021900	-2.38838300	2.29412900

C -0.70644700	-1.60618000	4.39235900

C	1.21539600	-2.87348200	3.61278900

C	0.36268700	-2.48633700	4.64738500

H -1.37098700	-1.29677500	5.20481500

H	2.03865800	-3.56775100	3.80323300

H	0.52918500	-2.87351900	5.65787900

H -1.72889100	-0.42645500	2.90606800

Rh -1.95326900	-0.00618800	0.06160600

Rh -3.98583800	1.06145000	-0.87312200

O -5.11058000	-0.19265100	0.31560000

O -3.89938500	2.44718500	0.64679300

O -2.76725400	2.25490100	-2.02437200

O -4.00445000	-0.34387800	-2.36518500

O -0.86510900	1.27568900	-1.16314100

O -2.02250000	1.45233500	1.54254500

O -2.12986800	-1.36487300	-1.49214200

O -3.22662200	-1.20269200	1.18565900

C -2.95843900	2.32872800	1.49414700

C -4.48994400	-1.02444600	1.05080400

C -3.08499900	-1.22621600	-2.33180600

C -1.51033000	2.09074600	-1.91179700

H -2.93264700	3.07995200	2.30733100

H -0.87538700	2.74066200	-2.54495100

H -3.10604100	-1.98498500	-3.13759100

H -5.11974400	-1.69460400	1.66812100

C	3.53590200	1.49995200	-0.58721500

C	4.21354500	2.48490800	-0.31990500

C	4.99599400	3.63589200	-0.00800700

C	6.38381800	3.51636900	0.24933800

C	4.39672900	4.91835800	0.04747900

C	7.14612400	4.64974500	0.54954900

H	6.84837700	2.52679200	0.20871900

C	5.16698700	6.04464500	0.35353600

H	3.32475600	5.01194200	-0.14952200

C	6.54225700	5.91582300	0.60373900

H	8.21835900	4.54445100	0.74457400

H	4.69157300	7.03000700	0.39573500

H	7.14227400	6.80026500	0.84105300

C	1.91879000	-2.87011800	1.29767800

C	2.69917200	-3.36912700	0.48494900

C	3.54472200	-3.90411900	-0.52263400

C	4.65347400	-4.72158900	-0.18507500

C	3.27657100	-3.62565200	-1.88961500

C	5.47187900	-5.24364300	-1.19049500

H	4.85705100	-4.93631300	0.86813300

C	4.10425700	-4.15599800	-2.88292800

H	2.41865000	-2.99403700	-2.13896800

C	5.20128900	-4.96355700	-2.53993600

H	6.32591300	-5.87349600	-0.92152300

H	3.89129900	-3.93908200	-3.93476100

H	5.84514100	-5.37518400	-3.32392600

**IN3**

Rh 3.96740200	-0.46760300	-0.42649200

Rh 1.54180600	-0.37154400	0.05127200

O	1.51619000	-2.40668000	-0.34979000

O	2.03947700	-0.79283800	2.01355700

O	1.77619100	1.67311900	0.33129400

O	1.29439200	0.01026600	-1.99326900

O	4.04740700	1.56243200	-0.05974100

O	4.29792100	-0.86295700	1.55802000

O	3.55876700	-0.09213400	-2.40568300

O	3.78175600	-2.48022400	-0.77531500

C	3.27276600	-0.95266200	2.31209500

C	2.61427100	-2.98393200	-0.65740900

C	2.33776800	0.05070000	-2.73527000

C	2.95686500	2.15987100	0.21565400

C -0.99822900	-1.73645300	0.56258800

O -0.92676100	-2.52928200	1.47761200

O -1.41864500	-2.12011900	-0.70617800

C -1.55717500	-0.99058200	-1.56248000

H -0.83207300	-1.06595900	-2.38879300

H -2.58977500	-0.95605900	-1.94718600

C -1.24989700	0.24274600	-0.70174500

C -1.51176300	1.48256000	-0.97411100

C -0.61367800	-0.27316800	0.58177000

H	3.47003000	-1.19794200	3.37341100

H	3.03170400	3.25315000	0.37998000

H	2.14662500	0.23982400	-3.81040700

H	2.53448200	-4.07131100	-0.84888000

C -1.77622200	2.82257800	-1.17420100

C -3.01343800	3.39104200	-0.71797800

C -0.82679500	3.66816100	-1.84089500

C -3.27460800	4.74256000	-0.91959400

H -3.72728700	2.73974100	-0.20536900

C -1.11615200	5.01289100	-2.04661700

H	0.11839400	3.22780200	-2.17020400

C -2.33407400	5.55294600	-1.58611900

H -4.21391600	5.17688700	-0.56381600

H -0.39322700	5.65627900	-2.55760000

H -2.55022500	6.61421700	-1.74558500

C -1.04275000	0.45105200	1.84281800

C -0.16264000	1.16939000	2.66595300

C -2.43090500	0.42975600	2.20964500

C -0.60627200	1.83865200	3.81639200

H	0.89142200	1.20419100	2.39912000

C -2.86571100	1.11024800	3.37303100

C -1.96010900	1.80856100	4.17449600

H	0.11566900	2.38306500	4.43406100

H -3.92763800	1.07066400	3.63387900

H -2.31095200	2.32494600	5.07391700

C -3.40473000	-0.25111600	1.42506900

C -4.27828100	-0.82438400	0.77380800

C -5.24989200	-1.52115500	0.00122900

C -6.60708400	-1.11113800	-0.00077700

C -4.86972500	-2.64088500	-0.78401900

C -7.55135500	-1.79816800	-0.76979300

H -6.90384100	-0.25154800	0.60780000

C -5.82448300	-3.31835300	-1.54896500

H -3.82534600	-2.96797100	-0.76956200

C -7.16525300	-2.90151100	-1.54752600

H -8.59653000	-1.47162300	-0.76161600

H -5.51996100	-4.18333900	-2.14741200

H -7.90814300	-3.43731200	-2.14719100

**^3^IN3**

Rh 3.01115600	-2.24101700	0.08428200

Rh 1.20988100	-0.59340200	-0.08167200

O	0.27619400	-1.95563200	-1.32379500

O	0.40771000	-1.51003400	1.59097400

O	2.37984700	0.64524100	1.12153000

O	2.19932100	0.17089500	-1.74889800

O	4.05066300	-0.92970600	1.28115300

O	2.10836300	-3.05634000	1.73314900

O	3.87460300	-1.39849500	-1.58551500

O	1.94534700	-3.52341100	-1.10918100

C	1.01407100	-2.51020500	2.10378700

C	0.82711100	-3.08556700	-1.54387000

C	3.28261400	-0.40510800	-2.11898500

C	3.50985600	0.19825100	1.52483700

C -1.78418000	-0.28772500	-0.81866300

O -2.44228300	-1.27176100	-0.54137500

O -1.59042300	0.10185600	-2.13491200

C -0.70421000	1.22271300	-2.21483400

H	0.18757800	0.93663800	-2.79741300

H -1.22150100	2.05867200	-2.71779100

C -0.35075200	1.59278100	-0.76892600

C	0.32803300	2.71091900	-0.43344000

C -1.03305200	0.64318800	0.08325500

H	0.52332600	-2.95815200	2.98825000

H	4.09279800	0.88707600	2.16602700

H	3.76613600	0.02269800	-3.01831700

H	0.25686300	-3.76981600	-2.19960800

C	1.10663800	3.83798500	-0.68698200

C	0.55225100	5.15637400	-0.57876200

C	2.49959600	3.71417500	-1.01204900

C	1.34647700	6.27724300	-0.80742300

H -0.50467100	5.26373600	-0.31775300

C	3.27394300	4.85101300	-1.23156900

H	2.92742800	2.71127900	-1.09052300

C	2.70884500	6.13666900	-1.13335000

H	0.90346100	7.27553600	-0.72961200

H	4.33338500	4.73933700	-1.48586300

H	3.32628900	7.02321000	-1.30743200

C -1.39487200	0.90879100	1.48400800

C -0.41299700	1.44837400	2.35085100

C -2.73521400	0.77037500	1.99515700

C -0.69429600	1.79420000	3.67285800

H	0.60739000	1.55987800	1.97871300

C -2.99161600	1.12110400	3.34867700

C -1.99180200	1.61716400	4.18204200

H	0.10201700	2.19348100	4.30925800

H -4.01528200	1.00645100	3.71634700

H -2.22402100	1.87545700	5.22029300

C -3.86378900	0.39935500	1.22073300

C -4.91727000	0.12385300	0.64733800

C -6.03221900	-0.29743500	-0.12748200

C -7.35742100	0.05301600	0.23196800

C -5.81694500	-1.09426400	-1.28299200

C -8.43607300	-0.37960900	-0.54563100

H -7.52316300	0.66563800	1.12329700

C -6.90464700	-1.51997400	-2.05042000

H -4.79102600	-1.37199300	-1.54340300

C -8.21475200	-1.16558900	-1.68786800

H -9.45613800	-0.10313200	-0.25916300

H -6.72991400	-2.13690400	-2.93818200

H -9.06231500	-1.50354000	-2.29321600

**IN4**

Rh 4.42069400	-0.61895200	0.13322200

Rh 2.01677000	-0.13627500	-0.27273900

O	1.99053900	-1.91124200	-1.36329200

O	1.62573700	-1.19111400	1.48015500

O	2.30744800	1.57894700	0.86862400

O	2.62924200	0.84789600	-1.99764200

O	4.54328300	1.13243300	1.20738000

O	3.86272500	-1.61307000	1.85080200

O	4.85745400	0.39588000	-1.60400000

O	4.21762500	-2.34878100	-0.95761000

C	2.62379000	-1.67583000	2.12736400

C	3.07433000	-2.59336900	-1.45515000

C	3.88427600	0.88219300	-2.26263100

C	3.48079200	1.81553600	1.33552700

C -2.53564400	-1.80150400	1.14469900

O -3.45702700	-2.05811000	1.89532700

O -1.94274800	-2.76814900	0.34682800

C -0.85093200	-2.19747200	-0.38014100

H	0.09082900	-2.67393800	-0.06198600

H -0.99391700	-2.39637200	-1.45761300

C -0.86326400	-0.71748300	-0.05684800

C	0.07642400	0.25480700	-0.57006400

C -1.85208800	-0.49950000	0.87567500

H	2.35458000	-2.22133000	3.05261400

H	3.56316400	2.74647700	1.92955100

H	4.14500900	1.41279400	-3.19909300

H	2.98759100	-3.52193100	-2.05213600

C -0.44008000	1.43934200	-1.22540400

C -1.76063300	1.44150900	-1.77308400

C	0.34471200	2.62053300	-1.37524400

C -2.25806800	2.55774800	-2.44350500

H -2.38402000	0.54945500	-1.66737200

C -0.17141600	3.74122000	-2.02521900

H	1.34152900	2.64802100	-0.93593800

C -1.46687700	3.71263300	-2.56973400

H -3.26854000	2.53344100	-2.86311100

H	0.43831300	4.64620600	-2.11229900

H -1.86135700	4.59166600	-3.09010900

C -2.13806100	0.74421300	1.60731000

C -1.07966100	1.42338500	2.24521000

C -3.45729000	1.29105600	1.67819800

C -1.30218100	2.61057800	2.95219900

H -0.07359800	0.99525800	2.19178600

C -3.65981900	2.49558300	2.39601900

C -2.59768600	3.14514800	3.02977800

H -0.46470200	3.11327800	3.44622900

H -4.67110900	2.91009500	2.43924300

H -2.78100900	4.07098900	3.58445100

C -4.54940900	0.68489200	1.00126400

C -5.50857500	0.17772500	0.42311400

C -6.58131100	-0.51362200	-0.20589500

C -7.66388100	0.18553100	-0.79436900

C -6.57459600	-1.93205100	-0.24088100

C -8.70746400	-0.51704000	-1.40505100

H -7.67124200	1.27927700	-0.76372800

C -7.62441000	-2.62194500	-0.85436200

H -5.74539500	-2.46636200	0.23304900

C -8.69148300	-1.92041300	-1.43900300

H -9.53944500	0.03346100	-1.85652400

H -7.61255100	-3.71666900	-0.87284500

H -9.51117800	-2.46653700	-1.91711500

**IN5**

Rh 2.85996700	-1.79080000	-0.46285900

Rh 0.90843700	-0.38430000	0.11272800

O -0.27379600	-2.00159400	-0.47645200

O	1.13222200	-1.10890700	2.05312800

O	2.30258500	1.09587000	0.57860800

O	0.91009900	0.23252100	-1.85883500

O	4.10353000	-0.24010800	0.04698500

O	2.90995100	-2.46157100	1.48155600

O	2.74913200	-1.05200300	-2.37649300

O	1.56029800	-3.30739400	-0.97630600

C	2.06068800	-1.95840500	2.28844500

C	0.31507200	-3.06866500	-0.87507100

C	1.82037100	-0.21822000	-2.63676600

C	3.54856100	0.83874200	0.44084600

C	0.89466000	3.98757200	1.31094100

O	1.42051200	5.04662600	1.00438000

O	1.02981000	3.44815300	2.58566600

C	0.32226100	2.19772700	2.66526800

H	1.03144600	1.39499600	2.93184300

H -0.43865700	2.27086900	3.46309000

C -0.27285500	1.98209100	1.29697000

C -0.96453100	0.80687300	0.82382300

C	0.05713200	3.06830700	0.51887600

H	2.12874400	-2.30277400	3.33885400

H	4.23175000	1.67012700	0.70081100

H	1.79012200	0.16871000	-3.67354900

H -0.36081200	-3.89570700	-1.17180600

C -0.35764900	3.19023700	-0.85739200

C -1.21044000	2.17965200	-1.38771700

C	0.04427900	4.26408400	-1.68602900

C -1.64498500	2.27572500	-2.72238100

C -0.39739500	4.34024700	-3.00832600

H	0.71026100	5.01962000	-1.25970000

C -1.25001300	3.34878400	-3.52751200

H -2.29374800	1.49309900	-3.13170600

H -0.08030500	5.17661000	-3.63963900

H -1.60580400	3.41231200	-4.56091300

C -1.65267400	1.05137200	-0.52260100

C -2.71964700	0.32297500	-0.80409900

C -3.68462400	-0.61834300	-1.07600600

C -3.38059700	-1.75567500	-1.90560500

C -5.02529300	-0.46984300	-0.57431600

C -4.38211500	-2.65818800	-2.24470300

H -2.35269900	-1.87803800	-2.25539900

C -5.99111700	-1.42338300	-0.87234700

H -5.25011600	0.38798400	0.06518800

C -5.68247800	-2.50624000	-1.72159200

H -4.15274300	-3.50843100	-2.89496400

H -7.00213100	-1.32024400	-0.46643500

H -6.45750000	-3.23689700	-1.97378800

C -1.85326700	0.11501200	1.85093600

C -2.73814400	0.93193200	2.59487800

C -1.90499400	-1.27527900	2.06366800

C -3.62764600	0.38393600	3.52811700

H -2.73300800	2.01520300	2.42586900

C -2.79666500	-1.82483800	2.99743200

H -1.24794300	-1.92873000	1.49050900

C -3.65800100	-1.00282300	3.73620500

H -4.29639200	1.04340100	4.09175800

H -2.81248500	-2.90983400	3.14772700

H -4.34823800	-1.43776100	4.46659800

**^3^IN5**

Rh 2.86941700	-1.54849800	-1.01373500

Rh 1.08554900	-0.24448500	0.00746000

O -0.22252200	-1.72347900	-0.66640900

O	1.46112200	-1.26196000	1.76869900

O	2.54202900	1.12662200	0.53013800

O	0.85203100	0.70035200	-1.81338900

O	4.21865900	-0.11130500	-0.44185400

O	3.13368300	-2.50860100	0.79034900

O	2.54247800	-0.53710600	-2.77376700

O	1.49037800	-2.96356000	-1.57686000

C	2.38475200	-2.14815900	1.75898500

C	0.27167100	-2.72779300	-1.28801200

C	1.62240200	0.34990500	-2.77172400

C	3.75331900	0.89508300	0.19079500

C	1.02263800	3.73694100	1.29892700

O	1.50741800	4.77269000	0.87087600

O	1.45023500	3.16870600	2.48853200

C	0.72273500	1.95566200	2.75999700

H	1.43662700	1.12037400	2.85367800

H	0.20779300	2.08479800	3.72680000

C -0.22256600	1.77942800	1.59040600

C -1.17275600	0.75939500	1.33432100

C -0.04812700	2.88059400	0.74656200

H	2.55356700	-2.67000400	2.72025000

H	4.48622000	1.66905800	0.48631800

H	1.46815600	0.88731900	-3.72666100

H -0.45877300	-3.49201000	-1.61822300

C -0.81016300	3.06680400	-0.44686300

C -1.78930200	2.06855300	-0.77195800

C -0.62491200	4.18497600	-1.30281500

C -2.54809200	2.23195400	-1.95228900

C -1.38996900	4.31600300	-2.45517700

H	0.13259900	4.92289400	-1.02542000

C -2.35366500	3.33554900	-2.78057800

H -3.29795600	1.48021200	-2.21496400

H -1.24511400	5.18003300	-3.11168000

H -2.95549100	3.44090300	-3.68916800

C -2.00480900	0.91438000	0.13404000

C -3.00897600	0.02729700	-0.10176400

C -4.03938800	-0.63785400	-0.74800000

C -3.75684200	-1.61354900	-1.76688000

C -5.41417400	-0.41496700	-0.39316700

C -4.79320700	-2.30106800	-2.39221700

H -2.71207000	-1.79372300	-2.03532800

C -6.43120400	-1.11670000	-1.03441700

H -5.64428300	0.31612600	0.38707400

C -6.13446500	-2.06172100	-2.03545200

H -4.55877600	-3.03471600	-3.17091600

H -7.47275400	-0.92852900	-0.75352400

H -6.94172200	-2.60864800	-2.53192100

C -1.57545600	-0.22742100	2.37759100

C -1.83518900	0.21133800	3.69516100

C -1.79696800	-1.58893900	2.07262000

C -2.27738800	-0.68208400	4.67964100

H -1.74055800	1.27297800	3.94221500

C -2.23592500	-2.48081900	3.05885600

H -1.58941000	-1.94736100	1.06254500

C -2.47356300	-2.03471400	4.36711500

H -2.48074300	-0.31420400	5.69071200

H -2.39035400	-3.53420400	2.80179400

H -2.81700900	-2.73440400	5.13606700

**IN6**

Rh -3.49076100	-0.85468100	-0.81651800

Rh -1.31752900	-0.04106400	0.09455400

O -1.00933000	-2.01097300	0.65428700

O -0.51589200	-0.51966500	-1.77097500

O -1.89939800	1.83395700	-0.62292100

O -2.32534700	0.30001900	1.87497100

O -3.90803300	1.05796200	-1.44521800

O -2.54446400	-1.24505100	-2.59892300

O -4.33551200	-0.44522400	1.02007100

O -3.02586700	-2.74854300	-0.18046600

C -1.29652200	-1.00874300	-2.66551500

C -1.90038000	-2.89406600	0.39414700

C -3.57842400	0.02193600	1.92771300

C -3.03469100	1.94994300	-1.21061700

C	0.42082200	0.64500000	0.83451500

H -1.64186300	-3.91968000	0.72084800

H -0.80373400	-1.25323700	-3.62660700

H -3.27386800	2.97425600	-1.55794000

H -4.05384900	0.22214700	2.90777500

C	2.37980000	1.77377800	-1.11904600

C	3.44576200	2.68365500	-0.93673600

C	1.25042000	2.19690800	-1.84860500

C	3.38165600	3.98030900	-1.46272100

H	4.31994000	2.37613800	-0.35179600

C	1.18985700	3.49536500	-2.37546100

H	0.42961400	1.49618500	-2.01450900

C	2.25147500	4.39077900	-2.18608400

H	4.21400900	4.67327600	-1.30100800

H	0.30471000	3.80507000	-2.94129700

H	2.20052200	5.40343700	-2.59925900

C	1.57079300	-2.91285000	2.98713600

C	1.23081200	-1.74999400	2.31054900

C	1.94725100	-1.34424100	1.14829300

C	3.05568700	-2.15761900	0.71745300

C	3.36883600	-3.35869500	1.40990900

C	2.63418800	-3.72850300	2.52748600

H	1.00912900	-3.20291400	3.88125000

H	0.40389900	-1.13057300	2.67052000

C	1.63843400	-0.10173000	0.46468400

C	3.82024000	-1.67096000	-0.38121900

H	4.20972600	-3.95715800	1.04904300

H	2.88681000	-4.64783800	3.06597200

C	3.57146900	-0.43404900	-0.94976900

C	2.50374900	0.40390300	-0.53530800

C	0.55753100	1.78025600	1.72207200

C	1.78400900	1.98445000	2.43044800

C -0.50749300	2.69851500	1.96649000

C	1.92740800	3.03228300	3.33773700

H	2.61122100	1.28863800	2.26548100

C -0.34814900	3.75596900	2.86163600

H -1.44087900	2.57833800	1.41879600

C	0.86267500	3.92443000	3.55488000

H	2.87050000	3.16038300	3.87848300

H -1.17330800	4.45645100	3.02544000

H	0.97733500	4.75096300	4.26404900

C	4.97578200	-2.28969700	-1.06926300

O	5.53397300	-3.35733700	-0.88394800

O	5.39439700	-1.40927100	-2.06150300

C	4.57637300	-0.22428800	-2.05534700

H	5.23229400	0.65051100	-1.89999200

H	4.09838800	-0.11512800	-3.04662200

**MECP-1**

Rh 3.80567800	-0.89427500	-0.22900100

Rh 1.39570600	-0.41398000	-0.05363300

O	1.10167400	-2.29100000	-0.87947600

O	1.53490200	-1.28425100	1.82125700

O	1.92884300	1.45670300	0.68152900

O	1.53955700	0.35328800	-2.00047000

O	4.17707600	0.95981900	0.59425600

O	3.78069200	-1.75302000	1.63059400

O	3.80145400	-0.05090900	-2.09957800

O	3.35802800	-2.71970700	-1.06012800

C	2.64884500	-1.76472000	2.22000200

C	2.11959700	-2.99880700	-1.18857600

C	2.68874600	0.35767900	-2.56492200

C	3.17491000	1.70657100	0.84114000

C -1.32228200	-1.63620900	0.29417100

O -1.39604300	-2.56338500	1.07190500

O -1.67545200	-1.77101800	-1.04361600

C -1.53123300	-0.53147100	-1.74247100

H -0.77209600	-0.64870800	-2.53522800

H -2.50311000	-0.26018000	-2.18952300

C -1.09913900	0.50337200	-0.69542000

C -1.07637600	1.82023300	-0.89046100

C -0.83589900	-0.22942500	0.54815700

H	2.62066100	-2.25091200	3.21356800

H	3.40462900	2.71022100	1.24836300

H	2.71253400	0.77705500	-3.58932800

H	1.88592300	-3.98664200	-1.62951700

C -0.95933000	3.18051600	-1.06032000

C -2.07085500	4.06184800	-0.81625300

C	0.28289400	3.76017000	-1.50418600

C -1.93351800	5.43341400	-1.00318300

H -3.01845100	3.63040800	-0.48080700

C	0.39074400	5.13465900	-1.68068800

H	1.12731600	3.09240000	-1.69275600

C -0.70837000	5.98202600	-1.43298900

H -2.78966700	6.09043000	-0.81780100

H	1.34433400	5.56010800	-2.01053500

H -0.60854000	7.06302900	-1.57026500

C -1.18802700	0.37975600	1.87695900

C -0.27334700	0.95127700	2.77426500

C -2.57996200	0.41837200	2.22124100

C -0.69357200	1.53563200	3.97859100

H	0.78590600	0.94618600	2.52415000

C -2.98758900	1.01168900	3.44026800

C -2.05342200	1.56682000	4.31562400

H	0.05038900	1.97106700	4.65355800

H -4.05571700	1.02751300	3.67669900

H -2.38636400	2.02506700	5.25252200

C -3.57867400	-0.10870400	1.35721700

C -4.47313300	-0.56878500	0.64833700

C -5.49297700	-1.13931300	-0.16091100

C -6.85048400	-0.77878900	0.03293900

C -5.16790800	-2.08903500	-1.16363500

C -7.85150800	-1.34941300	-0.75844600

H -7.10040600	-0.05222000	0.81229400

C -6.17863800	-2.65095400	-1.94954300

H -4.12164600	-2.38311400	-1.29584900

C -7.52000200	-2.28410700	-1.75266800

H -8.89639500	-1.06417800	-0.59738600

H -5.91751400	-3.38554800	-2.71842700

H -8.30684500	-2.72849100	-2.37078400

**MECP-2**

Rh 2.85683600	-1.79819200	-0.45694800

Rh 0.91551700	-0.37904600	0.11146100

O -0.27129000	-1.99597500	-0.47448800

O	1.13477300	-1.09703200	2.05521600

O	2.31110700	1.09808800	0.57487300

O	0.92042400	0.23373800	-1.85987500

O	4.10507200	-0.24865000	0.04850400

O	2.90370300	-2.46359800	1.48918400

O	2.74725100	-1.06878700	-2.37500800

O	1.55613400	-3.31487700	-0.96412900

C	2.05660100	-1.95278300	2.29374400

C	0.31170900	-3.06845900	-0.86636400

C	1.82477800	-0.22898200	-2.63791100

C	3.55595700	0.83426800	0.43916300

C	0.90257100	3.98688000	1.30133500

O	1.42731800	5.04595300	0.99317300

O	1.03601800	3.45031600	2.57722500

C	0.32591500	2.20110000	2.66046700

H	1.03357100	1.39799700	2.92960000

H -0.43553100	2.27811700	3.45738600

C -0.26956200	1.98240900	1.29316300

C -0.98131500	0.82090700	0.82649400

C	0.06550200	3.06609900	0.51018100

H	2.12053500	-2.29582000	3.34475200

H	4.24312600	1.66259700	0.69797500

H	1.79470700	0.15232000	-3.67667900

H -0.36793100	-3.89342600	-1.15989100

C -0.35239100	3.18718400	-0.86385700

C -1.21026800	2.17673400	-1.38936500

C	0.05035400	4.25938100	-1.69544700

C -1.64453100	2.27017800	-2.72451800

C -0.39234200	4.33174900	-3.01696700

H	0.71656200	5.01595100	-1.27142500

C -1.24648000	3.33887800	-3.53301200

H -2.29579400	1.48772300	-3.13033700

H -0.07554700	5.16580100	-3.65167900

H -1.60032800	3.39851100	-4.56715900

C -1.65499500	1.05357900	-0.52027800

C -2.71807900	0.31728400	-0.81508500

C -3.68641900	-0.62029400	-1.08278700

C -3.38752400	-1.76277800	-1.90853500

C -5.02672500	-0.46827900	-0.57974000

C -4.39070500	-2.66580900	-2.23962000

H -2.36095600	-1.88865100	-2.26171800

C -5.99534100	-1.41956400	-0.87528100

H -5.24978100	0.39060000	0.05941200

C -5.69141400	-2.50831500	-1.71853200

H -4.16222000	-3.52064400	-2.88458300

H -7.00579000	-1.31121200	-0.46850700

H -6.46882500	-3.23747100	-1.96645000

C -1.86452800	0.12758800	1.85402400

C -2.74844000	0.94475800	2.59841400

C -1.91416100	-1.26248300	2.06760800

C -3.63640300	0.39654500	3.53311600

H -2.74522500	2.02768500	2.42726700

C -2.80389300	-1.81196800	3.00308600

H -1.25702800	-1.91516300	1.49394800

C -3.66524400	-0.99010300	3.74205600

H -4.30548000	1.05561600	4.09704000

H -2.81869900	-2.89681200	3.15464300

H -4.35507900	-1.42566000	4.47257300

**TS1**

C	0.75181500	-0.82045300	-0.52381700

O	0.76745800	-1.55466900	-1.50559800

O	1.51462600	0.28941200	-0.36850900

C	2.36196500	0.58971700	-1.52280200

H	2.89721500	-0.33368100	-1.80930700

H	1.70236400	0.86914100	-2.36390900

C -0.20355900	-0.91957900	0.64308200

C	0.35396900	-0.82223500	2.02196600

C -0.35419700	-0.14627900	3.03719500

C	1.61142600	-1.43628100	2.36117100

C	0.17030900	-0.01177200	4.32696100

C	2.12098100	-1.29499800	3.67444400

C	1.41300300	-0.58165400	4.64378500

H -0.39516800	0.53707100	5.08638600

H	3.07849700	-1.76496400	3.91593000

H	1.82677600	-0.48011800	5.65231100

N -1.29342300	-3.47552900	0.69551600

N -0.58486600	-2.61544500	0.52511100

H -1.31787000	0.29730900	2.79406000

Rh -2.02431400	-0.02435000	0.02178100

Rh -4.14325500	1.04338100	-0.66762600

O -5.03208100	0.16653600	0.97526700

O -3.65216400	2.69313600	0.45799500

O -3.16753700	1.85808100	-2.28040600

O -4.56175900	-0.63429900	-1.78060400

O -1.17494000	0.87617500	-1.65522600

O -1.65074400	1.73286900	1.07905800

O -2.59275800	-1.63994900	-1.13871700

O -3.06385400	-0.85421700	1.60850200

C -2.52661200	2.66666300	1.05228000

C -4.31343500	-0.57785400	1.71450700

C -3.70773500	-1.57783800	-1.76635300

C -1.92752400	1.59115900	-2.40433700

H -2.26043000	3.57315900	1.63036800

H -1.42335100	2.03857300	-3.28327100

H -3.95179100	-2.46939800	-2.37544000

H -4.82517300	-1.05588600	2.57240200

C	3.28025900	1.65545900	-1.16700300

C	4.08241800	2.54088500	-0.89656400

C	5.02299700	3.56274400	-0.57100700

C	6.27822200	3.22714600	-0.00626000

C	4.71900300	4.92628500	-0.80447400

C	7.19868700	4.22978400	0.31459200

H	6.51358700	2.17442300	0.17549300

C	5.64669200	5.92155600	-0.48080800

H	3.74954600	5.18718100	-1.23889500

C	6.88745600	5.57818900	0.07885100

H	8.16515900	3.95795700	0.75145300

H	5.40014700	6.97216400	-0.66551500

H	7.61073300	6.36030900	0.33137500

C	2.32947800	-2.23227300	1.43373800

C	2.96069900	-2.96249000	0.66856100

C	3.66389500	-3.79539700	-0.24275600

C	4.81226700	-4.51404300	0.17658300

C	3.21788600	-3.91963600	-1.58448000

C	5.49415500	-5.33547600	-0.72530000

H	5.15315200	-4.41740200	1.21163200

C	3.91115300	-4.74502200	-2.47450500

H	2.33349000	-3.35874500	-1.90162000

C	5.04710900	-5.45425300	-2.05129100

H	6.37923400	-5.88738100	-0.39268500

H	3.56170700	-4.83665900	-3.50800800

H	5.58411300	-6.09954800	-2.75400500

**TS2**

Rh 3.81752300	-1.00368100	-0.20614000

Rh 1.40729000	-0.53646000	0.16866900

O	1.16556200	-0.87074100	-1.87167100

O	1.21695500	-2.57435900	0.49202200

O	1.91118500	-0.29605000	2.16904000

O	1.84904500	1.45884300	-0.23076000

O	4.14646800	-0.72486600	1.80174500

O	3.45909900	-2.99358500	0.14918300

O	4.08841600	1.00976100	-0.55733100

O	3.40832600	-1.28986600	-2.20090800

C	2.25422900	-3.31839400	0.41038200

C	2.19731200	-1.17156100	-2.56994100

C	3.06629700	1.76430500	-0.49574300

C	3.13347900	-0.45019500	2.52109900

C -1.35632800	-1.43727900	0.33518500

O -1.48280600	-2.22182200	1.26576000

O -1.76285500	-1.79587900	-0.91840900

C -1.83334300	-0.75590100	-1.91450000

H -1.20851700	-1.08277800	-2.76108100

H -2.89015600	-0.70413700	-2.23553700

C -1.39178700	0.57239300	-1.46206600

C -1.23597000	1.80004600	-1.54519600

C -0.57925400	-0.17850800	0.53858000

H	2.06763000	-4.39389300	0.59561300

H	3.32364600	-0.32287200	3.60466600

H	3.24052400	2.83966300	-0.70007100

H	1.99751900	-1.34772300	-3.64565500

C -1.02665700	3.19613900	-1.46172400

C -1.91172700	4.00351300	-0.69858700

C	0.06078400	3.80990900	-2.13850500

C -1.70343600	5.38227800	-0.61172300

H -2.74344500	3.52702000	-0.17194300

C	0.24980000	5.19064300	-2.05332400

H	0.74280300	3.18324600	-2.71984500

C -0.62819400	5.97957200	-1.28982900

H -2.38469200	5.99683500	-0.01472600

H	1.08726800	5.65812600	-2.58105800

H -0.47329100	7.06131400	-1.22368200

C -1.10989100	0.81916200	1.46554700

C -2.52024700	0.92337500	1.77607300

C -0.23907100	1.76034600	2.07192900

C -2.95876800	1.89696000	2.70647500

C -0.69476600	2.71580500	2.98257500

H	0.81826200	1.71870600	1.81717200

C -2.05810700	2.77621800	3.31238600

H -4.02650800	1.94748700	2.93895100

H	0.01518700	3.41341800	3.43779700

H -2.42275400	3.51503600	4.03361200

C -3.51122500	0.12172000	1.15614900

C -4.43972200	-0.51740400	0.65961700

C -5.45635200	-1.31953200	0.07415300

C -5.11536600	-2.51970000	-0.60359500

C -6.82000900	-0.94137200	0.16549000

C -6.11875500	-3.31004700	-1.17171900

H -4.06352900	-2.81505700	-0.66079300

C -7.81197000	-1.74153000	-0.40825200

H -7.08285000	-0.01856700	0.69110300

C -7.46640700	-2.92635300	-1.07829500

H -5.84719700	-4.23654800	-1.68806900

H -8.86192200	-1.44099000	-0.33115200

H -8.24702000	-3.55108200	-1.52430600

**TS3**

Rh 3.78672100	-1.44626100	-0.29036100

Rh 1.65009400	-0.32631500	-0.09072100

O	0.97435800	-1.55246400	-1.59735400

O	1.12963800	-1.74368900	1.30919100

O	2.49177500	0.81595300	1.43094900

O	2.31991300	1.01675200	-1.53215100

O	4.52423300	-0.24052500	1.20052300

O	3.16271300	-2.80688800	1.11323000

O	4.34984800	-0.05610300	-1.69909000

O	2.99789500	-2.63226000	-1.77192200

C	1.98505300	-2.65076600	1.58767800

C	1.77622200	-2.42044000	-2.08221800

C	3.49758500	0.84485600	-2.00107800

C	3.71630300	0.60006500	1.72382600

C -1.84568600	-0.76816000	-0.64552100

O -2.32484700	-1.87668200	-0.48275400

O -1.58657900	-0.27662600	-1.92497800

C -0.98945500	1.00754700	-1.84072800

H -0.08310200	1.02743100	-2.46653100

H -1.69871600	1.78241500	-2.19252000

C -0.70142500	1.26452800	-0.34742700

C -0.02294900	2.37697800	0.06486800

C -1.35971100	0.21203300	0.35572300

H	1.66264100	-3.39667600	2.33802300

H	4.13440500	1.22526000	2.53608300

H	3.82076500	1.57483000	-2.76803400

H	1.35560000	-3.06828800	-2.87376900

C -0.14652200	3.73717600	-0.27323100

C -1.42413500	4.34905900	-0.51008900

C	1.01848200	4.57116300	-0.31446600

C -1.52077500	5.71750200	-0.75064400

H -2.32347200	3.72641600	-0.46021000

C	0.91332800	5.92128100	-0.63740100

H	1.98712900	4.10625300	-0.10838600

C -0.35465200	6.50296500	-0.83678800

H -2.50203000	6.17934300	-0.90124600

H	1.81316900	6.54154200	-0.70042900

H -0.43481000	7.57361600	-1.05098100

C -1.67955900	0.16780600	1.78251400

C -0.70280400	0.57142100	2.72477400

C -2.97219000	-0.22775700	2.27240200

C -0.94738200	0.53117800	4.09610100

H	0.27251500	0.90800700	2.36013000

C -3.19810300	-0.25922900	3.67488100

C -2.20143500	0.10550200	4.57649600

H -0.16103300	0.83090000	4.79651400

H -4.18617600	-0.56608800	4.02979700

H -2.40167100	0.07594500	5.65245000

C -4.06311100	-0.51725900	1.41518800

C -5.04833000	-0.75074400	0.71608200

C -6.08281200	-1.08665800	-0.19999900

C -7.44602900	-0.84161700	0.09839900

C -5.74345100	-1.68437700	-1.44236200

C -8.44074500	-1.17991500	-0.82454800

H -7.70759700	-0.38298400	1.05674600

C -6.74838500	-2.01981800	-2.35409200

H -4.68953000	-1.88741300	-1.65647300

C -8.09701900	-1.76811700	-2.05222100

H -9.49120600	-0.98450100	-0.58486200

H -6.47780900	-2.48450600	-3.30804200

H -8.87915600	-2.03254200	-2.77146000

**^3^TS3**

Rh 3.18628700	-2.13279300	-0.02985100

Rh 1.41223500	-0.48501000	-0.07948400

O	0.49466700	-1.66154200	-1.49121100

O	0.51197900	-1.54600900	1.44206200

O	2.49463800	0.58489500	1.34700000

O	2.44717300	0.46028400	-1.61398400

O	4.17394600	-0.98979400	1.36370400

O	2.20186000	-3.10797300	1.48435200

O	4.12205800	-1.11822300	-1.55733300

O	2.15579900	-3.24844100	-1.41123000

C	1.09347700	-2.59970400	1.86987700

C	1.05002700	-2.76217600	-1.82676100

C	3.54678200	-0.06853300	-2.00039400

C	3.60799600	0.09004900	1.73955800

C -1.97738500	-0.29643400	-0.81025400

O -2.63568500	-1.29696700	-0.58876600

O -1.68527800	0.09887300	-2.10811400

C -0.74504600	1.17570700	-2.10716800

H	0.16279800	0.86438200	-2.65003900

H -1.19185600	2.04938200	-2.61551800

C -0.46402300	1.49396600	-0.63134900

C	0.35921700	2.50009500	-0.22757300

C -1.32458400	0.64620900	0.14040600

H	0.56851600	-3.13860800	2.68043100

H	4.15166500	0.67879100	2.50313300

H	4.06604100	0.45566000	-2.82521300

H	0.50019500	-3.36472300	-2.57312400

C	1.07008100	3.66696300	-0.53798800

C	0.39103900	4.80497100	-1.08511400

C	2.47039100	3.78406900	-0.26575500

C	1.08883600	5.97861100	-1.36430200

H -0.68430500	4.73787900	-1.27725200

C	3.14921000	4.96628700	-0.55445800

H	2.98716500	2.92523000	0.17010100

C	2.46879700	6.06872700	-1.10459100

H	0.55450000	6.83540800	-1.78803800

H	4.22228900	5.03637000	-0.34739100

H	3.00921900	6.99465700	-1.32441200

C -1.65377100	0.79822700	1.55156000

C -0.65212900	1.24992600	2.44942400

C -2.98463900	0.60386700	2.07250200

C -0.91210800	1.47211800	3.80121300

H	0.36491100	1.38814600	2.07330400

C -3.21929900	0.83310900	3.45397400

C -2.20390900	1.25429300	4.31106500

H -0.10412600	1.80440500	4.46087800

H -4.23588200	0.68362700	3.82881300

H -2.41860800	1.41921900	5.37181800

C -4.11151800	0.28030200	1.27569500

C -5.15152800	0.02292900	0.67063700

C -6.25218500	-0.37050900	-0.13890200

C -7.58518700	-0.04652200	0.21605300

C -6.01371600	-1.11394200	-1.32490800

C -8.64893600	-0.45292200	-0.59562900

H -7.76886600	0.52502300	1.13083100

C -7.08676100	-1.51459700	-2.12586100

H -4.98200800	-1.37264400	-1.58218500

C -8.40483400	-1.18661500	-1.76754000

H -9.67525800	-0.19705400	-0.31231700

H -6.89409500	-2.09110200	-3.03678900

H -9.24080200	-1.50454700	-2.39928700

**TS4**

Rh 2.92221900	-1.76022200	-0.45166400

Rh 0.88388600	-0.47989200	0.18285900

O -0.20040900	-1.95259000	-0.82011800

O	0.90049700	-1.59970500	1.94388200

O	2.20470300	0.86837800	1.08665600

O	1.12340300	0.50592700	-1.61901700

O	4.07437800	-0.32592400	0.46057800

O	2.78047300	-2.78331900	1.32741900

O	3.00743900	-0.68498900	-2.20107900

O	1.69705300	-3.14895800	-1.36231400

C	1.82738300	-2.47040900	2.10873500

C	0.44595700	-2.92910200	-1.34563100

C	2.10607200	0.19690700	-2.37831100

C	3.46456000	0.64628000	1.01067100

C	0.54001700	3.92459400	1.76586200

O	0.98700700	5.05418000	1.65337300

O	0.54950700	3.27011300	2.98771500

C -0.02570300	1.97096800	2.84140200

H	0.71486200	1.20883400	3.13986900

H -0.90579500	1.88829100	3.50446500

C -0.39164300	1.83810900	1.37236900

C -0.79985900	0.54923000	0.84498900

C -0.06232700	3.01675200	0.75084400

H	1.78123800	-3.02273500	3.06786000

H	4.10230000	1.41198300	1.49377400

H	2.16981300	0.78192400	-3.31638100

H -0.18652100	-3.68331800	-1.85581000

C -0.23618300	3.34439300	-0.65618000

C -1.00240600	2.49571200	-1.50661700

C	0.35898900	4.50106300	-1.21181700

C -1.13543900	2.81904200	-2.87551200

C	0.21707100	4.80480100	-2.56847900

H	0.93171500	5.15314800	-0.54834500

C -0.53158300	3.96236400	-3.40570200

H -1.72549300	2.15522400	-3.51434600

H	0.69136500	5.70460300	-2.97324900

H -0.65221100	4.19676000	-4.46827700

C -1.69918200	1.31944900	-1.03475300

C -2.62636100	0.50156800	-1.25188500

C -3.62913400	-0.48128000	-1.33258400

C -3.40725900	-1.68347400	-2.06277900

C -4.89235400	-0.27413000	-0.71023000

C -4.41833500	-2.63945700	-2.16212900

H -2.43020200	-1.84066200	-2.52628500

C -5.88819000	-1.24728800	-0.80496600

H -5.06180300	0.64977400	-0.15128600

C -5.65743200	-2.42778500	-1.53182700

H -4.24226100	-3.56032000	-2.72722800

H -6.85401400	-1.08486300	-0.31629500

H -6.44516700	-3.18413600	-1.60885800

C -1.92678400	-0.10943000	1.55211300

C -2.90858200	0.68353800	2.20955600

C -2.08077700	-1.51596700	1.60278700

C -3.96028100	0.09989600	2.92061800

H -2.84502400	1.77475400	2.13738800

C -3.13349900	-2.09879800	2.31982600

H -1.35957500	-2.14656500	1.08467200

C -4.07083100	-1.29901600	2.98771600

H -4.69592100	0.73640400	3.42375700

H -3.22066000	-3.18977600	2.35506000

H -4.88918300	-1.76022700	3.55049200

**TS5**

Rh -3.38852100	-1.23226300	-0.06981100

Rh -1.10794100	-0.47000700	0.12990300

O -0.54141800	-2.44957900	0.37771100

O -0.93501700	-0.66971000	-1.92113100

O -1.81177600	1.44273000	-0.14551000

O -1.41891200	-0.30717200	2.15897500

O -3.98476600	0.71352500	-0.34186000

O -3.10285400	-1.42728700	-2.09850700

O -3.60492100	-1.00570100	1.95790800

O -2.72543800	-3.16022000	0.21020400

C -1.95779400	-1.09794200	-2.55965600

C -1.46870300	-3.33025600	0.36352800

C -2.57860700	-0.59994500	2.60658500

C -3.06814900	1.60490900	-0.31833100

C	0.20259400	4.07537900	-1.52142800

O -0.35658500	5.13880800	-1.30340600

O	0.40061100	3.60989100	-2.81776600

C	1.05910200	2.32901500	-2.79117500

H	0.39459000	1.57202700	-3.24753700

H	1.97185200	2.40146500	-3.40631700

C	1.30097000	2.02695300	-1.33146400

C	1.88414100	0.88471200	-0.73895300

C	0.76545500	3.07552000	-0.59447100

H -1.83301500	-1.19709900	-3.65471200

H -3.40011900	2.64922600	-0.46688800

H -2.71401200	-0.48937500	3.69899200

H -1.13979700	-4.37768800	0.50363200

C	0.72950500	3.06061500	0.83270500

C	1.18035200	1.86464900	1.48389900

C	0.24067100	4.14547700	1.60704500

C	1.09429300	1.78900500	2.89524300

C	0.18775000	4.04620000	2.99251800

H -0.09107800	5.04536600	1.08211000

C	0.60297500	2.85850100	3.63767500

H	1.40227300	0.86093200	3.38757200

H -0.19001100	4.88595200	3.58510900

H	0.52905200	2.77414700	4.72698500

C	1.77802100	0.76224400	0.70289200

C	2.15118300	-0.39601700	1.39859300

C	3.41112800	-1.02841300	1.47891900

C	3.50298900	-2.40146100	1.87918200

C	4.63227700	-0.29281800	1.31370900

C	4.74089500	-3.01159200	2.05153600

H	2.56910300	-2.95264300	2.02799200

C	5.86403600	-0.89409600	1.56437400

H	4.57323300	0.75769400	1.01209200

C	5.92419700	-2.25616200	1.91353800

H	4.79934500	-4.06855200	2.33060300

H	6.78821700	-0.31541400	1.46377400

H	6.89547600	-2.72998900	2.08990400

C	2.55039200	-0.14092800	-1.58163400

C	3.54146900	0.26114500	-2.50771300

C	2.24358500	-1.51607500	-1.48880600

C	4.20522600	-0.67768500	-3.30585900

H	3.82348100	1.31783400	-2.56671500

C	2.90858300	-2.45411300	-2.28914200

H	1.46007800	-1.85141700	-0.80268000

C	3.89002300	-2.04144500	-3.20144500

H	4.98058700	-0.34361800	-4.00335200

H	2.65147700	-3.51524300	-2.20165000

H	4.40733100	-2.77603000	-3.82714800

**^3^TS5**

Rh 3.27301300	-1.01068000	0.23938600

Rh 0.94799100	-0.37319300	0.07499000

O	0.48297000	-2.17294200	1.00621200

O	1.15338800	0.55447700	1.91238500

O	1.57224400	1.35769500	-0.83850200

O	0.88661700	-1.33131600	-1.74902900

O	3.78507400	0.74852600	-0.68221400

O	3.36226200	-0.07751400	2.06737000

O	3.10833500	-1.91915500	-1.59716600

O	2.70326000	-2.75771200	1.16337400

C	2.29681200	0.49231100	2.48238000

C	1.45272600	-2.94121000	1.33655900

C	1.96558000	-1.87345500	-2.16872900

C	2.82757900	1.53008400	-1.00970400

C -0.35626100	4.47324500	-0.45008300

O	0.08515500	5.27954800	-1.25364800

O -0.32442700	4.71894200	0.91815900

C -0.90308900	3.61336200	1.63808000

H -0.14581600	3.19549100	2.32643000

H -1.73976700	4.00213800	2.24284200

C -1.30432300	2.60879300	0.58379900

C -1.81664600	1.30640500	0.74296200

C -0.96882100	3.14558100	-0.65471700

H	2.37217100	0.99750400	3.46387500

H	3.11763800	2.47628200	-1.50296100

H	1.90035500	-2.36887500	-3.15577900

H	1.16831700	-3.88752400	1.83500700

C -1.15357500	2.42792400	-1.87746700

C -1.61952300	1.07495400	-1.78327500

C -0.87581700	2.99642400	-3.14769500

C -1.78735700	0.33723700	-2.97797000

C -1.06049100	2.24728700	-4.30389900

H -0.51298900	4.02726900	-3.18165000

C -1.51835300	0.91407500	-4.21738800

H -2.12987300	-0.69954200	-2.92391800

H -0.85028700	2.69151900	-5.28228000

H -1.66277100	0.32507200	-5.12907400

C -1.93111400	0.48260900	-0.45616100

C -2.38517600	-0.81242000	-0.35286200

C -3.03047100	-1.97039500	-0.77627400

C -2.41260000	-3.26060000	-0.65590600

C -4.36910400	-1.91130300	-1.29877300

C -3.09412100	-4.40913700	-1.05068400

H -1.40007400	-3.31321700	-0.24679300

C -5.03018600	-3.07520500	-1.68263800

H -4.85676900	-0.93600900	-1.38835400

C -4.40267100	-4.33010900	-1.56464500

H -2.60434700	-5.38433900	-0.95689000

H -6.04854300	-3.00869900	-2.08011900

H -4.93097800	-5.23922400	-1.86795700

C -2.31417600	0.83208400	2.05867000

C -3.19782500	1.65203800	2.79879300

C -1.96839900	-0.42953000	2.59470000

C -3.71125600	1.23232000	4.03227800

H -3.52065500	2.60987700	2.37791300

C -2.48095200	-0.84444900	3.83059500

H -1.26899400	-1.07607300	2.05961600

C -3.35143500	-0.01790100	4.55600700

H -4.40336300	1.88095300	4.57934700

H -2.19137700	-1.82179100	4.23132100

H -3.74982100	-0.34700200	5.52127200

**TS6**

Rh -3.36022800	-1.24228600	-0.64981000

Rh -1.28205800	-0.06101800	-0.03609500

O -0.46960900	-1.93677900	0.28473000

O -0.71527800	-0.21709600	-2.02767700

O -2.29586300	1.71070100	-0.49321200

O -2.00118400	-0.02460600	1.91396900

O -4.23144600	0.58567500	-1.03740700

O -2.66254900	-1.31589800	-2.59115900

O -3.94510700	-1.11836600	1.32164500

O -2.42619100	-3.02658600	-0.26424300

C -1.52359700	-0.80248000	-2.83284500

C -1.20657800	-2.96717800	0.10236000

C -3.14514500	-0.55716300	2.13858300

C -3.51529400	1.62380700	-0.88677600

C	0.57815600	1.08519100	0.50415000

H -0.70788300	-3.93762400	0.29078700

H -1.17431000	-0.86605300	-3.88192600

H -4.00278800	2.59027900	-1.12310000

H -3.48081400	-0.51794300	3.19356300

C	2.20561800	1.69182600	-1.57623600

C	2.90262900	2.38176300	-2.59344800

C	1.01785800	2.28166000	-1.11855400

C	2.42166400	3.61613100	-3.05092200

H	3.82961100	1.97985200	-3.01045400

C	0.52287600	3.51662300	-1.53024300

H	0.15663600	1.52311300	-0.67989100

C	1.25155200	4.19249800	-2.52179900

H	2.97927400	4.14959900	-3.82728200

H -0.40887400	3.91642900	-1.12104400

H	0.90184000	5.16270000	-2.88777700

C	1.74293400	-2.04743900	3.30504000

C	1.36193100	-1.05278100	2.41636000

C	2.10002000	-0.82476000	1.21854700

C	3.24216400	-1.66508700	0.95080300

C	3.60194100	-2.68541000	1.87138000

C	2.86525700	-2.86785300	3.03294300

H	1.16312300	-2.20376700	4.22057700

H	0.47781600	-0.44601700	2.62758800

C	1.77154100	0.23529500	0.29358500

C	3.98297800	-1.41471400	-0.24348500

H	4.47099000	-3.30666200	1.63886500

H	3.15233300	-3.65073100	3.74258900

C	3.67684400	-0.37414500	-1.10131500

C	2.58361000	0.48951000	-0.84082300

C	0.54129900	2.08831100	1.60172300

C	1.70900700	2.34006400	2.36496800

C -0.63884000	2.79838900	1.92728200

C	1.69165600	3.25619800	3.42125200

H	2.63325500	1.80660000	2.12487000

C -0.64427900	3.72888200	2.97160600

H -1.54592300	2.61538200	1.34845500

C	0.51609400	3.95871700	3.72696900

H	2.60355200	3.42863000	4.00211600

H -1.56660700	4.27114600	3.20514200

H	0.50397500	4.68061200	4.55007200

C	5.16615100	-2.13281900	-0.76791200

O	5.77290600	-3.09670400	-0.33522400

O	5.54843600	-1.50573600	-1.95174300

C	4.68521100	-0.38928600	-2.22162900

H	5.30936500	0.52395000	-2.25699100

H	4.22384400	-0.53013600	-3.21723700

**Rh_2_(*S*-TFPTTL)_4_**

Rh 0.00000000	0.00000000	0.25254200

Rh 0.00000000	0.00000000	2.63725000

N	4.35247500	1.35476400	0.40573800

O	2.92317000	2.89452500	1.43153300

O	5.54543100	0.20334400	-1.24491000

C	3.65804500	2.57334200	0.51217000

C	3.96850400	3.33090100	-0.73432500

C	3.57100300	4.60453900	-1.13232200

C	3.98648200	5.06135900	-2.40534500

C	4.78897900	4.24755300	-3.22924700

C	5.18998000	2.95531000	-2.81044900

C	4.77161400	2.51411900	-1.55711400

C	4.98394300	1.20847200	-0.85285300

N	1.35476400	-4.35247500	0.40573800

O	2.89452500	-2.92317000	1.43153300

O	0.20334400	-5.54543100	-1.24491000

C	2.57334200	-3.65804500	0.51217000

C	3.33090100	-3.96850400	-0.73432500

C	4.60453900	-3.57100300	-1.13232200

C	5.06135900	-3.98648200	-2.40534500

C	4.24755300	-4.78897900	-3.22924700

C	2.95531000	-5.18998000	-2.81044900

C	2.51411900	-4.77161400	-1.55711400

C	1.20847200	-4.98394300	-0.85285300

N -4.35247500	-1.35476400	0.40573800

O -2.92317000	-2.89452500	1.43153300

O -5.54543100	-0.20334400	-1.24491000

C -3.65804500	-2.57334200	0.51217000

C -3.96850400	-3.33090100	-0.73432500

C -3.57100300	-4.60453900	-1.13232200

C -3.98648200	-5.06135900	-2.40534500

C -4.78897900	-4.24755300	-3.22924700

C -5.18998000	-2.95531000	-2.81044900

C -4.77161400	-2.51411900	-1.55711400

C -4.98394300	-1.20847200	-0.85285300

N -1.35476400	4.35247500	0.40573800

O -2.89452500	2.92317000	1.43153300

O -0.20334400	5.54543100	-1.24491000

C -2.57334200	3.65804500	0.51217000

C -3.33090100	3.96850400	-0.73432500

C -4.60453900	3.57100300	-1.13232200

C -5.06135900	3.98648200	-2.40534500

C -4.24755300	4.78897900	-3.22924700

C -2.95531000	5.18998000	-2.81044900

C -2.51411900	4.77161400	-1.55711400

C -1.20847200	4.98394300	-0.85285300

O	2.03192900	0.00603600	0.30362800

O	2.05624900	0.00000000	2.57729500

O	0.00603600	-2.03192900	0.30362800

O	0.00000000	-2.05624900	2.57729500

O -2.03192900	-0.00603600	0.30362800

O -2.05624900	0.00000000	2.57729500

O -0.00603600	2.03192900	0.30362800

O	0.00000000	2.05624900	2.57729500

C	2.61987100	0.05608600	1.44000400

C	4.14549500	0.21702000	1.30910900

C	5.03191100	0.22667900	2.59120900

C	6.50041000	0.34749200	2.13077300

C	4.69791900	1.38678300	3.54746600

C	4.85158900	-1.12674800	3.31037600

C	0.05608600	-2.61987100	1.44000400

C	0.21702000	-4.14549500	1.30910900

C	0.22667900	-5.03191100	2.59120900

C	1.38678300	-4.69791900	3.54746600

C	0.34749200	-6.50041000	2.13077300

C -1.12674800	-4.85158900	3.31037600

C -2.61987100	-0.05608600	1.44000400

C -4.14549500	-0.21702000	1.30910900

C -5.03191100	-0.22667900	2.59120900

C -4.85158900	1.12674800	3.31037600

C -6.50041000	-0.34749200	2.13077300

C -4.69791900	-1.38678300	3.54746600

C -0.05608600	2.61987100	1.44000400

C -0.21702000	4.14549500	1.30910900

C -0.22667900	5.03191100	2.59120900

C	1.12674800	4.85158900	3.31037600

C -0.34749200	6.50041000	2.13077300

C -1.38678300	4.69791900	3.54746600

H -4.46174500	0.66322500	0.70905800

H -6.77208300	0.45796200	1.41823000

H -7.17729500	-0.27756900	3.00590800

H -6.69521200	-1.32046700	1.63519700

H -5.33610200	-1.31470100	4.45207100

H -4.88944600	-2.37309100	3.08114100

H -3.63843700	-1.36052700	3.86190000

H -5.53890500	1.17905800	4.17899900

H -3.81680100	1.26183700	3.67568000

H -5.07999100	1.97425600	2.63400700

H -1.26183700	-3.81680100	3.67568000

H -1.17905800	-5.53890500	4.17899900

H -1.97425600	-5.07999100	2.63400700

H -0.45796200	-6.77208300	1.41823000

H	0.27756900	-7.17729500	3.00590800

H	1.32046700	-6.69521200	1.63519700

H	1.36052700	-3.63843700	3.86190000

H	2.37309100	-4.88944600	3.08114100

H	1.31470100	-5.33610200	4.45207100

H -0.66322500	-4.46174500	0.70905800

H	3.81680100	-1.26183700	3.67568000

H	5.53890500	-1.17905800	4.17899900

H	5.07999100	-1.97425600	2.63400700

H	4.88944600	2.37309100	3.08114100

H	5.33610200	1.31470100	4.45207100

H	3.63843700	1.36052700	3.86190000

H	6.77208300	-0.45796200	1.41823000

H	7.17729500	0.27756900	3.00590800

H	6.69521200	1.32046700	1.63519700

H	4.46174500	-0.66322500	0.70905800

H -2.37309100	4.88944600	3.08114100

H -1.36052700	3.63843700	3.86190000

H -1.31470100	5.33610200	4.45207100

H	1.17905800	5.53890500	4.17899900

H	1.26183700	3.81680100	3.67568000

H	1.97425600	5.07999100	2.63400700

H -1.32046700	6.69521200	1.63519700

H	0.45796200	6.77208300	1.41823000

H -0.27756900	7.17729500	3.00590800

H	0.66322500	4.46174500	0.70905800

F -2.81923100	-5.39845300	-0.37231900

F -3.63051000	-6.26844700	-2.83198700

F -5.16777200	-4.70427000	-4.42056700

F -5.94236200	-2.22328300	-3.62619200

F	2.22328300	-5.94236200	-3.62619200

F	4.70427000	-5.16777200	-4.42056700

F	6.26844700	-3.63051000	-2.83198700

F	5.39845300	-2.81923100	-0.37231900

F	5.94236200	2.22328300	-3.62619200

F	5.16777200	4.70427000	-4.42056700

F	3.63051000	6.26844700	-2.83198700

F	2.81923100	5.39845300	-0.37231900

F -2.22328300	5.94236200	-3.62619200

F -4.70427000	5.16777200	-4.42056700

F -6.26844700	3.63051000	-2.83198700

F -5.39845300	2.81923100	-0.37231900

**TS-pro-S**

Rh -1.09117400	0.05124600	-0.80368500

Rh -2.05345700	0.45229200	-3.03130400

N -0.58026200	4.66680600	-0.63812700

O	0.48068900	3.84272700	-2.55022700

O -0.95296300	5.40686200	1.55036400

C	0.52545100	4.38313400	-1.45670200

C	1.72857100	4.80091000	-0.67987300

C	3.07833000	4.73681900	-1.01694000

C	4.02497600	5.05025800	-0.01508900

C	3.60359900	5.45271100	1.26628000

C	2.22727200	5.55877300	1.58192000

C	1.30245300	5.23527600	0.59182400

C -0.19285500	5.15157400	0.63715200

N -5.32637600	0.28890900	0.86399000

O -4.59650600	2.29894700	-0.07578000

O -5.69536300	-1.44216400	2.39158900

C -4.82337200	1.60537200	0.90013800

C -4.58398200	1.91646300	2.33818800

C -4.05098800	3.05659800	2.93448900

C -3.82908000	3.03219300	4.33202700

C -4.15355300	1.88867600	5.08858600

C -4.70765600	0.74278700	4.47116100

C -4.91499800	0.77408900	3.09470600

C -5.37672800	-0.29150800	2.15090400

N -1.47110700	-4.41413400	-1.58689000

O -3.54002800	-3.34657300	-1.31772900

O	0.55998400	-5.46107200	-1.09927900

C -2.63676900	-4.04050800	-0.88258400

C -2.48191000	-4.60992800	0.48815600

C -3.30788200	-4.50763600	1.60335300

C -2.84356800	-5.03874000	2.82895000

C -1.58581300	-5.66519200	2.91032800

C -0.76427900	-5.77875200	1.76410100

C -1.22520500	-5.24219400	0.56525600

C -0.55740400	-5.10792300	-0.76516100

N	3.13732300	0.20472000	-2.64865600

O	2.06143900	-1.81830500	-3.13524200

O	4.44446400	1.78572700	-1.52789300

C	2.99086400	-1.19706100	-2.64530100

C	4.18811600	-1.71780800	-1.91329000

C	4.62362000	-3.01992900	-1.69270600

C	5.82911100	-3.21354100	-0.97785400

C	6.56880000	-2.11350500	-0.50967900

C	6.09724700	-0.79462300	-0.70468400

C	4.91815600	-0.61601300	-1.42191200

C	4.20093200	0.62714000	-1.82636500

O -1.48448800	2.02128200	-0.41626300

O -2.32069800	2.41096500	-2.49036000

O -3.00687700	-0.42872100	-0.23418000

O -3.91020300	0.00257300	-2.27423800

O -0.78100400	-1.86928600	-1.44262600

O -1.73751600	-1.51706100	-3.47808800

O	0.69517700	0.63866100	-1.59447500

O -0.15252200	0.84584700	-3.69318200

C -1.93598500	2.78666800	-1.34485900

C -1.96153100	4.27689900	-0.94273500

C -2.73037400	5.28853000	-1.85229200

C -2.67055000	6.66525500	-1.15586500

C -2.13460800	5.41084900	-3.26732100

C -4.20514000	4.84386800	-1.93311700

C -3.98721300	-0.29301500	-1.04725600

C -5.34975100	-0.52694900	-0.35855500

C -6.65092200	-0.42449000	-1.20739800

C -6.84577100	0.96340000	-1.84554600

C -7.83738500	-0.72934400	-0.26874600

C -6.58792600	-1.51421900	-2.29944300

C -1.20916800	-2.24709400	-2.58860500

C -1.02049600	-3.75834000	-2.82065900

C -1.53930700	-4.39133000	-4.14605100

C -0.76149100	-3.74840100	-5.31496600

C -1.20464800	-5.89698200	-4.09535800

C -3.05543400	-4.21031000	-4.34673600

C	0.79594600	0.84158900	-2.85295100

C	2.23411500	1.15442600	-3.29619000

C	2.52255400	1.36468600	-4.82105300

C	1.74204400	2.60720000	-5.30080600

C	4.03112300	1.65615100	-4.97463000

C	2.14840000	0.13735500	-5.67379800

H	0.08373100	-3.90042100	-2.81136200

H -0.12180600	-6.06995700	-3.92989200

H -1.49139900	-6.37961800	-5.05123600

H -1.75816600	-6.40885900	-3.28199100

H -3.35846500	-4.67105300	-5.30933800

H -3.63596800	-4.70023400	-3.54064000

H -3.33812500	-3.14146000	-4.36546900

H -1.01959400	-4.26134200	-6.26353000

H -1.00455500	-2.67517700	-5.42415300

H	0.33493200	-3.83719800	-5.16678300

H -5.77304000	-1.32060500	-3.02150600

H -7.54651500	-1.54223300	-2.85601700

H -6.41881400	-2.51811000	-1.85829800

H -7.72095100	-1.71181600	0.23236000

H -8.78273600	-0.74522400	-0.84761200

H -7.94172800	0.04260300	0.52082500

H -6.00144700	1.22604300	-2.50925100

H -6.93248700	1.75745400	-1.07836400

H -7.77992200	0.96802400	-2.44399000

H -5.29393600	-1.57353000	0.01404600

H -4.31314500	3.86677900	-2.43927200

H -4.78739400	5.59717700	-2.50153300

H -4.65150400	4.74859100	-0.92368600

H -1.09926500	5.80380700	-3.24608100

H -2.74700800	6.11931700	-3.86232300

H -2.11774900	4.43580000	-3.78674600

H -3.06886300	6.61975700	-0.12201400

H -3.27128200	7.40123000	-1.72722200

H -1.63285200	7.05328500	-1.10327300

H -2.46543000	4.28683400	0.04660000

H	2.74266900	-0.75584500	-5.39899900

H	1.07841300	-0.11968200	-5.56377000

H	2.34749500	0.35613100	-6.74305300

H	2.00047800	2.81922100	-6.35833900

H	0.64973900	2.45641300	-5.23064400

H	1.99758700	3.49783300	-4.69342200

H	4.65279400	0.77984600	-4.69938600

H	4.34673000	2.51263400	-4.34490300

H	4.25803700	1.90339200	-6.03107600

H	2.45804400	2.12177900	-2.79764300

F -4.48576300	-3.89175100	1.57707400

F -3.58674900	-4.92164300	3.92727500

F -1.16802000	-6.14165500	4.07978700

F	0.43283700	-6.35399300	1.88558800

F -4.97863900	-0.32213200	5.22331200

F -3.92382400	1.88968900	6.39972600

F -3.31089900	4.09168900	4.94447100

F -3.72686600	4.14990300	2.24865900

F	1.87903200	5.94474500	2.80580400

F	4.52011500	5.72739100	2.19248100

F	5.32829600	4.96646000	-0.27293600

F	3.50195300	4.39095200	-2.22983100

F	6.80498700	0.21574300	-0.20028300

F	7.70666400	-2.32290400	0.14624200

F	6.26686300	-4.44554800	-0.72744000

F	3.93716600	-4.08895600	-2.09818400

C	1.13730700	-1.13734400	0.74295400

O	2.17682600	-0.68706100	0.29647200

O	0.93443700	-2.45040700	1.01786400

C	2.02703200	-3.34791300	0.68066600

H	2.19511800	-3.30462500	-0.41638500

H	1.63783000	-4.35730000	0.90930000

C -0.15054500	-0.38662300	1.02256000

C -0.75488400	-0.72468100	2.34324800

C -2.04049600	-1.30360500	2.40162700

C -0.01018700	-0.59410200	3.57560000

C -2.58833900	-1.75356600	3.61143500

C -0.60196800	-1.01778400	4.79194400

C -1.87332800	-1.59981100	4.81062700

H -3.59071600	-2.20605400	3.61363800

H -0.03006800	-0.89175600	5.72262500

H -2.31330700	-1.93190900	5.76303100

N	0.46869000	2.38273200	1.27522000

N	0.59310700	1.27073400	1.35493400

H -2.60641900	-1.41432400	1.46923000

C	3.26054900	-3.09972700	1.41071500

C	4.34312200	-2.93283100	1.96546800

C	5.63470400	-2.70963100	2.53232600

C	6.08370400	-1.38898300	2.79044500

C	6.51141400	-3.79527200	2.78393200

C	7.37283700	-1.16625100	3.28739400

H	5.41479700	-0.54317000	2.58343700

C	7.79959100	-3.55986600	3.27939900

H	6.17140400	-4.81902700	2.57002500

C	8.23496100	-2.24799700	3.53301700

H	7.70486700	-0.13366300	3.47472000

H	8.47411100	-4.40974500	3.46482200

H	9.25046300	-2.06922000	3.91827000

C	1.28499300	-0.01097600	3.58639900

C	2.37604100	0.56454500	3.51779600

C	3.56968500	1.30449100	3.28819800

C	4.34888700	1.82032700	4.35642600

C	3.98026600	1.55704700	1.94881800

C	5.49455700	2.57892400	4.08682100

H	4.03971400	1.62155200	5.39286200

C	5.12898000	2.31466600	1.69309200

H	3.39483800	1.13285600	1.12072800

C	5.88492200	2.83319400	2.75958400

H	6.08857700	2.98032900	4.92202500

H	5.42895400	2.48661000	0.64911500

H	6.77853000	3.44157400	2.55364100

**TS-pro-R**

Rh 0.81283300	-0.07818300	-1.04431200

Rh 2.27147000	0.15690000	-2.99934800

N	2.90807200	3.65345700	0.64603800

O	4.54208000	2.02526300	0.26084400

O	1.16664700	4.82490300	1.67985100

C	3.87248400	2.70066700	1.02511000

C	3.82082600	2.63400900	2.51174700

C	4.56821100	1.86238800	3.39566400

C	4.18213700	1.85510500	4.75631300

C	3.09339000	2.63639500	5.19314500

C	2.36302900	3.44383200	4.28694700

C	2.74310200	3.43244100	2.94729400

C	2.13727100	4.09092400	1.74721300

N -2.11347500	2.68889700	-3.23646700

O -0.03499300	3.69785800	-2.90216600

O -4.30187700	1.95556200	-2.87139000

C -1.22169500	3.60183600	-2.64051400

C -2.01174500	4.34581300	-1.61990700

C -1.62678900	5.35343300	-0.74166100

C -2.57124700	5.80731400	0.20658900

C -3.86069100	5.24528800	0.25768000

C -4.24315800	4.23061000	-0.65087700

C -3.30974400	3.80015200	-1.59031800

C -3.38045600	2.71135300	-2.61515500

N -0.91761300	-3.84933000	-2.86453600

O -1.60368300	-1.91980400	-4.00435900

O -0.77032900	-5.52845400	-1.24728900

C -1.81159800	-2.80245500	-3.18988900

C -2.99506000	-2.98882000	-2.30094200

C -4.15725600	-2.23254000	-2.17910200

C -5.08876200	-2.59728200	-1.17956500

C -4.83476000	-3.68839400	-0.32791100

C -3.65302500	-4.45300600	-0.46391300

C -2.74704900	-4.09114500	-1.45794300

C -1.39510800	-4.63374000	-1.79089200

N	4.09344600	-2.40888300	1.12628700

O	3.22603200	-3.68364400	-0.63744200

O	4.24879100	-1.38009200	3.22424900

C	3.36340100	-3.47146900	0.55621800

C	2.75401500	-4.20246900	1.70679200

C	1.95454000	-5.34021200	1.72697200

C	1.40241400	-5.74150000	2.96518800

C	1.67105700	-5.01822500	4.14124100

C	2.50223500	-3.87275200	4.11214200

C	3.03389500	-3.48137700	2.88568100

C	3.86380500	-2.29341500	2.51739400

O	1.32533000	1.84361100	-0.56978600

O	2.65302400	2.07891400	-2.40182600

O -0.69376900	0.61955300	-2.23251100

O	0.63933500	0.81143700	-4.06743400

O	0.45904600	-1.96766200	-1.73262700

O	1.83806400	-1.77995300	-3.52802100

O	2.42944500	-0.71181800	0.00370400

O	3.80436600	-0.51280400	-1.79753400

C	2.14600800	2.48839700	-1.31627100

C	2.48896600	3.87869500	-0.74206000

C	3.40894300	4.84917100	-1.54840500

C	3.50440900	6.15803200	-0.73492600

C	4.82392400	4.29287100	-1.79473200

C	2.72920400	5.16088200	-2.89789100

C -0.45593600	0.95264600	-3.44399100

C -1.69118000	1.58983700	-4.11483000

C -1.62718600	1.94288000	-5.63063900

C -0.54662000	2.98962900	-5.95893900

C -3.01125300	2.49320300	-6.03536700

C -1.36417900	0.63602100	-6.40904200

C	0.99584800	-2.39689700	-2.81368500

C	0.51552200	-3.81466000	-3.18203500

C	0.92059300	-4.41591700	-4.56155100

C	2.45540700	-4.58544100	-4.57180000

C	0.26657400	-5.80958500	-4.66347400

C	0.47281900	-3.54720200	-5.75142400

C	3.56448600	-0.80863800	-0.59170200

C	4.67370400	-1.31130300	0.34840900

C	6.09940000	-1.58818400	-0.22323500

C	6.68246900	-0.25508200	-0.73891100

C	6.97077500	-2.08199800	0.95152100

C	6.10483100	-2.63788000	-1.34961400

H	0.97299900	-4.46948200	-2.40483100

H	0.54202600	-6.45343600	-3.80321200

H	0.59753800	-6.31433200	-5.59327400

H -0.83950600	-5.73832400	-4.69422100

H	0.80256500	-4.01935900	-6.69966400

H -0.62828000	-3.44017500	-5.78874900

H	0.90835800	-2.53175100	-5.69812800

H	2.76826800	-5.10688800	-5.49902100

H	2.97243400	-3.60888000	-4.53190200

H	2.80024300	-5.19092400	-3.70776300

H -0.36354000	0.22294500	-6.18319100

H -1.42170600	0.83249500	-7.49876800

H -2.11434200	-0.14131500	-6.15801200

H -3.82342700	1.77839100	-5.79235600

H -3.03552600	2.68870800	-7.12639800

H -3.23728000	3.44940700	-5.52060600

H	0.45901300	2.64222500	-5.65940800

H -0.74056100	3.95237300	-5.44611300

H -0.54229100	3.18707100	-7.05067300

H -2.49068300	0.82701400	-3.99277300

H	2.63233200	4.25601400	-3.52547600

H	3.33483400	5.90843100	-3.44949100

H	1.71286900	5.57445900	-2.75014500

H	5.38456800	4.14684800	-0.85130100

H	5.39615600	5.01271400	-2.41552200

H	4.79092300	3.32202300	-2.32276100

H	2.50051400	6.58484300	-0.53305700

H	4.09147600	6.91043300	-1.29890900

H	4.01126200	6.00072400	0.23894100

H	1.50368800	4.38084000	-0.64469100

H	5.75141800	-3.62617000	-0.99737000

H	5.45947800	-2.32760000	-2.19263400

H	7.13919300	-2.76492800	-1.73023800

H	7.72814600	-0.41425700	-1.07193200

H	6.10091200	0.14025700	-1.59178900

H	6.68206400	0.51791900	0.05477800

H	6.61942200	-3.05944800	1.34035100

H	6.96694900	-1.35778700	1.79205800

H	8.01866700	-2.21278700	0.61447500

H	4.78758900	-0.48859500	1.08775600

F -4.41438700	-1.16841900	-2.93732500

F -6.21065500	-1.89620400	-1.02831000

F -5.71871500	-3.99753700	0.61895500

F -3.44823800	-5.46989300	0.37116100

F -5.46434900	3.70633400	-0.55150300

F -4.72305500	5.67592600	1.17615500

F -2.24632300	6.77554100	1.06099300

F -0.41259100	5.90303100	-0.75643400

F	1.33719100	4.15791900	4.74549700

F	2.75299300	2.61246900	6.47959100

F	4.85041300	1.12081600	5.63781500

F	5.61249500	1.13604000	3.00953200

F	2.72376500	-3.21249400	5.24477400

F	1.11641000	-5.40862700	5.28662200

F	0.57595800	-6.78467400	3.01523900

F	1.63458000	-6.02107800	0.63056500

C -1.61032100	0.71490300	0.37254500

O -1.58345400	1.92974500	0.44192100

O -2.68247800	-0.02253400	-0.02984900

C -3.98574200	0.61906900	0.00102200

H -3.84149700	1.69932100	0.20655800

H -4.43607000	0.51838300	-1.00628900

C -0.46114000	-0.24120400	0.61521600

C -0.82684800	-1.46765200	1.38921100

C -0.41893000	-2.74891800	0.96745500

C -1.68419600	-1.36897400	2.54815500

C -0.92149500	-3.90629600	1.58064300

C -2.15431900	-2.54884500	3.17183700

C -1.79235800	-3.80708000	2.67796600

H -0.65881300	-4.89051500	1.16807000

H -2.82291100	-2.45797100	4.04003100

H -2.18877600	-4.71852000	3.15055700

N	1.47311100	0.60991900	2.35592500

N	0.49064400	0.63303400	1.80452300

H	0.24699700	-2.82982600	0.10032500

C -4.79242200	-0.01486500	1.03152200

C -5.39736600	-0.59857900	1.92595700

C -6.06530200	-1.27735000	2.98945100

C -5.61507900	-1.12427800	4.32687500

C -7.17060600	-2.12624700	2.72933600

C -6.25598500	-1.80404300	5.37014200

H -4.75129800	-0.47161800	4.52553300

C -7.80622100	-2.79783600	3.78066900

H -7.50818400	-2.25585600	1.69137500

C -7.35322100	-2.64057100	5.10178100

H -5.89729200	-1.67913000	6.40342400

H -8.66310200	-3.45499700	3.56711700

H -7.85576800	-3.17218800	5.92424200

C -2.04125900	-0.08971100	3.04940500

C -2.29977400	1.07549900	3.36607400

C -2.54554200	2.45872000	3.58831300

C -3.78230100	2.92471000	4.10533500

C -1.54998000	3.40329900	3.22288900

C -4.01088300	4.29922300	4.24013900

H -4.56277300	2.19744800	4.37396800

C -1.78487400	4.77282100	3.37207900

H -0.60322500	3.05494300	2.79208200

C -3.01674200	5.22519100	3.87611300

H -4.97867200	4.65381000	4.62602500

H -0.99764800	5.47827600	3.06960200

H -3.20848800	6.30453200	3.97144200

**TS-S**

Rh -0.23138400	0.49964400	0.55842300

Rh -0.39505200	0.99124500	2.96371400

N -3.93966000	3.36915100	0.26745600

O -4.33314600	1.66574600	1.82269500

O -3.91778100	4.58786000	-1.73011300

C -4.59738700	2.23257400	0.77735800

C -5.62601000	1.86737200	-0.24224800

C -6.56059300	0.83515500	-0.24613600

C -7.40487700	0.70659800	-1.37428100

C -7.29681500	1.60554300	-2.45427900

C -6.33982500	2.65013800	-2.44019500

C -5.51408800	2.76584400	-1.32357700

C -4.39149000	3.71475300	-1.02929800

N	3.25350100	3.45428000	0.06752300

O	1.30133900	4.74715700	-0.06558500

O	5.00720000	2.03076400	-0.53863700

C	2.25093200	4.19946500	-0.59497900

C	2.58300700	4.10984600	-2.05222700

C	1.93252400	4.64150000	-3.16377300

C	2.41171800	4.28525500	-4.44746800

C	3.51868200	3.42696700	-4.59148400

C	4.18672600	2.91450100	-3.45521100

C	3.69883300	3.26085200	-2.19819300

C	4.12219500	2.81512600	-0.83936600

N	3.46276600	-2.10796200	1.97655700

O	3.69626100	0.08073300	2.77278300

O	3.73591500	-4.04824600	0.70389200

C	4.10655400	-0.86367500	2.11873400

C	5.32616300	-0.93634900	1.26300300

C	6.34156100	-0.00582500	1.06593000

C	7.33694200	-0.30554300	0.10658800

C	7.31250700	-1.52848100	-0.59196000

C	6.29699000	-2.48677100	-0.35082800

C	5.31238000	-2.17195700	0.58286000

C	4.11430100	-2.94041800	1.04097100

N -3.22590500	-2.94272700	1.24027600

O -1.12902100	-3.29389700	2.21344200

O -4.96261800	-2.91159100	-0.32513800

C -1.98061000	-3.57839300	1.38702000

C -1.91335400	-4.59746400	0.30301200

C -0.89321600	-5.48599400	-0.02188800

C -1.03116500	-6.25412200	-1.19975800

C -2.17563800	-6.11713800	-2.00797000

C -3.21913100	-5.23167800	-1.64831500

C -3.06956600	-4.47052600	-0.49320300

C -3.91689600	-3.37928300	0.08676800

O -1.44193900	2.12361800	0.29287000

O -1.49941500	2.65186500	2.49991800

O	1.41587700	1.71387500	0.53030100

O	1.30085000	2.14110600	2.76199500

O	0.99042800	-1.08522300	1.11435400

O	0.72019300	-0.67383800	3.33651100

O -1.89589600	-0.67540600	0.81051100

O -2.07183300	-0.18530300	3.02786700

C -1.81835100	2.83498700	1.29132100

C -2.75439700	3.98534000	0.87442100

C -3.05081200	5.10295600	1.92041800

C -3.89720800	6.18797000	1.22244600

C -3.81354600	4.58206300	3.15274100

C -1.69692800	5.71688300	2.33928300

C	1.83303500	2.25055400	1.61890500

C	3.16503000	3.00776600	1.45851900

C	3.58322300	4.05580200	2.54262400

C	2.49621600	5.10959700	2.82138400

C	4.86982600	4.74482500	2.04139900

C	3.91738100	3.29208500	3.84358500

C	1.20793300	-1.31054000	2.35893100

C	2.19203700	-2.47265500	2.61324800

C	2.35301400	-3.01539500	4.07203600

C	0.97620300	-3.50627900	4.56496300

C	3.31196000	-4.22457100	4.01400700

C	2.92417900	-1.96959300	5.04863700

C -2.42684000	-0.78647400	1.97129500

C -3.62740400	-1.75590300	1.99733300

C -4.31431700	-2.07308300	3.36579800

C -4.90097600	-0.76817000	3.94454100

C -5.48129400	-3.04297400	3.08048800

C -3.34880700	-2.71642700	4.37861300

H	1.80441700	-3.31247900	1.99784600

H	2.96017200	-4.99586400	3.29971700

H	3.38688800	-4.68935500	5.01759800

H	4.33690600	-3.92529800	3.71321500

H	2.98979900	-2.41470200	6.06266800

H	3.94638500	-1.65196800	4.76259100

H	2.28741000	-1.06819800	5.09944900

H	1.08664800	-3.96343900	5.56917500

H	0.24715700	-2.67832400	4.63686700

H	0.55052800	-4.26707300	3.88157700

H	3.02774900	2.77849200	4.25066800

H	4.29243000	4.00538300	4.60538500

H	4.69866500	2.52529000	3.66909000

H	5.65736800	4.00105200	1.79946400

H	5.26627200	5.41838500	2.82758200

H	4.68560700	5.35754400	1.13590200

H	1.55616400	4.63336900	3.16042900

H	2.26348600	5.71427900	1.92506700

H	2.84179700	5.79485600	3.62252500

H	3.92444300	2.19724400	1.52105800

H -1.08341400	4.99878900	2.91525400

H -1.87008400	6.60833900	2.97523700

H -1.10457100	6.03729000	1.45687800

H -4.80678600	4.17569600	2.87461000

H -3.98499200	5.41712100	3.86261400

H -3.25285000	3.78524000	3.67389900

H -3.39693000	6.58350800	0.31579300

H -4.07203600	7.03205500	1.91942700

H -4.89036100	5.79864400	0.91855900

H -2.24059100	4.47370200	0.01805400

H -2.97342400	-3.69729100	4.02663200

H -2.47478300	-2.06709500	4.57099300

H -3.87916500	-2.88623700	5.33798300

H -5.48308200	-1.00123500	4.85938200

H -4.10938300	-0.04412100	4.20812100

H -5.57815900	-0.27547700	3.21959400

H -5.12249900	-4.01940300	2.69604100

H -6.18744100	-2.62209600	2.33606100

H -6.04226200	-3.24005600	4.01607700

H -4.39870800	-1.27383900	1.35799500

F	6.41568500	1.13839400	1.73787500

F	8.31391000	0.56222200	-0.13406500

F	8.26628800	-1.78603700	-1.48429800

F	6.32097700	-3.63091900	-1.02596000

F	5.23654800	2.11580400	-3.63293700

F	3.93676900	3.10084700	-5.81255700

F	1.80800200	4.75597000	-5.53639900

F	0.87942300	5.44530400	-3.07344500

F -6.27060200	3.46875800	-3.48419300

F -8.10782800	1.46418400	-3.49937000

F -8.31287300	-0.26210200	-1.42255600

F -6.68273500	-0.03709500	0.75287700

F -4.27180900	-5.12470200	-2.45964200

F -2.26526800	-6.82023700	-3.13520200

F -0.06612300	-7.09064100	-1.57073900

F	0.21426600	-5.61027900	0.70776400

C -0.12726200	0.09321600	-1.55052500

C -1.36607000	-0.58950100	-2.06148200

C -2.58940400	0.12556000	-1.96184400

C -1.40312500	-1.90217700	-2.62896200

C -3.79128600	-0.37135400	-2.46086300

C -2.64067000	-2.37476100	-3.13777800

C -3.82072700	-1.63461000	-3.07025500

H -4.70884900	0.22441500	-2.36583600

H -2.66900000	-3.38275200	-3.57491100

H -4.76061000	-2.05871200	-3.45148400

N -0.32313900	2.81736600	-2.07562200

N -0.32932200	1.69226600	-2.13188300

H -2.58002700	1.10686000	-1.46772100

C -0.26107200	-2.85251600	-2.69863400

C -0.05666000	-3.60895900	-3.87831400

C	0.58290100	-3.10455000	-1.59270800

C	0.94820100	-4.57948600	-3.96212600

H -0.68615900	-3.41491100	-4.75985500

C	1.59716300	-4.06991800	-1.66272100

H	0.44197300	-2.55059200	-0.65181200

C	1.76511600	-4.79827400	-2.84583200

H	1.11404200	-5.16282700	-4.87909400

H	2.25378100	-4.28074500	-0.80748900

C	1.18964900	-0.17640700	-2.24990000

C	1.22470500	-0.23901400	-3.66451800

C	2.40462100	-0.28296800	-1.54261300

C	2.43175600	-0.41942500	-4.34868000

H	0.28357400	-0.14372600	-4.22879100

C	3.61341200	-0.47056600	-2.22935900

H	2.39435400	-0.22787500	-0.44906700

C	3.63385000	-0.54109500	-3.62968800

H	2.43719400	-0.46305300	-5.44852800

H	4.55174100	-0.52976000	-1.66059800

H	4.58708100	-0.67321500	-4.16286400

F	2.73193600	-5.73004600	-2.91505300

**TS-R**

Rh 0.32629200	-0.13990200	-0.79721000

Rh 0.54013700	-1.09849900	-3.05170100

N -3.18955400	-3.37130700	-0.49423700

O -3.50094400	-2.10603500	-2.43761200

O -3.43269300	-4.20198000	1.67990100

C -3.90099700	-2.52585000	-1.36347800

C -5.18538700	-2.21804400	-0.67147100

C -6.27567100	-1.46544200	-1.09622400

C -7.32990600	-1.24883000	-0.18007900

C -7.28145700	-1.81019700	1.11044500

C -6.18402100	-2.60706500	1.51423700

C -5.14422100	-2.80276600	0.61037500

C -3.85732700	-3.55741400	0.73977100

N	3.62046100	-2.94423400	0.70088200

O	1.56192400	-4.04846400	0.50343100

O	5.33936300	-1.60121900	1.54271200

C	2.40297000	-3.48419700	1.17832900

C	2.35281500	-3.14771500	2.63164000

C	1.31859900	-3.31300100	3.54901700

C	1.43883300	-2.67738800	4.80617600

C	2.60248200	-1.95959700	5.13861600

C	3.67315000	-1.85301800	4.22007400

C	3.51432600	-2.42027600	2.95836900

C	4.32181600	-2.25375500	1.71040400

N	4.02420500	2.39183100	-2.09002000

O	4.49485200	0.15889200	-2.62163400

O	3.88514600	4.40028000	-0.89628000

C	4.71294300	1.16886800	-1.97394000

C	5.69369100	1.35877000	-0.86310400

C	6.64777800	0.48442200	-0.35040700

C	7.40292900	0.90378700	0.76941800

C	7.21030400	2.18743400	1.31718800

C	6.25276000	3.07820200	0.77253900

C	5.50404800	2.64655400	-0.32013700

C	4.39940900	3.31445100	-1.07887200

N -3.46939000	2.22277100	-2.01438100

O -1.65542400	3.63155200	-2.49115700

O -5.25175900	1.20005300	-0.89379300

C -2.61855100	3.33208700	-1.81036400

C -3.12861200	4.00356300	-0.56933500

C -2.62044300	5.08028300	0.15488500

C -3.24392900	5.40482500	1.38316500

C -4.34993600	4.67270500	1.85370100

C -4.86135800	3.58727500	1.10731100

C -4.23541200	3.27080500	-0.09547400

C -4.44395000	2.10625600	-1.00505600

O -0.82148900	-1.80278400	-0.35192600

O -0.47972600	-2.73664600	-2.39996100

O	2.02807900	-1.20557400	-0.35852100

O	2.24875900	-2.06775000	-2.45114600

O	1.48860200	1.39467500	-1.44346400

O	1.59103900	0.59796300	-3.57036500

O -1.38294100	0.77884900	-1.44652500

O -1.20980000	-0.13075300	-3.52122500

C -0.96937000	-2.72173200	-1.22912400

C -1.84759000	-3.89873700	-0.75186600

C -1.83690400	-5.21801100	-1.59250700

C -2.68108600	-6.26328900	-0.83216100

C -2.41974800	-5.03994700	-3.00746300

C -0.38275500	-5.72562300	-1.67508800

C	2.59950300	-1.93802200	-1.24370900

C	3.86288300	-2.65313300	-0.71696900

C	4.48666000	-3.81617700	-1.55110400

C	3.48675400	-4.94964800	-1.83809300

C	5.68394100	-4.36292700	-0.74499300

C	5.01740000	-3.23197800	-2.87898400

C	1.88750200	1.42809700	-2.66469500

C	2.83820000	2.61076100	-2.92717100

C	3.12100400	3.01816600	-4.40331100

C	1.75835900	3.32818900	-5.06132300

C	3.96998400	4.30618600	-4.37289300

C	3.86834300	1.93530100	-5.20259400

C -1.80099900	0.55853700	-2.63810200

C -3.20326000	1.11998600	-2.93837200

C -3.60723200	1.37679700	-4.43042400

C -3.78456100	0.00896500	-5.12611100

C -4.97459000	2.09295600	-4.42115200

C -2.57467400	2.22631900	-5.19423500

H	2.34309000	3.48155500	-2.44565000

H	3.47154000	5.11537900	-3.80171100

H	4.14479000	4.66793400	-5.40603500

H	4.96213700	4.12660700	-3.91054700

H	4.02377000	2.28703400	-6.24320300

H	4.86656100	1.72589800	-4.76967200

H	3.30388200	0.98569200	-5.23078200

H	1.91625100	3.74166600	-6.07770400

H	1.13367200	2.41936200	-5.15569300

H	1.18488900	4.07760400	-4.47632400

H	4.19601400	-2.84879400	-3.51078600

H	5.55452400	-4.02292600	-3.44073700

H	5.72076800	-2.39456300	-2.69928500

H	6.40805600	-3.55900500	-0.49963600

H	6.21539000	-5.13474100	-1.33728300

H	5.35914000	-4.83335900	0.20516400

H	2.60398400	-4.57264200	-2.38794800

H	3.12603200	-5.43055300	-0.90866800

H	3.97516900	-5.72779500	-2.45976500

H	4.63633900	-1.85510300	-0.69416300

H	0.25431600	-5.04164800	-2.26532700

H -0.36388600	-6.72088600	-2.16364500

H	0.07084100	-5.82491700	-0.66886800

H -3.48955500	-4.75246400	-2.97733600

H -2.35266900	-6.00267600	-3.55467200

H -1.87415800	-4.26795100	-3.57898400

H -2.32043000	-6.41055800	0.20551300

H -2.63241300	-7.23743800	-1.35925400

H -3.74935700	-5.96968900	-0.78027500

H -1.45536300	-4.15136600	0.25638700

H -2.44863200	3.22929600	-4.74492200

H -1.58247500	1.73475200	-5.20637300

H -2.90511300	2.35517100	-6.24529400

H -4.14776400	0.16576500	-6.16209400

H -2.83344300	-0.55162700	-5.16764400

H -4.52228000	-0.62034400	-4.58906200

H -4.90648000	3.11394200	-3.99396200

H -5.72487400	1.52210500	-3.83573500

H -5.35323400	2.19038100	-5.45840700

H -3.87783600	0.30976000	-2.58203600

F	6.87054700	-0.72375400	-0.86318200

F	8.30992800	0.09533600	1.30422600

F	7.93757100	2.56049000	2.36820200

F	6.10425700	4.27526000	1.32998500

F	4.76100600	-1.17654000	4.58059000

F	2.67677500	-1.35020400	6.31963300

F	0.42430800	-2.71199600	5.67030900

F	0.19732400	-3.97192200	3.26427300

F -6.17731600	-3.10922700	2.74989800

F -8.28685400	-1.58810000	1.95566700

F -8.38075000	-0.51427000	-0.53281700

F -6.36456700	-0.94534300	-2.31733800

F -5.88121100	2.88707000	1.60004300

F -4.88259500	4.98858000	3.03102900

F -2.75559200	6.38887300	2.13886000

F -1.56127300	5.78255100	-0.23135600

C	0.11179200	0.74214100	1.12750200

C	1.26653800	1.01992400	2.05796000

C	2.59222600	0.90560300	1.57594700

C	1.07475800	1.42701300	3.42127800

C	3.69678500	1.08324900	2.41579300

C	2.20128200	1.57666000	4.25554800

C	3.50381600	1.40296800	3.76682500

H	4.70946500	0.93565200	2.01814700

H	2.04046800	1.87531500	5.30268700

H	4.36581700	1.52834200	4.43843700

N	0.15963900	3.23367200	0.01064000

N -0.23654900	2.36138700	0.60821500

H	2.74601100	0.64582400	0.52389400

C -0.24672800	1.83576200	3.98929600

C -0.78334500	3.09650800	3.65041800

C -0.93803300	1.03487400	4.92259800

C -2.00185000	3.53304800	4.18933200

H -0.23202000	3.75785800	2.96573600

C -2.16636000	1.44930500	5.45374100

H -0.52427800	0.05689500	5.21004600

C -2.69075600	2.68997500	5.06858700

H -2.42051200	4.51904700	3.94672100

H -2.73052800	0.81922000	6.15578400

C -1.12903500	0.20781200	1.74010000

C -1.01028600	-1.01664300	2.43733500

C -2.41041900	0.77691000	1.60681400

C -2.12655600	-1.62667000	3.01670400

H -0.02238600	-1.48947100	2.50087900

C -3.53075500	0.17020600	2.18943200

H -2.51740900	1.72427600	1.06443500

C -3.39283300	-1.02794100	2.90987100

H -2.00679600	-2.58405100	3.54300900

H -4.51746800	0.64628100	2.08331600

H -4.26717000	-1.50610700	3.37490100

F -3.87638300	3.08741200	5.56598500

**TS-pMe-CH**

Rh 3.50362100	-0.65140100	-1.10614200

Rh 1.36254900	0.00778500	-0.06466900

O	0.78086700	-1.95960500	-0.34576600

O	0.66573000	0.42470800	-1.97603200

O	2.14742000	1.94685600	0.03486800

O	2.22292400	-0.48594900	1.76178700

O	4.14283800	1.30042600	-0.91725800

O	2.66556300	-0.20375800	-2.93804900

O	4.22283100	-1.08150800	0.77618200

O	2.80056800	-2.57051500	-1.27369100

C	1.46597400	0.22087200	-2.95694700

C	1.60959300	-2.78071500	-0.87095700

C	3.43103600	-0.91379000	1.75949700

C	3.33561300	2.14012300	-0.41176200

C -0.55820000	0.67332000	0.89746400

H	1.23205100	-3.81499000	-0.98843000

H	1.04690500	0.44772000	-3.95679800

H	3.69969500	3.18422300	-0.34089800

H	3.83901800	-1.16879500	2.75739700

C -2.41498600	1.72635100	-0.76654500

C -3.27491900	2.62254600	-1.43861300

C -1.25301500	2.26932800	-0.20013300

C -2.99221600	3.99911200	-1.47793200

H -4.19290200	2.26336400	-1.91308900

C -0.94274800	3.62891500	-0.18426900

H -0.28806700	1.53144900	-0.10512600

C -1.83116000	4.49267100	-0.83611100

H -0.02513000	3.99360900	0.28532500

H -1.61918900	5.56730200	-0.85899400

C -1.13196600	-3.33634400	2.50861900

C -0.93760900	-2.06292500	1.99417300

C -1.80305700	-1.54410600	0.98763700

C -2.87350700	-2.38468900	0.50929400

C -3.04164700	-3.69025000	1.04344600

C -2.18617300	-4.15422900	2.03269100

H -0.45599200	-3.71470300	3.28241800

H -0.10048500	-1.45628300	2.34902700

C -1.67035800	-0.20345600	0.46720500

C -3.74167500	-1.84608900	-0.48816300

H -3.86055200	-4.30324200	0.65763000

H -2.32553500	-5.15866000	2.44581300

C -3.62168100	-0.54899000	-0.95334300

C -2.60307400	0.31004800	-0.46949200

C -0.52951500	1.25177400	2.26678100

C -1.63020900	1.05407900	3.13791300

C	0.58415100	1.98041000	2.74844800

C -1.60834200	1.55053100	4.44514600

H -2.50339500	0.49931800	2.78319400

C	0.59056200	2.49349300	4.04943200

H	1.43880500	2.14150700	2.08872300

C -0.49966600	2.27601600	4.90650200

H -2.46587900	1.37645600	5.10326900

H	1.46068400	3.05739100	4.40187400

H -0.48477200	2.66893100	5.92836000

C -4.88728300	-2.49202400	-1.16729800

O -5.35112200	-3.61362800	-1.06034100

O -5.43617300	-1.56016200	-2.04547100

C -4.72006800	-0.31771500	-1.95950000

H -5.43511900	0.46764000	-1.64857500

H -4.33817000	-0.05743700	-2.96465400

C -3.92687900	4.95531700	-2.18131300

H -4.78658800	4.42975200	-2.62701900

H -4.31995000	5.71668700	-1.48400000

H -3.40654200	5.49905500	-2.99003500

**TS-oMe-CH**

Rh 3.29050800	-1.38373900	-0.67602000

Rh 1.27850100	-0.10464600	-0.03540700

O	0.29800900	-1.92952900	-0.02764800

O	0.85131400	0.05379400	-2.05973600

O	2.48158100	1.60362900	-0.16857300

O	1.87246500	-0.37137700	1.94027100

O	4.34839700	0.38371300	-0.74285200

O	2.74239900	-1.12684500	-2.64925500

O	3.73556200	-1.58571200	1.32325100

O	2.18997400	-3.11187400	-0.60366300

C	1.66965400	-0.48494000	-2.88764900

C	0.95629900	-2.99011700	-0.30656000

C	2.94041700	-1.04538300	2.15899300

C	3.71684300	1.45472500	-0.48640500

C -0.59066200	1.06378600	0.54153300

H	0.36621900	-3.92671500	-0.28473500

H	1.39727900	-0.37143700	-3.95507800

H	4.30440800	2.39242500	-0.54216500

H	3.20565300	-1.16982700	3.22731600

C -2.22793400	1.64903300	-1.53564700

C -2.94672300	2.29844700	-2.56141900

C -1.08313600	2.29858000	-1.04325700

C -2.53095300	3.56459700	-2.99087400

H -3.83345400	1.84078500	-3.00587100

C -0.64216700	3.57664000	-1.41874100

H -0.18507300	1.55503100	-0.61510400

C -1.41535200	4.19858400	-2.42202000

H -3.09614700	4.07709000	-3.77598100

H -1.12202700	5.19732000	-2.76353700

C -1.69520700	-2.12709400	3.30831200

C -1.34105600	-1.10574300	2.43846800

C -2.06992600	-0.88931200	1.23345200

C -3.17570700	-1.76855400	0.94026500

C -3.51094500	-2.81237100	1.84363600

C -2.78299500	-2.98449300	3.01216900

H -1.12160200	-2.27341600	4.22948800

H -0.48193900	-0.47289400	2.67120700

C -1.76171600	0.18617900	0.31825300

C -3.90452200	-1.53732400	-0.26491800

H -4.35311500	-3.46203100	1.59061600

H -3.04972500	-3.78708100	3.70758700

C -3.62156000	-0.48007100	-1.11036500

C -2.56415500	0.42028800	-0.82448200

C -0.57834800	2.00685300	1.69603300

C -1.79541800	2.33552000	2.34444500

C	0.62075100	2.56056000	2.20094900

C -1.81152100	3.18095800	3.45710300

H -2.73266800	1.91648000	1.96549800

C	0.59884200	3.41534600	3.31019700

H	1.56645800	2.31428800	1.71535300

C -0.61302700	3.72703900	3.94415600

H -2.76289800	3.41754800	3.94453000

H	1.53832300	3.83326700	3.68702600

H -0.62447300	4.38984800	4.81562600

C -5.04912300	-2.29654500	-0.81560600

O -5.62533000	-3.28724100	-0.40150200

O -5.43444900	-1.67563800	-2.00146100

C -4.61148700	-0.52362100	-2.24661500

H -5.27066300	0.36462200	-2.28250100

H -4.13042300	-0.63603900	-3.23646100

C	0.56557200	4.25295700	-0.82688500

H	0.35935700	4.58882800	0.20591300

H	1.42409200	3.56400000	-0.77045500

H	0.84922300	5.13470000	-1.42380000

**TS-pF-DDC-CH**

Rh 3.20448800	-0.56712300	-0.42093200

Rh 0.87457700	0.07947900	0.06909200

O	1.35509400	-0.20878000	2.06738500

O	0.37627200	-1.93012200	-0.11189500

O	0.62099700	0.32034600	-1.98849100

O	1.55815900	2.03504900	0.23071700

O	2.80462100	-0.27559200	-2.42381800

O	2.55603400	-2.52375100	-0.57495100

O	3.73298600	1.41485000	-0.23300800

O	3.52156700	-0.83988900	1.58736200

C	1.31824700	-2.75455200	-0.38785200

C	2.53947000	-0.59659300	2.36153700

C	2.80820400	2.24518500	0.05127600

C	1.63168700	0.08875100	-2.74722900

C -1.26730900	0.59512600	0.46214500

H	2.73046600	-0.73990100	3.44335400

H	1.00319300	-3.81317100	-0.47258500

H	1.44672200	0.23075100	-3.83042100

H	3.12518100	3.30155600	0.15702400

C -3.18651600	-1.20942100	-0.11887700

C -4.26811000	-1.88064000	-0.72073900

C -2.43852600	-0.32809900	-0.91659300

C -4.55567900	-1.60405200	-2.05950600

H -4.90326700	-2.57657900	-0.16621300

C -2.73991200	-0.02225000	-2.24528700

H -1.24492700	-0.15854800	-0.62438700

C -3.82696400	-0.68245200	-2.83143300

H -2.12847100	0.68022700	-2.81674200

C -1.39830400	-0.04475700	2.91382200

C -1.93761300	-0.85323600	3.92136800

C -1.81116000	-0.21974800	1.57741100

C -2.86919200	-1.86305100	3.61787100

C -3.29313200	-2.04482200	2.29903500

C -2.78293900	-1.21710200	1.28303600

C -1.65472000	2.01930300	0.28760900

C -2.50484800	2.64425900	1.23090000

C -1.17190200	2.79203500	-0.79619500

C -2.84537100	3.99608900	1.10304700

H -2.89456800	2.06345600	2.07179300

C -1.53929700	4.13331900	-0.93403300

H -0.50419400	2.32758100	-1.52658200

C -2.37127400	4.74575800	0.01747900

H -3.49671200	4.46062800	1.85080800

H -1.16033000	4.71129500	-1.78356800

H -2.64561000	5.80050800	-0.08694100

H -0.65816000	0.72034300	3.15514500

H -1.62380900	-0.69638600	4.95847600

H -3.26924800	-2.49866800	4.41385900

H -4.03047400	-2.81772600	2.05768500

H -4.11472800	-0.50474800	-3.87066600

F -5.60043200	-2.23531700	-2.63737900

**TS-oF-DDC-CH**

Rh -3.19052800	-0.17190800	0.33253600

Rh -0.76872500	0.03427500	-0.09262600

O -1.06920500	-1.33123400	-1.62984700

O -0.49017600	-1.52218800	1.25452000

O -0.71587500	1.37110900	1.50309100

O -1.23856600	1.56917000	-1.41001700

O -2.98713800	1.20168500	1.85537800

O -2.75317600	-1.68937400	1.66265700

O -3.50336200	1.34727100	-1.02159300

O -3.32163200	-1.54186300	-1.18925200

C -1.53213200	-2.01325400	1.81727300

C -2.24438500	-1.80133300	-1.81756100

C -2.47476700	1.85901200	-1.57439000

C -1.81977300	1.64425200	2.09723500

C	1.43837500	0.18728900	-0.50049500

H -2.33023000	-2.53603100	-2.64225500

H -1.33363700	-2.83315400	2.53509700

H -1.73469700	2.36582200	2.93338000

H -2.67209700	2.67311900	-2.29942500

C	3.09918000	-1.36030700	0.96817600

C	4.06366700	-1.81194600	1.89067600

C	2.45695700	-0.13510300	1.21798400

C	4.38609800	-1.01448600	2.99669000

H	4.58297600	-2.76082800	1.72699100

C	2.79497000	0.66880000	2.30855900

H	1.28512200	0.02117500	0.80002400

C	3.76852900	0.23035300	3.21214800

C	1.59640700	-1.45627700	-2.42105000

C	2.05459500	-2.67637000	-2.93061100

C	1.92051200	-1.08125800	-1.10131800

C	2.81319600	-3.55241300	-2.13275200

C	3.15590100	-3.18697800	-0.82819900

C	2.73247400	-1.94642600	-0.31808000

C	1.96097600	1.50007100	-0.96373900

C	2.96499400	1.54997400	-1.96085800

C	1.45690000	2.71986300	-0.45347900

C	3.43544000	2.77687200	-2.44193300

H	3.37037700	0.61728300	-2.36359700

C	1.94969100	3.94173200	-0.92003200

H	0.68148000	2.69524400	0.31512200

C	2.93498300	3.97837900	-1.91986900

H	4.20486400	2.79228000	-3.22098200

H	1.55231400	4.87521800	-0.50789700

H	3.30927600	4.93844200	-2.29023000

H	0.99079300	-0.78967600	-3.03875900

H	1.81262900	-2.95043900	-3.96263400

H	3.14868500	-4.51215800	-2.53791200

H	3.76805100	-3.85380900	-0.21210600

H	4.02643500	0.86209300	4.06648400

F	2.16804400	1.84290200	2.51697900

H	5.14792100	-1.35649200	3.70419300

***o*Me-IN6**

Rh	3.57100400	-0.76575900	-0.81368200

Rh	1.38804500	-0.01546600	0.12655200

O	1.29412700	-1.90755100	0.95749400

O	0.52922000	-0.79467200	-1.60632800

O	1.77670500	1.79505700	-0.83847100

O	2.45451900	0.64738800	1.78051900

O	3.78595300	1.07021400	-1.70701000

O	2.56178900	-1.47379400	-2.46239600

O	4.47135900	-0.04322500	0.89500800

O	3.31353200	-2.59047900	0.08461600

C	1.29734900	-1.34229700	-2.47724500

C	2.24124400	-2.74574900	0.75072600

C	3.72761100	0.47999300	1.78332000

C	2.86102800	1.92039800	-1.51334800

C	-0.36928700	0.60188300	0.87022200

H	2.09206600	-3.73561200	1.22323600

H	0.77435300	-1.75196200	-3.36338300

H	2.99695400	2.91056500	-1.99073900

H	4.23893700	0.84872400	2.69405400

C	-2.31241000	1.60071100	-1.14953000

C	-3.27765800	2.60576400	-0.86183300

C	-1.29951100	1.86144800	-2.09360000

C	-3.18283600	3.83708100	-1.53666200

C	-1.22289400	3.09715600	-2.74980600

H	-0.57086300	1.07562100	-2.30609900

C	-2.17053300	4.08945800	-2.47231400

H	-3.92294800	4.61497100	-1.31563800

H	-0.42695500	3.27730900	-3.48002000

H	-2.12756800	5.05738400	-2.98266000

C	-1.41511600	-3.00354200	3.05688700

C	-1.11951400	-1.82284800	2.38908200

C	-1.81977900	-1.45850200	1.20404100

C	-2.86551700	-2.33436600	0.73849100

C	-3.13408000	-3.54964300	1.42429100

C	-2.41587900	-3.87743000	2.56560100

H	-0.86760800	-3.26087300	3.96962800

H	-0.34355500	-1.15609000	2.77582300

C	-1.55923700	-0.20133600	0.52651800

C	-3.61212200	-1.89854600	-0.39332700

H	-3.92766900	-4.19497600	1.03781500

H	-2.63350800	-4.80928900	3.09775500

C	-3.40640300	-0.65395500	-0.96168500

C	-2.41448700	0.25022800	-0.50515600

C	-0.53431300	1.75211100	1.73226200

C	-1.65714000	1.80983200	2.61632500

C	0.41245300	2.81702100	1.78796900

C	-1.80622300	2.86002800	3.52202500

H	-2.38533700	0.99360400	2.60066300

C	0.23289300	3.88267700	2.67028300

H	1.25656200	2.80401600	1.09837800

C	-0.86600100	3.90474200	3.54655200

H	-2.65929700	2.87158400	4.20812500

H	0.95741300	4.70322000	2.68302900

H	-0.99040400	4.73616800	4.24818300

C	-4.69994200	-2.58344400	-1.12757900

O	-5.20796600	-3.67838100	-0.95810000

O	-5.11999300	-1.73273600	-2.14568000

C	-4.36330700	-0.50767400	-2.11679500

H	-5.06686100	0.33762200	-2.00529700

H	-3.84266800	-0.38688700	-3.08487900

C	-4.35832100	2.40893900	0.17819900

H	-5.11697600	3.20593100	0.11147200

H	-4.87389800	1.43835600	0.08312900

H	-3.93172900	2.44032700	1.19754100

***o*Me-TS6**

Rh	3.41016900	-1.09191400	-0.81158300

Rh	1.31667500	-0.00732500	-0.07562100

O	0.70244700	-1.90058000	0.49654300

O	0.56091100	-0.38458700	-1.97477200

O	2.13101500	1.78187100	-0.79349700

O	2.22822800	0.25555000	1.76916000

O	4.07565100	0.74920100	-1.47037800

O	2.51575800	-1.41206000	-2.64207900

O	4.19404500	-0.72287800	1.05733400

O	2.68320100	-2.88906000	-0.14551700

C	1.32442000	-0.99556100	-2.80545500

C	1.50440100	-2.88490700	0.33996400

C	3.43377700	-0.15705300	1.90857500

C	3.30036400	1.74351600	-1.32346400

C	-0.54768100	1.07536300	0.54346600

H	1.11285600	-3.86511800	0.67445400

H	0.87647200	-1.18348600	-3.80084100

H	3.67708200	2.71558400	-1.69896600

H	3.87111400	0.00778600	2.91296400

C	-2.45317300	1.56504000	-1.29091600

C	-3.38710100	2.36736900	-2.01212100

C	-1.19876000	2.15123900	-1.02856900

C	-2.96585300	3.64048200	-2.44562300

C	-0.78676000	3.42617200	-1.41854900

H	-0.28603400	1.41703800	-0.77555900

C	-1.69367100	4.17151400	-2.17645100

H	-3.69067500	4.26087300	-2.98477700

H	0.20632300	3.79822600	-1.15489200

H	-1.42901000	5.17178000	-2.53248700

C	-1.23100200	-1.96456300	3.59301400

C	-1.03273400	-0.96245300	2.65416500

C	-1.82585600	-0.90852600	1.47148200

C	-2.81787500	-1.93099400	1.26735800

C	-2.98717100	-2.95829200	2.23334900

C	-2.20873800	-2.96738300	3.38251300

H	-0.61701300	-1.98420100	4.49935000

H	-0.25484200	-0.21319200	2.82060000

C	-1.69305500	0.14231300	0.49117700

C	-3.57339200	-1.87392700	0.05833700

H	-3.73915300	-3.72834500	2.04279600

H	-2.34984700	-3.75593600	4.12905900

C	-3.50389900	-0.80848500	-0.82673400

C	-2.61922100	0.28363800	-0.58045700

C	-0.46208100	2.14645600	1.57102400

C	-1.57671500	2.38982100	2.41334800

C	0.70683700	2.91971300	1.76630700

C	-1.51555900	3.35319800	3.42472200

H	-2.49240100	1.80816500	2.27018000

C	0.75450300	3.89673400	2.76628300

H	1.57214700	2.74699900	1.12433500

C	-0.35023800	4.11417900	3.60429600

H	-2.38492700	3.51539600	4.07027000

H	1.66805700	4.48550800	2.90062000

H	-0.30307200	4.87160600	4.39350900

C	-4.47459600	-2.90987800	-0.49220600

O	-4.83990000	-3.97718300	-0.03009800

O	-4.89402200	-2.47815800	-1.74308500

C	-4.35445800	-1.17589500	-2.02308700

H	-5.20835900	-0.50631900	-2.20432000

H	-3.75587700	-1.23104500	-2.95209100

C	-4.82954200	1.98806600	-2.24454900

H	-4.95464100	1.32130600	-3.11664000

H	-5.26440300	1.48549700	-1.36469800

H	-5.43148900	2.88932400	-2.44475100

***c*-INT1**

Rh -0.96499900	-0.24044100	-0.79120800

Rh -1.86149200	-0.17903500	-3.08012700

N -0.25427400	4.26145000	-1.20014100

O	0.94833800	3.14899400	-2.86910000

O -0.80108900	5.23931000	0.85420100

C	0.91079000	3.84375100	-1.86567200

C	2.04740300	4.39730100	-1.07401100

C	3.41091500	4.41163700	-1.35212700

C	4.26703000	5.06176200	-0.43337200

C	3.74940300	5.65857900	0.73012300

C	2.36208300	5.62433900	1.00911900

C	1.52210000	5.01007900	0.08358600

C	0.03051200	4.88788200	0.03916600

N -5.27840800	0.58556600	0.57549500

O -4.43401900	2.27003600	-0.80339400

O -5.81561900	-0.66470600	2.47890600

C -4.77777000	1.87177600	0.29587600

C -4.70909900	2.58169900	1.60525200

C -4.25648000	3.86212400	1.91340800

C -4.19087600	4.23545700	3.27640200

C -4.59234300	3.33438200	4.28213000

C -5.07492700	2.04677900	3.95298800

C -5.12324900	1.68489700	2.61006800

C -5.47117800	0.38186800	1.96092800

N -1.55659400	-4.69916700	-0.75386000

O -3.58123600	-3.52587800	-0.85839600

O	0.41498900	-5.63393200	0.08844400

C -2.73028200	-4.12219600	-0.22055600

C -2.65794700	-4.33413000	1.25388200

C -3.51000300	-3.89904600	2.26528300

C -3.12476700	-4.12306500	3.60697100

C -1.92158400	-4.79171100	3.90356600

C -1.07412100	-5.24357000	2.86479000

C -1.44707700	-4.99162300	1.54737600

C -0.70757100	-5.19526300	0.26214300

N	3.28104700	-0.60353600	-2.59836900

O	2.01239500	-2.57074200	-2.62837200

O	4.82650200	1.03157300	-1.98064100

C	2.98901600	-1.93953800	-2.25901400

C	4.10733700	-2.37548600	-1.36603300

C	4.36447100	-3.60450100	-0.76868100

C	5.51181300	-3.72719500	0.04993900

C	6.38080500	-2.63640900	0.23356700

C	6.10294900	-1.38691400	-0.36634100

C	4.96981600	-1.27779300	-1.16745900

C	4.41954600	-0.11788500	-1.92666200

O -1.27717700	1.77593300	-0.71471400

O -2.02463900	1.85492800	-2.86571000

O -2.91730300	-0.52765200	-0.20438300

O -3.74809600	-0.43259600	-2.31729300

O -0.71419400	-2.24182400	-1.08657200

O -1.61303600	-2.22187300	-3.17591800

O	0.86510000	0.13057600	-1.64490700

O	0.04374500	0.04832500	-3.76300800

C -1.65910300	2.38819600	-1.77676800

C -1.61798600	3.91744700	-1.60792200

C -2.20343900	4.82931500	-2.73458900

C -2.08494500	6.28877000	-2.24469100

C -1.47370500	4.68292600	-4.08338700

C -3.69866200	4.49149600	-2.90767800

C -3.87069000	-0.49211800	-1.06087700

C -5.26043700	-0.52122400	-0.38939100

C -6.52517100	-0.63155700	-1.29404200

C -6.69111600	0.56529900	-2.24872800

C -7.75202900	-0.71346300	-0.36120600

C -6.42078600	-1.94734100	-2.09472000

C -1.13637900	-2.80320200	-2.15851200

C -1.01713500	-4.33805600	-2.07446400

C -1.50935700	-5.20413000	-3.26928600

C -0.65538800	-4.83226400	-4.50108800

C -1.24395100	-6.67936500	-2.90366700

C -3.00570100	-5.01214400	-3.57785000

C	0.98489200	0.12444300	-2.91761700

C	2.43541800	0.27437300	-3.40517400

C	2.70647000	0.19341900	-4.94461300

C	1.99353500	1.38072800	-5.62802500

C	4.22637400	0.35477300	-5.16529800

C	2.24548400	-1.14253800	-5.55835500

H	0.07640800	-4.51989600	-1.97273100

H -0.17687700	-6.85445700	-2.65660900

H -1.51359800	-7.33528000	-3.75570600

H -1.85020800	-6.99570800	-2.03062000

H -3.28711600	-5.63799400	-4.44953800

H -3.64148300	-5.31639600	-2.72367400

H -3.23971300	-3.95742000	-3.81349600

H -0.90382600	-5.50569400	-5.34613700

H -0.83831700	-3.79036700	-4.82401600

H	0.42845400	-4.93866900	-4.28747100

H -5.57841400	-1.92445500	-2.81089500

H -7.35722700	-2.11165400	-2.66530900

H -6.26895600	-2.81756800	-1.42416100

H -7.66470500	-1.55050300	0.36093300

H -8.67127800	-0.86715000	-0.96150200

H -7.88589900	0.22184200	0.21976600

H -5.81899000	0.67425700	-2.91881700

H -6.81631300	1.51682100	-1.69522200

H -7.59835900	0.41818500	-2.86998600

H -5.24223700	-1.43319200	0.24482200

H -3.84710800	3.45525700	-3.26304800

H -4.14898700	5.18369200	-3.64784500

H -4.24824500	4.59781700	-1.95206000

H -0.41592600	5.00416600	-4.02191600

H -1.97218600	5.32363400	-4.83972700

H -1.49054700	3.63745400	-4.44199900

H -2.57897200	6.43158900	-1.26204800

H -2.56437300	6.97093100	-2.97530600

H -1.02620500	6.60318000	-2.14238300

H -2.22344700	4.10869100	-0.69720400

H	2.78499900	-2.00492700	-5.11890000

H	1.16230400	-1.30735900	-5.40935500

H	2.45144200	-1.14013000	-6.64845500

H	2.25819400	1.40164000	-6.70472500

H	0.89483100	1.30472400	-5.54041000

H	2.30220000	2.34501800	-5.17599900

H	4.79764100	-0.49838300	-4.74540400

H	4.61088300	1.28493500	-4.70109700

H	4.44250900	0.39282500	-6.25194900

H	2.72247800	1.30079500	-3.08606400

F -4.64132200	-3.24180200	2.02526600

F -3.88943000	-3.67898500	4.60123000

F -1.57954300	-4.98767600	5.17340600

F	0.06909700	-5.84960300	3.19166500

F -5.41532100	1.21944100	4.94056300

F -4.49681000	3.69760700	5.55983200

F -3.74907500	5.44035900	3.61939100

F -3.87286100	4.73725400	0.98591600

F	1.92221400	6.19128000	2.12908400

F	4.58164900	6.25677400	1.58046800

F	5.57690500	5.10959100	-0.66494400

F	3.93530500	3.85128400	-2.43832000

F	6.93325100	-0.37032700	-0.13653600

F	7.45687900	-2.78089600	1.00165200

F	5.77481400	-4.87839600	0.66250300

F	3.55426700	-4.65628900	-0.90310100

C	1.04754500	-1.13514000	1.11119700

O	2.11770500	-0.82485900	0.61928000

O	0.81716500	-2.30106300	1.77760600

C	1.89696400	-3.27962900	1.76773000

H	2.08807700	-3.58122700	0.71605300

H	1.47396000	-4.16057900	2.28532700

C -0.24209000	-0.38390000	1.01961200

C -0.90876300	0.01476900	2.25093200

C -2.15061000	-0.57742900	2.59472300

C -0.29498900	0.92297900	3.18211400

C -2.75098800	-0.32776600	3.83292500

C -0.96165100	1.21628200	4.39860500

C -2.16639000	0.58780100	4.72873700

H -3.69478800	-0.83477000	4.08391100

H -0.49650800	1.93563200	5.08830400

H -2.66276100	0.81800100	5.68379100

H -2.61960000	-1.26228100	1.87983100

C	3.12018600	-2.83024600	2.41152200

C	4.18999300	-2.46392900	2.88928700

C	5.46216900	-1.99407500	3.33434200

C	5.86380600	-0.66771700	3.02955200

C	6.36388300	-2.84781600	4.01754900

C	7.13886300	-0.21769200	3.38951500

H	5.16935600	-0.00238000	2.49731800

C	7.63627500	-2.38552700	4.37444800

H	6.05716800	-3.87891400	4.24625200

C	8.02909800	-1.07312100	4.06034500

H	7.43549600	0.81102200	3.13427700

H	8.33254000	-3.05852900	4.89784000

H	9.03340000	-0.71771900	4.33736000

C	0.96026500	1.51139500	2.88274900

C	2.07645500	1.96507200	2.62288300

C	3.39459700	2.40685400	2.32683400

C	4.11059200	3.23014600	3.23623500

C	4.02889000	1.98731300	1.12674600

C	5.42976100	3.60487300	2.95672500

H	3.61765200	3.56191000	4.16153600

C	5.34899100	2.37110100	0.85773200

H	3.46968200	1.34518300	0.43102800

C	6.05276800	3.17607900	1.77128800

H	5.97510900	4.24497300	3.66647200

H	5.81482700	2.04428000	-0.08259300

H	7.08563500	3.48375500	1.54877500

***c*-INT2**

Rh -0.79958100	-0.09086000	-0.87146900

Rh -1.56840100	-0.23341500	-3.20982300

N	3.46463800	-1.15013700	-2.64634900

O	1.88640300	-2.86661700	-2.79844000

O	5.29834400	0.14145700	-1.97269000

C	2.95534600	-2.43008300	-2.39348500

C	3.94313700	-3.09065600	-1.48842300

C	3.90045800	-4.34181000	-0.87563800

C	4.92394000	-4.66257500	0.04750000

C	5.94736400	-3.73687200	0.33144200

C	5.97906100	-2.47179200	-0.30002500

C	4.97318900	-2.16925400	-1.21178500

C	4.67802600	-0.90000900	-1.94793200

N	0.13880500	4.37630800	-1.76515100

O	1.43424400	2.82156500	-2.92932100

O -0.58138500	5.94128200	-0.18599300

C	1.33086400	3.69466500	-2.08166900

C	2.36222000	4.22196300	-1.14745800

C	3.68540000	3.82954400	-0.95968700

C	4.40948500	4.41428800	0.10770600

C	3.79875000	5.37287400	0.93877200

C	2.46340400	5.78616600	0.71893700

C	1.75423900	5.19130900	-0.32027200

C	0.31105400	5.28457200	-0.69225400

N -5.07771600	0.92270100	0.34544600

O -4.04700900	2.65407500	-0.84831000

O -5.73959100	-0.48023300	2.09150700

C -4.41067000	2.15936100	0.20480500

C -4.20503100	2.66779100	1.59256500

C -3.56112700	3.81722900	2.04136400

C -3.40524600	3.99223500	3.43655400

C -3.90193500	3.03199400	4.33904600

C -4.57239200	1.87753700	3.87032600

C -4.71478200	1.71223900	2.49515400

C -5.26491400	0.57164600	1.70042700

N -1.56553500	-4.61106100	-0.51545900

O -3.54289700	-3.37692300	-0.74316800

O	0.31504600	-5.60891200	0.45464600

C -2.78074300	-4.05549300	-0.07292500

C -2.88273700	-4.41078300	1.37338200

C -3.87360700	-4.11269600	2.30272000

C -3.66133100	-4.49333400	3.64752700

C -2.48790300	-5.17251000	4.02488300

C -1.48861400	-5.47712000	3.06861300

C -1.70467400	-5.08860700	1.74838800

C -0.82004400	-5.18152500	0.54423500

O	1.13130300	-0.03628900	-1.66673300

O	0.37256900	-0.13959900	-3.80685700

O -0.90323900	1.94874500	-1.09094700

O -1.65820100	1.80452700	-3.22714800

O -2.78816300	-0.09642600	-0.38487100

O -3.50597300	-0.31500300	-2.53050900

O -0.73067900	-2.13391000	-0.92486900

O -1.42285700	-2.27333400	-3.08776400

C	1.29139000	-0.10188900	-2.93734300

C	2.76378800	-0.10808100	-3.39825300

C	3.04715400	-0.13451100	-4.93822600

C	4.57875300	-0.09890900	-5.13512800

C	2.48621200	-1.39488500	-5.62450200

C	2.45310100	1.14124900	-5.57318900

C -1.25964400	2.45432000	-2.21645300

C -1.18502900	3.99353100	-2.26040700

C -1.62344700	4.70598700	-3.58049600

C -0.76200000	4.29259700	-4.78873600

C -1.48292600	6.22697600	-3.35852600

C -3.11206800	4.38493500	-3.83504200

C -3.69442800	-0.17291500	-1.29067000

C -5.12109700	-0.09615000	-0.70976300

C -6.32715400	0.00249600	-1.69321800

C -6.38330700	-1.30664400	-2.51044000

C -7.60807500	0.10697900	-0.83876500

C -6.23685400	1.21822500	-2.63405800

C -1.06041300	-2.77632500	-1.98699000

C -0.94788400	-4.29957100	-1.80662500

C -1.37193600	-5.23309400	-2.98206800

C -0.44090800	-4.95361600	-4.18170000

C -1.14660600	-6.68721700	-2.51532800

C -2.84532900	-5.04791600	-3.38961700

H -5.24495400	-1.05252400	-0.15190400

H -7.69014200	-0.73367100	-0.11994100

H -8.50074500	0.08833000	-1.49560800

H -7.63696300	1.05276300	-0.26071300

H -7.11320900	1.22485000	-3.31404100

H -6.23552700	2.17215600	-2.07200400

H -5.31890500	1.18888000	-3.25016700

H -7.29112700	-1.31070000	-3.14702400

H -5.49940800	-1.41722500	-3.16521000

H -6.42886700	-2.19439900	-1.84597400

H -3.27109200	3.31375800	-4.05671500

H -3.47755600	4.97600200	-4.69908500

H -3.73510700	4.64419200	-2.95455900

H -2.05568800	6.56887300	-2.47349800

H -1.85874000	6.76969500	-4.24917000

H -0.42461100	6.52299400	-3.21088400

H -0.81159000	3.20419300	-4.97413800

H	0.30187300	4.56377600	-4.64002800

H -1.11884100	4.82155600	-5.69616200

H -1.88485300	4.32926400	-1.46290900

H	1.35098700	1.16592300	-5.49906200

H	2.73234600	1.18335200	-6.64551300

H	2.84379500	2.05245700	-5.07775900

H	2.94782600	-2.31879800	-5.22225500

H	2.71453100	-1.35640900	-6.70945900

H	1.39127700	-1.47815500	-5.49993900

H	5.04092800	0.76445000	-4.61665100

H	4.81236700	-0.02357600	-6.21622100

H	5.06548000	-1.02112400	-4.75720700

H	3.17940100	0.84579200	-3.00734000

H -3.53494400	-5.28863200	-2.55674200

H -3.05061200	-4.00981600	-3.71083000

H -3.08493600	-5.72901000	-4.23177400

H -0.65394000	-5.67889500	-4.99293900

H -0.58250000	-3.93269100	-4.58137300

H	0.62513500	-5.05705800	-3.89500100

H -1.81614900	-6.95572900	-1.67289600

H -0.10065000	-6.85329500	-2.18612600

H -1.36199300	-7.38594600	-3.34846900

H	0.13867600	-4.47173200	-1.63955200

F -3.05782000	4.73298500	1.21619100

F -2.77631000	5.06596000	3.90895900

F -3.72865600	3.21440200	5.64556600

F -5.01048400	0.98668800	4.75812000

F	1.93084200	6.69483200	1.53293200

F	4.49109600	5.89444000	1.94908100

F	5.66696600	4.06749300	0.33199400

F	4.27940400	2.92025100	-1.72311000

F	6.94331500	-1.61460100	0.02947700

F	6.88963800	-4.06145000	1.21704500

F	4.92633900	-5.84446000	0.65890400

F	2.93734000	-5.23009400	-1.10953400

F -0.38865800	-6.10537500	3.47095000

F -2.31284800	-5.51156300	5.29963000

F -4.56334700	-4.17927200	4.57520100

F -4.97496700	-3.43218500	1.99399900

C	3.40104800	0.78439300	0.99709400

O	4.38050800	1.48914800	0.89214500

O	3.44661900	-0.56984300	0.69162500

C	2.14431600	-1.12728400	0.77532000

H	1.87217600	-1.62877200	-0.17217100

H	2.10534800	-1.87763500	1.59803200

C	1.22621800	0.02893300	1.10777400

C -0.20453900	-0.14133400	1.04671600

C	2.00919000	1.12110700	1.39245200

C -0.93907300	-0.41091100	2.26491600

C -0.33849500	-0.07910100	3.52416300

C -2.24586000	-0.98461700	2.28846600

C -1.03463900	-0.24552600	4.72225300

H	0.67771700	0.33898800	3.54323100

C -2.92469500	-1.16928300	3.49625500

H -2.71220400	-1.28186600	1.34294900

C -2.33302200	-0.78796500	4.71425500

H -0.55897300	0.04424300	5.67136700

H -3.94302200	-1.58316200	3.48556900

H -2.88760100	-0.91616800	5.65626500

C	1.61565500	2.36168200	2.08003300

C	0.45735800	3.08096600	1.72437600

C	2.35454200	2.76582300	3.24527700

C	0.04595800	4.19949000	2.46427800

H -0.11538300	2.76158100	0.83910500

C	1.93157500	3.91122900	3.96610200

C	0.78995000	4.62007500	3.57922700

H -0.83036000	4.77482800	2.13938900

H	2.51028600	4.21854200	4.84931800

H	0.47428600	5.50837500	4.14622100

C	3.43527200	1.96568300	3.71513400

C	4.32269800	1.17760600	4.04943500

C	5.34482300	0.21681900	4.30912100

C	5.67513400	-0.17415300	5.63215100

C	6.02870500	-0.39009400	3.21961100

C	6.64960500	-1.15446400	5.85662100

H	5.15100500	0.29697500	6.47682400

C	6.99533000	-1.37417300	3.45857300

H	5.79720200	-0.06887700	2.19335400

C	7.30767000	-1.76162000	4.77336500

H	6.89597800	-1.45102700	6.88782800

H	7.50749500	-1.84306300	2.60559300

H	8.06870900	-2.53617000	4.95429600

***c*-INT3**

Rh 0.12402300	0.20866400	0.73454500

Rh 0.27201200	0.19723400	3.20045900

N -2.44154100	-3.65656000	1.22258300

O -0.30461600	-3.92644500	2.12433600

O -4.18995100	-3.61100000	-0.32277400

C -1.16276300	-4.23357000	1.31437100

C -1.06547200	-5.19749500	0.18238300

C -0.00427800	-6.00165000	-0.21675500

C -0.10615000	-6.66902700	-1.45748000

C -1.24470500	-6.50388000	-2.26706600

C -2.32349700	-5.69280100	-1.84388900

C -2.21859000	-5.04783700	-0.61480600

C -3.11562200	-4.05090000	0.05061500

N -3.73885100	2.85136000	1.50130500

O -3.72409400	0.65809900	2.29463100

O -4.20959000	4.75951000	0.23067900

C -4.20803700	1.52923300	1.59001800

C -5.35461900	1.43642300	0.64649700

C -6.15085200	0.34783900	0.30108700

C -7.15363300	0.53704500	-0.67747200

C -7.33811200	1.79878900	-1.27595100

C -6.51506000	2.89359200	-0.92215800

C -5.52864200	2.69716300	0.04016200

C -4.46155000	3.61308900	0.55067600

N	3.20495100	3.59055400	0.48993500

O	1.12282200	4.54286600	1.00194400

O	4.95714500	2.64132700	-0.73393600

C	2.02066100	4.30962300	0.21276500

C	2.09091400	4.64721900	-1.24238000

C	1.21402600	5.37024200	-2.04491800

C	1.47088000	5.42865600	-3.43445800

C	2.61174300	4.80615000	-3.97735400

C	3.52235300	4.11417700	-3.14486900

C	3.24208200	4.03979500	-1.78306600

C	3.95483100	3.33401400	-0.67685700

N	4.08634000	-2.30276500	1.00813400

O	4.37382800	-0.28594500	2.15666200

O	4.10301800	-3.89888600	-0.70089700

C	4.68593600	-1.05637600	1.26328200

C	5.70185400	-0.86618500	0.18934800

C	6.62411700	0.15968000	0.00261000

C	7.42614800	0.12171600	-1.16274800

C	7.30712600	-0.94362100	-2.07744200

C	6.38642400	-1.99847500	-1.85709100

C	5.59375500	-1.94534900	-0.71320800

C	4.53176600	-2.87248400	-0.20573400

O -1.39339500	-1.16744200	0.93180200

O -1.32781600	-1.08322100	3.20972500

O -1.24192400	1.72926400	0.97926100

O -0.96272000	1.80790900	3.23157900

O	1.65292900	1.56041200	0.77352600

O	1.86823600	1.48927800	3.03791900

O	1.52787800	-1.31224500	0.82309000

O	1.53211400	-1.40945300	3.09708100

C -1.77771200	-1.51208200	2.10849400

C -2.90936800	-2.56161700	2.07777500

C -3.54343800	-3.04442800	3.42257200

C -4.66929100	-4.03915900	3.06456200

C -2.52711900	-3.73712400	4.34979200

C -4.17572200	-1.83352600	4.13924900

C -1.49426100	2.18913700	2.14681000

C -2.52932300	3.33628000	2.16748600

C -2.78513400	4.03096600	3.54739600

C -3.35892000	3.05512800	4.59249100

C -3.79579600	5.17850600	3.33491200

C -1.45434100	4.64547800	4.03416100

C	2.20153000	1.89095200	1.88698100

C	3.42365500	2.81066700	1.70862300

C	3.95733100	3.61371700	2.93979200

C	4.54555600	2.60802400	3.95397400

C	5.10279900	4.51654300	2.43442800

C	2.87276200	4.47206300	3.61566200

C	1.92742400	-1.77439600	1.95575600

C	3.00140500	-2.87307900	1.81199300

C	3.50090900	-3.62569200	3.09137500

C	2.30325900	-4.33768700	3.75192100

C	4.50631900	-4.70067700	2.62356400

C	4.18719800	-2.69808700	4.11182600

H	4.23970900	2.10752400	1.43037200

H	5.88377900	3.92377700	1.91461500

H	5.57912800	5.03412500	3.29120800

H	4.73825600	5.29473000	1.73377200

H	3.30105500	4.97674600	4.50585500

H	2.47792700	5.25150100	2.93690000

H	2.01846100	3.85244900	3.94967600

H	5.00979200	3.16143600	4.79525500

H	3.76630900	1.93912100	4.36166500

H	5.32353600	1.97398800	3.48297500

H -0.70919300	3.87039800	4.28954900

H -1.63620600	5.26016700	4.93877000

H -1.00907500	5.30539500	3.26053500

H -3.46389600	5.88535500	2.54935900

H -3.91926700	5.73996100	4.28294200

H -4.79642400	4.79950100	3.04519600

H -2.66865100	2.21671700	4.79472400

H -4.32665100	2.63047600	4.25665000

H -3.54687400	3.59631200	5.54224400

H -2.11997000	4.10816100	1.47835900

H -3.41549600	-1.08686300	4.43234200

H -4.69989600	-2.18131100	5.05258600

H -4.91152400	-1.32403200	3.48716600

H -2.11491700	-4.66055700	3.89714800

H -3.03111900	-4.02861200	5.29404900

H -1.68112100	-3.06911100	4.59451200

H -5.40979000	-3.58650800	2.37428200

H -5.20112500	-4.35072100	3.98587600

H -4.27264800	-4.95737000	2.58500800

H -3.71936000	-2.07977200	1.48929700

H	5.11173500	-2.24309600	3.70576600

H	3.51736800	-1.88041400	4.43460000

H	4.47628900	-3.28980300	5.00442800

H	2.66431700	-4.92951300	4.61744500

H	1.54472900	-3.61876100	4.11054000

H	1.80212300	-5.02663700	3.04501800

H	5.41900500	-4.25074700	2.18200900

H	4.05815000	-5.37720700	1.86761900

H	4.82704500	-5.31461200	3.48899700

H	2.53788400	-3.63797100	1.15301500

F	0.14117100	5.99552200	-1.56159800

F	0.63792200	6.08233600	-4.23864800

F	2.83041100	4.87438200	-5.28849400

F	4.59852500	3.55703700	-3.69667900

F -6.68843400	4.05282100	-1.55409700

F -8.27479200	1.95091900	-2.20094300

F -7.91917400	-0.48099500	-1.04959400

F -5.99440600	-0.86304900	0.84239900

F -3.36646600	-5.54043000	-2.65903400

F -1.28505700	-7.09270000	-3.45879200

F	0.91051300	-7.39944300	-1.90793000

F	1.12523000	-6.11405500	0.48532700

F	6.31832900	-2.98389400	-2.74606200

F	8.08073800	-0.95781000	-3.16046100

F	8.31436500	1.08197200	-1.39419400

F	6.78579600	1.15255700	0.86988000

C	0.04228600	0.21598400	-1.26128000

C -0.84312400	-2.24933400	-2.65787200

C -0.81537800	-2.81538700	-3.95703600

C	0.10278100	-2.70882000	-1.71541100

C	0.12741100	-3.79334600	-4.30353200

H -1.51037300	-2.44269200	-4.72425500

C	1.06613600	-3.66560900	-2.07177100

H	0.08905400	-2.31324000	-0.69215200

C	1.08276200	-4.21394500	-3.36361600

H	0.13122400	-4.20887800	-5.32289000

H	1.81847100	-3.98744700	-1.33624100

H	1.83681500	-4.96972900	-3.63120900

C -2.21132100	3.78540200	-2.03361100

C -1.51824100	2.62206000	-1.72663600

C -2.07222700	1.33948000	-2.02139500

C -3.38202800	1.29056700	-2.62048800

C -4.07282400	2.49722200	-2.92337200

C -3.49035600	3.72538300	-2.64207900

H -1.77105900	4.76128700	-1.79151600

H -0.52779500	2.68504200	-1.25427200

C -1.29564000	0.12293400	-1.87202300

C -3.90017000	-0.00060100	-2.92899500

H -5.06199400	2.42565000	-3.39672000

H -4.03190900	4.65559300	-2.86677900

C -3.11751600	-1.14329900	-2.85417100

C -1.76524200	-1.10711700	-2.40451800

C	1.17918900	0.37022900	-2.14768000

C	0.96373200	0.69969400	-3.52376600

C	2.52859000	0.24037600	-1.70323800

C	2.03421300	0.89655000	-4.39728000

H -0.06709200	0.81083800	-3.88961000

C	3.59273000	0.42837200	-2.58710400

H	2.71515700	-0.02102000	-0.65620100

C	3.35516300	0.75698200	-3.93339900

H	1.84235700	1.16080300	-5.44844200

H	4.62378100	0.34762800	-2.21906000

H	4.20139500	0.91746600	-4.61792900

C -5.24507000	-0.37424900	-3.42616200

O -6.22868600	0.31342500	-3.63032900

O -5.24906300	-1.73267200	-3.63029600

C -3.98312600	-2.30668800	-3.27233100

H -3.59694400	-2.88223100	-4.13804800

H -4.14620800	-3.01690500	-2.43326600

***c*-TS-INT3-CH**

Rh -0.21161400	-0.26268800	0.73805000

Rh -0.30044500	-0.34230800	3.17999800

N	1.62981200	3.98269300	1.24499600

O -0.53907700	3.83274700	2.10797000

O	3.39743500	4.35251700	-0.23750000

C	0.26105800	4.30080300	1.31618100

C	0.01189600	5.27428400	0.21234300

C -1.15910300	5.92477500	-0.16587000

C -1.10704500	6.77452300	-1.29577500

C	0.09463700	6.94938000	-2.00617900

C	1.27531400	6.27987500	-1.61144800

C	1.21619000	5.44664900	-0.49916200

C	2.25447000	4.56897700	0.12886200

N	4.08046500	-2.17587100	1.34319100

O	3.70578300	-0.04614700	2.22533500

O	4.86060700	-3.92508700	-0.00168900

C	4.31932200	-0.79699100	1.48473600

C	5.42793700	-0.47516400	0.54405000

C	6.02985800	0.74356100	0.24243500

C	7.04836100	0.76109800	-0.73816700

C	7.44179700	-0.42872100	-1.38123800

C	6.81381700	-1.65774800	-1.07236700

C	5.81055800	-1.66486500	-0.10762200

C	4.91527500	-2.76603400	0.36577600

N -2.41338100	-4.35013000	0.69282000

O -0.21690900	-4.56210900	1.47901000

O -4.26595400	-4.27327100	-0.72986200

C -1.05892200	-4.73087600	0.61399500

C -0.88595800	-5.31555800	-0.74882900

C	0.24746100	-5.83674700	-1.36331500

C	0.15476300	-6.21933600	-2.72106900

C -1.06764300	-6.11156200	-3.41228400

C -2.22432100	-5.61736700	-2.76365300

C -2.11324800	-5.21764100	-1.43448800

C -3.10665300	-4.57813400	-0.51583900

N -4.42454100	1.87733100	0.97043600

O -4.52412700	-0.37828300	1.59024000

O -4.58075600	3.85248000	-0.27623700

C -4.83162400	0.53527000	0.84290100

C -5.64377800	0.48010200	-0.41051300

C -6.26167100	-0.59728200	-1.03751500

C -6.87408000	-0.37552600	-2.29377300

C -6.87365000	0.90585600	-2.87808200

C -6.25237300	1.99686400	-2.22388000

C -5.65119800	1.76565200	-0.98951700

C -4.85757600	2.67814100	-0.10813000

O	1.00887700	1.37681500	0.92431900

O	1.06976200	1.18392400	3.19419000

O	1.42631200	-1.50123300	0.88266000

O	1.18524500	-1.72591900	3.13312900

O -1.44865000	-1.90131100	0.76585400

O -1.68983300	-1.83600500	3.02923600

O -1.87544600	0.97841700	0.85675400

O -1.80650800	1.03571600	3.12832300

C	1.37705200	1.73803500	2.09889000

C	2.29053900	2.98035800	2.08736400

C	2.81352400	3.55184400	3.44610500

C	3.70249300	4.77334900	3.12673100

C	1.67402700	3.98560400	4.38780800

C	3.69082000	2.48105400	4.12734600

C	1.76352400	-1.95969800	2.02914500

C	2.99351300	-2.89479400	2.01084800

C	3.41134600	-3.55650100	3.36818000

C	3.80720000	-2.51153300	4.42942700

C	4.62309600	-4.47722200	3.10799600

C	2.24287900	-4.43340900	3.86797500

C -1.93519600	-2.30716100	1.87914600

C -2.94611700	-3.46055000	1.72615300

C -3.46614900	-4.17829400	3.01179900

C -4.27973100	-3.16035100	3.84132200

C -4.41659600	-5.30701900	2.55852800

C -2.32846900	-4.77588800	3.86018700

C -2.29043500	1.36723700	2.00953500

C -3.46878100	2.36212900	1.96727800

C -4.12638200	2.79699800	3.31994700

C -3.08396500	3.57846000	4.14817300

C -5.28909100	3.75744600	2.98795500

C -4.67048300	1.60242900	4.12639700

H -3.83268800	-2.98694700	1.24686100

H -5.23163400	-4.92049600	1.91324000

H -4.87550000	-5.78971000	3.44463800

H -3.87786000	-6.09364400	1.99208300

H -2.75419800	-5.26405000	4.76089000

H -1.75687600	-5.54100200	3.29950200

H -1.61567600	-3.99665100	4.18859800

H -4.75489900	-3.67584200	4.70061800

H -3.64162700	-2.34692900	4.23231500

H -5.08295200	-2.69787200	3.23191000

H	1.35366200	-3.83131800	4.12733200

H	2.56071000	-4.99364700	4.77065300

H	1.93701600	-5.16934300	3.09699800

H	4.41476300	-5.22155700	2.31451100

H	4.87860800	-5.02065500	4.03999200

H	5.52279100	-3.90424800	2.80538700

H	2.97509200	-1.82244700	4.65948100

H	4.67385600	-1.90438800	4.09773200

H	4.10585900	-3.02853000	5.36444700

H	2.72434500	-3.71664900	1.31098400

H	3.11085600	1.57582900	4.38187900

H	4.12185900	2.89932000	5.05969700

H	4.52504600	2.16952100	3.46834700

H	1.07143400	4.80853300	3.95519200

H	2.10458000	4.35550200	5.34080200

H	0.99224800	3.14540100	4.61386700

H	4.51578300	4.51370800	2.41919900

H	4.16444900	5.15340700	4.05993700

H	3.11790600	5.60685700	2.68591600

H	3.18174600	2.66924900	1.50074700

H -5.46329300	1.05926100	3.57521600

H -3.87208700	0.87859900	4.37317100

H -5.11506100	1.96920100	5.07417100

H -3.55973800	3.95702600	5.07557200

H -2.22300000	2.94602900	4.42905600

H -2.69417900	4.44623800	3.57954500

H -6.10701800	3.24552500	2.44080800

H -4.94842300	4.61369200	2.37172300

H -5.72139900	4.15644600	3.92744500

H -3.03793700	3.28104800	1.51231100

F	1.42098500	-5.95017700	-0.73956800

F	1.23068400	-6.67642200	-3.35697000

F -1.12791100	-6.47453400	-4.69158000

F -3.36016100	-5.54034000	-3.45382800

F	7.17639300	-2.74775800	-1.74672900

F	8.38887000	-0.38672200	-2.30772200

F	7.63107100	1.90674000	-1.07068900

F	5.67080700	1.89164600	0.82316000

F	2.37446600	6.42576900	-2.35304300

F	0.09376500	7.70385000	-3.10496900

F -2.20976500	7.38004400	-1.72785200

F -2.31567900	5.76355600	0.46912000

F -6.24358000	3.18688000	-2.82098200

F -7.44955300	1.08239200	-4.06373000

F -7.43658800	-1.38984400	-2.94332500

F -6.25224000	-1.82933700	-0.53649600

C -0.21088300	-0.05774200	-1.47791000

C	0.29209800	2.40777300	-2.49416100

C	0.12325900	3.63149300	-3.18754300

C -0.86151300	1.85865100	-1.91488500

C -1.15745900	4.19330100	-3.31109600

H	0.97827200	4.13073200	-3.66348100

C -2.14822800	2.37205400	-2.04601100

H -0.69905100	1.09654300	-0.97007000

C -2.29600700	3.57370000	-2.75483000

H -1.27529500	5.13558500	-3.86823300

H -2.99900400	1.85114900	-1.59244100

H -3.29313100	4.02847200	-2.85142400

C	2.69670200	-3.23524800	-2.20231800

C	1.80403800	-2.22429300	-1.87221000

C	2.11297900	-0.85753200	-2.14933100

C	3.40698000	-0.55751500	-2.71819700

C	4.30259100	-1.61410800	-3.04153000

C	3.94431700	-2.93355300	-2.80284300

H	2.45233200	-4.28063900	-1.97553300

H	0.84771400	-2.47187600	-1.39332900

C	1.14841600	0.20890300	-2.00152400

C	3.71769700	0.81141000	-2.98133500

H	5.27078900	-1.35483800	-3.49145900

H	4.64481000	-3.74596200	-3.04367400

C	2.77638000	1.82125400	-2.86438100

C	1.45611000	1.52954400	-2.43181300

C -1.19370900	-0.83279900	-2.27967500

C -0.82185300	-1.35612600	-3.54521600

C -2.50384200	-1.09566200	-1.80582000

C -1.71070600	-2.14265100	-4.28877900

H	0.18005400	-1.14760700	-3.94462900

C -3.39315100	-1.86815900	-2.56203400

H -2.80784500	-0.70473000	-0.82623600

C -3.00100100	-2.40319900	-3.80136300

H -1.39210800	-2.54994400	-5.26074200

H -4.38732000	-2.10549700	-2.16100000

H -3.69696700	-3.03409200	-4.37330200

C	4.98750100	1.40358500	-3.46595300

O	6.06706800	0.88817100	-3.68597100

O	4.77799200	2.75352000	-3.63589000

C	3.43247600	3.10724000	-3.29485400

H	2.95448200	3.55920800	-4.19214500

H	3.45911600	3.86984100	-2.48656300

***c*-TS-INT3-Rotation**

Rh 0.06693400	-0.24438100	-1.11319700

Rh -0.60819900	-0.65671200	-3.45829600

N	3.87420400	-2.62488900	-1.82285800

O	1.96945700	-3.94857000	-1.49572200

O	5.85475700	-1.44431800	-1.44286700

C	2.96816000	-3.39786200	-1.06684700

C	3.47650300	-3.36471800	0.33566300

C	2.89297700	-3.83293600	1.51011400

C	3.54640000	-3.54784400	2.73066600

C	4.73784200	-2.79998800	2.75313100

C	5.33205000	-2.34772900	1.55176300

C	4.67635200	-2.62528900	0.35504300

C	4.94616400	-2.14280000	-1.03509000

N	0.69727800	4.20984200	-1.87695500

O	2.79249700	4.23774200	-2.94502500

O -0.90742600	4.06588200	-0.16667200

C	2.10661300	4.30219100	-1.94122200

C	2.58133000	4.41453200	-0.52161700

C	3.87303600	4.43447700	0.00404100

C	4.02722600	4.24595200	1.39856200

C	2.90311600	4.09921400	2.23535600

C	1.59942600	4.16265100	1.69838300

C	1.46253600	4.29215100	0.32015900

C	0.24758300	4.18973100	-0.53104400

N -4.26644600	1.65176900	-0.56282700

O -3.19198500	3.12783100	-2.00895200

O -5.17944000	0.67343100	1.35598200

C -3.71803000	2.88865400	-0.93413700

C -3.89481400	3.78287200	0.24744300

C -3.55532900	5.12053300	0.42712900

C -3.76562400	5.69148700	1.70385900

C -4.30160100	4.92465100	2.75640700

C -4.65631400	3.57052000	2.55401000

C -4.45130300	3.01753900	1.29279000

C -4.70597300	1.63446700	0.77750700

N -2.24288700	-4.19598300	-0.19848100

O -3.60133900	-2.66957500	-1.31778900

O -1.19232400	-5.37056600	1.53230000

C -3.35449600	-3.35157600	-0.33719600

C -4.09443600	-3.42662400	0.95318900

C -5.26931400	-2.79698500	1.35324700

C -5.69083000	-2.96573800	2.69212400

C -4.94826200	-3.77305900	3.57600600

C -3.76812300	-4.42520500	3.14794600

C -3.35576300	-4.24411700	1.83103300

C -2.12531700	-4.71264800	1.11261800

O	1.88675400	-1.00263000	-1.67735600

O	1.35641200	-1.15635400	-3.87927100

O	0.32056000	1.69601700	-1.71204400

O -0.39126200	1.37740200	-3.83771400

O -1.93756800	0.31352900	-0.83291600

O -2.52839800	-0.22669000	-2.96510800

O -0.44410400	-2.19054700	-0.73966100

O -0.89383600	-2.61939200	-2.92696300

C	2.12616600	-1.33653100	-2.89027800

C	3.52553800	-1.96833800	-3.08676900

C	3.79244600	-2.79896200	-4.38417000

C	5.19241400	-3.43284000	-4.24747500

C	2.74521100	-3.90306500	-4.60990300

C	3.80495300	-1.82646700	-5.58318000

C -0.05819700	2.11445200	-2.86344200

C -0.19608300	3.65339700	-2.90967100

C -0.27281400	4.36632100	-4.29962900

C	0.77873500	3.88229200	-5.31723800

C -0.12366700	5.88208100	-4.06261500

C -1.68597600	4.08751000	-4.86479900

C -2.78179000	0.16824800	-1.78655000

C -4.24876900	0.45825300	-1.40283100

C -5.35413300	0.44286500	-2.52119700

C -5.49060800	-0.99097200	-3.07685100

C -6.68987600	0.81817300	-1.84171000

C -5.08274200	1.41879500	-3.68267500

C -0.82325000	-2.94420300	-1.70315900

C -1.21298900	-4.36056300	-1.22661200

C -1.56362100	-5.44561100	-2.29610700

C -0.33492500	-5.64992700	-3.20496500

C -1.84232900	-6.76373900	-1.54195000

C -2.79453900	-5.07997000	-3.14690400

H -4.50695300	-0.36670400	-0.70533100

H -6.91106300	0.15353100	-0.98197700

H -7.51889800	0.72337900	-2.57141800

H -6.68995900	1.86513400	-1.47582700

H -5.87567700	1.29651000	-4.44910600

H -5.09187700	2.47511300	-3.35561800

H -4.10587200	1.22015700	-4.16126600

H -6.30850300	-1.01290100	-3.82566600

H -4.55961000	-1.33238200	-3.56394900

H -5.73357700	-1.71187200	-2.27313800

H -1.86224100	3.00158700	-4.98754200

H -1.79368400	4.56906700	-5.85769400

H -2.47596100	4.48397500	-4.19609300

H -0.83946200	6.24481300	-3.29562100

H -0.32885800	6.42887900	-5.00496300

H	0.90195200	6.14755900	-3.73803300

H	0.65161900	2.80479000	-5.53690200

H	1.80698500	4.05077100	-4.95116500

H	0.64833200	4.43789400	-6.26856400

H -1.20390400	3.78833700	-2.45061800

H	2.82252600	-1.33827200	-5.72162500

H	4.05175800	-2.37989400	-6.51168200

H	4.57360800	-1.03586200	-5.45176700

H	2.72152600	-4.62069200	-3.76830600

H	2.98847000	-4.46379100	-5.53542100

H	1.72965500	-3.47878400	-4.72147000

H	5.96707000	-2.67177400	-4.02210200

H	5.46871500	-3.93900300	-5.19408200

H	5.21777600	-4.19184800	-3.43996200

H	4.22672000	-1.10195500	-3.12453900

H -3.71319800	-5.00198500	-2.53292600

H -2.65421300	-4.11838100	-3.67326600

H -2.96782300	-5.87423700	-3.90182700

H -0.52848800	-6.48434700	-3.90907000

H -0.11228800	-4.74366500	-3.79692700

H	0.56795400	-5.89885700	-2.61289800

H -2.74630400	-6.68900700	-0.90365100

H -0.99143900	-7.05462900	-0.89344500

H -2.01878100	-7.58138800	-2.26940100

H -0.31894600	-4.71735700	-0.67198400

F -3.04791800	5.87901400	-0.53832400

F -3.44722000	6.96534100	1.92501700

F -4.47533300	5.48799200	3.95072200

F -5.14224400	2.87222300	3.58121000

F	0.54919200	4.02833900	2.51485200

F	3.08237700	3.87843300	3.53908500

F	5.24245000	4.16982900	1.93303400

F	4.95884900	4.56677400	-0.74900900

F	6.44911000	-1.63190000	1.62214400

F	5.29142800	-2.48976600	3.92583000

F	3.00231700	-3.93897200	3.88529400

F	1.73581700	-4.48928400	1.53685800

F -3.07502800	-5.14217000	4.03132200

F -5.35926000	-3.90493300	4.83608200

F -6.79648900	-2.37043400	3.12673800

F -6.00299000	-2.04891200	0.53279200

C	0.72954500	0.10638400	0.77363400

C	1.29195400	0.15906700	3.91083000

C	0.57750100	1.25793300	4.43317300

C	1.31587000	-1.04208100	4.65578700

C -0.10632000	1.15059200	5.65374400

H	0.57598100	2.20897300	3.88580000

C	0.62686400	-1.14907000	5.87094300

H	1.87550600	-1.90770900	4.27548700

C -0.08548900	-0.05049100	6.37799100

H -0.65923200	2.02045500	6.03993500

H	0.64899600	-2.10062600	6.42353700

H -0.62165500	-0.13057500	7.33565900

C	4.21623700	1.57556200	-1.62481500

C	3.10479200	1.24466500	-0.85219700

C	3.20024600	0.87161800	0.51179100

C	4.53252400	0.97591100	1.07640100

C	5.66445100	1.31047000	0.28021600

C	5.51368000	1.58171100	-1.06954500

H	4.06288400	1.87726100	-2.67178200

H	2.12165800	1.31897300	-1.31026300

C	2.02814600	0.46656000	1.33909100

C	4.66651200	0.79132000	2.47470800

H	6.64347200	1.35234500	0.77572200

H	6.38610400	1.83038800	-1.69032800

C	3.56966900	0.51184200	3.27054500

C	2.25269100	0.35662000	2.77258900

C -0.39227300	-0.22584900	1.66083000

C -1.35435500	0.75395000	2.01082400

C -0.53724900	-1.54152600	2.16340200

C -2.39154300	0.43289500	2.89390400

H -1.25549300	1.77406300	1.60926500

C -1.56578000	-1.84185200	3.06361900

H	0.18510500	-2.31828700	1.87690200

C -2.49624100	-0.85890400	3.43538700

H -3.12162500	1.19987300	3.18744200

H -1.60949000	-2.84785700	3.50520300

H -3.29768000	-1.09184100	4.15288800

C	5.86664600	0.94314600	3.34864600

O	7.01894600	1.18525400	3.06738800

O	5.44375200	0.76255100	4.64781200

C	4.04019100	0.48782100	4.70356000

H	3.53165500	1.26139700	5.31891000

H	3.88422000	-0.49285200	5.19877100
